# Clinical trial landscape and translational priorities for adoptive T-cell and NK-cell therapies in gynecologic malignancies

**DOI:** 10.3389/fimmu.2026.1936815

**Published:** 2026-09-28

**Authors:** Qingyu Chen, Yuxin Zhu, Shilin Ye, Yaoyi Wang, Huilong Lin

**Affiliations:** 1 The Second School of Medicine, Wenzhou Medical University, Wenzhou, Zhejiang, China; 2 Department of Gynecology, The First Affiliated Hospital of Wenzhou Medical University, Wenzhou, Zhejiang, China; 3 School of Laboratory Medicine and Life Sciences, Wenzhou Medical University, Wenzhou, Zhejiang, China

**Keywords:** adoptive cellular therapy, CAR-T, clinical trials, gynecologic malignancies, natural killer cells, registry-based landscape, TCR-T, tumor-infiltrating lymphocytes

## Abstract

**Background:**

Adoptive T-cell and natural killer (NK)-cell therapies are expanding across solid tumors, but their registered development in gynecologic malignancies remains difficult to interpret because many studies use basket designs and registry records vary in detail.

**Methods:**

We conducted a cross-sectional analysis of therapeutic adoptive T-cell and NK-cell trial records identified from TrialTrove, ClinicalTrials.gov, and the WHO International Clinical Trials Registry Platform. Trials were classified into three mutually exclusive evidence-specificity groups: single gynecologic disease family specific, multiple gynecologic disease families only, and broader baskets including non-gynecologic cancers. We evaluated source sensitivity, target applicability, field-specific registry visibility, and the availability of posted registry safety results. A complementary registry-informed exploratory survey of 25 experts assessed translational barriers and development priorities using domain-specific 1–5 scales.

**Results:**

Among 442 unique records screened, 211 therapeutic trials met the eligibility criteria. Of these, 43 were restricted to a single gynecologic disease family, 2 included multiple gynecologic disease families only, and 166 were broader basket trials permitting enrollment of non-gynecologic malignancies. The public-registry-linked and public-search-captured sensitivity subsets included 200 and 141 records, respectively, with broadly similar structural patterns across datasets. The landscape was predominantly early phase, with 149 Phase I and 50 Phase I/II trials. CAR-T, TIL, and TCR-T were the leading modalities, accounting for 79, 60, and 42 trials, respectively. Only 20 records had posted registry results with an adverse-event module, indicating limited availability of registry-reported safety data. In the exploratory expert survey, an immunosuppressive tumor microenvironment and a lack of gynecologic-specific cohorts were identified as leading barriers, whereas ovarian cancer, TIL therapy, MSLN, target reporting, and registry completeness emerged as prominent development priorities.

**Conclusions:**

Registered cellular-therapy activity in gynecologic malignancies is broad but remains early phase, largely basket-derived, and unevenly visible in registry fields. Separating disease-specific trials from broader eligibility-only baskets, testing source sensitivity, and distinguishing modality biology from missing target reporting provide a more reproducible basis for future trial planning.

## 1 Introduction

Gynecologic malignancies comprise a heterogeneous group of cancers, including ovarian, cervical, endometrial, vulvar, vaginal, fallopian tube, and primary peritoneal cancers (1). In 2022, cervical, endometrial, and ovarian cancers together accounted for more than 1.4 million new cases and more than 650,000 deaths worldwide, representing roughly 15% of all cancer deaths among women (2). In US population data, five-year relative survival for distant-stage disease remains approximately one-fifth for cervical and endometrial cancers and one-third for ovarian cancer (3). These diseases do not represent a single biological or clinical entity: they differ in epidemiology, molecular drivers, treatment pathways, recurrence patterns, immune context, and prognosis (4–6). Immune checkpoint inhibition has changed selected cervical and endometrial cancer settings (7–10), yet clinical benefit remains uneven and many patients with advanced or recurrent disease require additional options. These advances show that antitumor immunity can be engaged therapeutically, but they do not resolve disease-specific immune escape or the barriers encountered by cellular products in solid tumors.

Adoptive cellular therapies encompass CAR-T cells, TCR-engineered T cells, tumor-infiltrating lymphocytes, NK-cell therapies, CAR-NK products, and other T- or NK-cell-based approaches (11–13). Unlike conventional systemic agents, these interventions introduce or expand living immune-cell products and therefore require clear product definition, manufacturing, conditioning, dosing, and longitudinal monitoring. Their translation in solid tumors remains constrained by antigen heterogeneity, on-target off-tumor risk, restricted trafficking and infiltration, limited persistence, immunosuppressive tumor microenvironments, manufacturing complexity, and toxicity-monitoring requirements (14, 15). These interacting barriers make early trial design and transparent reporting particularly important.

Development pathways also differ across cellular platforms. Engineered NK cells and other innate immune-cell approaches broaden the field beyond conventional T-cell products and may support distinct manufacturing and safety strategies (16, 17). Engineered T-cell approaches directed against viral and non-viral epithelial cancer antigens provide another route to disease-specific development (18, 19). These contrasting routes illustrate why disease type, antigen source, and product modality should be examined together rather than treated as interchangeable categories.

Broader solid-tumor CAR-T literature further emphasizes that target selection, tissue trafficking, local immune suppression, persistence, and product engineering must be addressed together rather than sequentially (20–23). These constraints influence not only cell design but also combination strategies, endpoint selection, safety surveillance, biomarker plans, and long-term follow-up. *In vivo* CAR engineering may eventually alter how cellular products are generated and delivered, but its clinical translation remains early (24, 25). Accordingly, a trial count alone cannot determine whether apparent gynecologic activity is disease-focused, driven by broader basket eligibility, robust to database source, or sufficiently documented for external interpretation.

Gynecologic cellular therapy development also intersects with regulatory T-cell biology, CD8 T-cell response dynamics, and T-cell senescence within the tumor microenvironment (26–28). Existing summaries often focus on individual modalities, selected targets, or early trial narratives. A registry-based analysis can address a complementary question by showing whether activity is disease-specific or basket-based, which modalities and targets have entered clinical development, and how consistently key design fields are reported. However, registry interpretation depends on complete and timely records, and empirical audits have identified persistent limitations in registration quality and results reporting (29–31). Registry data describe what is being tested, whereas expert ratings can support exploratory priority setting but should not be treated as independent validation of registry findings.

We therefore conducted a cross-sectional landscape analysis of adoptive T-cell and NK-cell therapies in gynecologic malignancies using TrialTrove, ClinicalTrials.gov, and WHO ICTRP, together with a registry-informed exploratory expert survey. The objectives were to distinguish disease-specific from broader basket activity, quantify the contribution of each data source, test whether major findings were robust in public-registry-linked and public-search-captured subsets, separate named-target comparisons from antigen-agnostic modalities and missing target reporting, evaluate field-specific registry visibility and posted safety evidence, and identify priorities for future translation. The study was descriptive and did not pool patient-level response, survival, toxicity incidence, or comparative treatment-effect outcomes.

## 2 Methods

### 2.1 Study design and data sources

This study was designed as a clinical trial registry/database-based cross-sectional landscape analysis of therapeutic adoptive T-cell and NK-cell approaches in gynecologic malignancies. The unit of analysis was the deduplicated clinical trial record, not the individual patient or disease-specific cohort. The analysis evaluated evidence specificity, source sensitivity, target applicability, registry-field visibility, and safety-evidence availability; it was not designed as an efficacy or toxicity meta-analysis.

Clinical trial records were identified from TrialTrove (exported June 23, 2026), ClinicalTrials.gov (exported June 25, 2026), and WHO ICTRP (exported July 1, 2026). TrialTrove and ClinicalTrials.gov were the primary discovery sources, while WHO ICTRP was used for supplementary public-registry reconciliation. Trials with reported study start dates after June 1, 2026, were excluded from the analytic dataset; the later export dates were used only to complete source reconciliation.

After merging 570 source records, removing 128 duplicates, and applying the therapeutic eligibility criteria, 211 unique trial records were retained in the primary analytic landscape. Two source-sensitivity sets were defined for this analysis: a public-registry-linked subset of 200 records with a public registry identifier and a public-search-captured subset of 141 records actually retrieved by this study’s ClinicalTrials.gov or WHO ICTRP searches.

### 2.2 Search strategy and eligibility criteria

Clinical trial records were identified through source-specific searches combining terms for gynecologic malignancies with terms for adoptive cellular therapies. The exact TrialTrove strategy, public-registry search strings, export dates, and freeze rule are provided in **Supplementary Table S1**. Eligible therapy approaches included CAR-T, TCR-T, tumor-infiltrating lymphocyte therapy, NK-cell therapy, CAR-NK therapy, and other adoptive T- or NK-cell approaches.

Trials were eligible if an adoptive T-cell or NK-cell product was administered therapeutically for an invasive gynecologic malignancy or if a broader malignant-tumor basket explicitly allowed at least one eligible gynecologic disease family. Observational, diagnostic, screening-only, long-term follow-up-only, cell-collection-only, preinvasive-lesion, and non-therapeutic immune-phenotyping records were excluded. Basket eligibility was determined from the condition and eligibility tumor-type list rather than the title alone.

Records were excluded for an ineligible cellular intervention (n = 162), no explicit eligible gynecologic cohort (n = 55), a non-interventional or non-therapeutic ACT purpose (n = 9), a reported start date after June 1, 2026 (n = 3), or a preinvasive lesion rather than invasive malignancy (n = 2). Q.C. and Y.Z. jointly screened and reviewed all records, and questions or unresolved disagreements were referred to H.L. for adjudication. Duplicate and potentially conflicting records were reviewed using public registry identifiers, protocol identifiers, titles, sponsors, interventions, disease eligibility, phase, status, and enrollment. Because the source files did not retain a prospective field-by-field conflict log, no universal source-precedence rule was inferred retrospectively.

### 2.3 Data extraction and variable standardization

For each eligible record, variables were extracted for discovery source, public-registry linkage, registry identifier, title, disease eligibility, evidence-specificity architecture, cellular modality, target-reporting state, phase, status, sponsor, center type, country, planned sample size, treatment backbone and antitumor combinations, route, endpoints, posted results, adverse-event availability, and registry-field visibility. The full list of the 211 included therapeutic trial records is provided in **Supplementary Table S2**.

Evidence specificity was classified using mutually exclusive eligibility rules. A single gynecologic disease family-specific trial allowed one gynecologic family only; tubo-ovarian, fallopian-tube, and primary-peritoneal cancers were treated as one family. A gynecologic multicancer-only trial allowed two or more gynecologic disease families and no non-gynecologic cancer. A broader basket trial allowed at least one non-gynecologic malignancy. Disease-eligibility summaries were separately analyzed as non-mutually-exclusive multi-label variables.

Target information was harmonized into four conceptually distinct states. Named target was used when a shared, viral, mutation-directed, or patient-specific target was explicit; named-target frequency comparisons used only this subset. No predefined antigen denoted antigen-agnostic modality biology, primarily TIL and non-engineered NK approaches. Not reported denoted absent registry target information, while unknown was reserved for wording that did not support reliable classification.

### 2.4 Registry field visibility and source sensitivity

Field-specific visibility was assessed for six generally applicable registry elements: interpretable target or mechanism, a cell-product name or cellular descriptor, route of administration, positive planned sample size, primary-endpoint text, and follow-up duration. A proprietary product name was not required when a usable cellular-product descriptor was present. Endpoint and monitoring indicators were coded as registry-visible only when the registry endpoint or monitoring text explicitly named safety as a primary endpoint, dose-limiting toxicity or maximum tolerated dose (DLT/MTD), objective response rate (ORR), progression-free survival (PFS), overall survival (OS), cytokine release syndrome (CRS), immune effector cell-associated neurotoxicity syndrome (ICANS), or cellular persistence. DLT/MTD visibility used Phase I and Phase I/II records as the denominator (n = 199 overall: CAR-T, 77; TIL, 55; TCR-T, 40; NK, 15; CAR-NK, 8; other ACT, 4), whereas all other endpoint and monitoring indicators used the full modality-specific therapeutic landscape. Registry nonmention was treated as lack of public visibility, not proof that the item was absent from the full protocol.

Each field was reported separately as n/N and percentage; the previous equal-weight composite completeness score was removed from the primary analysis because the fields differ in applicability and interpretive importance. Source sensitivity was evaluated by repeating the same structural summaries in the 211-record full landscape, the 200-record public-registry-linked subset, and the 141-record public-search-captured subset.

### 2.5 Registry safety-evidence availability

For included ClinicalTrials.gov records with posted results, we extracted whether an adverse-event module was available and grouped reported serious-event terms into broad clinical domains. This analysis describes evidence availability only: no adverse-event incidences were pooled, no event was attributed solely to the cellular product, and no cross-modality rate comparison was performed because populations, conditioning regimens, concomitant treatments, grading systems, and ascertainment differed. Current FDA-approved CAR-T, TCR-T, and TIL products were used only as qualitative external safety benchmarks in the Discussion.

### 2.6 Statistical analysis and visualization

Descriptive statistics were used throughout. Counts and percentages used 211 eligible trial records as the denominator unless otherwise specified; disease-eligibility percentages were non-mutually exclusive, named-target distributions used the 101 named-target records, and posted safety-evidence summaries used the 20 records with registry results. Ordinal survey items were summarized primarily with medians, interquartile ranges, full response distributions, and top-box proportions (scores 4-5).

Visualizations retained the original analytic structure where possible and were updated only for the revised denominator and reviewer-requested definitions. The three-level evidence-specificity architecture, treatment-backbone versus antitumor-combination distinction, field-specific visibility, and source-sensitivity analysis were incorporated without introducing any supplementary figures.

Because this study used publicly available clinical trial registry records and did not involve individual patient-level data, ethics approval and informed consent were not required.

### 2.7 Registry-informed exploratory expert survey

An exploratory cross-sectional expert survey was conducted from June to July 2026 after preliminary registry mapping. Respondents reported relevant clinical or research experience in gynecologic oncology, cellular therapy, tumor immunology, translational medicine, or clinical trial design. The collected files did not preserve a complete recruitment log or formal eligibility-screening record, so the recruitment channel and eligibility-verification procedure could not be reconstructed reliably. The number invited, institution-level affiliations, and institution count were not collected, so a response rate and institutional distribution could not be calculated. Participation was voluntary and anonymous, written informed consent was obtained from all respondents, and 25 complete questionnaires with no item-level missing data were included. The full bilingual questionnaire and all item-level results are provided in **Supplementary Table S3**.

The survey included three registry-derived current-state items (Q6-Q8) before the barrier and priority ratings; because this ordering could prime responses, the survey was treated as a registry-informed priority-setting exercise rather than independent validation. The questionnaire was not a psychometrically validated scale, and no separate pilot-validation dataset was generated. No item specifically ascertained Phase I/II trial experience. Open-text responses to Q14-Q16 were reproduced verbatim in **Supplementary Table S3**; no formal qualitative thematic analysis was undertaken, and these responses were not used to generate quantitative conclusions. No inferential testing was performed. The survey collected professional opinions only and was exempted from formal ethics review by the Ethics Committee of Wenzhou Medical University; no exemption reference number was issued.

## 3 Results

### 3.1 Study selection and dataset construction

The raw sources contained 201 TrialTrove records, 330 ClinicalTrials.gov records, and 39 WHO ICTRP records (**Figure 1**). After merging 570 source records and removing 128 duplicates, 442 unique records were screened. Eligibility screening excluded 231 records, leaving 211 therapeutic ACT trial records for the primary landscape analysis.

**Figure 1:**
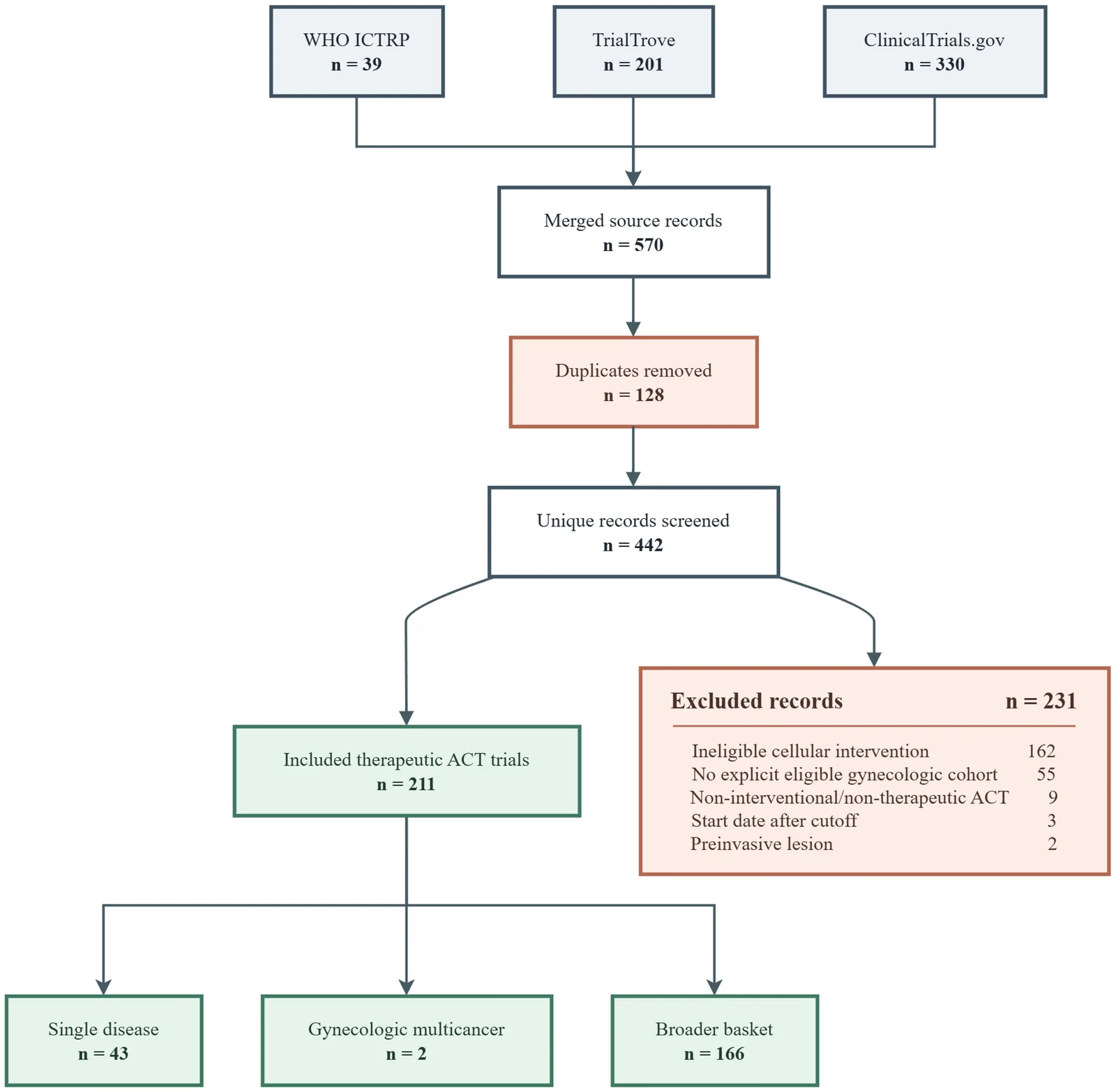
Registry screening flow. TrialTrove, ClinicalTrials.gov, and WHO ICTRP yielded 570 source records, of which 128 duplicates were removed and 442 unique records were screened. The 231 exclusions comprised an ineligible cellular intervention (n = 162), no explicit eligible gynecologic cohort (n = 55), a non-interventional or non-therapeutic ACT purpose (n = 9), a reported start date after the analytic cutoff (n = 3), and a preinvasive lesion rather than invasive malignancy (n = 2). The 211 eligible therapeutic records were classified as single gynecologic disease family-specific (n = 43), gynecologic multicancer-only (n = 2), or broader basket (n = 166).

The primary landscape comprised 43 single gynecologic disease family-specific trials, 2 gynecologic multicancer-only trials, and 166 broader baskets including non-gynecologic cancers. Seventy included records were discovered only through the TrialTrove search; 59 of these had public registry identifiers and 11 did not. The public-registry-linked and public-search-captured sensitivity subsets contained 200 and 141 records, respectively, and the leading scope, modality, target-state, and disease-eligibility proportions were broadly similar across the three sets (**Table 1**).

**Table 1:** Source-sensitivity analysis of principal structural findings.

Metric	Full analytic landscape (N = 211)	Public-registry-linked subset (N = 200)	Public-search-captured subset (N = 141)
Broader basket including non-gynecologic cancers	166 (78.7%)	161 (80.5%)	104 (73.8%)
Gynecologic-restricted trials	45 (21.3%)	39 (19.5%)	37 (26.2%)
CAR-T	79 (37.4%)	73 (36.5%)	45 (31.9%)
TIL	60 (28.4%)	58 (29.0%)	35 (24.8%)
TCR-T	42 (19.9%)	39 (19.5%)	33 (23.4%)
Named target	101 (47.9%)	97 (48.5%)	68 (48.2%)
Target not reported or unknown	41 (19.4%)	36 (18.0%)	28 (19.9%)
Tubo-ovarian/peritoneal eligibility	164 (77.7%)	154 (77.0%)	109 (77.3%)
Cervical eligibility	79 (37.4%)	77 (38.5%)	48 (34.0%)
Endometrial/uterine-corpus eligibility	39 (18.5%)	37 (18.5%)	19 (13.5%)
Registry results posted	20 (9.5%)	20 (10.0%)	20 (14.2%)

The public-registry-linked subset includes records with a public registry identifier; the public-search-captured subset includes records actually retrieved by this study’s ClinicalTrials.gov or WHO ICTRP searches.

### 3.2 Temporal evolution and global distribution

Among 204 records with a reported study start date, trial activity increased after the mid-2010s and was concentrated after 2017 (**Figure 2a**); 2026 was partial through the June 1 start-date cutoff. The annual profile included CAR-T, TCR-T, TIL, NK, and CAR-NK approaches, indicating a broadening clinical development space rather than a single-modality program.

**Figure 2:**
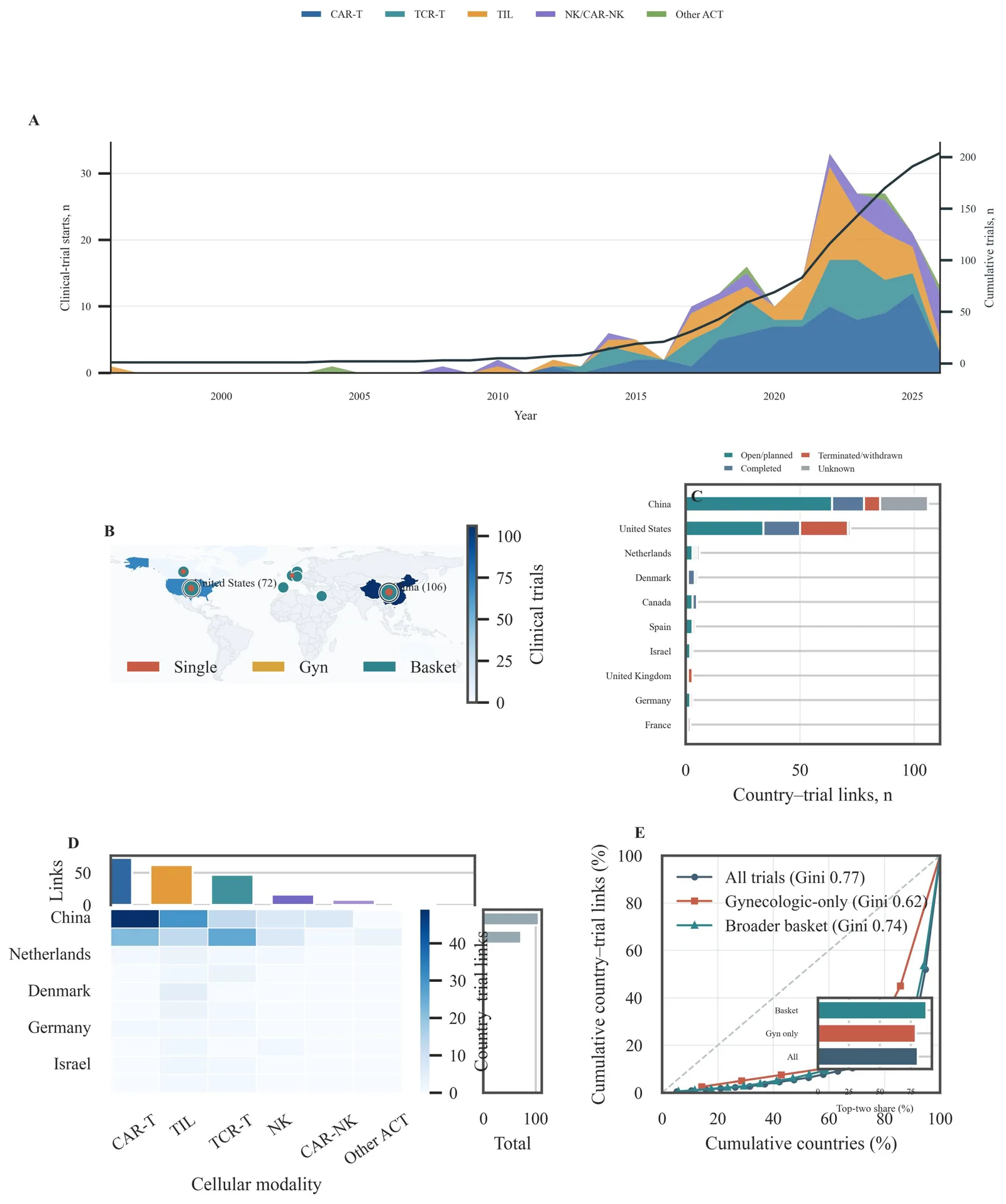
Temporal and geographic clinical-trial landscape. **(A)** Annual clinical-trial starts by modality and cumulative trial starts. This panel uses the 204 records with a reported start date; 2026 is partial through the June 1 cutoff. **(B)** Country-level clinical-trial distribution with evidence-specificity overlays; 14 records without country information were excluded, and multicountry trials contribute once to each listed country. **(C)** Status composition in the ten most active countries. **(D)** Country-by-modality matrix with row and column totals. **(E)** Geographic concentration curves and top-two-country shares.

Country-level mapping showed concentrated clinical activity (**Figures 2b, c**). China and the United States accounted for 106 and 72 registry records, respectively; 14 records lacked country information, and multicountry trials contributed once to each listed country.

### 3.3 Clinical development maturity

The clinical development landscape was dominated by early-phase trials and broader baskets (**Figure 3**, **Table 2**). Phase I trials accounted for 149 (70.6%) records and Phase I/II trials for 50 (23.7%); only 6 records were Phase II. Recruiting trials accounted for 100 (47.4%), completed trials for 35 (16.6%), and terminated trials for 27 (12.8%).

**Figure 3:**
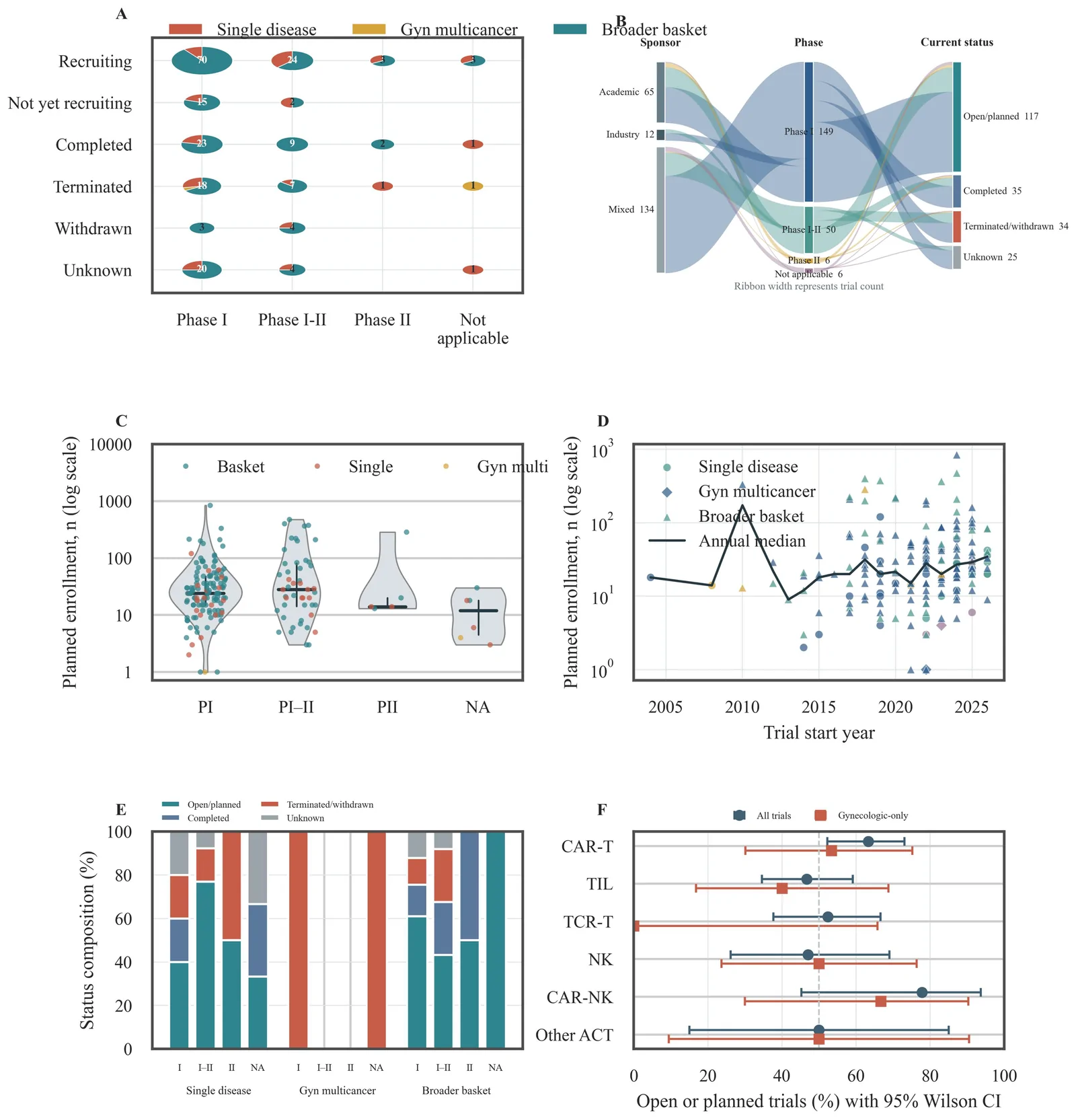
Clinical development maturity map. **(A)** Phase-status cells, with bubble area proportional to trial count and sectors indicating the mutually exclusive evidence-specificity architecture. **(B)** Sponsor-to-phase-to-status alluvial map. **(C)** Planned enrollment distributions by phase; points indicate individual trials and color indicates evidence specificity. **(D)** Planned enrollment over time; point color indicates phase and shape indicates evidence specificity. **(E)** Status composition across evidence-specificity and phase strata. **(F)** Open/planned proportions by modality with 95% Wilson confidence intervals for the full landscape and gynecologic-restricted subset.

**Table 2:** Characteristics of the 211 included therapeutic clinical trial records.

Section	Category	n	Percent
Evidence-specificity architecture	Broader basket including non-gynecologic cancers	166	78.7
	Single gynecologic disease family specific	43	20.4
	Multiple gynecologic disease families only	2	0.9
Discovery source	Both	84	39.8
	TrialTrove	70	33.2
	ClinicalTrials.gov	55	26.1
	WHO ICTRP	2	0.9
Cell modality	CAR-T	79	37.4
	TIL	60	28.4
	TCR-T	42	19.9
	NK	17	8.1
	CAR-NK	9	4.3
	Other ACT	4	1.9
Development phase	Phase I	149	70.6
	Phase I-II	50	23.7
	Not applicable	6	2.8
	Phase II	6	2.8
Recruitment/status	Recruiting	100	47.4
	Completed	35	16.6
	Terminated	27	12.8
	Unknown	25	11.8
	Not yet recruiting	17	8.1
	Withdrawn	7	3.3
Target reporting state	Named target	101	47.9
	No predefined antigen (modality biology)	69	32.7
	Not reported (registry visibility)	39	18.5
	Unknown	2	0.9
Gynecologic disease eligibility (multi-label)	tubo-ovarian/peritoneal	164	77.7
	cervical	79	37.4
	endometrial/uterine corpus	39	18.5
	vulvar	20	9.5
	vaginal	19	9.0
Sponsor group	Mixed	134	63.5
	Academic	65	30.8
	Industry	12	5.7
Center type	Single-center	170	80.6
	Multicenter single-country	31	14.7
	Multinational	10	4.7
Sample size group	<30	114	54.0
	30-100	64	30.3
	>100	20	9.5
	Unclear	13	6.2

Percentages use the 211-record primary landscape as the denominator unless otherwise specified; disease-eligibility categories are non-mutually exclusive.

Cellular modality was unevenly distributed. CAR-T was the most common modality with 79 trials, followed by TIL with 60 and TCR-T with 42. NK, CAR-NK, and other ACT approaches accounted for 17, 9, and 4 records, respectively.

### 3.4 Disease-modality-target registry structure

Disease eligibility was heavily influenced by broader baskets and was therefore analyzed as a non-mutually-exclusive multi-label variable. Tubo-ovarian/peritoneal, cervical, and endometrial/uterine-corpus eligibility appeared in 164, 79, and 39 records, respectively. Among the 43 trials restricted to a single gynecologic disease family, 36 were tubo-ovarian/peritoneal, 6 cervical, and 1 endometrial/uterine-corpus; two additional trials allowed multiple gynecologic disease families but no non-gynecologic cancer. Thus, broad-basket eligibility accounted for much of the apparent disease activity.

Target annotation reflected both engineered and antigen-agnostic strategies. A named target was reported in 101 trials, 69 used a modality without a predefined antigen, 39 did not report a target, and 2 remained unknown. Within the named-target subset, MSLN was the most frequent category (40 trials), followed by MUC16 (11), NY-ESO-1 (10), and HPV E6/E7 and patient-specific targets (9 each) (**Figure 4**). Separating modality biology from missing registry information prevented antigen-agnostic TIL or NK approaches from being treated as unreported targets.

**Figure 4:**
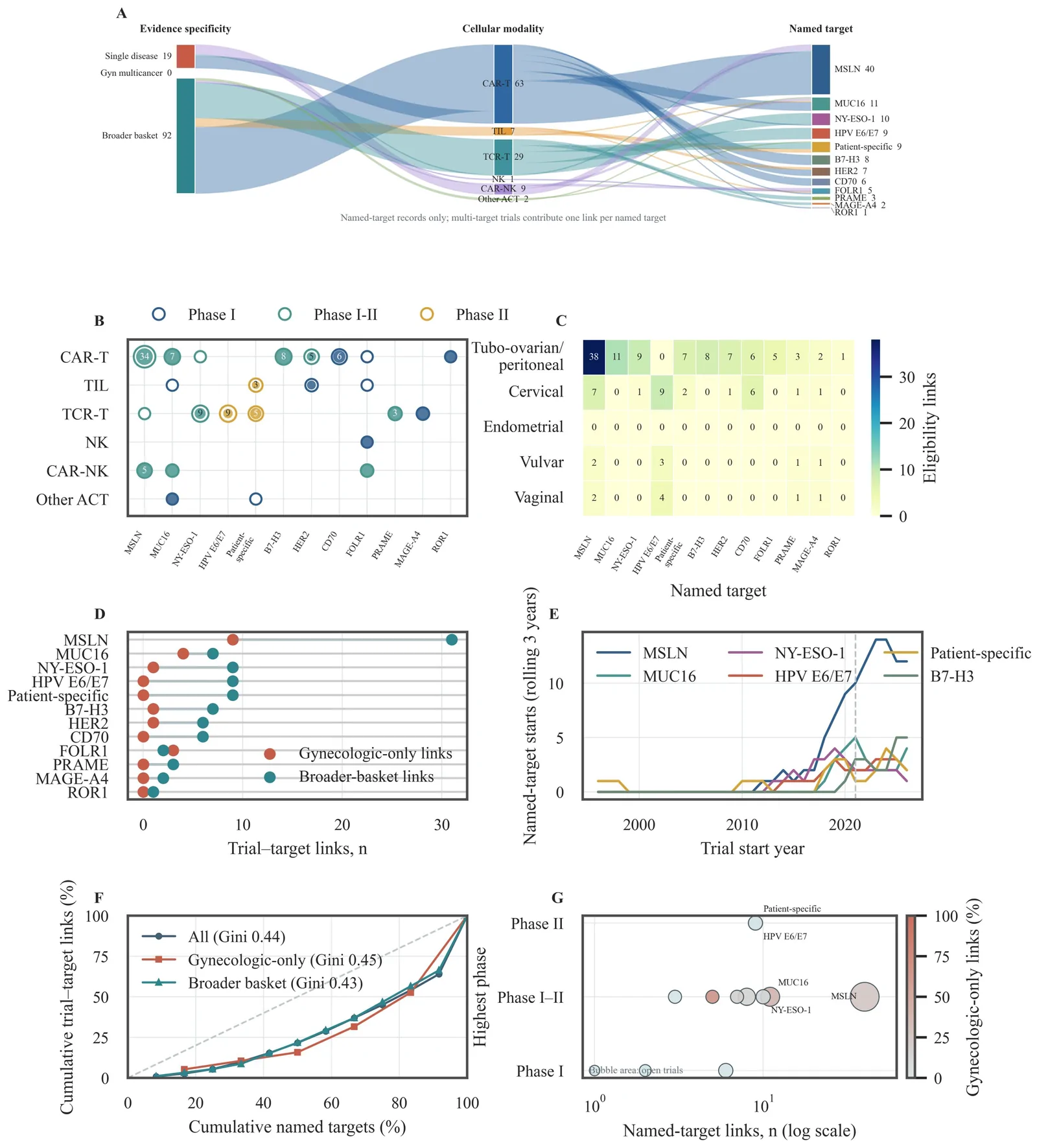
Named-target development landscape. Analyses use the 101 records with an explicit named target; multi-target records contribute one link per named target. **(A)** Evidence specificity-to-modality-to-target alluvial map. **(B)** Modality-target matrix; outer circle size represents trial-target links, inner fill represents open trials, and color indicates highest phase. **(C)** Disease-family eligibility by target; counts are eligibility links and must not be interpreted as disease-specific clinical evidence. **(D)** Named-target links in gynecologic-restricted versus broader-basket trials. **(E)** Rolling three-year target-specific trial starts. **(F)** Target-concentration curves. **(G)** Target maturity by evidence volume, highest phase, open-trial count, and gynecologic-restricted share.

### 3.5 Treatment backbone, antitumor combinations, endpoints, and monitoring

Preparative and supportive components were separated from true antitumor combinations (**Figure 5**). Lymphodepletion and IL-2 support were reported in 147 and 85 trials, respectively. Explicit antitumor combinations included immune checkpoint inhibition in 56 trials, antiangiogenic therapy in 14, PARP inhibition in 11, antitumor chemotherapy in 2, and local radiotherapy in 1. This distinction avoids interpreting conditioning chemotherapy or total-body irradiation as concurrent antitumor treatment.

**Figure 5:**
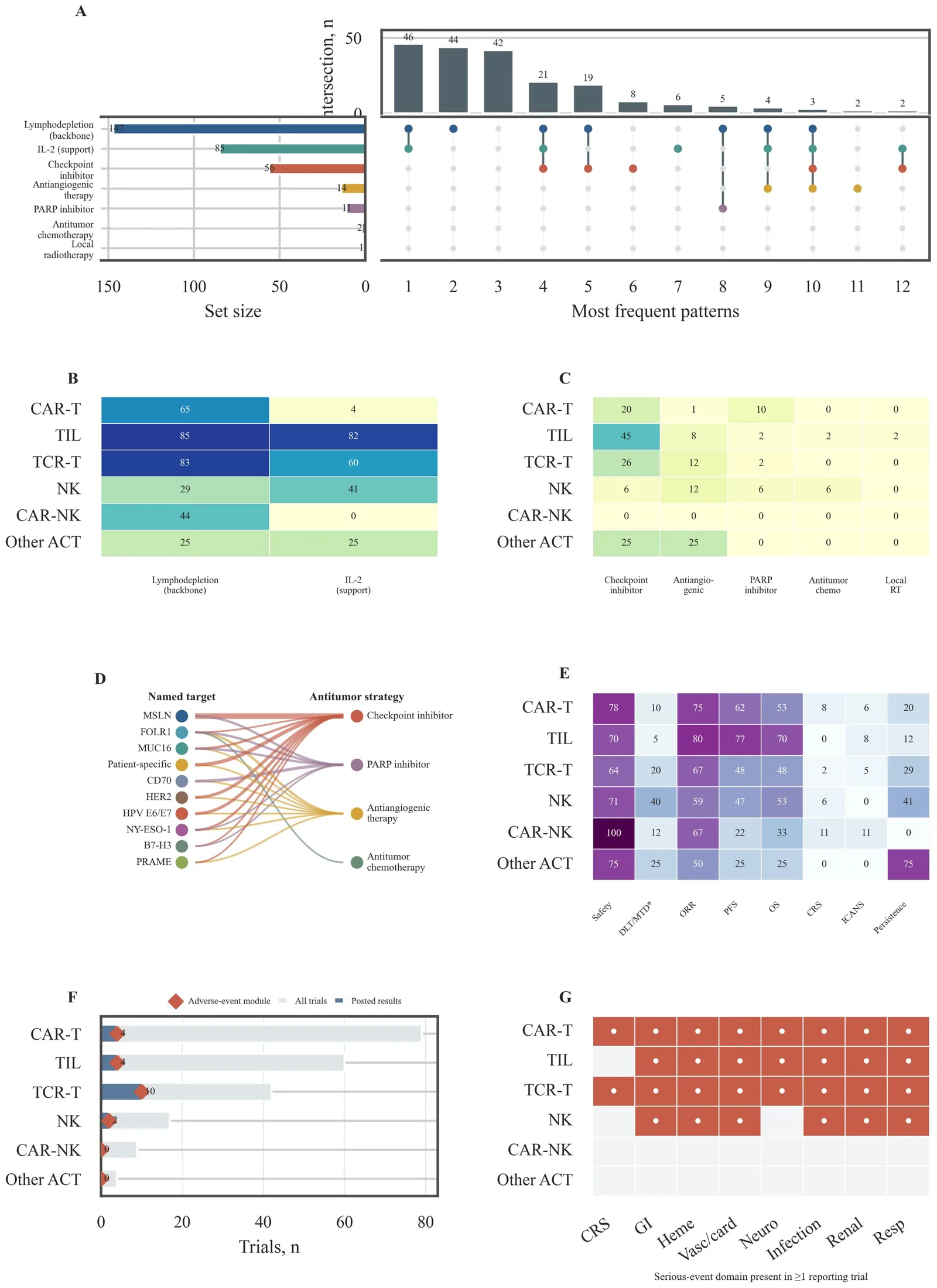
Treatment architecture, endpoints, and registry safety evidence. **(A)** Frequent combinations of preparative, supportive, and antitumor strategies. **(B)** Lymphodepletion and IL-2 support by modality. **(C)** Explicit antitumor combinations by modality. **(D)** Named-target-to-antitumor-strategy links. **(E)** Registry visibility of endpoints and monitoring terms by modality; DLT/MTD proportions use Phase I and Phase I/II denominators (27/199 overall), whereas all other proportions use the full modality-specific therapeutic landscape. Values indicate explicit registry text only. **(F)** Availability of posted results and adverse-event modules. **(G)** Presence of broad serious-event domains in at least one reporting trial. **(F, G)** describe evidence availability only; event rates were not pooled or compared across modalities.

Endpoint selection was heterogeneous across modalities. Among the 199 Phase I or Phase I/II records, DLT/MTD terminology was registry-visible in 27 (13.6%); modality-specific proportions in **Figure 5e** use the corresponding early-phase denominators. Safety and objective-response endpoints were common, whereas progression-free survival, overall survival, cell persistence, immune biomarkers, quality of life, CRS, and ICANS were less consistently visible. These are registry-field observations and do not establish that unreported protocol-level monitoring was absent.

### 3.6 Registry field visibility

Field-specific reporting replaced the previous equal-weight composite score (**Figure 6**). A cell-product name or usable cellular descriptor was present in 211/211 records (100.0%); positive planned sample size in 193 (91.5%); follow-up duration in 188 (89.1%); primary-endpoint text in 187 (88.6%); route in 184 (87.2%); and an interpretable target or mechanism in 170 (80.6%).

**Figure 6:**
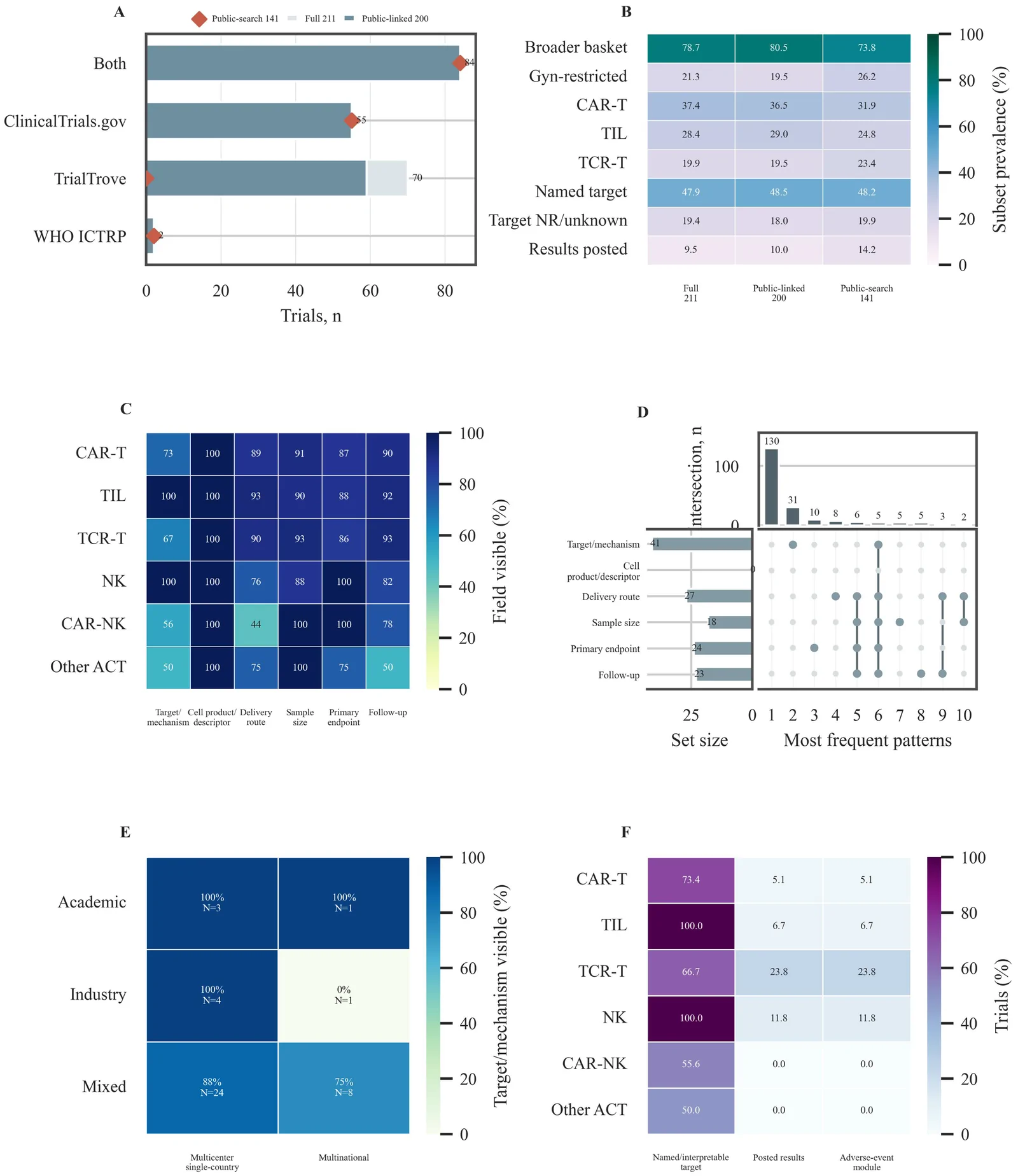
Source sensitivity and field-specific registry visibility. **(A)** Discovery-source composition in the 211-record full landscape, 200-record public-registry-linked subset, and 141-record public-search-captured subset. **(B)** Stability of core structural findings across these subsets. **(C)** Visibility of six generally applicable registry fields by modality; the former equal-weight composite score was removed. **(D)** Co-missingness patterns across the six fields. **(E)** Target/mechanism visibility by sponsor and center structure. **(F)** Target interpretability, posted results, and adverse-event-module availability by modality.

Visibility differed by modality, particularly for target/mechanism and route. The source-sensitivity analysis showed that these reporting patterns were not explained solely by the 11 records without a public registry identifier or by TrialTrove-exclusive discovery.

### 3.7 Registry safety-evidence availability

Only 20/211 records (9.5%) had posted ClinicalTrials.gov results with an adverse-event module: 10 TCR-T, 4 CAR-T, 4 TIL, and 2 NK trials; no CAR-NK or other-ACT record had posted safety results (**Table 3**). Serious-event terminology spanned CRS, neurologic events, cytopenias, infection or febrile neutropenia, hypotension or other cardiovascular events, respiratory toxicity or hypoxia, gastrointestinal toxicity, and renal or urinary toxicity. Because these events could reflect the cell product, lymphodepletion, cytokine support, underlying disease, or concomitant treatment, no pooled incidence or causal attribution was attempted.

**Table 3:** Availability and domains of posted registry safety evidence.

Modality	All trials	Posted results	Adverse-event module	Any serious-event term	Broad serious-event domains observed
TCR-T	42	10	10	8	Cytokine release syndrome; Gastrointestinal toxicity; Hematologic toxicity or cytopenia; Hypotension or vascular/cardiac events; ICANS or other neurologic events; Infection or febrile neutropenia; Renal or urinary toxicity; Respiratory toxicity or hypoxia
CAR-T	79	4	4	4	Cytokine release syndrome; Gastrointestinal toxicity; Hematologic toxicity or cytopenia; Hypotension or vascular/cardiac events; ICANS or other neurologic events; Infection or febrile neutropenia; Renal or urinary toxicity; Respiratory toxicity or hypoxia
TIL	60	4	4	4	Gastrointestinal toxicity; Hematologic toxicity or cytopenia; Hypotension or vascular/cardiac events; ICANS or other neurologic events; Infection or febrile neutropenia; Renal or urinary toxicity; Respiratory toxicity or hypoxia
NK	17	2	2	2	Gastrointestinal toxicity; Hematologic toxicity or cytopenia; Hypotension or vascular/cardiac events; Infection or febrile neutropenia; Renal or urinary toxicity; Respiratory toxicity or hypoxia
CAR-NK	9	0	0	0	
Other ACT	4	0	0	0	

This table summarizes evidence availability only. Event rates were not pooled or compared across modalities, and events were not attributed solely to the cell product.

### 3.8 Expert perspectives on translational priorities

**Figure 7** summarizes the 25 respondents. Eighteen reported cell-therapy research experience, 15 trial design or management experience, 15 gynecologic clinical research experience, and 10 translational-medicine experience; 22 had at least 5 years of relevant experience. Sixteen were based in mainland China and 11 were clinicians. Specific Phase I/II experience and institution-level affiliations were not ascertained.

**Figure 7:**
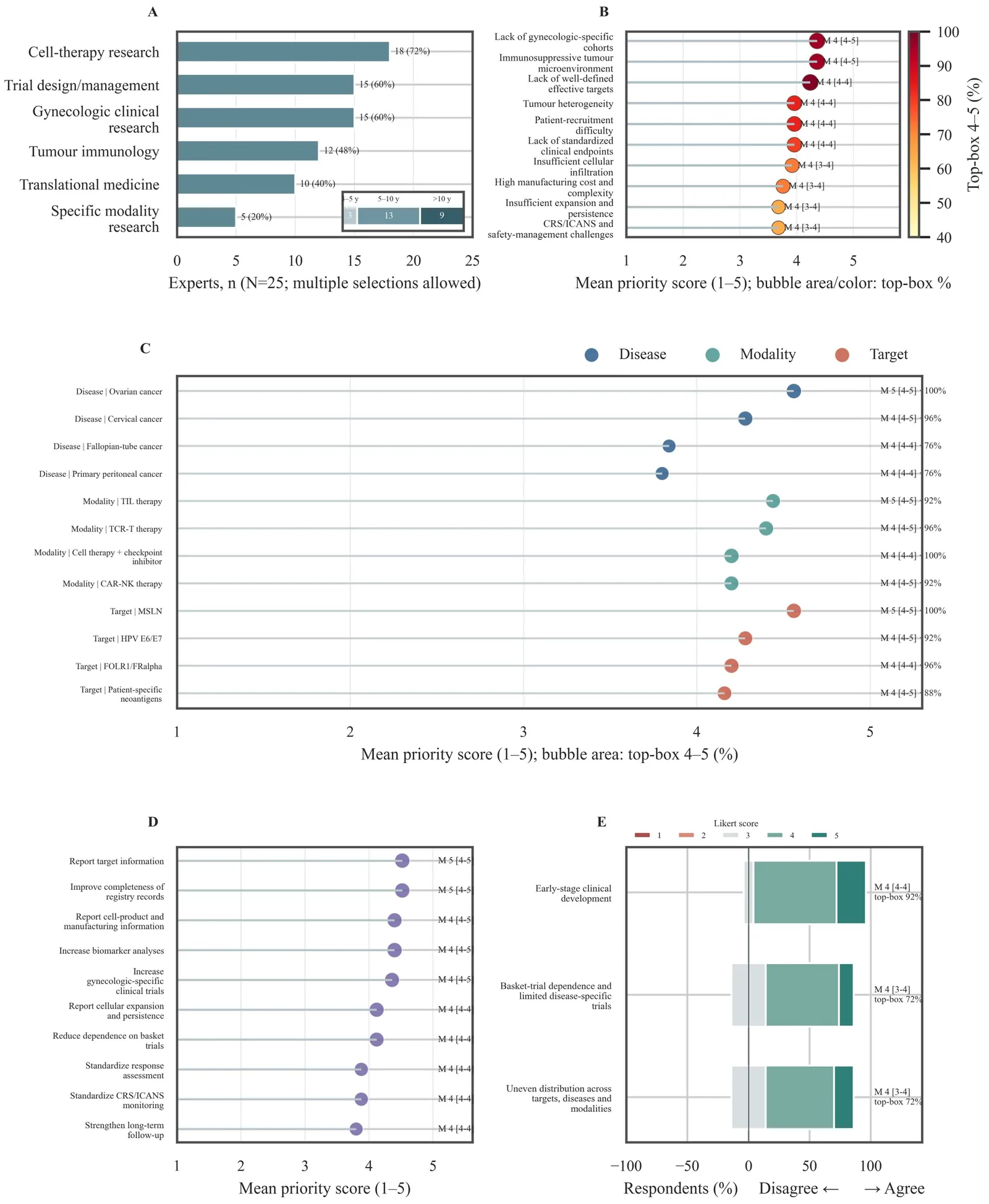
Registry-informed exploratory expert priorities (N = 25). **(A)** Recorded experience domains and years of experience; multiple selections were allowed. **(B)** Development barriers, with point position indicating the mean score and point size/color indicating the top-box proportion (scores 4-5); annotations give median and interquartile range. **(C)** The four highest-rated disease, modality, and target priorities; complete item-level results are in **Supplementary Table S3**. **(D)** Trial-design and reporting priorities. **(E)** Full Likert distributions for the three registry-derived current-state items (Q6-Q8). The survey followed preliminary registry mapping and is interpreted as exploratory priority setting rather than independent validation.

The immunosuppressive tumor microenvironment and lack of gynecologic-specific cohorts were the leading barriers (both median 4, IQR 4-5; 24/25 top-box responses), followed by lack of effective targets (median 4, IQR 4-4; 25/25 top-box). Ovarian cancer was the highest-rated disease priority (median 5, IQR 4-5; 25/25 top-box), and TIL therapy was the highest-rated modality (median 5, IQR 4-5; 23/25 top-box).

MSLN received the highest target-priority rating (median 5, IQR 4-5; 25/25 top-box). Reporting target information and improving registry completeness were the leading design priorities (both median 5, IQR 4-5; 25/25 top-box). Reporting cell-product and manufacturing information (24/25 top-box) and increasing biomarker analyses (25/25 top-box) were also highly rated.

## 4 Discussion

### 4.1 Principal findings

This study analyzed 211 therapeutic adoptive T-cell and NK-cell trial records and a registry-informed exploratory survey of 25 experts. The key new finding is that apparent gynecologic activity was dominated by 166 broader baskets, while only 43 trials were restricted to one gynecologic disease family and 2 to multiple gynecologic families only. The main structural conclusions persisted in the 200-record public-registry-linked and 141-record public-search-captured subsets. Registry safety evidence was sparse, with posted adverse-event results available for only 20 records.

The most important message of this analysis is structural rather than therapeutic. The study was not designed to pool patient-level outcomes, response rates, survival estimates, toxicity events, or comparative treatment effects. Rather, the findings define where trials are being registered, which diseases and modalities are most represented, which target categories have entered clinical development, how trial designs differ, and how completely key design fields are visible in registries.

### 4.2 Early clinical development and the role of basket trials

The clinical landscape was overwhelmingly early phase: Phase I and Phase I/II trials accounted for 199/211 records, whereas only 6 records were Phase II and no mature late-phase pattern was evident. This pattern is consistent with the solid-tumor CAR-T literature, which emphasizes target heterogeneity, restricted trafficking, limited persistence, immunosuppressive tumor microenvironments, toxicity, manufacturing constraints, and predominantly early clinical development (32, 33).

Basket trials were a defining feature of the dataset and can be efficient for early antigen-defined or molecularly defined development when any single tumor type is uncommon. However, eligibility for a broad solid-tumor basket does not demonstrate that a gynecologic cohort enrolled, received treatment, or generated disease-specific outcomes. The three-level architecture therefore distinguishes disease-focused development from eligibility-only opportunity, while the source-sensitivity analysis shows that the broader-basket predominance was not a database artifact.

### 4.3 Disease, modality, and target concentration

The previous ovarian-versus-endometrial contrast was substantially inflated by assigning broad baskets to a single disease label. After eligibility-specific reclassification, 164 records allowed tubo-ovarian/peritoneal disease and 39 allowed endometrial/uterine-corpus disease, but only 36 and 1, respectively, were restricted to those single disease families. The remaining imbalance may reflect the longer history of ovarian antigen-directed ACT programs, accessible ascites and peritoneal disease for tissue acquisition or regional delivery, and persistent unmet need after recurrent platinum-resistant disease. By contrast, biomarker-defined endometrial cancer subsets have rapidly gained effective checkpoint-inhibitor-based options, which may reduce the relative development incentive for cellular therapy even though immune responsiveness does not eliminate unmet need in checkpoint-resistant or biomarker-negative disease. These are plausible explanations rather than causal inferences from registry data.

The modality distribution showed concentration around CAR-T, TIL, and TCR-T approaches. CAR-T represented the largest trial modality group, while TIL and TCR-T formed substantial development streams. NK and CAR-NK approaches were present but accounted for a smaller proportion of trial records, consistent with a still-developing clinical landscape for engineered NK-cell platforms and ongoing questions around manufacturing, persistence, and solid-tumor activity (34, 35).

Named target categories such as MSLN, HPV E6/E7, NY-ESO-1, CD70, HER2, PRAME, ROR1, and patient-specific antigens illustrate different development paths. HPV E6/E7 is most relevant to HPV-associated disease settings, especially cervical cancer, and reflects virus-associated antigen development (36). Neoantigen-specific TIL approaches illustrate a different model in which antitumor recognition may not depend on a single predefined shared antigen (37). CAR-NK development faces separate solid-tumor translation challenges related to persistence, trafficking, and tumor microenvironment resistance (38). Mutation-directed and neoantigen-recognition programs illustrate personalized antigen-selection models (39, 40). Cancer-testis antigen programs, including NY-ESO-1, show how shared antigens can support TCR-engineered cell development (41, 42). Other antigen-directed programs, including armored GPC3 CAR-T, HPV-targeted TIL therapy, CD70 CAR-T, and PRAME-directed T-cell therapy, reinforce the need for explicit target and product annotation in registry records (43–46).

### 4.4 Trial design complexity, endpoints, and safety evidence

Cellular therapy trials were frequently embedded within a treatment backbone, but lymphodepletion and IL-2 support should not be conflated with antitumor combinations. After this distinction, checkpoint inhibitors were the dominant explicit antitumor partner, followed by antiangiogenic and PARP-inhibitor combinations; true antitumor chemotherapy and local-radiotherapy combinations were uncommon (47–49). This pattern is biologically plausible because immune exclusion and tumor-microenvironment pathways can limit cellular activity, while endometrial and cervical systemic-treatment contexts continue to evolve (50–53).

The endpoint and monitoring landscape was heterogeneous. Safety and objective-response endpoints were common, whereas progression-free survival, overall survival, cell persistence, immune biomarkers, quality-of-life outcomes, CRS, and ICANS were less consistently visible. Registry records may omit protocol-level detail, so these findings indicate reporting visibility rather than proof that monitoring was absent.

The sparse registry-result subset limits direct platform comparison, but it identifies the safety domains that future records should report consistently. Engineered CAR-T and TCR-T products require attention to CRS, neurologic toxicity, prolonged cytopenia, infection, target-related tissue injury, and delayed secondary malignancy surveillance; TCR-T additionally depends on HLA restriction and receptor cross-reactivity. TIL programs add substantial toxicity from lymphodepletion and post-infusion IL-2, including cytopenia, infection, and cardiopulmonary or renal stress, whereas NK and CAR-NK programs remain less mature and lack an approved oncology comparator.

These concerns are consistent with marketed-cell-therapy labels and recent reviews of solid-tumor T-cell therapies and second primary cancers after CAR-T therapy but should not be translated into numerical cross-modality comparisons (54, 55). Current FDA labeling for hematologic CAR-T products highlights CRS, neurologic toxicities, and lifelong monitoring for secondary malignancies (56); the approved MAGE-A4 TCR-T product afamitresgene autoleucel carries a boxed warning for CRS and warnings for ICANS, prolonged cytopenia, infection, and secondary malignancy (57); and the approved TIL product lifileucel carries boxed warnings for treatment-related mortality, prolonged severe cytopenia, severe infection, and cardiopulmonary and renal impairment (58). The FDA eliminated REMS requirements for approved BCMA- and CD19-directed autologous CAR-T products in June 2025, while labeling and long-term monitoring requirements remain (59).

### 4.5 Registry reporting completeness and trial learning

Field-specific reporting identified a transparent visibility gap without assuming that heterogeneous fields should receive equal weight. Target/mechanism was interpretable in 80.6% of records, whereas the other five generally applicable fields were visible in 87.2-100.0%. Contemporary protocol guidance emphasizes that public records are most useful when design fields are complete, linkable, and sufficiently detailed for interpretation (60, 61).

We therefore removed the previous composite completeness score from the primary analysis. A public registry cannot substitute for a full protocol, statistical analysis plan, regulatory submission, or manufacturing dossier, and a missing registry field is not equivalent to absent trial conduct. The field-specific estimates should be interpreted as external visibility measures rather than trial-quality scores.

### 4.6 Strengths and limitations

This study has several strengths. It integrates TrialTrove with ClinicalTrials.gov and WHO ICTRP, discloses the contribution of the subscription database, provides a full trial-level list, applies mutually exclusive evidence-specificity rules, and tests the stability of findings in two public-source sensitivity sets. Target classification also separates named targets, antigen-agnostic modality biology, missing reporting, and unresolved information.

Several limitations should be considered. Seventy included records were discovered only through the TrialTrove search, including 11 without public registry identifiers; conversely, 59 TrialTrove-only discoveries were publicly verifiable but were not captured by our public search strings, showing that public availability and public-search capture are different concepts. Registry records may not capture full protocol detail or actual gynecologic enrollment within a broader basket. Only 20 records had posted adverse-event results, precluding reliable cross-modality incidence comparisons, and event attribution to the cell product, lymphodepletion, IL-2, or concomitant therapy was not possible. The trial-record unit of analysis cannot estimate patient-level efficacy, toxicity incidence, response durability, survival, or comparative benefit.

The survey was exploratory, modest in size, purposively sampled, and geographically concentrated. The invited denominator, response rate, institution count, affiliations, and specific Phase I/II experience were not collected. Because the questionnaire followed preliminary registry mapping and presented registry-derived current-state items before the priority ratings, priming and circularity cannot be excluded. The ratings should therefore be interpreted as structured priority setting rather than representative consensus or independent validation.

### 4.7 Implications

Future development would benefit from more gynecologic-specific trials or clearly reported gynecologic cohorts within baskets. Registry records should consistently report disease-specific enrollment, target or antigen-agnostic mechanism, cellular product and manufacturing descriptor, route, lymphodepletion, cytokine support, true antitumor combinations, CRS and ICANS monitoring, persistence, biomarkers, long-term follow-up, and cohort-specific outcomes.

The value of the present landscape is not to rank therapies. It is to make the registered development map visible enough that future trials can be designed with fewer blind spots. Earlier ovarian antigen-directed adoptive cell therapy programs illustrate that gynecologic tumor antigens have long been explored clinically and preclinically (62–64). More complete registry annotation can help investigators understand which development paths are mature enough for cohort-specific evaluation and which remain early, fragmented, or underreported.

The registry-informed expert survey translated observed gaps into hypotheses and development priorities. Its prominent priorities included gynecologic-specific cohorts, target selection, tumor-microenvironment barriers, and more complete reporting of targets, cellular products, biomarkers, and registry fields. These ratings should guide hypothesis generation and trial planning rather than be interpreted as comparative therapeutic evidence or validation of the registry analysis.

## 5 Conclusions

Adoptive T-cell and NK-cell development in gynecologic malignancies is active but remains early phase, broader-basket dominated, and unevenly documented. The primary landscape included 211 therapeutic trial records: 43 single gynecologic disease family-specific trials, 2 gynecologic multicancer-only trials, and 166 broader baskets. The main structural findings remained similar in public-registry-linked and public-search-captured sensitivity subsets.

The field is concentrated around CAR-T, TIL, and TCR-T approaches, but disease eligibility should not be equated with disease-specific evidence. Clearer cohort annotation, field-specific registry reporting, explicit separation of treatment backbone from antitumor combinations, and fuller safety-result disclosure would improve the interpretability and reproducibility of future cellular-therapy development.

## Data Availability

The raw data supporting the conclusions of this article will be made available by the authors, without undue reservation.
