# Supplementary material for: Clinical trial landscape and translational priorities for adoptive T-cell and NK-cell therapies in gynecologic malignancies
Source: Front Immunol. 2026 Sep 28;17:1936815. doi: 10.3389/fimmu.2026.1936815 (PMC13655096; doi:10.3389/fimmu.2026.1936815)
Supplement: Supplementary file 1 [file Table1.docx]

**Supplementary Table S1**

# **S1A. Search strategies and dates**

**Note:** *The analytic cutoff was applied to reported study start dates; source export dates are reported separately.*

| **Source** | **Search/export date** | **Exact strategy** | **Restriction/freeze rule** |
| --- | --- | --- | --- |
| TrialTrove | 2026-06-23 | [(Trial Title contains Gynecologic cancer) OR (Disease is Oncology: Ovarian) OR (Disease is Oncology: Cervical) OR [(Disease is Oncology: Endometrial) OR (Disease is Oncology: Vulvar) OR (Disease is Oncology: Vaginal)]] AND [(Drug Type is Biological > Cellular > Cell technology type > T cell receptor > TCR-T cell) OR (Drug Type is Biological > Cellular > Cell technology type > Chimaeric antigen receptor > CAR-T cell) OR (Drug Type is Biological > Cellular > Cell technology type > ceNK cell) OR (Drug Type is Biological > Cellular > Cell type > Blood cell > Leukocyte > Lymphocyte > Tumour-infiltrating lymphocyte)] | Records with reported study start dates after 2026-06-01 were excluded from the analytic dataset; the later export date is reported separately. |
| ClinicalTrials.gov | 2026-06-25 | Condition: ovarian cancer OR cervical cancer OR endometrial cancer OR uterine cancer OR gynecologic cancer OR gynecological cancer OR gynaecologic cancer OR gynaecological cancer OR fallopian tube cancer OR primary peritoneal cancer OR vulvar cancer OR vaginal cancer; Intervention: CAR-T OR chimeric antigen receptor OR TCR-T OR T cell receptor OR tumor infiltrating lymphocyte OR tumor-infiltrating lymphocyte OR TIL OR natural killer cell OR NK cell OR CAR-NK OR adoptive cell therapy OR adoptive cellular therapy | Records with reported study start dates after 2026-06-01 were excluded from the analytic dataset; the later export date is reported separately. |
| WHO ICTRP | 2026-07-01 | Title: blank; Condition: ovarian cancer OR cervical cancer OR endometrial cancer OR uterine cancer OR gynecologic cancer OR gynecological cancer OR gynaecologic cancer OR gynaecological cancer OR fallopian tube cancer OR primary peritoneal cancer OR vulvar cancer OR vaginal cancer; Intervention: CAR-T OR chimeric antigen receptor OR TCR-T OR T cell receptor OR tumor infiltrating lymphocyte OR tumor-infiltrating lymphocyte OR TIL OR natural killer cell OR NK cell OR CAR-NK OR adoptive cell therapy OR adoptive cellular therapy; other fields blank. | Supplementary public-registry reconciliation; 39 records exported and deduplicated against the other sources. |

# **S1B. Screening flow and analysis sets**

| **Stage** | **Category** | **n** |
| --- | --- | --- |
| Raw records | TrialTrove | 201 |
| Raw records | ClinicalTrials.gov | 330 |
| Raw records | WHO ICTRP | 39 |
| After deduplication | Unique records screened | 442 |
| Excluded | Ineligible ACT intervention | 162 |
| Excluded | No explicit eligible gynecologic cohort | 55 |
| Excluded | Non-interventional or non-therapeutic ACT record | 9 |
| Excluded | Record outside the study-start-date analytic cutoff | 3 |
| Excluded | Preinvasive lesion rather than invasive malignancy | 2 |
| Included | Recommended therapeutic landscape | 211 |
| Sensitivity | Public-registry-linked subset | 200 |
| Sensitivity | Public-search-captured subset | 141 |

# **S1C. Operational definitions**

| **Classification item** | **Operational definition** |
| --- | --- |
| Therapeutic ACT record | A record in which an eligible adoptive T-cell or NK-cell product is administered for invasive gynecologic malignancy or for a broader malignant-tumour basket explicitly allowing an eligible gynecologic disease family. |
| Single gynecologic disease family specific | Eligibility is restricted to one gynecologic disease family; tubo-ovarian, fallopian-tube, and primary-peritoneal cancers are treated as one family. |
| Multiple gynecologic disease families only | Eligibility includes two or more gynecologic disease families but no non-gynecologic malignancy. |
| Broader basket including non-gynecologic cancers | Eligibility explicitly includes at least one non-gynecologic malignant tumour in addition to a gynecologic disease family. |
| Public-registry-linked subset | Records with a nonblank public registry identifier, regardless of discovery source. |
| Public-search-captured subset | Records actually captured by this study's ClinicalTrials.gov or WHO ICTRP searches, including records also found in TrialTrove. |
| Named target | A specific shared, viral, mutation-directed, or patient-specific target is explicitly named. |
| No predefined antigen | The cell modality is biologically antigen-agnostic or does not require a fixed shared antigen (primarily TIL and non-engineered NK approaches). |
| Not reported | The registry-derived record does not state a target; this is treated as a reporting-visibility state. |
| Unknown | The source wording does not support a reliable named-target or antigen-agnostic classification. |
| Target/mechanism interpretable | Named target or no-predefined-antigen biology; excludes not-reported and unknown states. |
| Cell product or descriptor reported | A nonblank product name or cellular-product descriptor is present; a proprietary name is not required. |
| Positive sample size interpretable | Enrollment/sample size is present and greater than zero. |
| DLT/MTD mention denominator | Restricted to Phase I and Phase I/II records. |
| Registry adverse-event synthesis | Restricted to ClinicalTrials.gov records with posted results and an adverse-events module; reports evidence availability and event domains, not pooled incidence or product attribution. |
