# Supplementary material for: Clinical trial landscape and translational priorities for adoptive T-cell and NK-cell therapies in gynecologic malignancies
Source: Front Immunol. 2026 Sep 28;17:1936815. doi: 10.3389/fimmu.2026.1936815 (PMC13655096; doi:10.3389/fimmu.2026.1936815)
Supplement: Supplementary file 2 [file Table2.docx]

**Supplementary Table S2**

# **S2A. Included trial records — Part 1 of 5: registry identifiers and study context**

**Note:** *One row represents one deduplicated trial record. The 211 records are repeated across five field groups and can be joined using Trial ID. Broader-basket eligibility does not imply that gynecologic participants were enrolled or that cohort-specific outcomes are available.*

| **TrialID** | **PublicregistryID** | **Registrytype** | **Discoverysource** | **Trialtitle** | **Sponsor** | **Countries** | **Evidence-specificityarchitecture** | **Gynecologicdiseaseeligibility** |
| --- | --- | --- | --- | --- | --- | --- | --- | --- |
| NCT07644403 | NCT07644403 | ClinicalTrials.gov | TrialTrove | Phase I/IIa Clinical Trial Evaluating the Safety and Efficacy of IMC001 in Patients With Advanced Epithelial Solid Tumors. | Suzhou Immunofoco Biotechnology Co. | China | Broader basket including non-gynecologic cancers | tubo-ovarian/peritoneal |
| NCT07534813 | NCT07534813 | ClinicalTrials.gov | TrialTrove | A Single-Arm Clinical Study of Autologous Tumor-Infiltrating Lymphocyte Injection (GT307) in the Treatment of Metastatic and Recurrent Advanced Solid Tumors | Zhuhai Grit Biotechnology/Shanghai Grit Biotechnology Fudan University | China | Broader basket including non-gynecologic cancers | cervical |
| 695613 | EUCT 2024-517594-24-01 | EU Clinical Trials Register | TrialTrove | A Phase I/IIa clinical study of novoleucel in patients with persistent or recurrent cervical cancer who have progressed after platinum-based chemotherapy and checkpoint inhibitors. | Karolinska Institutet Invion Limited {CBio} | Sweden | Single gynecologic disease family specific | cervical |
| NCT07420010 | NCT07420010 | ClinicalTrials.gov | Both | An Open-label, Single-arm Clinical Study to Evaluate the Safety, Tolerability, Pharmacokinetic Profile, and Preliminary Efficacy of KT032 Cell Injection in Patients With Mesothelin-positive Advanced Solid Tumors. | Zhejiang University | China | Broader basket including non-gynecologic cancers | tubo-ovarian/peritoneal |
| NCT07266298 | NCT07266298 | ClinicalTrials.gov | TrialTrove | A Phase I, Multicenter, Dose-escalation, Single-arm Study of PRAME Antigen-targeted TCR-T Cells(NW-101C) in the Treatment of Subjects With Advanced Solid Malignant Tumors. | Neowise Biotechnology | China | Broader basket including non-gynecologic cancers | tubo-ovarian/peritoneal |
| NCT07231081 | NCT07231081 | ClinicalTrials.gov | TrialTrove | An Open-label, Single-arm, Phase I Clinical Trial to Evaluate the Safety and Efficacy of TX103 CAR-T Cells in Participants With Advanced Solid Tumors | Fuzhou Tcelltech Biotechnology Co. | China | Broader basket including non-gynecologic cancers | tubo-ovarian/peritoneal |
| NCT07216105 | NCT07216105 | ClinicalTrials.gov | Both | A Phase I, Open-Label Study of FT836, an Off-the-Shelf CAR T-Cell Therapy, With or Without Chemotherapy and/or Monoclonal Antibodies, in Participants With Advanced Solid Tumors | Fate Therapeutics | United States | Broader basket including non-gynecologic cancers | tubo-ovarian/peritoneal; endometrial/uterine corpus |
| 668508 | ChiCTR2500109506 | ChiCTR | TrialTrove | A study to evaluate the safety and primary efficacy of the combination therapy of recombinant human IL-21 oncolytic vaccinia virus injection (hV01) and ready-to-use CAR-raNK cells (IBR854) in patients with advanced malignant solid tumors | (Other Hospital/Academic/Medical Center) Hangzhou Converd Co. | China | Broader basket including non-gynecologic cancers | tubo-ovarian/peritoneal; cervical; endometrial/uterine corpus |
| NCT07181720 | NCT07181720 | ClinicalTrials.gov | Both | Clinical Study of CD70-Targeted Chimeric Antigen Receptor T Lymphocytes (CAR-T) in Advanced CD70-Positive Malignant Solid Tumors | Chongqing Precision Biotech Co. | China | Broader basket including non-gynecologic cancers | tubo-ovarian/peritoneal; cervical |
| NCT07106814 | NCT07106814 | ClinicalTrials.gov | Both | Safety and Efficacy of Metabolically Armed Tumor-lnfiltrating Lymphocytes (Meta10-TIL) for the Treatment of Advanced Solid Tumors | (Other Hospital/Academic/Medical Center) Leman Biotech | China | Broader basket including non-gynecologic cancers | tubo-ovarian/peritoneal; cervical |
| NCT07101549 | NCT07101549 | ClinicalTrials.gov | TrialTrove | A Single-center, Single-arm, Dose-escalation Exploratory Clinical Trial on the Safety, Efficacy, and Pharmacokinetics of R-Star001 Cell Injection in Patients With Nectin-4-positive Advanced Solid Tumors | (Other Hospital/Academic/Medical Center) | China | Broader basket including non-gynecologic cancers | tubo-ovarian/peritoneal |
| NCT07067255 | NCT07067255 | ClinicalTrials.gov | Both | Phase I/II Study of Sequential CD146 and GPC3 CAR-T Cell Therapy in Advanced Ovarian Cancer | Essen Biotech | China | Broader basket including non-gynecologic cancers | tubo-ovarian/peritoneal |
| NCT07030907 | NCT07030907 | ClinicalTrials.gov | Both | A Phase 1a/b Study to Evaluate the Safety and Efficacy of OPB-101, an Autologous Mesothelin (MSLN) CAR T Cell Therapy With Antigen-dependent Expression of OUTSMART™ Designed IL-2 Cytokine in Platinum-resistant Ovarian Cancer | Outpace Bio | United States | Broader basket including non-gynecologic cancers | tubo-ovarian/peritoneal |
| NCT07010523 | NCT07010523 | ClinicalTrials.gov | TrialTrove | Exploratory Study to Evaluate the Safety, Tolerability and Preliminary Efficacy of Anti-Mesothelin CAR-T Cell Injection in Patients With Mesothelin-positive Advanced Malignant Solid Tumors | (Other Hospital/Academic/Medical Center) UTC Therapeutics | China | Broader basket including non-gynecologic cancers | endometrial/uterine corpus |
| NCT06951425 | NCT06951425 | ClinicalTrials.gov | TrialTrove | A Phase l, Open-Label, Dose-escalation Study to Evaluate the Safety, Tolerability and Antitumor Activity of TH027 CAR-T Cells (TH-CART-027) in Subjects With Relapsed or Refractory Solid Tumors | (Other Hospital/Academic/Medical Center) Thinking Biomed (Beijing) Co. | China | Broader basket including non-gynecologic cancers | tubo-ovarian/peritoneal |
| NCT06912152 | NCT06912152 | ClinicalTrials.gov | Both | Phase I Dose-Escalation Study of MT027: Evaluating Tolerability, Safety, Pharmacokinetics, and Preliminary Efficacy in Patients With Advanced Peritoneal Malignancies or Abdominal Metastatic Solid Tumors | (Other Hospital/Academic/Medical Center) | China | Broader basket including non-gynecologic cancers | tubo-ovarian/peritoneal |
| NCT06904131 | NCT06904131 | ClinicalTrials.gov | TrialTrove | A Phase I Clinical Trial of MUC1-targeted CAR-T Cells with PD1 Nanobody MSLN Dual-target for Advanced Gynecological Solid Tumors | (Other Hospital/Academic/Medical Center) | China | Broader basket including non-gynecologic cancers | tubo-ovarian/peritoneal; cervical; endometrial/uterine corpus; vulvar; vaginal |
| NCT06846424 | NCT06846424 | ClinicalTrials.gov | Both | A Clinical Study to Evaluate the Safety and Tolerability of SCT-001 CAR-T Cells in Patients with Recurrent and Refractory Epithelial Ovarian Cancer, Fallopian Tube Cancer, and Peritoneal Cancer | Gynecologic Oncology Group (GOG) | China | Single gynecologic disease family specific | tubo-ovarian/peritoneal |
| NCT06834542 | NCT06834542 | ClinicalTrials.gov | TrialTrove | Single Arm Clinical Study of Autologous Tumor Infiltrating Lymphocyte Injection (GT201) in the Treatment of Advanced Gynecological Tumors | Zhuhai Grit Biotechnology | China | Multiple gynecologic disease families only | tubo-ovarian/peritoneal; cervical; endometrial/uterine corpus; vulvar; vaginal |
| NCT06748872 | NCT06748872 | ClinicalTrials.gov | Both | EPITOME-1015-I: a Phase I Study to Investigate the Safety, Tolerability and Preliminary Efficacy of a Third Generation TCR-T Therapy, MDG1015, in Epithelial Ovarian Carcinoma, Gastroesophageal (Junction) Adenocarcinoma, Myxoid (Round Cell) Liposarcoma And/or Synovial Sarcoma Subjects with Advanced Disease Expressing NY-ESO-1 And/or LAGE-1a | Medigene | Netherlands; United States | Broader basket including non-gynecologic cancers | tubo-ovarian/peritoneal |
| NCT06710288 | NCT06710288 | ClinicalTrials.gov | Both | A Phase II, Open-label, Single-arm Study of Autologous Memory Cytokine Enriched Natural Killer (M-CENK) Adoptive Cell Therapy and N-803 (IL-15 Superagonist) in Combination with Gemcitabine in Participants with Recurrent Platinum-Resistant High-Grade Ovarian Cancer;A Phase 2, Open-label, Single-arm Study of Autologous M-CENK Adoptive Cell Therapy and N-803 (IL-15 Superagonist) in Combination with Gemcitabine in Participants with Recurrent Platinum-Resistant High-Grade Ovarian Cancer | NantWorks/ImmunityBio | United States | Broader basket including non-gynecologic cancers | tubo-ovarian/peritoneal |
| 545596 | EUCT 2023-504632-17-00 | EU Clinical Trials Register | TrialTrove | A phase II, Multicenter Study of TILs Treatment in Advanced Tumors with Alterations in the SWI/SNF Complex: the TILTS Study | (Other Hospital/Academic/Medical Center) | Spain | Broader basket including non-gynecologic cancers | tubo-ovarian/peritoneal |
| NCT06697665 | NCT06697665 | ClinicalTrials.gov | TrialTrove | An Exploratory Clinical Study to Evaluate the Safety, Tolerability, Immune Response and Preliminary Efficacy of LM103 Injection in Combination With PD-1 in Patients With Advanced Solid Tumours | Suzhou BlueHorse Therapeutics Co. | China | Broader basket including non-gynecologic cancers | tubo-ovarian/peritoneal; cervical |
| NCT06658951 | NCT06658951 | ClinicalTrials.gov | TrialTrove | Exploratory Study to Evaluate the Safety and Preliminary Efficacy of Anti-HER2 CAR-T Cell Injection in Patients With HER2-positive Advanced Malignant Solid Tumors | UTC Therapeutics (Other Hospital/Academic/Medical Center) | China | Broader basket including non-gynecologic cancers | tubo-ovarian/peritoneal; cervical; endometrial/uterine corpus |
| NCT06646627 | NCT06646627 | ClinicalTrials.gov | Both | Phase I Clinical Trial of Autologous B7-H3 Chimeric Receptor (CAR) T Cells in Adults With Recurrent, Platinum Resistant Ovarian Tumors | Stanford University Medical Center | United States | Broader basket including non-gynecologic cancers | tubo-ovarian/peritoneal |
| NCT06630611 | NCT06630611 | ClinicalTrials.gov | Both | Phase II Randomized Study Evaluating a Pragmatic Approach to Adoptive Cell Therapy (ACT) Using an IL2 Analog (ANV419) vs High Dose IL2 After Tumor Infiltrating Lymphocytes (TIL) Therapy in Patients With Melanoma, NSCLC and Cervical Cancer | (Other Hospital/Academic/Medical Center) | Denmark; Israel; Netherlands; Spain | Broader basket including non-gynecologic cancers | cervical |
| NCT06598371 | NCT06598371 | ClinicalTrials.gov | TrialTrove | A Phase I/II Study of KSQ-004EX, Autologous Tumor Infiltrating Lymphocytes Engineered to Inactivate Genes Encoding SOCS1 and Regnase-1, in Patients With Select Advanced Solid Tumors | MD Anderson Cancer Center, University of Texas KSQ Therapeutics | United States | Broader basket including non-gynecologic cancers | cervical |
| NCT06544720 | NCT06544720 | ClinicalTrials.gov | Both | A Phase I Clinical Study of Autologous TCR-T Cells (SCG142) Therapy for Advanced HPV Associated Carcinomas | (Other Hospital/Academic/Medical Center) | China | Broader basket including non-gynecologic cancers | cervical; vulvar; vaginal |
| NCT06509490 | NCT06509490 | ClinicalTrials.gov | TrialTrove | A Single-center, Open, Single-arm Clinical Study of the Safety and Efficacy of KD-025 CAR-T Therapy in Advanced NKG2DL+ Solid Tumors | Chinese Academy of Medical Sciences | China | Broader basket including non-gynecologic cancers | tubo-ovarian/peritoneal |
| NCT06505551 | NCT06505551 | ClinicalTrials.gov | Both | A Phase I/II Open Label, Single Arm, Multicenter Study to Evaluate the Safety and Preliminary Eficacy of Autologous SCG142 T Cell Receptor (TCR) T Cells in Patients With Advanced or Metastatic HPV16- or HPV52-positive Carcinomas | SCG Cell Therapy | China; Singapore; United States | Broader basket including non-gynecologic cancers | cervical; vulvar; vaginal |
| NCT06481592 | NCT06481592 | ClinicalTrials.gov | Both | A Phase II, Multicenter, Open-label Study of Lifileucel (tumor-infiltrating Lymphocytes [TIL]) in Participants with Previously Treated Advanced Endometrial Cancer. | Iovance Biotherapeutics {Lion Biotechnologies} | United States | Single gynecologic disease family specific | endometrial/uterine corpus |
| NCT06453057 | NCT06453057 | ClinicalTrials.gov | TrialTrove | A Single-arm Clinical Study of Autologous Tumor-Infiltrating Lymphocyte (GT307) for Treatment of Patients With Solid Tumours | Zhuhai Grit Biotechnology | China | Broader basket including non-gynecologic cancers | tubo-ovarian/peritoneal |
| 518933 | ChiCTR2000032124 | ChiCTR | TrialTrove | Safety and Efficacy of Chimeric Antigen Receptor Engineered T cells for Treating Malignant Cancers | (Other Hospital/Academic/Medical Center) Hunan Siweikang Therapeutics Co. | China | Broader basket including non-gynecologic cancers | tubo-ovarian/peritoneal |
| NCT06375187 | NCT06375187 | ClinicalTrials.gov | Both | A Phase I Study to Evaluate the Safety and Efficacy of Engineering Tumor Infiltrating Lymphocytes Injection (GC203 TIL)in Patients With Advanced Malignant Solid Tumors | Shanghai Juncell Therapeutics Co. | China | Broader basket including non-gynecologic cancers | tubo-ovarian/peritoneal; cervical; endometrial/uterine corpus |
| NCT06358053 | NCT06358053 | ClinicalTrials.gov | Both | A Phase I Study to Evaluate the Safety, Tolerance and Efficacy of CRTE7A2-01 TCR-T Cells in HLA-A*02:01+ Subjects With HPV16 Positive Advanced Cervical, Anal, or Head and Neck Cancers and Other Solid Tumors | Corregene Biotechnology Co. | China | Broader basket including non-gynecologic cancers | cervical |
| 510939 |  | Other registry | TrialTrove | A Clinical Study of CD19 CAR-T therapy in Patients with Ovarian Cancer/Solid Tumors | H. Lee Moffitt Cancer Center and Research Institute Anixa Biosciences |  | Broader basket including non-gynecologic cancers | tubo-ovarian/peritoneal |
| NCT06342336 | NCT06342336 | ClinicalTrials.gov | TrialTrove | Single Arm, Phase I Clinical Study of HS-IT101 Injection in the Treatment of Advanced Solid Tumors | Qingdao Sino-Cell Biomedicine Co. (Other Hospital/Academic/Medical Center) Chinese Academy of Medical Sciences | China | Broader basket including non-gynecologic cancers | cervical |
| NCT06327997 | NCT06327997 | ClinicalTrials.gov | TrialTrove | Exploratory Study on the Treatment of Advanced Solid Tumor With Non-viral Vector Multi-targeted Autosecretory Multifunctional Antibody CAR-T Cell Injection | Shanghai Cell Therapy Group Co. | China | Broader basket including non-gynecologic cancers | tubo-ovarian/peritoneal; cervical; endometrial/uterine corpus |
| NCT06305299 | NCT06305299 | ClinicalTrials.gov | Both | Administration of T Cells Expressing B7-H3 Specific Chimeric Antigen Receptors (CAR) and Containing the Inducible Caspase 9 Safety Switch in Subjects With Recurrent Platinum Resistant Epithelial Ovarian Cancer | UNC Lineberger Comprehensive Cancer Center | United States | Single gynecologic disease family specific | tubo-ovarian/peritoneal |
| NCT06256055 | NCT06256055 | ClinicalTrials.gov | Both | A First-In-Human, Single Arm, Open-label, Phase I Dose-Escalation Study of UCMYM802 Injection in Mesothelin-positive Advanced Malignant Solid Tumors | UTC Therapeutics Peking Union Medical College Hospital, CAMS | China | Broader basket including non-gynecologic cancers | tubo-ovarian/peritoneal; endometrial/uterine corpus |
| NCT06241781 | NCT06241781 | ClinicalTrials.gov | Both | A Phase II, Multicenter Randomized Controlled Open-label Study of Autologous Tumor Infiltrating Lymphocytes (GT101 Injection) in Patients With Recurrent or Metastatic Cervical Cancer | Zhuhai Grit Biotechnology | China | Broader basket including non-gynecologic cancers | cervical |
| NCT06218914 | NCT06218914 | ClinicalTrials.gov | Both | Open-label, Phase I, Multi-Center Master Protocol to Evaluate the Safety and Preliminary Anti-Tumor Activity of TCR-engineered T Cells Recognizing KRAS Mutations in Adult Subjects With Unresectable, Advanced, and/or Metastatic Solid Tumors | AstraZeneca/Neogene Therapeutics | United States | Broader basket including non-gynecologic cancers | endometrial/uterine corpus |
| NCT06191900 | NCT06191900 | ClinicalTrials.gov | Both | Single Arm Clinical Study of Autologous Tumor Infiltrating Lymphocyte Injection (GT201) in the Treatment of Advanced Gynecological Tumors (Advanced Cervical Cancer) | Zhuhai Grit Biotechnology/Shanghai Grit Biotechnology | China | Multiple gynecologic disease families only | tubo-ovarian/peritoneal; cervical; endometrial/uterine corpus; vulvar; vaginal |
| 496428 | CTR20233999 | China CDE CTR | TrialTrove | Phase I Clinical Study of Safety, Tolerability, Immune Response and Preliminary Efficacy of LM103 Injection in Patients with Advanced Solid Tumors | Suzhou BlueHorse Therapeutics Co. | China | Broader basket including non-gynecologic cancers | tubo-ovarian/peritoneal; cervical |
| NCT06145802 | NCT06145802 | ClinicalTrials.gov | TrialTrove | Single Arm Clinical Study of Autologous Tumor Infiltrating Lymphocyte Injection (GT316) in the Treatment of Advanced Solid Tumors (gynecological Tumors) | Zhuhai Grit Biotechnology | China | Broader basket including non-gynecologic cancers | tubo-ovarian/peritoneal; cervical |
| 489390 |  | Other registry | TrialTrove | A Clinical Study of anticancer cell therapy, TCR-T in Patients with Solid Tumors | AdaptImmune |  | Broader basket including non-gynecologic cancers | tubo-ovarian/peritoneal; endometrial/uterine corpus |
| NCT06051695 | NCT06051695 | ClinicalTrials.gov | Both | A Seamless Phase I/II Study to Evaluate the Safety and Efficacy of Mesothelin-Targeting Autologous Logic-gated Tmod™ CAR T Products, in Heterozygous HLA-A*02 Adults With Recurrent Unresectable, Locally Advanced, or Metastatic Solid Tumors That Express MSLN and Have Lost HLA-A*02 Expression | A2 Biotherapeutics Tempus AI | United States | Broader basket including non-gynecologic cancers | tubo-ovarian/peritoneal |
| NCT06010875 | NCT06010875 | ClinicalTrials.gov | Both | A Phase I Clinical Study to Assess the Safety and Efficacy of CD70-targeted CAR-T in the Treatment of CD70-positive Advanced/Metastatic Solid Tumors | Chongqing Precision Biotech Co. (Other Hospital/Academic/Medical Center) | China | Broader basket including non-gynecologic cancers | tubo-ovarian/peritoneal; cervical |
| NCT05973487 | NCT05973487 | ClinicalTrials.gov | Both | A Phase I Basket Study Evaluating the Safety and Feasibility of T-Plex, Autologous Customized T Cell Receptor-Engineered T Cells Targeting Multiple Peptide/HLA Antigens in Participants With Antigen-positive Locally Advanced (Unresectable) or Metastatic Solid Tumors | TScan Therapeutics | United States | Broader basket including non-gynecologic cancers | tubo-ovarian/peritoneal; cervical; vulvar; vaginal |
| NCT05971576 | NCT05971576 | ClinicalTrials.gov | TrialTrove | An Exploratory Clinical Study Evaluating the Safety, Tolerance, Immune Response, and Initial Efficacy of Autologous Tumor Infiltrating Lymphocytes (TILs) LM103 Injection in Patients With Advanced Solid Tumors | Suzhou BlueHorse Therapeutics Co. |  | Broader basket including non-gynecologic cancers | tubo-ovarian/peritoneal; cervical |
| NCT05952947 | NCT05952947 | ClinicalTrials.gov | Both | A Multicenter, Single Arm, Open Label, Phase I Clinical Study to Evaluate the Safety, Tolerability and Efficacy of HRYZ-T101 Injection for HPV18 Positive Solid Tumor | HRYZ Biotech Co. | China | Broader basket including non-gynecologic cancers | cervical; vulvar; vaginal |
| NCT05941936 | NCT05941936 | ClinicalTrials.gov | TrialTrove | An Exploratory Clinical Study Evaluating the Safety, Tolerance, Immune Response, and Initial Efficacy of Autologous Tumor Infiltrating Lymphocytes (TILs) LM103 Injection in Patients With Advanced Solid Tumors | Suzhou BlueHorse Therapeutics Co. | China | Broader basket including non-gynecologic cancers | tubo-ovarian/peritoneal; cervical |
| NCT05902520 | NCT05902520 | ClinicalTrials.gov | Both | Phase I Randomized Open Label Trial Investigating Adoptive Transfer of CD39+CD103+CD8+ Tumor-isolated T Cells (AGX148) Alone or Combined With siRNA Modulation of PD-1 (AGX148/PH-762) in Patients With Advanced Solid Tumors | AgonOx (Other Cooperative Group) Phio Pharmaceuticals {RXi Pharmaceuticals} | United States | Broader basket including non-gynecologic cancers | tubo-ovarian/peritoneal; cervical; endometrial/uterine corpus; vulvar |
| NCT05877599 | NCT05877599 | ClinicalTrials.gov | Both | An Open-label, Phase 1, Multicenter Study to Evaluate the Safety and Preliminary Anti-tumor Activity of NT-175 in Human Leukocyte Antigen-A*02:01-Positive Adult Subjects With Unresectable, Advanced and/or Metastatic Solid Tumors That Are Positive for the TP53 R175H Mutation | AstraZeneca/Neogene Therapeutics | United States | Broader basket including non-gynecologic cancers | tubo-ovarian/peritoneal |
| NCT05848999 | NCT05848999 | ClinicalTrials.gov | TrialTrove | Exploratory Study to Evaluate the Safety, Tolerability and Preliminary Efficacy of UCLM802 (Anti-Mesothelin CAR-T) Cell Injection in Patients With Mesothelin-positive Advanced Malignant Solid Tumors | (Other Hospital/Academic/Medical Center) UTC Therapeutics | China | Broader basket including non-gynecologic cancers | tubo-ovarian/peritoneal; endometrial/uterine corpus |
| NCT05787535 | NCT05787535 | ClinicalTrials.gov | Both | A Phase I Study to Evaluate the Safety, Tolerance and Efficacy of HRYZ-T101 TCR-T Cell for HPV18 Positive Advanced Solid Tumor | SYZ Cell Therapy Co. | China | Broader basket including non-gynecologic cancers | cervical; vulvar; vaginal |
| 463409 |  | Other registry | TrialTrove | A First-in-Human Clinical Study of SNAP-CAR-T cell therapy to Evaluate Numerous cancers, including HER2-expressing Ovarian Cancer | Coeptis Therapeutics Holdings University of Pittsburgh |  | Single gynecologic disease family specific | tubo-ovarian/peritoneal |
| NCT05783089 | NCT05783089 | ClinicalTrials.gov | TrialTrove | Anti-Mesothelin CAR-T Therapy in Subjects With Mesothelin-positive Advanced Malignant Solid Tumors. | Chinese Academy of Medical Sciences UTC Therapeutics |  | Broader basket including non-gynecologic cancers | tubo-ovarian/peritoneal; endometrial/uterine corpus |
| NCT05775666 | NCT05775666 | ClinicalTrials.gov | TrialTrove | Exploratory Study to Evaluate the Safety, Tolerability and Preliminary Efficacy of UCLM802 (Anti-Mesothelin CAR-T) Cell Injection in Patients With Mesothelin-positive Advanced Malignant Solid Tumors | Peking Union Medical College Hospital, CAMS UTC Therapeutics | China | Broader basket including non-gynecologic cancers | tubo-ovarian/peritoneal; endometrial/uterine corpus |
| NCT05729399 | NCT05729399 | ClinicalTrials.gov | Both | A Multi-center,Single-arm Phase I/II Clinical Study of Autologous Tumor-infiltrating Lymphocyte Injection (GT201) for Advanced Solid Tumors | Zhuhai Grit Biotechnology | China | Broader basket including non-gynecologic cancers | tubo-ovarian/peritoneal; cervical; endometrial/uterine corpus; vulvar; vaginal |
| 456726 |  | Other registry | TrialTrove | A Clinical Trial of CAR T targeting MUC16 in Patients with Relapsed/Refractory Ovarian Cancer. | 2Seventy Bio Regeneron |  | Single gynecologic disease family specific | tubo-ovarian/peritoneal |
| NCT05672459 | NCT05672459 | ClinicalTrials.gov | Both | A Phase I/IIa, Safety And Efficacy Study Of HLA-G- Targeted CAR-T Cells IVS-3001 In Subjects With Previously Treated Advanced HLA-G-Positive Solid Tumors | MD Anderson Cancer Center, University of Texas Invectys National Resilience | United States | Broader basket including non-gynecologic cancers | tubo-ovarian/peritoneal |
| NCT05881525 | NCT05881525 | ClinicalTrials.gov | TrialTrove | A Phase I/II Clinical Study of TC-N201 Injection for the Treatment of Advanced Solid Tumors With HLA-A2 Expression and Positive NY-ESO-1. | TCRCure Biopharma Technology Co. Chinese Academy of Medical Sciences | China | Broader basket including non-gynecologic cancers | cervical |
| 443882 | ChiCTR2200064112 | ChiCTR | TrialTrove | Clinical Study on the Safety and Efficacy of CAR-T cells in the Treatment of Advanced Tumors | (Other Hospital/Academic/Medical Center) Innovative Cellular Therapeutics Co. | China | Broader basket including non-gynecologic cancers | tubo-ovarian/peritoneal; cervical; endometrial/uterine corpus |
| NCT05568680 | NCT05568680 | ClinicalTrials.gov | Both | A Phase I Study of SynKIR-110, Autologous T Cells Transduced with Mesothelin KIR-CAR, in Subjects with Mesothelin-Expressing Advanced Ovarian Cancer, Cholangiocarcinoma, or Mesothelioma | Verismo Therapeutics | United States | Broader basket including non-gynecologic cancers | tubo-ovarian/peritoneal |
| NCT05539768 | NCT05539768 | ClinicalTrials.gov | TrialTrove | Study on the Safety and Efficacy of Autogenous Tumor Infiltrates Lymphocytes (HS-IT101) for the Treatment of Advanced Solid Tumor | (Other Hospital/Academic/Medical Center) Qingdao Sino-Cell Biomedicine Co. | China | Broader basket including non-gynecologic cancers | cervical |
| 439506 | CTR20221207 | China CDE CTR | TrialTrove | An Open-label, Single-arm, Phase I Clinical Trial of Autologous Native Tumor-infiltrating Lymphocyte Injection (GC101 TIL) in Patients with Advanced Malignant Solid Tumors | Shanghai Junshi Biosciences Co. | China | Broader basket including non-gynecologic cancers | cervical |
| NCT05483491 | NCT05483491 | ClinicalTrials.gov | Both | T Cell Receptor Gene Therapy Targeting KK-LC-1 for Gastric, Breast, Cervical, Lung, and Other KK-LC-1 Positive Cancers | (Other Hospital/Academic/Medical Center) National Institutes of Health/National Cancer Institute T-Cure Bioscience | United States | Broader basket including non-gynecologic cancers | cervical |
| NCT05475847 | NCT05475847 | ClinicalTrials.gov | Both | A Phase I Clinical Study to Evaluate Safety and Efficacy of C-TIL052A Cell Therapy (Autologous Tumor Infiltrating Lymphocytes Injection Combined With IL-2) in Subjects With Persistent, Recurrent and/or Metastatic Cervical Cancer | Fudan University - Shanghai, China AbelZeta Pharma {Cellular Biomedicine Group} | China | Single gynecologic disease family specific | cervical |
| NCT05472857 | NCT05472857 | ClinicalTrials.gov | Both | Clinical Study of CLDN18.2-targeting Chimeric Antigen Receptor-modified Autologous T Cells in Advanced Solid Tumors With Positive CLDN18.2 Expression | Suzhou Immunofoco Biotechnology Co. (Other Hospital/Academic/Medical Center) | China | Broader basket including non-gynecologic cancers | tubo-ovarian/peritoneal |
| NCT05468307 | NCT05468307 | ClinicalTrials.gov | Both | A Clinical Study of Engineered Tumor Infiltrating Lymphocytes Injection (GC203 TIL) in Patients With Advanced Gynecologic Tumors | Shanghai Juncell Therapeutics Co. (Other Hospital/Academic/Medical Center) | China | Broader basket including non-gynecologic cancers | tubo-ovarian/peritoneal; cervical; endometrial/uterine corpus; vulvar; vaginal |
| NCT05468190 | NCT05468190 | ClinicalTrials.gov | Both | A Phase I Clinical Study to Assess the Safety and Tolerability of CD70-targeting CAR-T in the Treatment of CD70-positive Advanced/Metastatic Solid Tumors | Chongqing Precision Biotech Co. | China | Broader basket including non-gynecologic cancers | tubo-ovarian/peritoneal; cervical |
| NCT05430373 | NCT05430373 | ClinicalTrials.gov | TrialTrove | A Single-arm Phase I Clinical Study of GT101 Injection for the Treatment of Metastatic or Recurrent Solid Tumors | Zhuhai Grit Biotechnology Boehringer Ingelheim Clinigen Group {Clinigen Healthcare} | China | Broader basket including non-gynecologic cancers | cervical |
| NCT05417750 | NCT05417750 | ClinicalTrials.gov | TrialTrove | An Open-label, Single-arm, Phase I Study to Evaluate the Safety and Efficacy of Autologous Tumor Infiltrating Lymphocytes Injection in Patients With Advanced Malignant Solid Tumors | Shanghai Juncell Therapeutics Co. | China | Broader basket including non-gynecologic cancers | tubo-ovarian/peritoneal; cervical; endometrial/uterine corpus |
| NCT05397093 | NCT05397093 | ClinicalTrials.gov | Both | A Phase Ia/Ib, Open-Label, Multicenter Study Evaluating the Safety and Feasibility of ITIL-306 in Subjects with Advanced Solid Tumors | Instil Bio | United States | Broader basket including non-gynecologic cancers | tubo-ovarian/peritoneal |
| NCT05373147 | NCT05373147 | ClinicalTrials.gov | Both | An Exploratory Study of αPD1-MSLN-CAR T Cells Secreting PD-1 Nanobodies for the Treatment of MSLN-positive Advanced Solid Tumors | (Other Hospital/Academic/Medical Center) Shanghai Cell Therapy Group Co. | China | Broader basket including non-gynecologic cancers | tubo-ovarian/peritoneal |
| NCT05366478 | NCT05366478 | ClinicalTrials.gov | Both | A Single-Arm, Open-Label, Exploratory Study to Evaluate Safety and Efficacy of LM103 Injection in the Treatment of Advanced Solid Tumors | Suzhou BlueHorse Therapeutics Co. | China | Broader basket including non-gynecologic cancers | cervical |
| 431389 |  | Other registry | TrialTrove | A Clinical Study of TSC-201 in Patients with Cancer | TScan Therapeutics |  | Broader basket including non-gynecologic cancers | tubo-ovarian/peritoneal; cervical |
| 430435 | CTR20260154 | China CDE CTR | TrialTrove | A single-arm, open-label phase I clinical trial of autologous tumor-infiltrating lymphocyte (TIL) injection for the treatment of advanced recurrent or metastatic cervical cancer | Guangzhou Zhiling Biopharma Guangzhou Hearty Care Biotechnology Co. | China | Broader basket including non-gynecologic cancers | cervical |
| NCT05342506 | NCT05342506 | ClinicalTrials.gov | Both | A Open, Single Arm, Phase IIa Clinical Study on the Safety, Pharmacokinetics, and Efficacy of ScTIL (Genetically Modified Tumor Infiltrating Lymphocytes) in the Treatment of Gynecological Malignancies | Peking Union Medical College Hospital, CAMS Chineo Med (Beijing) Co. | China | Broader basket including non-gynecologic cancers | tubo-ovarian/peritoneal; cervical |
| NCT05302037 | NCT05302037 | ClinicalTrials.gov | TrialTrove | A Phase I Trial to Evaluate Allogeneic NKG2DL-targeting Chimeric Antigen Receptor-grafted gamma delta T Cells (CTM-N2D) in Subjects With Advanced Solid Tumours or Haematological Malignancies | Nuo Therapeutics {Cytomedix} (Other Hospital/Academic/Medical Center) | Singapore | Broader basket including non-gynecologic cancers | tubo-ovarian/peritoneal |
| 427237 | ChiCTR2200057362 | ChiCTR | TrialTrove | Clinical Study on the Safety and Effectiveness of CART Cells in the Treatment of Advanced Gynecological Tumors | (Other Hospital/Academic/Medical Center) Innovative Cellular Therapeutics Co. | China | Broader basket including non-gynecologic cancers | tubo-ovarian/peritoneal; cervical; endometrial/uterine corpus; vulvar; vaginal |
| NCT05238818 | NCT05238818 | ClinicalTrials.gov | TrialTrove | Single Arm Phase I Trail of Autologous Tumor Infiltrating Lymphocyte Injection (GT202) in the Treatment of Metastatic or Recurrent Gynecological Tumors | (Other Hospital/Academic/Medical Center) | China | Broader basket including non-gynecologic cancers | tubo-ovarian/peritoneal; cervical; endometrial/uterine corpus |
| NCT05211557 | NCT05211557 | ClinicalTrials.gov | Both | A Single-Arm, Open-Label Study to Evaluate Safety and Efficacy of Fully Human B7H3 CAR-T in Treating Patients With Recurrent Malignant Ovarian Cancer | (Other Hospital/Academic/Medical Center) | China | Broader basket including non-gynecologic cancers | tubo-ovarian/peritoneal |
| NCT05194735 | NCT05194735 | ClinicalTrials.gov | Both | Phase I/II Study of Autologous T Cells Engineered Using the Sleeping Beauty System to Express T-Cell Receptors (TCRs) Reactive Against Cancer-specific Mutations in Subjects With Solid Tumors | Alaunos Therapeutics | United States | Broader basket including non-gynecologic cancers | tubo-ovarian/peritoneal; endometrial/uterine corpus |
| 421887 | ChiCTR2100054914 | ChiCTR | TrialTrove | Clinical Study On The Safety And Efficacy Of CART Cells In The Treatment Of Advanced MSLN Positive Tumors | (Other Hospital/Academic/Medical Center) Innovative Cellular Therapeutics Co. | China | Broader basket including non-gynecologic cancers | tubo-ovarian/peritoneal; cervical; endometrial/uterine corpus |
| NCT05274451 | NCT05274451 | ClinicalTrials.gov | Both | A Phase I Study to Assess the Safety and Efficacy of LYL797, ROR1-Targeting CAR T Cells, in Adults With Relapsed and/or Refractory Solid-Tumor Malignancies | Lyell Immunopharma | United States | Broader basket including non-gynecologic cancers | tubo-ovarian/peritoneal; endometrial/uterine corpus |
| NCT05152797 | NCT05152797 | ClinicalTrials.gov | TrialTrove | Efficacy and Safety of Autologous Tumor-infiltrating Lymphocytes in Treatment of Patients With Advanced or Metastatic Refractory Gynecological Cancer: a Prospective Multicenter One-arm Phase l/ll Trial | (Other Hospital/Academic/Medical Center) | China | Broader basket including non-gynecologic cancers | tubo-ovarian/peritoneal; endometrial/uterine corpus |
| NCT05141253 | NCT05141253 | ClinicalTrials.gov | TrialTrove | A Single-Center Exploratory Clinical Study to Evaluate the Safety and Efficacy of RD133 in Subjects With Relapsed or Refractory MSLN-Positive Solid Tumors | (Other Hospital/Academic/Medical Center) Nanjing IASO Biotechnology Co. {Nanjing Iaso Biotherapeutics Co.}/Shanghai Iaso Biotechnology Co. | China | Broader basket including non-gynecologic cancers | tubo-ovarian/peritoneal |
| NCT05357027 | NCT05357027 | ClinicalTrials.gov | Both | A Phase I/II Clinical Study of TC-E202 injection Intended for the Treatment of Patients with HPV16-positive recurrent or metastatic cervical cancer that has failed previous treatment | TCRCure Biopharma Technology Co. Fudan University - Shanghai, China | China | Single gynecologic disease family specific | cervical |
| NCT05122221 | NCT05122221 | ClinicalTrials.gov | Both | A Phase I Study to Evaluate the Safety, Tolerance and Efficacy of CRTE7A2-01 TCR-T Cell for HPV16 Positive Advanced Cervical, Anal, or Head and Neck Cancers | Corregene Biotechnology Co. | China | Broader basket including non-gynecologic cancers | cervical |
| NCT05098171 | NCT05098171 | ClinicalTrials.gov | Both | A Clinical Study on Signal Switch Receptor Modified Tumor Infiltrating Lymphocytes Injection (GC201 TIL) in Patients With Gynecologic Tumors | Shanghai Juncell Therapeutics Co. {Shanghai Gencells Therapeutics Co.} (Other Hospital/Academic/Medical Center) | China | Broader basket including non-gynecologic cancers | tubo-ovarian/peritoneal; cervical; endometrial/uterine corpus; vulvar; vaginal |
| NCT05089266 | NCT05089266 | ClinicalTrials.gov | TrialTrove | Study of αPD1-MSLN-CAR T Cells to Evaluate the Safety, Tolerability, and Effectiveness for Patients With MSLN-positive Advanced Solid Tumors. | Shanghai Cell Therapy Group Co. | China | Broader basket including non-gynecologic cancers | tubo-ovarian/peritoneal |
| NCT05499715 | NCT05499715 | ClinicalTrials.gov | TrialTrove | A Phase I Clinical Study to Evaluate the Safety, the Tolerability, the Pharmacokinetic Characteristics and the Efficacy of ScTIL Injection (Genetically Modified Tumor Infiltrating Lymphocytes) in the Treatment of Advanced Malignant Solid Tumors | (Other Hospital/Academic/Medical Center) Chineo Med (Beijing) Co. | China | Broader basket including non-gynecologic cancers | cervical |
| NCT04981691 | NCT04981691 | ClinicalTrials.gov | TrialTrove | An Open, Single-center, Exploratory Clinical Trial to Evaluate the Safety and Efficacy of mRNA CAR-mesothelin T Cells in Patients With Advanced Refractory Solid Tumors | (Other Hospital/Academic/Medical Center) UTC Therapeutics | China | Broader basket including non-gynecologic cancers | tubo-ovarian/peritoneal |
| NCT05393635 | NCT05393635 | ClinicalTrials.gov | Both | A Phase II, Open-Label, Multicenter Study Evaluating the Safety, Feasibility, and Preliminary Efficacy of ITIL-168 with Pembrolizumab in Subjects with Advanced Solid Tumors | Instil Bio | United States | Broader basket including non-gynecologic cancers | cervical |
| 403252 |  | Other registry | TrialTrove | A Phase Ib Study of CAR-T Therapy in Patients with Ovarian Cancer | Cartherics Peter MacCallum Cancer Centre, Australia |  | Single gynecologic disease family specific | tubo-ovarian/peritoneal |
| NCT04766320 | NCT04766320 | ClinicalTrials.gov | Both | A Clinical Safety and Efficacy Study on TIL for the Treatment of r/r Gynecologic Tumors | (Other Hospital/Academic/Medical Center) Shanghai Juncell Therapeutics Co. {Shanghai Gencells Therapeutics Co.} | China | Broader basket including non-gynecologic cancers | tubo-ovarian/peritoneal; cervical; endometrial/uterine corpus |
| NCT05107739 | NCT05107739 | ClinicalTrials.gov | Both | A Phase I Safety and Tolerability Study of DeTIL-0255 in Adults With Advanced Malignancies | Nurix Therapeutics | United States | Broader basket including non-gynecologic cancers | tubo-ovarian/peritoneal; cervical; endometrial/uterine corpus; vulvar; vaginal |
| 394743 | ChiCTR2100042320 | ChiCTR | TrialTrove | A Clinical Study on the Treatment of Ovarian Cancer with CAR-T cells Targeted by Mesothelin Antigen | (Other Hospital/Academic/Medical Center) Shanghai Yihao Biotechnology Co. | China | Broader basket including non-gynecologic cancers | tubo-ovarian/peritoneal |
| NCT04674488 | NCT04674488 | ClinicalTrials.gov | TrialTrove | An Exploratory Study Of TILs Treatment For Metastatic Or Recurrent Cervical Cancer | Origincell Technology Group Co. {OriCell Therapeutics {Shanghai OriginCell Therapeutics Co.}} (Other Hospital/Academic/Medical Center) | China | Broader basket including non-gynecologic cancers | cervical |
| NCT05239143 | NCT05239143 | ClinicalTrials.gov | Both | A Phase I Dose Escalation and Expanded Cohort Study of P-MUC1C-ALLO1 in Adult Subjects with Advanced or Metastatic Solid Tumors | Poseida Therapeutics | United States | Broader basket including non-gynecologic cancers | tubo-ovarian/peritoneal |
| NCT04670068 | NCT04670068 | ClinicalTrials.gov | Both | A Phase I Study of Autologous Activated T-cells Targeting the B7-H3 Antigen in Subjects With Recurrent Epithelial Ovarian Cancer | UNC Lineberger Comprehensive Cancer Center National Institutes of Health National Institutes of Health/National Cancer Institute | United States | Broader basket including non-gynecologic cancers | tubo-ovarian/peritoneal |
| 389985 |  | Other registry | TrialTrove | A Cinical Study of Mesothelin CAR-T Cells in Patients with Mesothelioma, Pancreatic, Ovarian, Breast and Endometrial Cancers | Avalon GloboCare/Hebei Senlang Biotechnology Co. |  | Broader basket including non-gynecologic cancers | tubo-ovarian/peritoneal; endometrial/uterine corpus |
| 389901 |  | Other registry | TrialTrove | A Phase I Study of Folate Receptor Alpha CAR-T therapy (TmFRa-01) in Ovarian Cancer | Gilead Sciences/Kite Pharma University of Pennsylvania | United States | Single gynecologic disease family specific | tubo-ovarian/peritoneal |
| NCT04627740 | NCT04627740 | ClinicalTrials.gov | TrialTrove | A Single-Arm, Single-Center, Open-Label Pilot Study of Anti-ALPP CART-cells in Patient With Alkaline Phosphatase, Placental (ALPP)-Positive Advanced Solid Tumor | (Other Hospital/Academic/Medical Center) Beijing TCRCure Biological Technology Co. | China | Broader basket including non-gynecologic cancers | tubo-ovarian/peritoneal; endometrial/uterine corpus |
| NCT04611126 | NCT04611126 | ClinicalTrials.gov | Both | T-cell Therapy in Combination With Nivolumab, Relatlimab and Ipilimumab for Patients With Advanced Ovarian-, Fallopian Tube- and Primary Peritoneal Cancer | Herlev and Gentofte Hospital {Herlev Hospital} | Denmark | Broader basket including non-gynecologic cancers | tubo-ovarian/peritoneal |
| NCT04562298 | NCT04562298 | ClinicalTrials.gov | Both | A Phase I Clinical Study to Evaluate the Safety, Tolerability, and Efficacy of LCAR-M23, a CAR-T Cell Therapy Targeting MSLN in Patients With Relapsed and Refractory Epithelial Ovarian Cancer | (Other Hospital/Academic/Medical Center) Nanjing Legend Biotechnology Co. | China | Single gynecologic disease family specific | tubo-ovarian/peritoneal |
| NCT04556669 | NCT04556669 | ClinicalTrials.gov | Both | Feasibility And Safety Of Anti-PD-L1 Armored Anti-CD22 CAR-T/CAR-TILS Targeting Patients With Solid Tumors | Avalon GloboCare/Hebei Senlang Biotechnology Co. | China | Broader basket including non-gynecologic cancers | cervical |
| NCT04483778 | NCT04483778 | ClinicalTrials.gov | TrialTrove | Phase I Study of B7H3 CAR T Cell Immunotherapy for Recurrent/Refractory Solid Tumors in Children and Young Adults | Seattle Children's Hospital | United States | Broader basket including non-gynecologic cancers | tubo-ovarian/peritoneal |
| NCT04476251 | NCT04476251 | ClinicalTrials.gov | Both | A Pilot Study of E7 TCR T Cell Induction Immunotherapy for Stage IIB-IVA Cervical Cancer | National Institutes of Health/National Cancer Institute | United States | Broader basket including non-gynecologic cancers | cervical |
| NCT04443296 | NCT04443296 | ClinicalTrials.gov | Both | Study of Tumor Infiltrating Lymphocytes Following Concurrent Chemoradiotherapy in the Treatment of Patients With Cervical Carcinoma | (Other Hospital/Academic/Medical Center) | China | Single gynecologic disease family specific | cervical |
| NCT04389229 | NCT04389229 | ClinicalTrials.gov | Both | Protocol Title: An Open Label, Multi-centre Phase I/IIa Study of Modified and Unmodified Autologous Tumour Infiltrating Lymphocytes (TIL) in Patients With Platinum-resistant Ovarian Cancer | Instil Bio {Immetacyte} | United Kingdom | Broader basket including non-gynecologic cancers | tubo-ovarian/peritoneal |
| 372638 |  | Other registry | TrialTrove | A Phase I Safety and Efficacy Trial of KK-LC-1 TCR Therapy in Patients with Kita-Kyushu Lung Cancer Antigen 1 (KK-LC-1) Expressing Malignancies Including Triple Negative Breast Cancer, Non-Small Cell Lung Cancer, Pancreatic Cancer, Gastric and Liver Cancers | T-Cure Bioscience |  | Broader basket including non-gynecologic cancers | cervical |
| NCT05035407 | NCT05035407 | ClinicalTrials.gov | Both | A Phase I Trial of T Cell Receptor Gene Therapy Targeting KK-LC-1 for Gastric, Breast, Cervical, Lung and Other KK-LC-1 Positive Epithelial Cancers | National Institutes of Health/National Cancer Institute (Other Hospital/Academic/Medical Center) T-Cure Bioscience | United States | Broader basket including non-gynecologic cancers | cervical |
| NCT04318964 | NCT04318964 | ClinicalTrials.gov | TrialTrove | An Open, Single Arm and Early Clinical Study of TAEST16001 in the Treatment of Solid Tumor Mainly Containing Soft Tissue Sarcoma With Positive Expression of Tumor Antigen NY-ESO-1 (HLA-A * 02:01) | (Other Academic Cancer Center) Guangzhou Xiangxue Pharmaceutical Co./Xiangxue Life Sciences | China | Broader basket including non-gynecologic cancers | tubo-ovarian/peritoneal |
| NCT04503278 | NCT04503278 | ClinicalTrials.gov | Both | Phase I/IIa, First-in-human (FIH), Open-label, Dose Escalation Trial With Expansion Cohorts to Evaluate Safety and Preliminary Efficacy of CLDN6 CAR-T With or Without CLDN6 RNA-LPX in Patients With CLDN6-positive Relapsed or Refractory Advanced Solid Tumors | BioNTech | Australia; Germany; Netherlands; Sweden | Broader basket including non-gynecologic cancers | tubo-ovarian/peritoneal; endometrial/uterine corpus |
| NCT04025216 | NCT04025216 | ClinicalTrials.gov | Both | A Phase I Open-Label, Multi-Center First in Human Study of TnMUC1-Targeted Genetically-Modified Chimeric Antigen Receptor T Cells in Patients With Advanced TnMUC1-Positive Solid Tumors and Multiple Myeloma | Gilead Sciences/Kite Pharma {Tmunity Therapeutics} | United States | Broader basket including non-gynecologic cancers | tubo-ovarian/peritoneal |
| NCT03992326 | NCT03992326 | ClinicalTrials.gov | TrialTrove | Phase Ib Study To Assess The Feasibility And Safety Of Adoptive Transfer Of Autologous Tumor-Infiltrating Lymphocytes In Advanced Solid Tumors | (Other Hospital/Academic/Medical Center) | Switzerland | Broader basket including non-gynecologic cancers | tubo-ovarian/peritoneal |
| NCT03970382 | NCT03970382 | ClinicalTrials.gov | Both | A Phase Ia/Ib, Open-label First-in-human Study of the Safety, Tolerability and Feasibility of Gene-edited Autologous NeoTCR-T Cells (NeoTCR-P1) Administered as a Single Agent or in Combination With Anti-PD-1 to Patients With Locally Advanced or Metastatic Solid Tumors | PACT Pharma | United States | Broader basket including non-gynecologic cancers | tubo-ovarian/peritoneal |
| NCT03912831 | NCT03912831 | ClinicalTrials.gov | TrialTrove | A Phase I Study Evaluating the Safety and Efficacy of HPV16 E7 T Cell Receptor Engineered T Cells (KITE-439) in HLA-A*02:01+ Subjects With Relapsed/Refractory HPV16+ Cancers | Gilead Sciences/Kite Pharma | United States | Broader basket including non-gynecologic cancers | cervical |
| NCT03907527 | NCT03907527 | ClinicalTrials.gov | Both | Phase I/Ib Study Evaluating Benefit of PRGN-3005 UltraCAR-T (Autologous CAR T Cells) in Advanced Stage Platinum Resistant Ovarian Cancer Patients | Precigen, Inc. {Intrexon}/Precigen Fred Hutchinson Cancer Research Center National Institutes of Health/National Cancer Institute | United States | Broader basket including non-gynecologic cancers | tubo-ovarian/peritoneal |
| NCT04650451 | NCT04650451 | ClinicalTrials.gov | TrialTrove | A Phase I/II, Open-Label, Multicenter, Non-Randomized, Safety and Activity Study of HER2-Targeted Dual Switch CAR-T Cells (BPX-603) In Subjects With Previously Treated Advanced HER2-Positive Solid Tumors | Bellicum Pharmaceuticals | United States | Broader basket including non-gynecologic cancers | tubo-ovarian/peritoneal; endometrial/uterine corpus |
| NCT03745326 | NCT03745326 | ClinicalTrials.gov | TrialTrove | A Phase I/II Study Administering Peripheral Blood Lymphocytes Transduced with a Murine T-Cell Receptor Recognizing the G12D Variant of Mutated RAS in HLA-A*11:01 Patients | National Institutes of Health/National Cancer Institute | United States | Broader basket including non-gynecologic cancers | tubo-ovarian/peritoneal |
| NCT03686124 | NCT03686124 | ClinicalTrials.gov | Both | Phase I/II Study Evaluating Genetically Modified Autologous T Cells Expressing a TCR Recognizing a Cancer/Germline Antigen as Monotherapy or in Combination With Atezolizumab in Patients With Recurrent and/or Refractory Solid Tumors | immatics biotechnologies | Germany; United States | Broader basket including non-gynecologic cancers | tubo-ovarian/peritoneal; endometrial/uterine corpus |
| NCT03638206 | NCT03638206 | ClinicalTrials.gov | Both | Autologous Immunotherapy With Multi-target Gene-modified CAR-T/TCR-T Cell For Malignancies | Shenzhen BinDeBio (Other Hospital/Academic/Medical Center) | China | Broader basket including non-gynecologic cancers | tubo-ovarian/peritoneal |
| NCT03610490 | NCT03610490 | ClinicalTrials.gov | Both | Clinical Study to Assess Efficacy and Safety of MDA-TIL (Autologous Expanded Tumor Infiltrating Lymphocytes) Across Multiple Tumor Types | MD Anderson Cancer Center, University of Texas Bristol-Myers Squibb Iovance Biotherapeutics {Lion Biotechnologies} National Institutes of Health/National Cancer Institute | United States | Broader basket including non-gynecologic cancers | tubo-ovarian/peritoneal |
| NCT03578406 | NCT03578406 | ClinicalTrials.gov | Both | Two-Arm Open-Labeled Trial of HPV-E6-Specific TCR-T Cells With or Without Anti-PD1 Auto-secreted Element in the Treatment of HPV-Positive Head and Neck Carcinoma or Cervical Cancer | Xinqiao Hospital of Chongqing Beijing TCRCure Biological Technology Co. | China | Broader basket including non-gynecologic cancers | cervical |
| 327082 | ChiCTR1800016805 | ChiCTR | TrialTrove | A Clinical Study in Patients with Solid Tumor Using Personalized Multi-Targets Neoantigen-reactive T Cells Combined Vaccine. | (Other Hospital/Academic/Medical Center) Chineo Med (Beijing) Co. | China | Broader basket including non-gynecologic cancers | cervical |
| 326892 | ChiCTR1800016764 | ChiCTR | TrialTrove | Chimeric Antigen Receptor Gene-Modified Autologous T cells Targeting Mesothelin for Advanced Ovarian Cancer. | (Other Hospital/Academic/Medical Center) (Other Industry Sponsor) | China | Broader basket including non-gynecologic cancers | tubo-ovarian/peritoneal |
| NCT03545815 | NCT03545815 | ClinicalTrials.gov | TrialTrove | Phase I Study to Evaluate Treatment of CRISPR-Cas9 Mediated PD-1 and TCR Gene-knocked out Chimeric Antigen Receptor (CAR) T Cells in Patients With Mesothelin Positive Multiple Solid Tumors. | (Other Hospital/Academic/Medical Center) | China | Broader basket including non-gynecologic cancers | tubo-ovarian/peritoneal |
| NCT03449108 | NCT03449108 | ClinicalTrials.gov | Both | Clinical Study to Assess Efficacy and Safety of LN-145/LN-145-S1 (Autologous Centrally Manufactured Tumor Infiltrating Lymphocytes) Across Multiple Tumor Types | MD Anderson Cancer Center, University of Texas Iovance Biotherapeutics {Lion Biotechnologies} National Institutes of Health/National Cancer Institute | United States | Broader basket including non-gynecologic cancers | tubo-ovarian/peritoneal |
| NCT04102436 | NCT04102436 | ClinicalTrials.gov | Both | A Phase I/II Study Using the Administration of Autologous T-Cells Engineered Using the Sleeping Beauty Transposon/Transposase System to Express T-Cell Receptors Reactive Against Mutated Neoantigens in Patients With Metastatic Cancer | National Institutes of Health/National Cancer Institute | United States | Broader basket including non-gynecologic cancers | tubo-ovarian/peritoneal |
| NCT03412526 | NCT03412526 | ClinicalTrials.gov | Both | A Phase II, Single-Center, Open Label Study of Autologous, Adoptive Cell Therapy Following a Reduced Intensity, Non-myeloablative, Lymphodepleting Induction Regimen in Metastatic Ovarian | Sheba Medical Center | Israel | Broader basket including non-gynecologic cancers | tubo-ovarian/peritoneal |
| NCT03907852 | NCT03907852 | ClinicalTrials.gov | TrialTrove | A Phase I/II Single Arm Open-Label Clinical Trial of Gavocabtagene Autoleucel (Gavo-cel) in Patients With Advanced Mesothelin-Expressing Cancer. | TCR2 Therapeutics Bristol-Myers Squibb | Canada; United States | Broader basket including non-gynecologic cancers | tubo-ovarian/peritoneal |
| NCT03296137 | NCT03296137 | ClinicalTrials.gov | TrialTrove | Adoptive Cell Therapy Across Cancer Diagnoses. | (Other Hospital/Academic/Medical Center) | Denmark | Broader basket including non-gynecologic cancers | tubo-ovarian/peritoneal; cervical; endometrial/uterine corpus; vulvar; vaginal |
| NCT04072263 | NCT04072263 | ClinicalTrials.gov | Both | Adoptive T Cell Therapy in Patients With Recurrent Ovarian Cancer. | Leiden University Medical Center | Netherlands | Broader basket including non-gynecologic cancers | tubo-ovarian/peritoneal |
| NCT03287674 | NCT03287674 | ClinicalTrials.gov | Both | T-cell Therapy in Combination With Checkpoint Inhibitors for Patients With Advanced Ovarian-, Fallopian Tube- and Primary Peritoneal Cancer | Herlev and Gentofte Hospital {Herlev Hospital} | Denmark | Broader basket including non-gynecologic cancers | tubo-ovarian/peritoneal |
| NCT03158935 | NCT03158935 | ClinicalTrials.gov | Both | Phase Ib Trial of Pembrolizumab Administered in Combination With or Following Adoptive Cell Therapy- A Multiple Cohort Study; The ACTIVATE (Adoptive Cell Therapy InVigorated to Augment Tumor Eradication) Trial | University Health Network, Toronto Merck & Co./Merck Sharp & Dohme (MSD) | Canada | Broader basket including non-gynecologic cancers | tubo-ovarian/peritoneal |
| NCT03159585 | NCT03159585 | ClinicalTrials.gov | Both | Application of NY-ESO-1-specific TCR Affinity Enhancing Specific T Cell Therapy (TAEST16001) in Solid Tumors Except Non Small Cell Lung Cancer,Including Liver Cancer,Gastric Cancer,Esophageal Cancer and so on. | (Other Hospital/Academic/Medical Center) Guangzhou Xiangxue Pharmaceutical Co. Guangzhou Xiangxue Pharmaceutical Co./Xiangxue Life Sciences | China | Broader basket including non-gynecologic cancers | tubo-ovarian/peritoneal |
| NCT03108495 | NCT03108495 | ClinicalTrials.gov | Both | A Phase II, Multicenter Study to Evaluate the Efficacy and Safety Using Autologous Tumor Infiltrating Lymphocytes (LN-145) in Patients with Recurrent, Metastatic or Persistent Cervical Carcinoma | Iovance Biotherapeutics {Lion Biotechnologies} | France; Germany; Hungary; Italy; Netherlands; Spain; Switzerland; United Kingdom; United States | Broader basket including non-gynecologic cancers | cervical |
| NCT03132922 | NCT03132922 | ClinicalTrials.gov | Both | Phase I Dose Escalation, Multi-tumor Study to Assess the Safety, Tolerability and Antitumor Activity of Genetically Engineered MAGE-A4C1032T in HLA-A2+ Subjects With MAGE-A4 Positive Tumors | AdaptImmune | Canada; United States | Broader basket including non-gynecologic cancers | tubo-ovarian/peritoneal |
| NCT02930993 | NCT02930993 | ClinicalTrials.gov | TrialTrove | A Safety and Efficacy Study of Autologous T Cells Engineered to Express Chimeric Antigen Receptor Targeting Mesothelin in Patients With Recurred or Metastatic Malignant Tumors | (Other Hospital/Academic/Medical Center) Marino Biotechnology Co. | China | Broader basket including non-gynecologic cancers | tubo-ovarian/peritoneal |
| NCT02858310 | NCT02858310 | ClinicalTrials.gov | Both | A Phase I/II Trial of T Cell Receptor Gene Therapy Targeting HPV-16 E7 for HPV-Associated Cancers | National Institutes of Health/National Cancer Institute | United States | Broader basket including non-gynecologic cancers | cervical; vulvar; vaginal |
| NCT02650986 | NCT02650986 | ClinicalTrials.gov | Both | A Phase I/IIa Study of TGF beta Blockade in TCR-Engineered T-Cell Cancer Immunotherapy in Patients With Advanced Malignancies | Roswell Park Cancer Institute (Other government agency) National Institutes of Health/National Cancer Institute | United States | Broader basket including non-gynecologic cancers | tubo-ovarian/peritoneal |
| NCT02498912 | NCT02498912 | ClinicalTrials.gov | TrialTrove | A Phase I Clinical Trial of Cyclophosphamide Followed by Intravenous and Intraperitoneal Infusion of Autologous T Cells Genetically Engineered to Secrete IL-12 and to Target the MUC16ecto Antigen in Patients With Recurrent MUC16ecto+ Solid Tumors | Memorial Sloan-Kettering Cancer Center Bristol-Myers Squibb/Celgene/Juno Therapeutics Stanford University Medical Center | United States | Broader basket including non-gynecologic cancers | tubo-ovarian/peritoneal |
| NCT02159716 | NCT02159716 | ClinicalTrials.gov | Both | Phase I Study of Chimeric Antigen Receptor Modified T Cells in Patients with Mesothelin Expressing Cancers | University of Pennsylvania Novartis | United States | Broader basket including non-gynecologic cancers | tubo-ovarian/peritoneal |
| NCT02111850 | NCT02111850 | ClinicalTrials.gov | Both | A Phase I/II Study of the Treatment of Metastatic Cancer That Expresses MAGE-A3 Using Lymphodepleting Conditioning Followed by Infusion of HLA-DP0401/0402 Restricted Anti-MAGE-A3 TCR-Gene Engineered Lymphocytes and Aldesleukin | National Institutes of Health/National Cancer Institute Gilead Sciences/Kite Pharma | Belgium; United States | Broader basket including non-gynecologic cancers | cervical |
| NCT01883297 | NCT01883297 | ClinicalTrials.gov | Both | Phase I Study Evaluating the Feasibility and Safety of Infusion of "Re-Stimulated" Autologous TILs Followed by Low-Dose IL-2 Therapy in Patients with Platinum Resistant High Grade Serous Ovarian, Fallopian Tube, or Primary Peritoneal Cancer | University Health Network, Toronto | Canada | Single gynecologic disease family specific | tubo-ovarian/peritoneal |
| NCT01585428 | NCT01585428 | ClinicalTrials.gov | Both | A Phase II Study of Lymphodepletion Followed by Autologous Tumor-Infiltrating Lymphocytes and High-Dose Aldesleukin for Human Papillomavirus-Associated Cancers | National Institutes of Health/National Cancer Institute | United States | Broader basket including non-gynecologic cancers | cervical; vulvar; vaginal |
| NCT01567891 | NCT01567891 | ClinicalTrials.gov | Both | A Phase I/IIa, Open Label Clinical Trial Evaluating the Safety and Efficacy of Autologous T Cells Expressing Enhanced TCRs Specific for NY-ESO-1 in Patients With Recurrent or Treatment Refractory Ovarian Cancer | AdaptImmune AstraZeneca Cardiff Oncology {Trovagene} GlaxoSmithKline iTeos Therapeutics | United States | Broader basket including non-gynecologic cancers | tubo-ovarian/peritoneal |
| 154752 |  | Other registry | TrialTrove | Phase II Exploratory Pilot Study of Contego in Patients With Ovarian Cancer | Iovance Biotherapeutics {Lion Biotechnologies {Genesis Biopharma}} |  | Single gynecologic disease family specific | tubo-ovarian/peritoneal |
| NCT01174121 | NCT01174121 | ClinicalTrials.gov | Both | A Phase II Study Using Short-Term Cultured, Autologous Tumor-Infiltrating Lymphocytes Following a Lymphodepleting Regimen in Metastatic Cancers Plus the Administration of Pembrolizumab | National Institutes of Health/National Cancer Institute | United States | Broader basket including non-gynecologic cancers | tubo-ovarian/peritoneal; endometrial/uterine corpus |
| 1772 |  | Other registry | TrialTrove | Phase II Pilot Study of Intravenous Paclitaxel/Cisplatin plus Intraperitoneal IL-2-Expanded TIL and Low-Dose IL-2 vs Intraperitoneal Low-Dose IL-2 Alone in Stage IIIC/IV Mullerian Cancer. | National Institutes of Health/National Cancer Institute | United States | Single gynecologic disease family specific | tubo-ovarian/peritoneal |
| NCT05686226 | NCT05686226 | ClinicalTrials.gov | ClinicalTrials.gov | E7 TCR-T Cell Immunotherapy for Human Papillomavirus (HPV) Associated Cancers | Christian Hinrichs | United States | Broader basket including non-gynecologic cancers | cervical; vulvar; vaginal |
| NCT05639972 | NCT05639972 | ClinicalTrials.gov | ClinicalTrials.gov | E7 T-cell Receptor (TCR) -T Cell Induction Therapy for Locoregionally Advanced HPV-associated Cancers | Christian Hinrichs | United States | Broader basket including non-gynecologic cancers | cervical; vulvar; vaginal |
| NCT07617753 | NCT07617753 | ClinicalTrials.gov | ClinicalTrials.gov | Dual-Targeting CAR-NK Cells for Recurrent Ovarian Cancer (MSLN, FRα, MUC16) pt2 | Beijing Biotech | China | Single gynecologic disease family specific | tubo-ovarian/peritoneal |
| NCT07589543 | NCT07589543 | ClinicalTrials.gov | ClinicalTrials.gov | Dual-Target CAR-NK Cells in Recurrent or Refractory Epithelial Ovarian Cancer | Beijing Biotech | China | Single gynecologic disease family specific | tubo-ovarian/peritoneal |
| NCT07410494 | NCT07410494 | ClinicalTrials.gov | ClinicalTrials.gov | Biomarker-Guided Allogeneic Single-Target or Dual-Target CAR-NK Cell Therapy for Advanced Solid Tumors | Essen Biotech | China | Broader basket including non-gynecologic cancers | tubo-ovarian/peritoneal |
| NCT07480954 | NCT07480954 | ClinicalTrials.gov | ClinicalTrials.gov | Dual-Targeting CAR-NK Cells for Recurrent Ovarian Cancer (MSLN, FRα, MUC16) | Beijing Biotech | China | Single gynecologic disease family specific | tubo-ovarian/peritoneal |
| NCT05776355 | NCT05776355 | ClinicalTrials.gov | ClinicalTrials.gov | NKG2D CAR-NK & Ovarian Cancer | Hangzhou Cheetah Cell Therapeutics Co., Ltd | China | Single gynecologic disease family specific | tubo-ovarian/peritoneal |
| NCT05410717 | NCT05410717 | ClinicalTrials.gov | ClinicalTrials.gov | CLDN6/GPC3/Mesothelin/AXL-CAR-NK Cell Therapy for Advanced Solid Tumors | Second Affiliated Hospital of Guangzhou Medical University | China | Broader basket including non-gynecologic cancers | tubo-ovarian/peritoneal; endometrial/uterine corpus |
| NCT06215950 | NCT06215950 | ClinicalTrials.gov | ClinicalTrials.gov | A Clinical Research About CD70-targeted CAR-T in the Treatment of CD70-positive Advanced/Metastatic Gynecologic Cancer | Chongqing Precision Biotech Co., Ltd | China | Broader basket including non-gynecologic cancers | tubo-ovarian/peritoneal; cervical |
| NCT03692637 | NCT03692637 | ClinicalTrials.gov | ClinicalTrials.gov | Study of Anti-Mesothelin Car NK Cells in Epithelial Ovarian Cancer | Allife Medical Science and Technology Co., Ltd. |  | Single gynecologic disease family specific | tubo-ovarian/peritoneal |
| NCT05225363 | NCT05225363 | ClinicalTrials.gov | ClinicalTrials.gov | Modified Immune Cells (TAG72-CAR T Cells) for the Treatment of Patients With Platinum Resistant Epithelial Ovarian Cancer | City of Hope Medical Center | United States | Single gynecologic disease family specific | tubo-ovarian/peritoneal |
| NCT07435701 | NCT07435701 | ClinicalTrials.gov | ClinicalTrials.gov | Intraperitoneal SK-NK Cell Injection for Advanced Ovarian Cancer With Massive Ascites | Cancer Institute and Hospital, Chinese Academy of Medical Sciences | China | Single gynecologic disease family specific | tubo-ovarian/peritoneal |
| NCT05922930 | NCT05922930 | ClinicalTrials.gov | ClinicalTrials.gov | Study of TROP2 CAR Engineered IL15-transduced Cord Blood-derived NK Cells Delivered Intraperitoneally for the Management of Platinum Resistant Ovarian Cancer, Mesonephric-like Adenocarcinoma, and Pancreatic Cancer | M.D. Anderson Cancer Center | United States | Broader basket including non-gynecologic cancers | tubo-ovarian/peritoneal |
| NCT05856643 | NCT05856643 | ClinicalTrials.gov | ClinicalTrials.gov | Single-arm, Open-label Clinical Study of SZ011 in the Treatment of Ovarian Epithelial Carcinoma | Shantou University Medical College | China | Single gynecologic disease family specific | tubo-ovarian/peritoneal |
| NCT07410676 | NCT07410676 | ClinicalTrials.gov | ClinicalTrials.gov | EBNK-001 Allogeneic NK Cells With Low-Dose IL-15 ± Pembrolizumab in Advanced Solid Tumors | Essen Biotech | China | Broader basket including non-gynecologic cancers | tubo-ovarian/peritoneal |
| NCT05963100 | NCT05963100 | ClinicalTrials.gov | ClinicalTrials.gov | Clinical Study of TCR-like CAR-T Cell Targeted MSLN in the Treatment of Ovarian Cancer | Zhongda Hospital | China | Single gynecologic disease family specific | tubo-ovarian/peritoneal |
| NCT02118285 | NCT02118285 | ClinicalTrials.gov | ClinicalTrials.gov | Intraperitoneal Natural Killer Cells and INCB024360 for Recurrent Ovarian, Fallopian Tube, and Primary Peritoneal Cancer | Masonic Cancer Center, University of Minnesota | United States | Single gynecologic disease family specific | tubo-ovarian/peritoneal |
| NCT03691376 | NCT03691376 | ClinicalTrials.gov | ClinicalTrials.gov | Genetically Engineered Cells (NY-ESO-1 TCR Engineered T Cells and HSCs) After Melphalan Conditioning Regimen in Treating Patients With Recurrent or Refractory Ovarian, Fallopian Tube, or Primary Peritoneal Cancer | Roswell Park Cancer Institute | United States | Single gynecologic disease family specific | tubo-ovarian/peritoneal |
| NCT01583686 | NCT01583686 | ClinicalTrials.gov | ClinicalTrials.gov | CAR T Cell Receptor Immunotherapy Targeting Mesothelin for Patients With Metastatic Cancer | National Cancer Institute (NCI) | United States | Broader basket including non-gynecologic cancers | tubo-ovarian/peritoneal; cervical |
| NCT03213964 | NCT03213964 | ClinicalTrials.gov | ClinicalTrials.gov | Intraperitoneal Delivery of Adaptive Natural Killer (NK) Cells (FATE-NK100) With Intraperitoneal Int | Masonic Cancer Center, University of Minnesota | United States | Single gynecologic disease family specific | tubo-ovarian/peritoneal |
| NCT01105650 | NCT01105650 | ClinicalTrials.gov | ClinicalTrials.gov | Allogeneic Natural Killer (NK) Cells for Ovarian, Fallopian Tube, Peritoneal and Metastatic Breast Cancer | Masonic Cancer Center, University of Minnesota | United States | Broader basket including non-gynecologic cancers | tubo-ovarian/peritoneal |
| NCT00652899 | NCT00652899 | ClinicalTrials.gov | ClinicalTrials.gov | Allogeneic Natural Killer Cells in Patients With Recurrent Ovarian Cancer, Fallopian Tube, and Primary Peritoneal Cancer | Masonic Cancer Center, University of Minnesota | United States | Single gynecologic disease family specific | tubo-ovarian/peritoneal |
| NCT06321484 | NCT06321484 | ClinicalTrials.gov | ClinicalTrials.gov | Intraperitoneal Cytokine-Induced Memory Like (CIML) Natural Killer (NK) Cells in Recurrent Ovarian Cancer | Dana-Farber Cancer Institute | United States | Single gynecologic disease family specific | tubo-ovarian/peritoneal |
| NCT07651124 | NCT07651124 | ClinicalTrials.gov | ClinicalTrials.gov | To Evaluate the Safety of Autologous Tumor-infiltrating Lymphocytes(TILs) for the Treatment of Recurrent Ovarian Cancer | Yong Wha Moon | South Korea | Single gynecologic disease family specific | tubo-ovarian/peritoneal |
| NCT05518253 | NCT05518253 | ClinicalTrials.gov | ClinicalTrials.gov | A Clinical Study of CD70-targeted CAR-T in the Treatment of CD70-positive Advanced/Metastatic Solid Tumors | Weijia Fang, MD | China | Broader basket including non-gynecologic cancers | tubo-ovarian/peritoneal; cervical |
| NCT05420545 | NCT05420545 | ClinicalTrials.gov | ClinicalTrials.gov | A Clinical Study of CD70-targeted CAR-T in the Treatment of CD70-positive Advanced/Metastatic Solid Tumors | Chongqing Precision Biotech Co., Ltd | China | Broader basket including non-gynecologic cancers | tubo-ovarian/peritoneal; cervical |
| NCT03539406 | NCT03539406 | ClinicalTrials.gov | ClinicalTrials.gov | Intraperitoneal Infusion of ex Vivo-cultured Allogeneic NK Cells in Recurrent Ovarian Carcinoma Patients | Radboud University Medical Center | Netherlands | Single gynecologic disease family specific | tubo-ovarian/peritoneal |
| NCT07613723 | NCT07613723 | ClinicalTrials.gov | ClinicalTrials.gov | A Study to Test the Safety, Tolerability and Effect of ZI-MA4-1 for Patients With Locally Advanced or Metastatic Solid Malignancies | Zelluna Immunotherapy AS | United Kingdom | Broader basket including non-gynecologic cancers | tubo-ovarian/peritoneal |
| NCT03356795 | NCT03356795 | ClinicalTrials.gov | ClinicalTrials.gov | Intervention of CAR-T Against Cervical Cancer | Shenzhen Geno-Immune Medical Institute | China | Single gynecologic disease family specific | cervical |
| NCT03585764 | NCT03585764 | ClinicalTrials.gov | ClinicalTrials.gov | MOv19-BBz CAR T Cells in aFR Expressing Recurrent High Grade Serous Ovarian, Fallopian Tube, or Primary Peritoneal Cancer | University of Pennsylvania | United States | Single gynecologic disease family specific | tubo-ovarian/peritoneal |
| NCT02580747 | NCT02580747 | ClinicalTrials.gov | ClinicalTrials.gov | Treatment of Relapsed and/or Chemotherapy Refractory Advanced Malignancies by CART-meso | Chinese PLA General Hospital | China | Broader basket including non-gynecologic cancers | tubo-ovarian/peritoneal; endometrial/uterine corpus |
| NCT07096583 | NCT07096583 | ClinicalTrials.gov | ClinicalTrials.gov | An Exploratory Study on NK Cell-assisted Prevention of Bone Marrow Suppression During Chemotherapy for Ovarian Cancer | Anhui Provincial Cancer Hospital | China | Single gynecologic disease family specific | tubo-ovarian/peritoneal |
| NCT03634501 | NCT03634501 | ClinicalTrials.gov | ClinicalTrials.gov | Clinical Study on Anti-tumor Effect Induced by Activated Primary Natural Killer（NK）Cells | Xuanwu Hospital, Beijing | China | Broader basket including non-gynecologic cancers | tubo-ovarian/peritoneal |
| NCT05372692 | NCT05372692 | ClinicalTrials.gov | ClinicalTrials.gov | Study of LD013 in Subjects With Refractory or Relapsed Mesothelin -Positive Ovarian Cancer | Weijia Fang, MD | China | Single gynecologic disease family specific | tubo-ovarian/peritoneal |
| NCT03916679 | NCT03916679 | ClinicalTrials.gov | ClinicalTrials.gov | MESO-CAR T Cells Therapy for Relapsed and Refractory Epithelial Ovarian Cancer | Second Affiliated Hospital, School of Medicine, Zhejiang University | China | Single gynecologic disease family specific | tubo-ovarian/peritoneal |
| NCT03735589 | NCT03735589 | ClinicalTrials.gov | ClinicalTrials.gov | Specialized Immune Cells (nCTLs) and a Vaccine (Alpha-type-1 Polarized Dendritic Cells) in Treating Patients With Stage II-IV Ovarian, Fallopian Tube, or Primary Peritoneal Cancer | Roswell Park Cancer Institute | United States | Single gynecologic disease family specific | tubo-ovarian/peritoneal |
| NCT06469281 | NCT06469281 | ClinicalTrials.gov | ClinicalTrials.gov | A Study to Learn if 27T51, a Mucin-16 (MUC16) Protein Targeting Immune Cell Therapy, Administered Alone or in Combination is Safe and How Well it Works for Adult Participants With Recurrent or Treatment Resistant Ovarian Cancers | Regeneron Pharmaceuticals | United States | Broader basket including non-gynecologic cancers | tubo-ovarian/peritoneal |
| NCT06395844 | NCT06395844 | ClinicalTrials.gov | ClinicalTrials.gov | Safety and Efficacy of Intraperitoneal Injection of METR-NK Cells as Neoadjuvant Therapy for Advanced Epithelial Ovarian Cancer | Anhui Provincial Cancer Hospital | China | Single gynecologic disease family specific | tubo-ovarian/peritoneal |
| NCT00101257 | NCT00101257 | ClinicalTrials.gov | ClinicalTrials.gov | Cellular Adoptive Immunotherapy in Treating Patients With Stage III or Stage IV Ovarian Cancer or Primary Peritoneal Cancer | Fred Hutchinson Cancer Center | United States | Single gynecologic disease family specific | tubo-ovarian/peritoneal |
| NCT03799913 | NCT03799913 | ClinicalTrials.gov | ClinicalTrials.gov | MESO-CAR T Cells Therapy for Relapsed and Refractory Ovarian Cancer | Zhejiang University | China | Single gynecologic disease family specific | tubo-ovarian/peritoneal |
| NCT03054298 | NCT03054298 | ClinicalTrials.gov | ClinicalTrials.gov | CAR T Cells in Mesothelin Expressing Cancers | University of Pennsylvania | United States | Broader basket including non-gynecologic cancers | tubo-ovarian/peritoneal |
| NCT06884345 | NCT06884345 | ClinicalTrials.gov | ClinicalTrials.gov | METR-NK Cells in Combination With Anti-angiogenic Neoadjuvant Therapy for Advanced Epithelial Ovarian Cancer | Anhui Provincial Cancer Hospital | China | Single gynecologic disease family specific | tubo-ovarian/peritoneal |
| NCT03814447 | NCT03814447 | ClinicalTrials.gov | ClinicalTrials.gov | The Fourth Generation CART-cell Therapy for Refractory-Relapsed Ovarian Cancer | Shanghai 6th People's Hospital | China | Single gynecologic disease family specific | tubo-ovarian/peritoneal |
| NCT02280811 | NCT02280811 | ClinicalTrials.gov | ClinicalTrials.gov | T Cell Receptor Immunotherapy Targeting HPV-16 E6 for HPV-Associated Cancers | National Cancer Institute (NCI) | United States | Broader basket including non-gynecologic cancers | cervical; vaginal |
| NCT00019084 | NCT00019084 | ClinicalTrials.gov | ClinicalTrials.gov | Vaccine Therapy and Biological Therapy in Treating Patients With Advanced Cancer | National Cancer Institute (NCI) | United States | Broader basket including non-gynecologic cancers | tubo-ovarian/peritoneal; cervical |
| NCT03412877 | NCT03412877 | ClinicalTrials.gov | ClinicalTrials.gov | Administration of Autologous T-Cells Genetically Engineered to Express T-Cell Receptors Reactive Against Neoantigens in People With Metastatic Cancer | National Cancer Institute (NCI) | United States | Broader basket including non-gynecologic cancers | tubo-ovarian/peritoneal |
| NCT02713984 | NCT02713984 | ClinicalTrials.gov | ClinicalTrials.gov | A Clinical Research of CAR T Cells Targeting HER2 Positive Cancer | Zhi Yang | China | Broader basket including non-gynecologic cancers | tubo-ovarian/peritoneal |
| NCT06284226 | NCT06284226 | ClinicalTrials.gov | ClinicalTrials.gov | An Exploratory Clinical Study to Evaluate the Safety and Efficacy of NK Cells (Combined With Standard Therapy) in the Treatment of Solid Tumor Patients | Beijing Kejing Biotechnology Co., Ltd. |  | Broader basket including non-gynecologic cancers | tubo-ovarian/peritoneal |
| NCT02457650 | NCT02457650 | ClinicalTrials.gov | ClinicalTrials.gov | T Cell Receptor-transduced T Cells Targeting NY-ESO-1 for Treatment of Patients With NY-ESO-1- Expressing Malignancies | Shenzhen Second People's Hospital | China | Broader basket including non-gynecologic cancers | tubo-ovarian/peritoneal |
| NCT06885697 | NCT06885697 | ClinicalTrials.gov | ClinicalTrials.gov | Anti-Mesothelin TNaive/SCM hYP218 (TNhYP218) CAR T Cells in Participants With Mesothelin-Expressing Solid Tumors Including Mesothelioma | National Cancer Institute (NCI) | United States | Broader basket including non-gynecologic cancers | tubo-ovarian/peritoneal |
| NCT05592626 | NCT05592626 | ClinicalTrials.gov | ClinicalTrials.gov | A Study of a Selective T Cell Receptor (TCR) Targeting, Bifunctional Antibody-fusion Molecule STAR0602 in Participants With Advanced Solid Tumors | Marengo Therapeutics, Inc. | United States; Canada; France; Spain | Broader basket including non-gynecologic cancers | vulvar |
| NCT02482090 | NCT02482090 | ClinicalTrials.gov | ClinicalTrials.gov | TIL Therapy for Metastatic Ovarian Cancer | Inge Marie Svane | Denmark | Broader basket including non-gynecologic cancers | tubo-ovarian/peritoneal |
| NCT06626256 | NCT06626256 | ClinicalTrials.gov | ClinicalTrials.gov | STIL101 for Injection for the Treatment of Locally Advanced, Metastatic or Unresectable Pancreatic Cancer, Colorectal Cancer, Renal Cell Cancer, Cervical Cancer and Melanoma | City of Hope Medical Center |  | Broader basket including non-gynecologic cancers | cervical |
| NCT05296564 | NCT05296564 | ClinicalTrials.gov | ClinicalTrials.gov | Anti-NY-ESO-1 TCR-Gene Engineered Lymphocytes Given by Infusion to Patients With NY-ESO-1 -Expressing Metastatic Cancers | Hadassah Medical Organization | Israel | Broader basket including non-gynecologic cancers | tubo-ovarian/peritoneal |
| NCT02153905 | NCT02153905 | ClinicalTrials.gov | ClinicalTrials.gov | T Cell Receptor Immunotherapy Targeting MAGE-A3 for Patients With Metastatic Cancer Who Are HLA-A*01 Positive | National Cancer Institute (NCI) | United States | Broader basket including non-gynecologic cancers | cervical |
| JPRN-jRCTc031200283 | JPRN-jRCTc031200283 | JPRN | WHO ICTRP | A Phase 2 Study Using Short-term Cultured Anti-tumor Autologous Lymphocytes Following a Nonmyeloablative Conditioning and Low-dose Interleukin-2 Treatment in Patients with Recurrent, Metastatic, or Persistent Cervical Cancer. - TILCC trial | Takashi Iwata | Japan | Single gynecologic disease family specific | cervical |
| ChiCTR1800019780 | ChiCTR1800019780 | ChiCTR | WHO ICTRP | Clinical study for CAR-T cell in the treatment of advanced ROR1+ malignancies | West China Hospital, Sichuan University | China | Broader basket including non-gynecologic cancers | tubo-ovarian/peritoneal |

# **S2A. Included trial records — Part 2 of 5: cell product, target, phase, and status**

**Note:** *One row represents one deduplicated trial record. The 211 records are repeated across five field groups and can be joined using Trial ID. Broader-basket eligibility does not imply that gynecologic participants were enrolled or that cohort-specific outcomes are available.*

| **TrialID** | **PublicregistryID** | **Cellmodality** | **Autologous/allogeneic** | **Cellproduct/descriptor** | **Target-reportingstate** | **Namedtarget** | **Phase** | **Registrystatus** |
| --- | --- | --- | --- | --- | --- | --- | --- | --- |
| NCT07644403 | NCT07644403 | CAR-T | Autologous | IMC-001, Suzhou Immunofoco Biotechnology | Not reported (registry visibility) |  | Phase I-II | Recruiting |
| NCT07534813 | NCT07534813 | TIL | Autologous | GT-307 | No predefined antigen (modality biology) |  | Phase I | Terminated |
| 695613 | EUCT 2024-517594-24-01 | Other ACT | Unclear | novoleucel | Unknown |  | Phase I-II | Not yet recruiting |
| NCT07420010 | NCT07420010 | CAR-T | Autologous | KT-032 | Named target | MSLN | Phase I | Recruiting |
| NCT07266298 | NCT07266298 | TCR-T | Autologous | NW-101C | Named target | PRAME | Phase I | Recruiting |
| NCT07231081 | NCT07231081 | CAR-T | Autologous | TX-103 | Named target | B7-H3 | Phase I | Recruiting |
| NCT07216105 | NCT07216105 | CAR-T | Allogeneic | FT-836 | Not reported (registry visibility) |  | Phase I | Recruiting |
| 668508 | ChiCTR2500109506 | NK | Unclear | IBR-854 recombinant human IL-21 oncolytic poxvirus, Hangzhou Converd | No predefined antigen (modality biology) |  | Phase I | Completed |
| NCT07181720 | NCT07181720 | CAR-T | Unclear | CD70 CAR-T therapy, Chongqing Precision Biotech (iv) | Named target | CD70 | Phase I | Recruiting |
| NCT07106814 | NCT07106814 | TIL | Unclear | tumour-infiltrating lymphocyte therapy, Leman Biotech | No predefined antigen (modality biology) |  | Phase I | Recruiting |
| NCT07101549 | NCT07101549 | CAR-T | Unclear | R-Star-001 | Not reported (registry visibility) |  | Phase I | Not yet recruiting |
| NCT07067255 | NCT07067255 | CAR-T | Unclear | LQ-102 | Not reported (registry visibility) |  | Phase I-II | Recruiting |
| NCT07030907 | NCT07030907 | CAR-T | Autologous | OPB-101 | Named target | MSLN | Phase I | Recruiting |
| NCT07010523 | NCT07010523 | CAR-T | Autologous | UCMYM-802 | Named target | MSLN | Phase I | Recruiting |
| NCT06951425 | NCT06951425 | CAR-T | Unclear | TH-027 | Named target | B7-H3 | Phase I | Not yet recruiting |
| NCT06912152 | NCT06912152 | CAR-T | Allogeneic | MT-027 | Named target | B7-H3 | Phase I | Recruiting |
| NCT06904131 | NCT06904131 | CAR-T | Unclear | Chimaeric antigen receptor therapy, unspecified | Named target | MSLN | Phase I | Recruiting |
| NCT06846424 | NCT06846424 | CAR-T | Autologous | SCT-001 | Not reported (registry visibility) |  | Phase I | Recruiting |
| NCT06834542 | NCT06834542 | TIL | Autologous | GT-201 | No predefined antigen (modality biology) |  | Phase I | Terminated |
| NCT06748872 | NCT06748872 | TCR-T | Autologous | MDG-1015 | Named target | NY-ESO-1 | Phase I | Recruiting |
| NCT06710288 | NCT06710288 | NK | Autologous | gemcitabine hydrochloride nogapendekin alfa inbakicept ceNK-cell therapy, NantKwest | Named target | FRα | Phase I | Recruiting |
| 545596 | EUCT 2023-504632-17-00 | TIL | Autologous | C-TIL052A, AbelZeta Pharma | No predefined antigen (modality biology) |  | Phase I | Not yet recruiting |
| NCT06697665 | NCT06697665 | TIL | Unclear | LM-103 | No predefined antigen (modality biology) |  | Phase I | Recruiting |
| NCT06658951 | NCT06658951 | CAR-T | Unclear | UCLE-802 | Named target | HER2 | Not applicable | Recruiting |
| NCT06646627 | NCT06646627 | CAR-T | Autologous | anti-B7-H3 CAR T cells | Named target | B7-H3 | Phase I | Recruiting |
| NCT06630611 | NCT06630611 | TIL | Autologous | cyclophosphamide fludarabine Tumour infiltrating lymphocyte (TIL)-adoptive cell therapy (ACT) interleukin-2, unspecified ANV-419 | No predefined antigen (modality biology) |  | Phase I | Recruiting |
| NCT06598371 | NCT06598371 | TIL | Autologous | aldesleukin KSQ-004 | No predefined antigen (modality biology) |  | Phase I-II | Recruiting |
| NCT06544720 | NCT06544720 | TCR-T | Autologous | SCG-142 | Not reported (registry visibility) |  | Phase I | Recruiting |
| NCT06509490 | NCT06509490 | CAR-T | Unclear | KD-025 cell injection | Not reported (registry visibility) |  | Phase I | Recruiting |
| NCT06505551 | NCT06505551 | TCR-T | Autologous | SCG-142 | Not reported (registry visibility) |  | Phase I-II | Recruiting |
| NCT06481592 | NCT06481592 | TIL | Unclear | lifileucel | No predefined antigen (modality biology) |  | Phase I | Recruiting |
| NCT06453057 | NCT06453057 | TIL | Autologous | GT-307 | No predefined antigen (modality biology) |  | Not applicable | Recruiting |
| 518933 | ChiCTR2000032124 | CAR-T | Unclear | IL-18BP, Yeda GM-CSF vaccine interleukin-1 beta interleukin-6 undisclosed - follicle-stimulating hormone chimaeric antigen receptor-T cell therapy, Hunan Siweikang Therapeutics SWK | Not reported (registry visibility) |  | Phase I | Recruiting |
| NCT06375187 | NCT06375187 | TIL | Unclear | GC-203 | No predefined antigen (modality biology) |  | Phase I | Recruiting |
| NCT06358053 | NCT06358053 | TCR-T | Unclear | HPV-16 E6 T-cell receptor therapy, Corregene Biotechnology | Named target | HPV E6/E7 | Phase I | Recruiting |
| 510939 |  | CAR-T | Unclear | anticancer therapy, Anixa Biosciences | Not reported (registry visibility) |  | Phase I | Recruiting |
| NCT06342336 | NCT06342336 | TIL | Autologous | HS-IT-101 | No predefined antigen (modality biology) |  | Phase I | Recruiting |
| NCT06327997 | NCT06327997 | CAR-T | Unclear | BZE-2203 | Named target | MSLN | Phase I | Recruiting |
| NCT06305299 | NCT06305299 | CAR-T | Unclear | cyclophosphamide fludarabine anti-B7-H3 CAR T cells | Named target | MUC16; B7-H3 | Phase I | Recruiting |
| NCT06256055 | NCT06256055 | CAR-T | Unclear | UCMYM-802 | Named target | MSLN | Phase I | Unknown |
| NCT06241781 | NCT06241781 | TIL | Autologous | GT-101 | No predefined antigen (modality biology) |  | Phase I | Recruiting |
| NCT06218914 | NCT06218914 | TCR-T | Autologous | AZD-0240 | Named target | Patient-specific antigen | Phase I | Recruiting |
| NCT06191900 | NCT06191900 | TIL | Autologous | GT-201 | No predefined antigen (modality biology) |  | Not applicable | Terminated |
| 496428 | CTR20233999 | TIL | Autologous | LM-103 | No predefined antigen (modality biology) |  | Phase I | Recruiting |
| NCT06145802 | NCT06145802 | TIL | Autologous | GT-316 | No predefined antigen (modality biology) |  | Phase I | Terminated |
| 489390 |  | TCR-T | Unclear | ADP-600 | Not reported (registry visibility) |  | Phase I | Not yet recruiting |
| NCT06051695 | NCT06051695 | CAR-T | Autologous | A2B-694 | Named target | MSLN | Phase I-II | Recruiting |
| NCT06010875 | NCT06010875 | CAR-T | Unclear | CD70 chimaeric antigen receptor cell therapy, Chongqing Precision Biotech | Named target | CD70 | Phase I | Recruiting |
| NCT05973487 | NCT05973487 | TCR-T | Autologous | TSC-200 TSC-201 TSC-202 TSC-203 TSC-204 | Named target | MAGE-A4; PRAME | Phase I | Recruiting |
| NCT05971576 | NCT05971576 | TIL | Autologous | LM-103 | No predefined antigen (modality biology) |  | Phase I | Not yet recruiting |
| NCT05952947 | NCT05952947 | TCR-T | Unclear | HRYZ-T101 | Not reported (registry visibility) |  | Phase I | Recruiting |
| NCT05941936 | NCT05941936 | TIL | Autologous | LM-103 | No predefined antigen (modality biology) |  | Phase I | Recruiting |
| NCT05902520 | NCT05902520 | TIL | Unclear | IL-2 iv gene therapy, Urigen AGX-148 | No predefined antigen (modality biology) |  | Phase I | Recruiting |
| NCT05877599 | NCT05877599 | TCR-T | Autologous | NT-175 | Named target | Patient-specific antigen | Phase I | Recruiting |
| NCT05848999 | NCT05848999 | CAR-T | Autologous | UCMYM-802 | Named target | MSLN | Phase I | Recruiting |
| NCT05787535 | NCT05787535 | TCR-T | Unclear | HRYZ-T101 | Not reported (registry visibility) |  | Phase I | Unknown |
| 463409 |  | CAR-T | Unclear | SNAP-chimaeric antigen receptor therapy cell therapy, Coeptis Therapeutics | Named target | HER2 | Phase I | Not yet recruiting |
| NCT05783089 | NCT05783089 | CAR-T | Autologous | UCMYM-802 | Named target | MSLN | Phase I | Not yet recruiting |
| NCT05775666 | NCT05775666 | CAR-T | Autologous | UCMYM-802 | Named target | MSLN | Phase I | Not yet recruiting |
| NCT05729399 | NCT05729399 | TIL | Autologous | GT-201 | No predefined antigen (modality biology) |  | Phase I-II | Unknown |
| 456726 |  | CAR-T | Unclear | bbT-4015 | Named target | MUC16 | Phase I | Not yet recruiting |
| NCT05672459 | NCT05672459 | CAR-T | Unclear | IVS-3001 | Not reported (registry visibility) |  | Phase I-II | Recruiting |
| NCT05881525 | NCT05881525 | TCR-T | Unclear | TC-N201 | Named target | NY-ESO-1 | Phase I | Recruiting |
| 443882 | ChiCTR2200064112 | CAR-T | Unclear | ICT-091 | Named target | MSLN | Phase I | Recruiting |
| NCT05568680 | NCT05568680 | CAR-T | Autologous | SynKIR-110 | Named target | MSLN | Phase I | Recruiting |
| NCT05539768 | NCT05539768 | TIL | Autologous | HS-IT-101 | No predefined antigen (modality biology) |  | Phase I | Not yet recruiting |
| 439506 | CTR20221207 | TIL | Autologous | GC-101, Shanghai Junshi Biosciences | No predefined antigen (modality biology) |  | Phase I | Recruiting |
| NCT05483491 | NCT05483491 | TCR-T | Unclear | 820-TCR program, T-Cure Bioscience | Not reported (registry visibility) |  | Phase I | Recruiting |
| NCT05475847 | NCT05475847 | TIL | Autologous | C-TIL052A, AbelZeta Pharma | No predefined antigen (modality biology) |  | Phase I | Unknown |
| NCT05472857 | NCT05472857 | CAR-T | Autologous | IMC-002, Suzhou Immunofoco Biotechnology | Not reported (registry visibility) |  | Phase I | Unknown |
| NCT05468307 | NCT05468307 | TIL | Autologous | GC-203 | No predefined antigen (modality biology) |  | Phase I | Completed |
| NCT05468190 | NCT05468190 | CAR-T | Unclear | CD70 chimaeric antigen receptor cell therapy, Chongqing Precision Biotech | Named target | CD70 | Phase I | Unknown |
| NCT05430373 | NCT05430373 | TIL | Autologous | GT-101 | No predefined antigen (modality biology) |  | Phase I | Completed |
| NCT05417750 | NCT05417750 | TIL | Autologous | nolgileucel | No predefined antigen (modality biology) |  | Phase I | Completed |
| NCT05397093 | NCT05397093 | TIL | Unclear | ITIL-306 | Named target | FRα | Phase I | Terminated |
| NCT05373147 | NCT05373147 | CAR-T | Unclear | BZDS-1901 | Named target | MSLN | Phase I | Unknown |
| NCT05366478 | NCT05366478 | TIL | Autologous | LM-103 | No predefined antigen (modality biology) |  | Phase I | Recruiting |
| 431389 |  | TCR-T | Unclear | TSC-201 | Not reported (registry visibility) |  | Phase I | Not yet recruiting |
| 430435 | CTR20260154 | TIL | Autologous | ZLT-001 | No predefined antigen (modality biology) |  | Phase I | Recruiting |
| NCT05342506 | NCT05342506 | TIL | Unclear | anti-cancer therapy, Beijing Carti Medical Technology | No predefined antigen (modality biology) |  | Phase I | Unknown |
| NCT05302037 | NCT05302037 | CAR-T | Allogeneic | CTM-N2D | Not reported (registry visibility) |  | Phase I | Recruiting |
| 427237 | ChiCTR2200057362 | CAR-T | Unclear | ICT-091 | Named target | MSLN | Phase I | Recruiting |
| NCT05238818 | NCT05238818 | TIL | Autologous | GT-202 | No predefined antigen (modality biology) |  | Phase I | Recruiting |
| NCT05211557 | NCT05211557 | CAR-T | Unclear | fhB7H3.CAR-T cells | Named target | MUC16; B7-H3 | Phase I-II | Recruiting |
| NCT05194735 | NCT05194735 | TCR-T | Autologous | T-cell receptor therapy, Ziopharm Oncology | Named target | Patient-specific antigen | Phase I-II | Terminated |
| 421887 | ChiCTR2100054914 | CAR-T | Unclear | ICT-091 | Named target | MSLN | Phase I | Recruiting |
| NCT05274451 | NCT05274451 | CAR-T | Autologous | LYL-797 | Not reported (registry visibility) |  | Phase I | Terminated |
| NCT05152797 | NCT05152797 | TIL | Autologous | NeoTIL-ACT | No predefined antigen (modality biology) |  | Phase I-II | Not yet recruiting |
| NCT05141253 | NCT05141253 | CAR-T | Unclear | RD-133 | Named target | MSLN | Phase I | Recruiting |
| NCT05357027 | NCT05357027 | TCR-T | Unclear | TC-E-202 | Not reported (registry visibility) |  | Phase I-II | Terminated |
| NCT05122221 | NCT05122221 | TCR-T | Unclear | HPV-16 E6 T-cell receptor therapy, Corregene Biotechnology | Named target | HPV E6/E7 | Phase I | Unknown |
| NCT05098171 | NCT05098171 | TIL | Autologous | nolgileucel | No predefined antigen (modality biology) |  | Phase I | Recruiting |
| NCT05089266 | NCT05089266 | CAR-T | Unclear | BZDS-1901 | Named target | MSLN | Phase I | Recruiting |
| NCT05499715 | NCT05499715 | TIL | Unclear | anti-cancer therapy, Beijing Carti Medical Technology | No predefined antigen (modality biology) |  | Phase I | Not yet recruiting |
| NCT04981691 | NCT04981691 | CAR-T | Autologous | UCMYM-802 | Named target | MSLN | Phase I | Completed |
| NCT05393635 | NCT05393635 | TIL | Unclear | ITIL-168 | No predefined antigen (modality biology) |  | Phase I | Withdrawn |
| 403252 |  | CAR-T | Unclear | CTH-004 | Not reported (registry visibility) |  | Phase I | Not yet recruiting |
| NCT04766320 | NCT04766320 | TIL | Autologous | nolgileucel | No predefined antigen (modality biology) |  | Phase I | Unknown |
| NCT05107739 | NCT05107739 | TIL | Autologous | DeTIL-0255 | No predefined antigen (modality biology) |  | Phase I | Terminated |
| 394743 | ChiCTR2100042320 | CAR-T | Unclear | mesothelin-targeted CAR-T cells | Named target | MSLN | Phase I | Recruiting |
| NCT04674488 | NCT04674488 | TIL | Unclear | Ori-TIL-102 | No predefined antigen (modality biology) |  | Phase I | Recruiting |
| NCT05239143 | NCT05239143 | CAR-T | Allogeneic | P-MUC1C-ALLO1 | Not reported (registry visibility) |  | Phase I | Recruiting |
| NCT04670068 | NCT04670068 | CAR-T | Autologous | B7H3-specific chimeric antigen receptor CAR T cells | Named target | MUC16; B7-H3 | Phase I | Recruiting |
| 389985 |  | CAR-T | Unclear | mesothelin chimaeric antigen receptor therapy, Hebei Senlang Biotechnology | Named target | MSLN | Phase I | Not yet recruiting |
| 389901 |  | CAR-T | Unclear | TmFRa-01 | Named target | FRα | Phase I | Terminated |
| NCT04627740 | NCT04627740 | CAR-T | Autologous | TC-A101 | Named target | MSLN | Phase I-II | Completed |
| NCT04611126 | NCT04611126 | TIL | Unclear | infiltrating lymphocytes nivolumab relatlimab ipilimumab (iv) | No predefined antigen (modality biology) |  | Phase I-II | Terminated |
| NCT04562298 | NCT04562298 | CAR-T | Unclear | LCAR-M23 CAR-T-cell therapy, Legend Biotech | Named target | MSLN | Phase I | Terminated |
| NCT04556669 | NCT04556669 | CAR-T | Autologous | SL-22P | Not reported (registry visibility) |  | Phase I | Unknown |
| NCT04483778 | NCT04483778 | CAR-T | Autologous | pembrolizumab B7H3-specific chimeric antigen receptor CAR T cells | Named target | B7-H3 | Phase I | Completed |
| NCT04476251 | NCT04476251 | TCR-T | Unclear | KITE-439 | Named target | HPV E6/E7 | Phase I | Terminated |
| NCT04443296 | NCT04443296 | TIL | Unclear | infiltrating lymphocytes | No predefined antigen (modality biology) |  | Phase I | Unknown |
| NCT04389229 | NCT04389229 | TIL | Autologous | ITIL-168 | No predefined antigen (modality biology) |  | Phase I-II | Withdrawn |
| 372638 |  | TCR-T | Unclear | 820-TCR program, T-Cure Bioscience | Not reported (registry visibility) |  | Phase I | Not yet recruiting |
| NCT05035407 | NCT05035407 | TCR-T | Unclear | 820-TCR program, T-Cure Bioscience | Not reported (registry visibility) |  | Phase I | Terminated |
| NCT04318964 | NCT04318964 | TCR-T | Unclear | TAEST-16001 | Named target | NY-ESO-1 | Phase I | Completed |
| NCT04503278 | NCT04503278 | CAR-T | Unclear | BNT-211 | Named target | MUC16 | Phase I | Recruiting |
| NCT04025216 | NCT04025216 | CAR-T | Autologous | TmTN-MUC-01 | Named target | MUC16; HER2 | Phase I | Terminated |
| NCT03992326 | NCT03992326 | TIL | Autologous | aldesleukin infiltrating lymphocytes | Named target | HER2 | Phase I | Terminated |
| NCT03970382 | NCT03970382 | Other ACT | Allogeneic | NeoTCR-P1 adoptive cell therapy, PACT Pharma | Named target | Patient-specific antigen | Phase I | Unknown |
| NCT03912831 | NCT03912831 | TCR-T | Unclear | KITE-439 | Named target | HPV E6/E7 | Phase I | Terminated |
| NCT03907527 | NCT03907527 | CAR-T | Autologous | PRGN-3005 | Named target | MUC16 | Phase I | Recruiting |
| NCT04650451 | NCT04650451 | CAR-T | Unclear | BPX-603 | Named target | HER2 | Phase I-II | Terminated |
| NCT03745326 | NCT03745326 | TCR-T | Unclear | anti-KRAS G12D mTCR therapy, Gilead | Not reported (registry visibility) |  | Phase I-II | Terminated |
| NCT03686124 | NCT03686124 | TCR-T | Autologous | anzutresgene autoleucel IMA-203CD8 | Named target | PRAME | Phase I-II | Recruiting |
| NCT03638206 | NCT03638206 | CAR-T | Autologous | CAR-T/TCR-T cell immunotherapy, Shenzhen BinDeBio | Named target | MSLN; NY-ESO-1 | Phase I-II | Unknown |
| NCT03610490 | NCT03610490 | TIL | Autologous | autologous tumour infiltrating lymphocytes MDA-TIL | No predefined antigen (modality biology) |  | Phase I | Terminated |
| NCT03578406 | NCT03578406 | TCR-T | Unclear | HPV E6-specific TCR-T cell therapy, Beijing TCRCure Biological | Named target | HPV E6/E7 | Phase I | Completed |
| 327082 | ChiCTR1800016805 | TIL | Unclear | neoVx + enhanced TIL + enhanced new antigen-reactive T cells, Chineo Med (Beijing) TILs + neoVX vaccine, Chineo Med new antigen vaccine (neoVx) + new antigen-reactive T cells, Chin | Named target | Patient-specific antigen | Phase I-II | Completed |
| 326892 | ChiCTR1800016764 | CAR-T | Autologous | mesothelin-targeted CAR-T cells | Named target | MSLN | Phase I | Completed |
| NCT03545815 | NCT03545815 | CAR-T | Unclear | GC-008t | Named target | MSLN | Phase I | Completed |
| NCT03449108 | NCT03449108 | TIL | Autologous | autologous tumour infiltrating lymphocytes, Iovance Biotherapeutics | Named target | HER2 | Phase I | Recruiting |
| NCT04102436 | NCT04102436 | TCR-T | Autologous | T-cell receptor therapy, Ziopharm Oncology | Named target | Patient-specific antigen | Phase I-II | Withdrawn |
| NCT03412526 | NCT03412526 | TIL | Autologous | aldesleukin infiltrating lymphocytes fludarabine | No predefined antigen (modality biology) |  | Phase I | Unknown |
| NCT03907852 | NCT03907852 | TCR-T | Unclear | gavocabtagene autoleucel | Named target | MSLN | Phase I-II | Completed |
| NCT03296137 | NCT03296137 | TIL | Autologous | infiltrating lymphocytes nivolumab ipilimumab | No predefined antigen (modality biology) |  | Phase I-II | Completed |
| NCT04072263 | NCT04072263 | TIL | Unclear | infiltrating lymphocytes interferon, Genentech (alpha2a) | Named target | MUC16 | Phase I | Unknown |
| NCT03287674 | NCT03287674 | TIL | Unclear | infiltrating lymphocytes nivolumab ipilimumab | No predefined antigen (modality biology) |  | Phase I-II | Completed |
| NCT03158935 | NCT03158935 | TIL | Unclear | aldesleukin infiltrating lymphocytes pembrolizumab | No predefined antigen (modality biology) |  | Phase I | Completed |
| NCT03159585 | NCT03159585 | TCR-T | Unclear | TAEST-16001 | Named target | NY-ESO-1 | Phase I | Completed |
| NCT03108495 | NCT03108495 | TIL | Autologous | autologous tumour infiltrating lymphocytes, Iovance Biotherapeutics | No predefined antigen (modality biology) |  | Phase I | Terminated |
| NCT03132922 | NCT03132922 | TCR-T | Autologous | afamitresgene autoleucel | Named target | MAGE-A4 | Phase I | Recruiting |
| NCT02930993 | NCT02930993 | CAR-T | Autologous | mesothelin-targeted CAR-T cells | Named target | MSLN | Phase I | Completed |
| NCT02858310 | NCT02858310 | TCR-T | Autologous | KITE-439 | Named target | HPV E6/E7 | Phase I-II | Completed |
| NCT02650986 | NCT02650986 | TCR-T | Autologous | infiltrating lymphocytes TGFb-DNR HBI 0201-ESO TCRT | Named target | NY-ESO-1 | Phase I-II | Recruiting |
| NCT02498912 | NCT02498912 | CAR-T | Autologous | JCAR-020 | Not reported (registry visibility) |  | Phase I | Completed |
| NCT02159716 | NCT02159716 | CAR-T | Autologous | chimaeric antigen receptor T-cell therapy, mesothelin, Novartis | Named target | MSLN | Phase I | Completed |
| NCT02111850 | NCT02111850 | TCR-T | Autologous | KITE-718 | Not reported (registry visibility) |  | Phase I-II | Completed |
| NCT01883297 | NCT01883297 | TIL | Autologous | cyclophosphamide aldesleukin infiltrating lymphocytes | No predefined antigen (modality biology) |  | Phase I | Recruiting |
| NCT01585428 | NCT01585428 | TIL | Autologous | aldesleukin infiltrating lymphocytes | No predefined antigen (modality biology) |  | Phase I | Completed |
| NCT01567891 | NCT01567891 | TCR-T | Autologous | letetresgene-autoleucel | Named target | NY-ESO-1 | Phase I-II | Completed |
| 154752 |  | TIL | Unclear | lifileucel | No predefined antigen (modality biology) |  | Phase I | Terminated |
| NCT01174121 | NCT01174121 | TIL | Autologous | aldesleukin infiltrating lymphocytes pembrolizumab | Named target | Patient-specific antigen | Phase I | Recruiting |
| 1772 |  | TIL | Autologous | cisplatin aldesleukin paclitaxel infiltrating lymphocytes | No predefined antigen (modality biology) |  | Phase I | Completed |
| NCT05686226 | NCT05686226 | TCR-T | Unclear | E7 TCR-T cells | Named target | HPV E6/E7 | Phase II | Recruiting |
| NCT05639972 | NCT05639972 | TCR-T | Unclear | E7 TCR-T cells | Named target | HPV E6/E7 | Phase I-II | Recruiting |
| NCT07617753 | NCT07617753 | CAR-NK | Unclear | Dual-target CAR-NK cell product | Named target | MSLN; MUC16; FRα | Phase I-II | Recruiting |
| NCT07589543 | NCT07589543 | CAR-NK | Unclear | EB-DUALNK | Named target | MSLN | Phase I-II | Recruiting |
| NCT07410494 | NCT07410494 | CAR-NK | Allogeneic | EB-SELECT Single-Target CAR-NK Cells | Not reported (registry visibility) |  | Phase I-II | Recruiting |
| NCT07480954 | NCT07480954 | CAR-NK | Unclear | Dual-target CAR-NK cell product | Named target | MSLN; MUC16; FRα | Phase I-II | Recruiting |
| NCT05776355 | NCT05776355 | CAR-NK | Unclear | NKG2D CAR-NK | Not reported (registry visibility) |  | Not applicable | Unknown |
| NCT05410717 | NCT05410717 | CAR-NK | Unclear | Claudin6, GPC3, Mesothelin, or AXL targeting CAR-NK cells | Named target | MSLN | Phase I | Recruiting |
| NCT06215950 | NCT06215950 | CAR-T | Unclear | CD70 CAR-T cells | Named target | CD70 | Phase I | Recruiting |
| NCT03692637 | NCT03692637 | CAR-NK | Unclear | anti-Mesothelin Car NK Cells | Named target | MSLN | Phase I | Unknown |
| NCT05225363 | NCT05225363 | CAR-T | Unclear | Chimeric Antigen Receptor T-cells | Not reported (registry visibility) |  | Phase I | Recruiting |
| NCT07435701 | NCT07435701 | NK | Unclear | SK-NK Cell Injection | No predefined antigen (modality biology) |  | Phase I-II | Recruiting |
| NCT05922930 | NCT05922930 | CAR-NK | Unclear | TROP2-CAR-NK | Not reported (registry visibility) |  | Phase I-II | Recruiting |
| NCT05856643 | NCT05856643 | CAR-NK | Unclear | SZ011 CAR-NK | Not reported (registry visibility) |  | Phase I | Recruiting |
| NCT07410676 | NCT07410676 | NK | Allogeneic | EBNK-001 + IL-15 + Pembrolizumab | No predefined antigen (modality biology) |  | Phase I-II | Recruiting |
| NCT05963100 | NCT05963100 | CAR-T | Unclear | TCR-like CAR-T | Named target | MSLN | Phase I-II | Recruiting |
| NCT02118285 | NCT02118285 | NK | Unclear | Fludarabine | No predefined antigen (modality biology) |  | Phase I | Completed |
| NCT03691376 | NCT03691376 | TCR-T | Autologous | Aldesleukin | Named target | NY-ESO-1 | Phase I | Terminated |
| NCT01583686 | NCT01583686 | CAR-T | Unclear | Fludarabine | Named target | MSLN | Phase I-II | Terminated |
| NCT03213964 | NCT03213964 | NK | Unclear | FATE-NK100 | No predefined antigen (modality biology) |  | Phase I | Completed |
| NCT01105650 | NCT01105650 | NK | Allogeneic | Fludarabine | No predefined antigen (modality biology) |  | Phase II | Completed |
| NCT00652899 | NCT00652899 | NK | Allogeneic | Allopurinol | No predefined antigen (modality biology) |  | Phase II | Terminated |
| NCT06321484 | NCT06321484 | NK | Unclear | Cytokine-Induced Memory-like Natural Killer Cells | No predefined antigen (modality biology) |  | Phase I | Recruiting |
| NCT07651124 | NCT07651124 | TIL | Autologous | OTHER: autologous tumor-infiltrating lymphocytes | No predefined antigen (modality biology) |  | Not applicable | Recruiting |
| NCT05518253 | NCT05518253 | CAR-T | Unclear | CD70 CAR-T cells | Named target | CD70 | Phase I | Recruiting |
| NCT05420545 | NCT05420545 | CAR-T | Unclear | CD70 CAR-T cells | Named target | CD70 | Phase I | Unknown |
| NCT03539406 | NCT03539406 | NK | Allogeneic | UCB-NK cells | No predefined antigen (modality biology) |  | Phase I | Completed |
| NCT07613723 | NCT07613723 | NK | Unclear | ZI-MA4-1 (TCR-NK cells) | No predefined antigen (modality biology) |  | Phase I | Recruiting |
| NCT03356795 | NCT03356795 | CAR-T | Unclear | Cervical cancer-specific CAR-T cells | Named target | MSLN | Phase I-II | Recruiting |
| NCT03585764 | NCT03585764 | CAR-T | Unclear | MOv19-BBz CAR T cells | Not reported (registry visibility) |  | Phase I | Terminated |
| NCT02580747 | NCT02580747 | CAR-T | Unclear | anti-meso-CAR vector transduced T cells | Not reported (registry visibility) |  | Phase I | Unknown |
| NCT07096583 | NCT07096583 | NK | Unclear | METR-NK cell（metabolic remodeling nature killer cells） | No predefined antigen (modality biology) |  | Phase I-II | Recruiting |
| NCT03634501 | NCT03634501 | NK | Unclear | Activated NK | No predefined antigen (modality biology) |  | Phase I-II | Unknown |
| NCT05372692 | NCT05372692 | CAR-T | Unclear | mesothelin-specific chimeric antigen receptor T cell injection | Named target | MSLN | Not applicable | Completed |
| NCT03916679 | NCT03916679 | CAR-T | Unclear | anti-MESO CAR-T cells | Not reported (registry visibility) |  | Phase I-II | Unknown |
| NCT03735589 | NCT03735589 | NK | Autologous | Alpha-type-1 Polarized Dendritic Cells | No predefined antigen (modality biology) |  | Phase I-II | Withdrawn |
| NCT06469281 | NCT06469281 | Other ACT | Unclear | OTHER: 27T51 | Named target | MUC16 | Phase I | Recruiting |
| NCT06395844 | NCT06395844 | NK | Unclear | METR-NK cell(Metabolic Remodeling Nature Killer Cells) | No predefined antigen (modality biology) |  | Phase I-II | Recruiting |
| NCT00101257 | NCT00101257 | Other ACT | Autologous | therapeutic autologous lymphocytes | Unknown |  | Phase I | Completed |
| NCT03799913 | NCT03799913 | CAR-T | Unclear | anti-MESO CAR-T cells | Not reported (registry visibility) |  | Phase I | Unknown |
| NCT03054298 | NCT03054298 | CAR-T | Unclear | huCART-meso cells | Named target | MSLN | Phase I | Completed |
| NCT06884345 | NCT06884345 | NK | Unclear | METR-NK cells combined with Solvatinib | No predefined antigen (modality biology) |  | Phase I-II | Recruiting |
| NCT03814447 | NCT03814447 | CAR-T | Unclear | anti- MESO CAR-T cells | Named target | MSLN | Phase I | Unknown |
| NCT02280811 | NCT02280811 | TCR-T | Unclear | Fludarabine | Named target | HPV E6/E7 | Phase I-II | Completed |
| NCT00019084 | NCT00019084 | TIL | Autologous | aldesleukin | Named target | Patient-specific antigen | Phase II | Completed |
| NCT03412877 | NCT03412877 | TCR-T | Autologous | Cyclophosphamide | Named target | Patient-specific antigen | Phase II | Recruiting |
| NCT02713984 | NCT02713984 | CAR-T | Unclear | Anti-HER2 CAR-T | Named target | HER2 | Phase I-II | Withdrawn |
| NCT06284226 | NCT06284226 | NK | Allogeneic | Allogeneic NK（CCT-ANK-11) | No predefined antigen (modality biology) |  | Phase I | Withdrawn |
| NCT02457650 | NCT02457650 | TCR-T | Unclear | Cyclophosphamide | Named target | NY-ESO-1 | Phase I | Unknown |
| NCT06885697 | NCT06885697 | CAR-T | Unclear | mesothelin expression testing | Named target | MSLN | Phase I | Recruiting |
| NCT05592626 | NCT05592626 | TCR-T | Unclear | STAR0602 | Not reported (registry visibility) |  | Phase I-II | Recruiting |
| NCT02482090 | NCT02482090 | TIL | Unclear | Cyclophosphamide | No predefined antigen (modality biology) |  | Phase I | Completed |
| NCT06626256 | NCT06626256 | TIL | Unclear | Aldesleukin | No predefined antigen (modality biology) |  | Phase I | Withdrawn |
| NCT05296564 | NCT05296564 | TCR-T | Unclear | CYCLOPHOSPHAMIDE and FLUDARABIN | Named target | NY-ESO-1 | Phase I-II | Recruiting |
| NCT02153905 | NCT02153905 | TCR-T | Unclear | Aldesleukin | Not reported (registry visibility) |  | Phase I-II | Terminated |
| JPRN-jRCTc031200283 | JPRN-jRCTc031200283 | TIL | Autologous | Autologous tumor-infiltrating lymphocytes | No predefined antigen (modality biology) |  | Phase II | Recruiting |
| ChiCTR1800019780 | ChiCTR1800019780 | CAR-T | Unclear | ROR1 CAR-T | Named target | ROR1 | Phase I | Recruiting |

# **S2A. Included trial records — Part 3 of 5: treatment context and trial design**

**Note:** *One row represents one deduplicated trial record. The 211 records are repeated across five field groups and can be joined using Trial ID. Broader-basket eligibility does not imply that gynecologic participants were enrolled or that cohort-specific outcomes are available.*

| **Trial ID** | **Public registry ID** | **Centre type** | **Planned sample size** | **Start date** | **Lymphodepletion** | **IL-2 support** | **ICI combination** | **PARP-inhibitor combination** | **Antiangiogenic combination** | **Delivery route** |
| --- | --- | --- | --- | --- | --- | --- | --- | --- | --- | --- |
| NCT07644403 | NCT07644403 | Single-center | 40.0 | 2026-03-05 | No | No | No | No | No | IV |
| NCT07534813 | NCT07534813 | Single-center |  | 2026-04-30 | Yes | Yes | No | No | Yes | IV |
| 695613 | EUCT 2024-517594-24-01 | Single-center | 20.0 | 2026-01-31 | No | No | No | No | No | Unclear |
| NCT07420010 | NCT07420010 | Single-center | 24.0 | 2026-03-15 | Yes | No | No | No | No | IP |
| NCT07266298 | NCT07266298 | Single-center | 24.0 | 2025-12-17 | Yes | No | No | No | Yes | IV |
| NCT07231081 | NCT07231081 | Single-center | 162.0 | 2025-07-29 | Yes | No | No | No | No | IP |
| NCT07216105 | NCT07216105 | Single-center | 113.0 | 2025-11-04 | No | No | No | No | No | IV |
| 668508 | ChiCTR2500109506 | Single-center | 5.0 | 2024-05-16 | No | Yes | No | No | No | Intratumoral |
| NCT07181720 | NCT07181720 | Single-center | 90.0 | 2025-09-12 | Yes | No | No | Yes | Yes | IP |
| NCT07106814 | NCT07106814 | Single-center | 36.0 | 2025-08-29 | Yes | Yes | No | No | No | IV |
| NCT07101549 | NCT07101549 | Single-center | 25.0 | 2025-08-01 | No | No | No | No | No | IV |
| NCT07067255 | NCT07067255 | Single-center | 80.0 | 2025-04-29 | Yes | No | Yes | No | No | IV |
| NCT07030907 | NCT07030907 | Multicenter single-country | 30.0 | 2025-06-16 | No | Yes | No | Yes | No | IV |
| NCT07010523 | NCT07010523 | Single-center | 24.0 | 2024-10-15 | Yes | No | No | No | No | IV |
| NCT06951425 | NCT06951425 | Single-center | 24.0 | 2025-06-01 | No | No | No | No | No | IP |
| NCT06912152 | NCT06912152 | Single-center | 12.0 | 2025-05-12 | Yes | No | No | No | No | IP |
| NCT06904131 | NCT06904131 | Single-center | 20.0 | 2025-04-01 | Yes | No | Yes | No | No | IV |
| NCT06846424 | NCT06846424 | Single-center | 24.0 | 2025-06-01 | Yes | No | No | No | No | IP |
| NCT06834542 | NCT06834542 | Single-center | 1.0 | 2022-11-01 | Yes | Yes | No | No | No | IV |
| NCT06748872 | NCT06748872 | Multinational | 55.0 | 2025-07-01 | Yes | No | Yes | No | No | IV |
| NCT06710288 | NCT06710288 | Multicenter single-country | 20.0 | 2024-11-06 | No | No | No | Yes | Yes | IV |
| 545596 | EUCT 2023-504632-17-00 | Single-center | 11.0 | 2023-06-01 | Yes | Yes | Yes | No | No | IV |
| NCT06697665 | NCT06697665 | Single-center | 10.0 | 2024-01-23 | Yes | Yes | Yes | No | No | IV |
| NCT06658951 | NCT06658951 | Single-center | 30.0 | 2025-04-03 | Yes | No | No | No | No | IV |
| NCT06646627 | NCT06646627 | Single-center | 48.0 | 2024-11-11 | Yes | No | No | No | No | IP |
| NCT06630611 | NCT06630611 | Multinational | 40.0 | 2025-01-15 | Yes | Yes | No | No | Yes | IV |
| NCT06598371 | NCT06598371 | Single-center | 141.0 | 2024-11-21 | Yes | Yes | Yes | No | No | IV |
| NCT06544720 | NCT06544720 | Single-center | 18.0 | 2023-08-02 | Yes | No | No | No | No | IV |
| NCT06509490 | NCT06509490 | Single-center | 9.0 | 2024-07-17 | Yes | No | No | No | No | IV |
| NCT06505551 | NCT06505551 | Multinational | 66.0 | 2024-10-01 | Yes | No | No | No | No | IV |
| NCT06481592 | NCT06481592 | Multicenter single-country | 60.0 | 2024-10-29 | Yes | Yes | Yes | No | No | IV |
| NCT06453057 | NCT06453057 | Single-center | 18.0 | 2024-07-04 | Yes | No | No | No | No | IV |
| 518933 | ChiCTR2000032124 | Single-center | 60.0 | 2019-01-01 | Yes | No | No | No | No | IV |
| NCT06375187 | NCT06375187 | Single-center | 18.0 | 2024-05-29 | Yes | No | Yes | No | No | IV |
| NCT06358053 | NCT06358053 | Single-center | 24.0 | 2024-04-15 | Yes | Yes | No | No | Yes | IV |
| 510939 |  | Single-center |  |  | No | No | No | No | No | Unclear |
| NCT06342336 | NCT06342336 | Single-center | 44.0 | 2024-01-18 | Yes | Yes | No | No | No | IV |
| NCT06327997 | NCT06327997 | Single-center | 20.0 | 2024-03-07 | No | No | Yes | No | No | IV |
| NCT06305299 | NCT06305299 | Single-center | 27.0 | 2024-07-29 | Yes | No | No | No | No | IP |
| NCT06256055 | NCT06256055 | Single-center | 24.0 | 2024-02-20 | Yes | No | No | No | No | IV |
| NCT06241781 | NCT06241781 | Multicenter single-country | 83.0 | 2024-04-02 | No | No | No | No | No | IV |
| NCT06218914 | NCT06218914 | Multicenter single-country | 108.0 | 2024-03-22 | No | No | No | No | No | IV |
| NCT06191900 | NCT06191900 | Single-center | 4.0 | 2023-06-05 | Yes | Yes | No | No | No | IV |
| 496428 | CTR20233999 | Single-center | 15.0 | 2023-12-28 | Yes | Yes | No | No | No | IV |
| NCT06145802 | NCT06145802 | Single-center |  | 2023-03-09 | Yes | Yes | No | No | No | IV |
| 489390 |  | Single-center |  | 2023-12-01 | No | No | No | No | No | Unclear |
| NCT06051695 | NCT06051695 | Multicenter single-country | 474.0 | 2024-04-03 | Yes | No | Yes | No | No | IV |
| NCT06010875 | NCT06010875 | Single-center | 48.0 | 2023-11-08 | Yes | No | No | Yes | No | IP |
| NCT05973487 | NCT05973487 | Multicenter single-country | 840.0 | 2024-05-06 | Yes | No | No | No | No | IV |
| NCT05971576 | NCT05971576 | Single-center | 20.0 | 2023-08-01 | Yes | Yes | No | No | No | IV |
| NCT05952947 | NCT05952947 | Single-center | 32.0 | 2023-11-01 | Yes | No | No | No | No | IV |
| NCT05941936 | NCT05941936 | Single-center | 5.0 | 2023-05-19 | Yes | Yes | No | No | No | IV |
| NCT05902520 | NCT05902520 | Single-center | 18.0 | 2023-06-19 | Yes | Yes | Yes | No | No | IV |
| NCT05877599 | NCT05877599 | Multicenter single-country | 45.0 | 2023-07-12 | Yes | Yes | No | No | No | IV |
| NCT05848999 | NCT05848999 | Single-center | 87.0 | 2023-04-27 | Yes | No | No | No | No | IV |
| NCT05787535 | NCT05787535 | Single-center | 17.0 | 2023-03-21 | Yes | No | No | No | No | IV |
| 463409 |  | Single-center |  |  | No | No | No | No | No | Unclear |
| NCT05783089 | NCT05783089 | Single-center | 87.0 | 2023-04-01 | Yes | No | No | No | No | IV |
| NCT05775666 | NCT05775666 | Single-center | 87.0 | 2023-03-01 | Yes | No | No | No | No | IV |
| NCT05729399 | NCT05729399 | Multicenter single-country | 6.0 | 2022-09-14 | Yes | Yes | No | No | No | IV |
| 456726 |  | Single-center |  |  | No | No | No | No | No | Unclear |
| NCT05672459 | NCT05672459 | Single-center | 31.0 | 2023-06-21 | Yes | No | Yes | Yes | No | IV |
| NCT05881525 | NCT05881525 | Single-center | 18.0 | 2023-06-01 | Yes | Yes | No | No | No | IV |
| 443882 | ChiCTR2200064112 | Single-center | 15.0 | 2022-09-13 | Yes | No | No | No | No | IV |
| NCT05568680 | NCT05568680 | Multicenter single-country | 42.0 | 2023-03-30 | Yes | No | No | No | No | IV |
| NCT05539768 | NCT05539768 | Single-center | 8.0 | 2022-10-08 | Yes | Yes | Yes | No | No | IV |
| 439506 | CTR20221207 | Single-center | 60.0 | 2022-10-12 | No | No | No | No | No | IV |
| NCT05483491 | NCT05483491 | Multicenter single-country | 42.0 | 2022-09-26 | Yes | Yes | No | No | No | IV |
| NCT05475847 | NCT05475847 | Multicenter single-country | 20.0 | 2022-07-01 | Yes | Yes | No | No | No | IV |
| NCT05472857 | NCT05472857 | Single-center | 30.0 | 2022-08-08 | Yes | No | No | No | No | IV |
| NCT05468307 | NCT05468307 | Single-center | 26.0 | 2022-03-10 | Yes | No | Yes | No | No | IV |
| NCT05468190 | NCT05468190 | Single-center | 48.0 | 2022-07-17 | Yes | No | No | Yes | No | IP |
| NCT05430373 | NCT05430373 | Multicenter single-country | 31.0 | 2022-05-23 | Yes | Yes | No | No | No | IV |
| NCT05417750 | NCT05417750 | Single-center | 43.0 | 2022-10-12 | Yes | Yes | Yes | No | No | IV |
| NCT05397093 | NCT05397093 | Multicenter single-country | 1.0 | 2022-08-24 | Yes | Yes | Yes | Yes | Yes | IV |
| NCT05373147 | NCT05373147 | Single-center | 21.0 | 2020-10-30 | No | No | Yes | No | No | IV |
| NCT05366478 | NCT05366478 | Single-center | 15.0 | 2022-05-30 | Yes | Yes | No | No | No | IV |
| 431389 |  | Single-center |  | 2023-12-01 | No | No | No | No | No | Unclear |
| 430435 | CTR20260154 | Single-center | 33.0 | 2026-01-06 | Yes | Yes | No | No | No | IV |
| NCT05342506 | NCT05342506 | Single-center | 30.0 | 2022-04-18 | No | Yes | Yes | No | No | IV |
| NCT05302037 | NCT05302037 | Single-center | 12.0 | 2024-11-19 | Yes | Yes | No | No | No | IV |
| 427237 | ChiCTR2200057362 | Single-center | 10.0 | 2022-03-09 | Yes | No | No | No | No | IV |
| NCT05238818 | NCT05238818 | Single-center | 36.0 | 2022-02-14 | Yes | Yes | Yes | No | No | IV |
| NCT05211557 | NCT05211557 | Single-center | 15.0 | 2021-11-16 | Yes | No | No | No | No | IP |
| NCT05194735 | NCT05194735 | Single-center | 8.0 | 2022-01-26 | Yes | Yes | No | No | No | IV |
| 421887 | ChiCTR2100054914 | Single-center | 15.0 | 2021-12-29 | Yes | No | No | No | No | IV |
| NCT05274451 | NCT05274451 | Multicenter single-country | 57.0 | 2022-03-29 | Yes | No | Yes | No | No | IV |
| NCT05152797 | NCT05152797 | Single-center | 91.0 | 2022-01-01 | Yes | Yes | No | No | No | IV |
| NCT05141253 | NCT05141253 | Single-center | 24.0 | 2022-01-12 | Yes | No | No | Yes | No | IV |
| NCT05357027 | NCT05357027 | Single-center | 5.0 | 2022-08-10 | Yes | Yes | No | No | No | IV |
| NCT05122221 | NCT05122221 | Single-center | 12.0 | 2022-06-28 | Yes | Yes | No | No | No | IV |
| NCT05098171 | NCT05098171 | Single-center | 50.0 | 2021-09-26 | Yes | No | Yes | No | No | IV |
| NCT05089266 | NCT05089266 | Single-center | 30.0 | 2021-11-30 | No | No | Yes | No | No | IV |
| NCT05499715 | NCT05499715 | Single-center | 48.0 | 2021-08-31 | No | Yes | Yes | No | No | IV |
| NCT04981691 | NCT04981691 | Single-center | 12.0 | 2021-07-29 | Yes | No | No | No | No | IV |
| NCT05393635 | NCT05393635 | Single-center | 0.0 | 2022-08-01 | Yes | Yes | Yes | No | No | IV |
| 403252 |  | Single-center |  | 2023-12-01 | No | No | No | No | No | Unclear |
| NCT04766320 | NCT04766320 | Single-center | 50.0 | 2021-01-04 | Yes | No | Yes | No | No | IV |
| NCT05107739 | NCT05107739 | Multicenter single-country | 54.0 | 2021-12-22 | Yes | Yes | No | No | No | IV |
| 394743 | ChiCTR2100042320 | Single-center | 9.0 | 2021-02-01 | No | No | No | No | No | IV |
| NCT04674488 | NCT04674488 | Single-center | 15.0 | 2020-12-09 | Yes | Yes | No | No | No | IV |
| NCT05239143 | NCT05239143 | Multicenter single-country | 180.0 | 2022-02-15 | Yes | No | No | No | No | IV |
| NCT04670068 | NCT04670068 | Single-center | 4.0 | 2021-01-27 | Yes | No | No | Yes | No | IP |
| 389985 |  | Single-center |  |  | No | No | No | No | No | Unclear |
| 389901 |  | Single-center |  |  | No | No | No | No | No | Unclear |
| NCT04627740 | NCT04627740 | Single-center | 5.0 | 2020-12-01 | Yes | No | No | No | No | IV |
| NCT04611126 | NCT04611126 | Single-center | 6.0 | 2021-04-22 | Yes | Yes | Yes | No | No | IV |
| NCT04562298 | NCT04562298 | Single-center | 15.0 | 2020-10-21 | Yes | No | No | No | No | IV |
| NCT04556669 | NCT04556669 | Single-center | 30.0 | 2020-08-01 | No | No | Yes | No | No | IV |
| NCT04483778 | NCT04483778 | Single-center | 68.0 | 2020-07-13 | Yes | No | Yes | No | No | IV |
| NCT04476251 | NCT04476251 | Single-center | 1.0 | 2021-01-14 | No | Yes | No | No | No | IV |
| NCT04443296 | NCT04443296 | Single-center | 120.0 | 2019-10-12 | No | No | No | No | No | IV |
| NCT04389229 | NCT04389229 | Multicenter single-country | 22.0 | 2020-07-01 | Yes | Yes | No | No | No | IV |
| 372638 |  | Single-center |  | 2022-12-01 | No | No | No | No | No | Unclear |
| NCT05035407 | NCT05035407 | Single-center | 33.0 | 2022-03-08 | Yes | Yes | No | No | No | IV |
| NCT04318964 | NCT04318964 | Single-center | 12.0 | 2020-03-19 | Yes | Yes | No | No | No | IV |
| NCT04503278 | NCT04503278 | Multinational | 214.0 | 2020-09-16 | Yes | No | No | No | No | IV |
| NCT04025216 | NCT04025216 | Multicenter single-country | 16.0 | 2019-10-10 | Yes | No | Yes | No | No | IV |
| NCT03992326 | NCT03992326 | Single-center | 6.0 | 2019-09-01 | Yes | Yes | Yes | No | Yes | IV |
| NCT03970382 | NCT03970382 | Multicenter single-country | 28.0 | 2019-07-03 | Yes | Yes | Yes | No | No | IV |
| NCT03912831 | NCT03912831 | Multicenter single-country | 8.0 | 2019-04-30 | Yes | Yes | No | No | No | IV |
| NCT03907527 | NCT03907527 | Multicenter single-country | 71.0 | 2019-04-30 | Yes | No | No | Yes | No | IP |
| NCT04650451 | NCT04650451 | Multicenter single-country | 220.0 | 2020-12-07 | No | No | No | No | No | IV |
| NCT03745326 | NCT03745326 | Single-center | 5.0 | 2019-05-16 | Yes | Yes | Yes | No | Yes | IV |
| NCT03686124 | NCT03686124 | Multinational | 375.0 | 2019-04-09 | Yes | Yes | Yes | No | No | IV |
| NCT03638206 | NCT03638206 | Single-center | 73.0 | 2018-03-01 | Yes | No | No | No | No | IV |
| NCT03610490 | NCT03610490 | Single-center | 27.0 | 2018-10-16 | Yes | Yes | No | No | No | IV |
| NCT03578406 | NCT03578406 | Single-center | 9.0 | 2018-09-01 | No | No | Yes | No | No | IV |
| 327082 | ChiCTR1800016805 | Single-center | 400.0 | 2018-06-26 | No | No | Yes | No | No | Unclear |
| 326892 | ChiCTR1800016764 | Single-center | 20.0 | 2018-07-01 | No | No | No | No | No | IV |
| NCT03545815 | NCT03545815 | Multicenter single-country | 10.0 | 2018-03-19 | Yes | No | Yes | No | No | IV |
| NCT03449108 | NCT03449108 | Single-center | 30.0 |  | Yes | Yes | Yes | No | No | IV |
| NCT04102436 | NCT04102436 | Single-center | 210.0 | 2024-03-08 | Yes | Yes | No | No | Yes | Intratumoral |
| NCT03412526 | NCT03412526 | Single-center | 15.0 | 2018-01-21 | Yes | Yes | No | No | No | IV |
| NCT03907852 | NCT03907852 | Multinational | 57.0 | 2019-04-15 | Yes | No | Yes | No | No | IV |
| NCT03296137 | NCT03296137 | Single-center | 25.0 | 2017-10-13 | Yes | No | Yes | No | No | IV |
| NCT04072263 | NCT04072263 | Single-center | 24.0 | 2018-08-01 | Yes | Yes | Yes | No | No | IV |
| NCT03287674 | NCT03287674 | Single-center | 7.0 | 2017-10-09 | Yes | Yes | Yes | No | No | IV |
| NCT03158935 | NCT03158935 | Single-center | 8.0 | 2017-07-07 | Yes | Yes | Yes | No | No | IV |
| NCT03159585 | NCT03159585 | Single-center | 6.0 | 2017-04-14 | Yes | Yes | No | No | No | IV |
| NCT03108495 | NCT03108495 | Multinational | 210.0 | 2017-06-22 | Yes | Yes | Yes | No | Yes | IV |
| NCT03132922 | NCT03132922 | Multinational | 71.0 | 2017-05-15 | Yes | No | Yes | No | No | IV |
| NCT02930993 | NCT02930993 | Single-center | 20.0 | 2016-08-01 | Yes | No | No | No | No | IV |
| NCT02858310 | NCT02858310 | Multicenter single-country | 224.0 | 2017-01-27 | Yes | Yes | Yes | No | No | IV |
| NCT02650986 | NCT02650986 | Single-center | 15.0 | 2017-07-14 | Yes | No | Yes | Yes | Yes | IV |
| NCT02498912 | NCT02498912 | Single-center | 18.0 | 2015-08-01 | Yes | No | Yes | No | No | IP |
| NCT02159716 | NCT02159716 | Single-center | 19.0 | 2014-06-01 | Yes | No | No | No | No | IV |
| NCT02111850 | NCT02111850 | Multinational | 21.0 | 2014-02-07 | Yes | Yes | Yes | No | No | IV |
| NCT01883297 | NCT01883297 | Single-center | 3.0 | 2015-01-01 | Yes | Yes | No | No | No | IV |
| NCT01585428 | NCT01585428 | Single-center | 29.0 | 2012-04-13 | Yes | Yes | No | No | No | IV |
| NCT01567891 | NCT01567891 | Multicenter single-country | 9.0 | 2013-07-09 | Yes | Yes | No | No | No | IV |
| 154752 |  | Single-center |  | 2014-06-01 | No | No | Yes | No | No | Unclear |
| NCT01174121 | NCT01174121 | Single-center | 332.0 | 2010-08-26 | Yes | Yes | Yes | No | No | IV |
| 1772 |  | Single-center | 62.0 |  | No | Yes | No | No | No | IP |
| NCT05686226 | NCT05686226 | Multicenter single-country | 20.0 | 2023-03-07 | Yes | Yes | No | No | No | Unclear |
| NCT05639972 | NCT05639972 | Multicenter single-country | 15.0 | 2025-08-11 | Yes | Yes | No | No | No | IV |
| NCT07617753 | NCT07617753 | Single-center | 36.0 | 2026-03-02 | Yes | No | No | No | No | Unclear |
| NCT07589543 | NCT07589543 | Single-center | 42.0 | 2026-03-02 | Yes | No | No | No | No | IV |
| NCT07410494 | NCT07410494 | Single-center | 85.0 | 2026-02-01 | No | No | No | No | No | Unclear |
| NCT07480954 | NCT07480954 | Single-center | 36.0 | 2026-02-04 | Yes | No | No | No | No | Unclear |
| NCT05776355 | NCT05776355 | Single-center | 18.0 | 2023-03-01 | No | No | No | No | No | Unclear |
| NCT05410717 | NCT05410717 | Single-center | 200.0 | 2022-06-01 | No | No | No | No | No | IV |
| NCT06215950 | NCT06215950 | Single-center | 36.0 | 2024-01-10 | No | No | No | No | No | IV |
| NCT03692637 | NCT03692637 | Single-center | 30.0 | 2019-03-01 | No | No | No | No | No | Unclear |
| NCT05225363 | NCT05225363 | Single-center | 33.0 | 2022-05-05 | Yes | No | Yes | No | No | IV |
| NCT07435701 | NCT07435701 | Single-center | 29.0 | 2026-01-05 | No | Yes | No | No | No | IP |
| NCT05922930 | NCT05922930 | Single-center | 51.0 | 2023-10-11 | Yes | No | No | No | No | IP |
| NCT05856643 | NCT05856643 | Single-center | 12.0 | 2024-08-13 | No | No | No | No | No | IV |
| NCT07410676 | NCT07410676 | Single-center | 83.0 | 2026-02-01 | Yes | No | Yes | No | No | Unclear |
| NCT05963100 | NCT05963100 | Single-center | 10.0 | 2023-08-07 | No | No | No | No | No | IV |
| NCT02118285 | NCT02118285 | Single-center | 2.0 | 2014-07-28 | Yes | Yes | No | No | No | IP |
| NCT03691376 | NCT03691376 | Single-center | 4.0 | 2019-03-08 | Yes | Yes | No | No | No | IV |
| NCT01583686 | NCT01583686 | Single-center | 15.0 | 2012-05-04 | Yes | Yes | No | No | No | Unclear |
| NCT03213964 | NCT03213964 | Single-center | 10.0 | 2017-10-19 | No | Yes | No | No | No | IP |
| NCT01105650 | NCT01105650 | Single-center | 13.0 | 2010-07-01 | Yes | Yes | No | No | No | Unclear |
| NCT00652899 | NCT00652899 | Single-center | 14.0 | 2008-03-01 | Yes | Yes | No | No | No | IV |
| NCT06321484 | NCT06321484 | Multicenter single-country | 18.0 | 2024-10-09 | No | Yes | No | No | No | IP |
| NCT07651124 | NCT07651124 | Single-center | 6.0 | 2025-04-01 | Yes | Yes | No | No | No | Unclear |
| NCT05518253 | NCT05518253 | Single-center | 30.0 | 2022-05-30 | No | No | No | No | No | IV |
| NCT05420545 | NCT05420545 | Single-center | 36.0 | 2021-12-31 | No | No | No | No | No | IV |
| NCT03539406 | NCT03539406 | Single-center | 11.0 | 2019-06-04 | No | No | No | No | No | IP |
| NCT07613723 | NCT07613723 | Multicenter single-country | 9.0 | 2026-05-01 | Yes | No | No | No | No | IV |
| NCT03356795 | NCT03356795 | Single-center | 20.0 | 2026-06-01 | No | No | No | No | No | IV |
| NCT03585764 | NCT03585764 | Single-center | 46.0 | 2018-10-24 | Yes | No | No | No | No | IP |
| NCT02580747 | NCT02580747 | Single-center | 20.0 | 2015-10-01 | No | No | No | No | No | IV |
| NCT07096583 | NCT07096583 | Single-center | 20.0 | 2025-07-07 | No | No | No | No | No | IV |
| NCT03634501 | NCT03634501 | Single-center | 200.0 | 2018-10-26 | No | No | No | No | No | IV |
| NCT05372692 | NCT05372692 | Single-center | 3.0 | 2022-04-12 | No | No | No | No | No | Unclear |
| NCT03916679 | NCT03916679 | Single-center | 20.0 | 2019-04-20 | No | No | Yes | No | No | IV |
| NCT03735589 | NCT03735589 | Single-center | 0.0 | 2023-12-15 | No | No | No | No | No | IP |
| NCT06469281 | NCT06469281 | Multicenter single-country | 90.0 | 2024-08-06 | No | No | No | No | Yes | IV |
| NCT06395844 | NCT06395844 | Single-center | 28.0 | 2024-05-01 | No | No | No | No | No | IP |
| NCT00101257 | NCT00101257 | Single-center | 18.0 | 2004-10-01 | No | No | No | No | No | IV |
| NCT03799913 | NCT03799913 | Single-center | 20.0 | 2019-04-10 | Yes | No | Yes | No | No | IV |
| NCT03054298 | NCT03054298 | Single-center | 65.0 | 2017-04-06 | Yes | No | No | No | No | IV |
| NCT06884345 | NCT06884345 | Single-center | 28.0 | 2025-03-13 | No | No | No | No | Yes | Unclear |
| NCT03814447 | NCT03814447 | Single-center | 10.0 | 2019-08-16 | Yes | No | No | No | No | IV |
| NCT02280811 | NCT02280811 | Single-center | 12.0 | 2014-10-14 | Yes | Yes | Yes | No | No | IV |
| NCT00019084 | NCT00019084 | Single-center |  | 1996-02-01 | No | Yes | No | No | No | Unclear |
| NCT03412877 | NCT03412877 | Single-center | 285.0 | 2018-09-06 | Yes | Yes | Yes | No | No | IV |
| NCT02713984 | NCT02713984 | Single-center | 0.0 | 2016-03-01 | No | No | No | No | No | Unclear |
| NCT06284226 | NCT06284226 | Single-center | 0.0 | 2022-03-25 | No | No | No | No | No | Unclear |
| NCT02457650 | NCT02457650 | Single-center | 36.0 | 2015-04-01 | Yes | No | No | No | No | IV |
| NCT06885697 | NCT06885697 | Single-center | 100.0 | 2025-07-08 | Yes | No | No | No | No | IV |
| NCT05592626 | NCT05592626 | Multinational | 365.0 | 2023-01-04 | No | No | No | No | No | IV |
| NCT02482090 | NCT02482090 | Single-center | 6.0 | 2015-07-01 | Yes | Yes | No | No | No | IV |
| NCT06626256 | NCT06626256 | Single-center | 0.0 | 2025-12-05 | Yes | Yes | No | No | No | IV |
| NCT05296564 | NCT05296564 | Single-center | 3.0 | 2022-04-01 | Yes | Yes | No | No | No | IV |
| NCT02153905 | NCT02153905 | Single-center | 3.0 | 2014-07-03 | Yes | Yes | No | No | No | IV |
| JPRN-jRCTc031200283 | JPRN-jRCTc031200283 | Single-center | 14.0 | 2021-10-19 | Yes | Yes | No | No | No | IV |
| ChiCTR1800019780 | ChiCTR1800019780 | Single-center | 36.0 | 2018-11-28 | No | No | No | No | No | IV |

# **S2A. Included trial records — Part 4 of 5: endpoints and reporting status**

**Note:** *One row represents one deduplicated trial record. The 211 records are repeated across five field groups and can be joined using Trial ID. Broader-basket eligibility does not imply that gynecologic participants were enrolled or that cohort-specific outcomes are available.*

| **TrialID** | **PublicregistryID** | **Primaryendpointtext** | **Secondaryendpointtext** | **Registryresultsposted** | **Publishedarticlefound** | **PMID** | **DOI** |
| --- | --- | --- | --- | --- | --- | --- | --- |
| NCT07644403 | NCT07644403 | Overall response rate Response evaluation criteria in solid tumors Platelet count Treatment Emergent Adverse Events Leukocyte count Hemoglobin Dose-limiting toxicities Maximum tolerated dose Safety And Tolerability | Response rate Area under the curve score Cmax Plasma pharmacokinetics Tmax Immunogenicity Safety And Tolerability Viral replication | No | No |  |  |
| NCT07534813 | NCT07534813 | Medication use Adverse Events Common Terminology Criteria for Adverse Events | Overall response rate Progression-free survival Plasma concentration | No | No |  |  |
| 695613 | EUCT 2024-517594-24-01 |  |  | No | No |  |  |
| NCT07420010 | NCT07420010 | Adverse Events Dose-limiting toxicities Maximum tolerated dose Safety And Tolerability Serious Adverse Event | Clinical benefit rate Overall response rate Overall response rate - duration Overall survival Progression-free survival Plasma pharmacokinetics | No | No |  |  |
| NCT07266298 | NCT07266298 | Common Terminology Criteria for Adverse Events Dose-limiting toxicities Maximum tolerated dose Safety And Tolerability | Duration of overall response Clinical benefit rate Overall response rate Response evaluation criteria in solid tumors Overall survival Progression-free survival Area under the curve score Cmax Plasma pharmacokinetics Tmax | No | No |  |  |
| NCT07231081 | NCT07231081 | Adverse Events Dose-limiting toxicities Maximum tolerated dose | Clinical benefit rate Overall response rate Overall response rate - duration Time to progression Time to Remission Immunogenicity (other timeframe) Overall survival Progression-free survival Area under the curve score Cmax Immunogenicity | No | No |  |  |
| NCT07216105 | NCT07216105 | Dose-limiting toxicities Safety And Tolerability | Duration of overall response Overall response rate Response evaluation criteria in solid tumors Overall survival Progression-free survival | No | No |  |  |
| 668508 | ChiCTR2500109506 | Adverse Events Dose-limiting toxicities Safety And Tolerability | Clinical benefit rate Immune Response Overall response rate Overall response rate - duration Overall survival Progression-free survival Leukocyte count | No | No |  |  |
| NCT07181720 | NCT07181720 | Common Terminology Criteria for Adverse Events Dose-limiting toxicities | Duration of overall response Clinical benefit rate Overall response rate Overall survival Progression-free survival Area under the curve score Cmax Tmax | No | No |  |  |
| NCT07106814 | NCT07106814 | Adverse Events Common Terminology Criteria for Adverse Events | Duration of overall response Clinical benefit rate Overall response rate Overall survival Progression-free survival Plasma pharmacokinetics | No | No |  |  |
| NCT07101549 | NCT07101549 | Adverse Events Treatment Emergent Adverse Events Dose-limiting toxicities Maximum tolerated dose Safety And Tolerability | Time to Death Percentage of Responders Clinical benefit rate Overall response rate Response rate Time to progression Immunogenicity (other timeframe) Overall survival Progression-free survival Area under the curve score Cmax Plasma pharmacokinetics Tmax Immunogenicity | No | No |  |  |
| NCT07067255 | NCT07067255 | Dose-limiting toxicities | Maximum tolerated dose | No | No |  |  |
| NCT07030907 | NCT07030907 |  |  | No | No |  |  |
| NCT07010523 | NCT07010523 | Adverse Events Dose-limiting toxicities Maximum tolerated dose Serious Adverse Event | Clinical benefit rate Overall response rate Overall response rate - duration Overall survival Progression-free survival Area under the curve score Cmax Tmax Immunogenicity | No | No |  |  |
| NCT06951425 | NCT06951425 | Adverse Events Dose-limiting toxicities | Clinical benefit rate Overall response rate Response evaluation criteria in solid tumors Time to progression Overall survival Progression-free survival | No | No |  |  |
| NCT06912152 | NCT06912152 | Adverse Events Common Terminology Criteria for Adverse Events | Clinical benefit rate Overall response rate Overall response rate - duration Overall survival Progression-free survival Accumulation index Area under the curve score Cmax Cmin Concentration at steady state Elimination half-life Plasma concentration Tmax Dose-limiting toxicities Maximum tolerated dose | No | No |  |  |
| NCT06904131 | NCT06904131 |  |  | No | No |  |  |
| NCT06846424 | NCT06846424 | Common Terminology Criteria for Adverse Events Dose-limiting toxicities | Overall response rate | No | No |  |  |
| NCT06834542 | NCT06834542 |  |  | No | No |  |  |
| NCT06748872 | NCT06748872 | Adverse Events Dose-limiting toxicities | Time to Death Duration of overall response Clinical benefit rate Overall response rate Overall response rate - duration Overall response rate - time Response evaluation criteria in solid tumors Response rate Time to response Overall survival Progression-free survival | No | No |  |  |
| NCT06710288 | NCT06710288 | Cancer Antigen 125 Clinical benefit rate Overall response rate Overall response rate - duration Overall survival Progression-free survival | Reduction in blood pressure Change in oxygen saturation Adverse Events Heart rate | No | No |  |  |
| 545596 | EUCT 2023-504632-17-00 | Overall response rate | Microbiome Clinical benefit rate Immune Response Overall response rate Overall response rate - duration Immunogenicity (other timeframe) Overall survival Progression-free survival Adverse Events Common Terminology Criteria for Adverse Events Treatment Emergent Adverse Events Cardiac Telemetry Dose-limiting toxicities Serious Adverse Event Vital signs | No | No |  |  |
| NCT06697665 | NCT06697665 | Adverse Events Safety And Tolerability Serious Adverse Event | Clinical benefit rate Overall response rate Overall response rate - duration Time to progression Time to response Overall survival Progression-free survival | No | No |  |  |
| NCT06658951 | NCT06658951 | Adverse Events Dose-limiting toxicities Maximum tolerated dose | Clinical benefit rate Overall response rate Overall survival Progression-free survival | No | No |  |  |
| NCT06646627 | NCT06646627 | Dose-limiting toxicities Maximum tolerated dose | Overall response rate Overall response rate - duration | No | No |  |  |
| NCT06630611 | NCT06630611 | Adverse Events Patient-Reported Outcomes version of the Common Terminology Criteria for Adverse Events | Overall response rate Overall response rate - duration Tumor control rate EORTC Quality of Life Questionnaire Mobility measurement Change in oxygen saturation Sleep Activity Sleep parameters by Polysomnography Overall survival Progression-free survival Cost effectiveness Acceptability of Intervention EQ-5D EQ-5D-5L Hospital Anxiety and Depression Scale Adverse Events Common Terminology Criteria for Adverse Events Heart rate | No | No |  |  |
| NCT06598371 | NCT06598371 |  |  | No | No |  |  |
| NCT06544720 | NCT06544720 | Plasma pharmacokinetics Dose-limiting toxicities Safety And Tolerability | Immune-related response evaluation criteria in solid tumors Response evaluation criteria in solid tumors | No | No |  |  |
| NCT06509490 | NCT06509490 | Treatment Emergent Adverse Events Dose-limiting toxicities | Complete response Overall response rate Overall survival Progression-free survival | No | No |  |  |
| NCT06505551 | NCT06505551 | Overall response rate Adverse Events Treatment Emergent Adverse Events Dose-limiting toxicities Safety And Tolerability | Duration of overall response Clinical benefit rate Overall response rate Overall response rate - duration Time to response Overall survival Progression-free survival Plasma pharmacokinetics Immunogenicity Treatment Emergent Adverse Events | No | No |  |  |
| NCT06481592 | NCT06481592 | Overall response rate Response evaluation criteria in solid tumors | Duration of overall response Clinical benefit rate Complete response Overall survival Progression-free survival Adverse Events | No | No |  |  |
| NCT06453057 | NCT06453057 |  |  | No | No |  |  |
| 518933 | ChiCTR2000032124 |  |  | No | No |  |  |
| NCT06375187 | NCT06375187 | Adverse Events Dose-limiting toxicities Maximum tolerated dose | Duration of overall response Clinical benefit rate Overall response rate EORTC Quality of Life Questionnaire Overall survival Progression-free survival | No | No |  |  |
| NCT06358053 | NCT06358053 | Adverse Events Dose-limiting toxicities Safety And Tolerability | Clinical benefit rate Immune Response Overall response rate Overall response rate - duration Tumor control rate Overall survival Progression-free survival Immunogenicity Viral replication | No | No |  |  |
| 510939 |  |  |  | No | No |  |  |
| NCT06342336 | NCT06342336 | Adverse Events Common Terminology Criteria for Adverse Events Serious Adverse Event | Clinical benefit rate Overall response rate Overall response rate - duration Overall response rate - time Tumor control rate Time to response Immunogenicity (other timeframe) Overall survival Progression-free survival Plasma pharmacokinetics Adverse Events Serious Adverse Event | No | No |  |  |
| NCT06327997 | NCT06327997 | Dose-limiting toxicities Safety And Tolerability | Patient survival Progressive disease rate Overall response rate Response evaluation criteria in solid tumors Overall survival Progression-free survival Area under the curve score Cmax Plasma pharmacokinetics Maximum tolerated dose Safety And Tolerability | No | No |  |  |
| NCT06305299 | NCT06305299 | CRS grading Common Terminology Criteria for Adverse Events Dose-limiting toxicities | CRS grading Clinical benefit rate Response evaluation criteria in solid tumors Overall survival Progression-free survival Tolerability score | No | No |  |  |
| NCT06256055 | NCT06256055 | Adverse Events Treatment Emergent Adverse Events Cardiac Telemetry Dose-limiting toxicities Vital signs | Duration of overall response Clinical benefit rate Overall response rate Overall response rate - duration Immunogenicity (other timeframe) Overall survival Progression-free survival Area under the curve score Cmax Elimination half-life Tmax Volume of distribution Immunogenicity Safety And Tolerability | No | No |  |  |
| NCT06241781 | NCT06241781 | Progression-free survival | Overall response rate - duration Treatment Emergent Adverse Events | No | No |  |  |
| NCT06218914 | NCT06218914 | Treatment Emergent Adverse Events Dose-limiting toxicities | Duration of overall response Clinical benefit rate Overall response rate Overall response rate - duration Overall response rate - time Time to response Overall survival Progression-free survival | No | No |  |  |
| NCT06191900 | NCT06191900 |  |  | No | No |  |  |
| 496428 | CTR20233999 | Adverse Events Dose-limiting toxicities Serious Adverse Event | Duration of overall response Clinical benefit rate Overall response rate Overall response rate - time Time to progression Overall survival Progression-free survival | No | No |  |  |
| NCT06145802 | NCT06145802 | Common Terminology Criteria for Adverse Events Cardiac Telemetry Vital signs | Duration of overall response Clinical benefit rate Overall response rate Overall survival Progression-free survival Plasma pharmacokinetics | No | No |  |  |
| 489390 |  |  |  | No | No |  |  |
| NCT06051695 | NCT06051695 | CRS grading Overall response rate Dose-limiting toxicities Safety And Tolerability | Overall survival Progression-free survival | No | No |  |  |
| NCT06010875 | NCT06010875 | Adverse Events Common Terminology Criteria for Adverse Events Dose-limiting toxicities Maximum tolerated dose | Clinical benefit rate Overall response rate Overall response rate - duration Overall survival Progression-free survival Area under the curve score Cmax Tmax Safety And Tolerability | No | No |  |  |
| NCT05973487 | NCT05973487 | Adverse Events Dose-limiting toxicities Safety And Tolerability Serious Adverse Event | Immune Response Overall response rate Overall response rate - duration Response evaluation criteria in solid tumors Response rate Adverse Events Dose-limiting toxicities Serious Adverse Event | No | No |  |  |
| NCT05971576 | NCT05971576 | Adverse Events Serious Adverse Event | Clinical benefit rate Overall response rate Overall response rate - duration Time to progression Time to response Overall survival Progression-free survival | No | No |  |  |
| NCT05952947 | NCT05952947 | Adverse Events Dose-limiting toxicities Maximum tolerated dose Safety And Tolerability | Duration of overall response Clinical benefit rate Overall response rate Time to response Overall survival Progression-free survival Area under the curve score Cmax Tmax Immunogenicity Safety And Tolerability Viral replication | No | No |  |  |
| NCT05941936 | NCT05941936 | Adverse Events Serious Adverse Event | Clinical benefit rate Immune Response Overall response rate Overall response rate - duration Time to progression Time to response Gene expression profiling Overall survival Progression-free survival Leukocyte count | No | No |  |  |
| NCT05902520 | NCT05902520 | Treatment Emergent Adverse Events Safety And Tolerability | Persistence of response Response evaluation criteria in solid tumors Immunogenicity (other timeframe) | No | No |  |  |
| NCT05877599 | NCT05877599 | Clinical benefit rate Overall response rate Overall response rate - duration Overall response rate - time Progression-free survival Adverse Events Treatment Emergent Adverse Events Dose-limiting toxicities Maximum tolerated dose Safety And Tolerability | Clinical benefit rate Overall response rate Overall response rate - duration Overall response rate - time Overall survival Progression-free survival Plasma pharmacokinetics | No | No |  |  |
| NCT05848999 | NCT05848999 | Adverse Events Dose-limiting toxicities Maximum tolerated dose Serious Adverse Event | Clinical benefit rate Overall response rate Overall response rate - duration Overall survival Progression-free survival Area under the curve score Cmax Tmax Immunogenicity | No | No |  |  |
| NCT05787535 | NCT05787535 | Adverse Events Treatment Emergent Adverse Events Dose-limiting toxicities Serious Adverse Event | Overall response rate Overall response rate - duration Overall survival Progression-free survival | No | No |  |  |
| 463409 |  |  |  | No | No |  |  |
| NCT05783089 | NCT05783089 | Adverse Events Dose-limiting toxicities Maximum tolerated dose Serious Adverse Event | Clinical benefit rate Overall response rate Overall response rate - duration Overall survival Progression-free survival Immunogenicity | No | No |  |  |
| NCT05775666 | NCT05775666 | Adverse Events Dose-limiting toxicities Maximum tolerated dose Serious Adverse Event | Duration of overall response Clinical benefit rate Overall response rate Overall survival Progression-free survival Area under the curve score Cmax Tmax Immunogenicity | No | No |  |  |
| NCT05729399 | NCT05729399 | Common Terminology Criteria for Adverse Events Treatment Emergent Adverse Events Dose-limiting toxicities Serious Adverse Event | Duration of overall response Clinical benefit rate Overall response rate Overall survival Progression-free survival | No | No |  |  |
| 456726 |  |  |  | No | No |  |  |
| NCT05672459 | NCT05672459 | Overall response rate Common Terminology Criteria for Adverse Events Safety And Tolerability | Immune Response Plasma pharmacokinetics Safety And Tolerability | No | No |  |  |
| NCT05881525 | NCT05881525 | Overall response rate Adverse Events Common Terminology Criteria for Adverse Events Treatment Emergent Adverse Events Dose-limiting toxicities | Duration of overall response Clinical benefit rate Overall response rate Overall response rate - duration Overall response rate - time Overall survival Progression-free survival Area under the curve score Cmax Tmax Immunogenicity | No | No |  |  |
| 443882 | ChiCTR2200064112 | Overall response rate Safety And Tolerability | Overall response rate - duration Progression-free survival Adverse Events Safety And Tolerability | No | No |  |  |
| NCT05568680 | NCT05568680 | Patient survival Persistence of response Overall response rate Rate of discontinuation Adverse Events Treatment Emergent Adverse Events Maximum tolerated dose Safety And Tolerability | Immune Response Immune-related response evaluation criteria in solid tumors Overall response rate Overall survival Maximum tolerated dose | No | No |  |  |
| NCT05539768 | NCT05539768 | Adverse Events Safety And Tolerability | Overall response rate Overall response rate - duration Time to response Overall survival | No | No |  |  |
| 439506 | CTR20221207 | Adverse Events Dose-limiting toxicities Maximum tolerated dose Serious Adverse Event | Clinical benefit rate Immune Response Immune-related response evaluation criteria in solid tumors Overall response rate Overall response rate - duration EORTC Quality of Life Questionnaire Progression-free survival | No | No |  |  |
| NCT05483491 | NCT05483491 | Dose-limiting toxicities Maximum tolerated dose | Overall response rate Overall response rate - duration Response evaluation criteria in solid tumors Adverse Events Common Terminology Criteria for Adverse Events | No | No |  |  |
| NCT05475847 | NCT05475847 | Adverse Events Treatment Emergent Adverse Events | Clinical benefit rate Disease response Overall response rate Overall response rate - duration Time to progression Time to response Overall survival Progression-free survival | No | No |  |  |
| NCT05472857 | NCT05472857 | Adverse Events Treatment Emergent Adverse Events Dose-limiting toxicities Maximum tolerated dose | Duration of overall response Clinical benefit rate Overall response rate Progression-free survival | No | No |  |  |
| NCT05468307 | NCT05468307 | Clinical benefit rate Overall response rate Overall response rate - duration Overall survival Progression-free survival Adverse Events Treatment Emergent Adverse Events Safety And Tolerability | Clinical benefit rate Overall response rate EORTC Quality of Life Questionnaire Overall survival Progression-free survival Quality of Life Plasma pharmacokinetics | No | No |  |  |
| NCT05468190 | NCT05468190 | Adverse Events Common Terminology Criteria for Adverse Events Dose-limiting toxicities Maximum tolerated dose | Clinical benefit rate Overall response rate Overall response rate - duration Overall survival Progression-free survival Area under the curve score Cmax Tmax Serum ferritin Safety And Tolerability | No | No |  |  |
| NCT05430373 | NCT05430373 | Duration of overall response Clinical benefit rate Overall response rate Overall survival Progression-free survival Adverse Events Common Terminology Criteria for Adverse Events Treatment Emergent Adverse Events Cardiac Telemetry Dose-limiting toxicities Safety And Tolerability Serious Adverse Event Vital signs | Duration of overall response Clinical benefit rate Overall response rate Overall survival Progression-free survival Plasma pharmacokinetics | No | No |  |  |
| NCT05417750 | NCT05417750 | Adverse Events Dose-limiting toxicities Maximum tolerated dose Safety And Tolerability | Duration of overall response Clinical benefit rate Overall response rate Overall response rate - duration EORTC Quality of Life Questionnaire Overall survival Progression-free survival | No | No |  |  |
| NCT05397093 | NCT05397093 | Treatment Emergent Adverse Events Dose-limiting toxicities | HSC Engraftment Progressive disease rate Circulating Tumor Cells Clinical benefit rate Complete response Overall response rate Overall response rate - duration Partial response Stable Disease Time to response Gene expression profiling Overall survival Progression-free survival Rate of discontinuation Plasma pharmacokinetics Adverse Events Treatment Emergent Adverse Events Serious Adverse Event | No | No |  |  |
| NCT05373147 | NCT05373147 | Dose-limiting toxicities Safety And Tolerability | Patient survival Overall response rate Response evaluation criteria in solid tumors Overall survival Progression-free survival Cmax Plasma pharmacokinetics Maximum tolerated dose Safety And Tolerability | No | No |  |  |
| NCT05366478 | NCT05366478 | Adverse Events Common Terminology Criteria for Adverse Events | Clinical benefit rate Immune Response Overall response rate Response evaluation criteria in solid tumors Progression-free survival | No | No |  |  |
| 431389 |  |  |  | No | No |  |  |
| 430435 | CTR20260154 | Overall response rate | Clinical benefit rate Overall response rate - duration Overall survival Progression-free survival | No | No |  |  |
| NCT05342506 | NCT05342506 | Complete response Overall response rate Response evaluation criteria in solid tumors hCG level | Duration of overall response Clinical benefit rate Overall response rate - duration Overall survival Progression-free survival | No | No |  |  |
| NCT05302037 | NCT05302037 | Treatment Emergent Adverse Events Dose-limiting toxicities | Duration of overall response Overall response rate Overall survival Progression-free survival Leukocyte count | No | No |  |  |
| 427237 | ChiCTR2200057362 | Overall response rate Safety And Tolerability | Frequency of CRS Duration of overall response Overall survival Progression-free survival Adverse Events Serious Adverse Event Vital signs | No | No |  |  |
| NCT05238818 | NCT05238818 | Adverse Events Common Terminology Criteria for Adverse Events Dose-limiting toxicities Maximum tolerated dose Serious Adverse Event | Clinical benefit rate Overall response rate Overall response rate - duration Overall response rate - time Overall survival Progression-free survival | No | No |  |  |
| NCT05211557 | NCT05211557 | Lesion increase in size Complete response Overall response rate Partial response Stable Disease Dose-limiting toxicities | Overall survival Progression-free survival | No | No |  |  |
| NCT05194735 | NCT05194735 |  |  | Yes | No |  |  |
| 421887 | ChiCTR2100054914 | PET scan | Overall response rate Response evaluation criteria in solid tumors | No | No |  |  |
| NCT05274451 | NCT05274451 | Treatment Emergent Adverse Events Dose-limiting toxicities Maximum tolerated dose Safety And Tolerability | Persistence of response Overall response rate Overall response rate - duration Response evaluation criteria in solid tumors Overall survival Progression-free survival Area under the curve score Cmax Plasma pharmacokinetics Tmax | No | No |  |  |
| NCT05152797 | NCT05152797 | Overall response rate | Clinical benefit rate Overall survival Progression-free survival Adverse Events | No | No |  |  |
| NCT05141253 | NCT05141253 | Adverse Events Dose-limiting toxicities Serious Adverse Event | Time to Death Duration of overall response Clinical benefit rate Disease response Overall response rate Overall response rate - time Response rate Time to progression Time to response Overall survival Progression-free survival Cmax Cmin Immunogenicity Viral replication | No | No |  |  |
| NCT05357027 | NCT05357027 | Overall response rate Adverse Events Treatment Emergent Adverse Events Dose-limiting toxicities Safety And Tolerability | Cancer Antigen 125 Overall response rate Overall response rate - duration Overall survival Progression-free survival Area under the curve score Cmax Tmax Immunogenicity | No | No |  |  |
| NCT05122221 | NCT05122221 | Adverse Events Common Terminology Criteria for Adverse Events Dose-limiting toxicities Maximum tolerated dose | Clinical benefit rate Overall response rate Overall response rate - duration Progression-free survival | No | No |  |  |
| NCT05098171 | NCT05098171 | Duration of overall response Clinical benefit rate Overall response rate Overall survival Progression-free survival Adverse Events Treatment Emergent Adverse Events | EORTC Quality of Life Questionnaire Quality of Life | No | No |  |  |
| NCT05089266 | NCT05089266 | Dose-limiting toxicities Safety And Tolerability | Overall response rate Response evaluation criteria in solid tumors Maximum tolerated dose Safety And Tolerability | No | No |  |  |
| NCT05499715 | NCT05499715 | Circulating Tumor Cells Clinical benefit rate Overall response rate Overall response rate - duration Disease-free survival Overall survival Adverse Events | Duration of overall response Clinical benefit rate Overall survival Progression-free survival Viral load at week 12 Adverse Events | No | No |  |  |
| NCT04981691 | NCT04981691 | Adverse Events Treatment Emergent Adverse Events Dose-limiting toxicities | Clinical benefit rate Overall response rate Overall response rate - duration Time to Remission EORTC Quality of Life Questionnaire Immunogenicity at 28 days Overall survival Progression-free survival Cmax Adverse Events Immunogenicity Treatment Emergent Adverse Events Serious Adverse Event | No | No |  |  |
| NCT05393635 | NCT05393635 | Adverse Events Treatment Emergent Adverse Events Serious Adverse Event | Duration of overall response Overall response rate Overall response rate - duration Overall survival Progression-free survival | No | No |  |  |
| 403252 |  |  |  | No | No |  |  |
| NCT04766320 | NCT04766320 | Common Terminology Criteria for Adverse Events Treatment Emergent Adverse Events Safety And Tolerability Serious Adverse Event | Percentage of Responders Progressive disease rate Clinical benefit rate Complete response Overall response rate Overall response rate - duration Partial response Response evaluation criteria in solid tumors Stable Disease Time to progression Overall survival Progression-free survival | No | No |  |  |
| NCT05107739 | NCT05107739 | Incidence of death or cerebral palsy Discontinuation Duration of overall response Clinical benefit rate Overall response rate Overall response rate - duration Overall survival Progression-free survival Treatment Emergent Adverse Events Dose-limiting toxicities Safety And Tolerability | Immune Response | No | No |  |  |
| 394743 | ChiCTR2100042320 | Overall response rate Adverse Events | Overall response rate - duration Progression-free survival | No | No |  |  |
| NCT04674488 | NCT04674488 | Dose-limiting toxicities | Fraction of Survivors Clinical benefit rate Immune Response Overall response rate Overall response rate - duration Tumor control rate Overall survival Progression-free survival | No | No |  |  |
| NCT05239143 | NCT05239143 | Disease response Immune-related response evaluation criteria in solid tumors Overall response rate Adverse Events Dose-limiting toxicities Maximum tolerated dose Safety And Tolerability | Circulating Tumor Cells | No | No |  |  |
| NCT04670068 | NCT04670068 | Dose-limiting toxicities Safety And Tolerability | Clinical benefit rate Immune Response Response evaluation criteria in solid tumors Response rate Gene expression profiling Overall survival Progression-free survival | Yes | No |  |  |
| 389985 |  |  |  | No | No |  |  |
| 389901 |  |  |  | No | No |  |  |
| NCT04627740 | NCT04627740 | Treatment Emergent Adverse Events | Overall response rate Progression-free survival | No | No |  |  |
| NCT04611126 | NCT04611126 | Adverse Events Common Terminology Criteria for Adverse Events Safety And Tolerability | Complete response Immune Response Overall response rate Partial response Overall survival Progression-free survival | Yes | No |  |  |
| NCT04562298 | NCT04562298 | Treatment Emergent Adverse Events Dose-limiting toxicities Maximum tolerated dose | Clinical benefit rate Overall response rate Overall response rate - duration Time to response Overall survival Progression-free survival | No | No |  |  |
| NCT04556669 | NCT04556669 | Treatment Emergent Adverse Events Safety And Tolerability | Objective remission rate Overall response rate - duration Overall survival Progression-free survival Area under the curve score Cmax Tmax | No | No |  |  |
| NCT04483778 | NCT04483778 | Adverse Events Dose-limiting toxicities Maximum tolerated dose Safety And Tolerability | Overall response rate | No | No |  |  |
| NCT04476251 | NCT04476251 |  |  | Yes | No |  |  |
| NCT04443296 | NCT04443296 | Common Terminology Criteria for Adverse Events Dose-limiting toxicities | Clinical benefit rate Complete response Complete response-duration Immune Response Overall response rate Partial response Stable Disease Disease Progression - duration | No | No |  |  |
| NCT04389229 | NCT04389229 | Response evaluation criteria in solid tumors Common Terminology Criteria for Adverse Events | Cancer Antigen 125 Overall response rate Overall response rate - duration Progression-free survival Common Terminology Criteria for Adverse Events | No | No |  |  |
| 372638 |  |  |  | No | No |  |  |
| NCT05035407 | NCT05035407 | Dose-limiting toxicities Maximum tolerated dose | Immune Response Tumor control rate Safety And Tolerability | Yes | No |  |  |
| NCT04318964 | NCT04318964 | Dose-limiting toxicities Maximum tolerated dose | Area under the curve score Cmax Tmax | No | No |  |  |
| NCT04503278 | NCT04503278 | Progressive disease rate Complete response Partial response Stable Disease Rate of discontinuation Adverse Events Treatment Emergent Adverse Events Dose-limiting toxicities Maximum tolerated dose Safety And Tolerability | Duration of overall response Clinical benefit rate Immune Response Overall response rate Overall response rate - duration | No | No |  |  |
| NCT04025216 | NCT04025216 | Overall response rate Dose-limiting toxicities | Clinical benefit rate Minimal Residual Disease Overall response rate Overall response rate - duration Overall response rate - time Time to progression Overall survival Progression-free survival Adverse Events Safety And Tolerability | No | No |  |  |
| NCT03992326 | NCT03992326 | Common Terminology Criteria for Adverse Events | Clinical benefit rate Complete response Overall response rate Partial response Stable Disease Overall survival Progression-free survival | No | No |  |  |
| NCT03970382 | NCT03970382 | Adverse Events Maximum tolerated dose Safety And Tolerability | Immune Response Overall response rate Overall response rate - duration Overall survival Progression-free survival Area under the curve score Cmax Plasma pharmacokinetics | No | No |  |  |
| NCT03912831 | NCT03912831 | Overall response rate Dose-limiting toxicities | Overall response rate - duration Overall survival Progression-free survival Adverse Events Immunogenicity | No | No |  |  |
| NCT03907527 | NCT03907527 | Adverse Events Common Terminology Criteria for Adverse Events Maximum tolerated dose | Response evaluation criteria in solid tumors | No | No |  |  |
| NCT04650451 | NCT04650451 | Dose-limiting toxicities Maximum tolerated dose | Overall response rate | No | No |  |  |
| NCT03745326 | NCT03745326 | Number of Participants With Clinical Response Overall response rate Common Terminology Criteria for Adverse Events Dose-limiting toxicities | Common Terminology Criteria for Adverse Events | No | No |  |  |
| NCT03686124 | NCT03686124 | Overall response rate Adverse Events Treatment Emergent Adverse Events Dose-limiting toxicities Maximum tolerated dose Safety And Tolerability | Persistence of response Duration of overall response Clinical benefit rate Immune Response Immune-related response evaluation criteria in solid tumors Overall response rate Response evaluation criteria in solid tumors EORTC Quality of Life Questionnaire Overall survival Progression-free survival EQ-5D-5L | No | No |  |  |
| NCT03638206 | NCT03638206 | Common Terminology Criteria for Adverse Events Safety And Tolerability | Overall response rate - time | No | No |  |  |
| NCT03610490 | NCT03610490 | Overall response rate | Duration of overall response Clinical benefit rate Immune Response Immune-related response evaluation criteria in solid tumors Overall response rate - duration Response rate Overall survival Progression-free survival Quality of Life Adverse Events Treatment Emergent Adverse Events Safety And Tolerability Serious Adverse Event | Yes | No |  |  |
| NCT03578406 | NCT03578406 |  |  | No | No |  |  |
| 327082 | ChiCTR1800016805 | Body weight Overall response rate Response evaluation criteria in solid tumors Tumor control rate Magnetic Resonance Imaging C reactive protein Quality of Life Cardiac Telemetry Vital signs | Clinical benefit rate Overall survival Progression-free survival | No | No |  |  |
| 326892 | ChiCTR1800016764 |  |  | No | No |  |  |
| NCT03545815 | NCT03545815 | Adverse Events Common Terminology Criteria for Adverse Events Treatment Emergent Adverse Events Dose-limiting toxicities | Clinical benefit rate Overall response rate Progression-free survival | No | No |  |  |
| NCT03449108 | NCT03449108 | Overall response rate Safety And Tolerability | Cancer Antigen 125 Duration of overall response Clinical benefit rate Immune Response Immune-related response evaluation criteria in solid tumors EORTC Quality of Life Questionnaire Overall survival Progression-free survival Adverse Events | No | No |  |  |
| NCT04102436 | NCT04102436 | Overall response rate Response rate | Immune Response Common Terminology Criteria for Adverse Events | No | No |  |  |
| NCT03412526 | NCT03412526 | Overall response rate Common Terminology Criteria for Adverse Events | Overall response rate Overall survival Progression-free survival Quality of Life | No | No |  |  |
| NCT03907852 | NCT03907852 | Clinical benefit rate Overall response rate Dose-limiting toxicities Maximum tolerated dose Safety And Tolerability | Duration of overall response Clinical benefit rate Overall response rate Immunogenicity (other timeframe) Overall survival Progression-free survival Safety And Tolerability | No | No |  |  |
| NCT03296137 | NCT03296137 | Adverse Events Common Terminology Criteria for Adverse Events Safety And Tolerability | Patient survival Immune Response Immune-related response evaluation criteria in solid tumors Overall response rate Response evaluation criteria in solid tumors Time to progression Overall survival Progression-free survival | No | No |  |  |
| NCT04072263 | NCT04072263 | Common Terminology Criteria for Adverse Events Treatment Emergent Adverse Events Dose-limiting toxicities Safety And Tolerability NCI-CTC scale | Cancer Antigen 125 Number of Participants With Clinical Response Clinical benefit rate Immune Response Immune-related response evaluation criteria in solid tumors Response evaluation criteria in solid tumors Overall survival Progression-free survival | No | No |  |  |
| NCT03287674 | NCT03287674 | Adverse Events Common Terminology Criteria for Adverse Events | Immune Response Overall response rate Response evaluation criteria in solid tumors Immunogenicity (other timeframe) Overall survival Progression-free survival | Yes | No |  |  |
| NCT03158935 | NCT03158935 | Serious Adverse Event | Overall response rate - time Response rate Overall survival Progression-free survival Common Terminology Criteria for Adverse Events | No | No |  |  |
| NCT03159585 | NCT03159585 | Treatment Emergent Adverse Events Safety And Tolerability | Overall response rate Overall response rate - duration Time to progression Overall survival Progression-free survival | No | No |  |  |
| NCT03108495 | NCT03108495 | Overall response rate Adverse Events Treatment Emergent Adverse Events Safety And Tolerability | Clinical benefit rate Immune Response Immune-related response evaluation criteria in solid tumors Overall response rate Overall response rate - duration Overall survival Progression-free survival Quality-adjusted life year Quality of Life Adverse Events Treatment Emergent Adverse Events | No | No |  |  |
| NCT03132922 | NCT03132922 | Adverse Events Dose-limiting toxicities Safety And Tolerability Viral replication | Opportunistic infection Disease flares Persistence of response Duration of overall response Immune Response Overall response rate Overall response rate - time Response evaluation criteria in solid tumors Disease Progression - duration Overall survival Progression-free survival Adverse Events Treatment Emergent Adverse Events | Yes | No |  |  |
| NCT02930993 | NCT02930993 | Treatment Emergent Adverse Events | Overall response rate Overall survival Progression-free survival | No | No |  |  |
| NCT02858310 | NCT02858310 | Complete response Overall response rate Response evaluation criteria in solid tumors Adverse Events Common Terminology Criteria for Adverse Events Dose-limiting toxicities Maximum tolerated dose | Circulating Tumor Cells Immune Response Overall survival Progression-free survival Adverse Events | Yes | No |  |  |
| NCT02650986 | NCT02650986 | Dose-limiting toxicities | Microbiome Immune-related response evaluation criteria in solid tumors Tumor control rate Viral replication | Yes | No |  |  |
| NCT02498912 | NCT02498912 | Dose-limiting toxicities Maximum tolerated dose Safety And Tolerability | Immune-related response evaluation criteria in solid tumors Overall response rate | No | No |  |  |
| NCT02159716 | NCT02159716 | Adverse Events Safety And Tolerability | Immunogenicity (other timeframe) Overall survival Progression-free survival | No | No |  |  |
| NCT02111850 | NCT02111850 | Overall response rate Adverse Events Treatment Emergent Adverse Events Dose-limiting toxicities Maximum tolerated dose | Common Terminology Criteria for Adverse Events Dose-limiting toxicities | Yes | No |  |  |
| NCT01883297 | NCT01883297 | Common Terminology Criteria for Adverse Events Treatment Emergent Adverse Events NCI-CTC scale | Histological Response Immune Response Response evaluation criteria in solid tumors | No | No |  |  |
| NCT01585428 | NCT01585428 | Lesion increase in size Lesion reduction in size Number of Participants With Clinical Response Complete response Stable Disease | Adverse Events | Yes | No |  |  |
| NCT01567891 | NCT01567891 | Adverse Events Common Terminology Criteria for Adverse Events Safety And Tolerability | Duration of overall response Immune Response Overall response rate Overall response rate - duration Overall response rate - time Response rate Time to response Overall survival Progression-free survival | Yes | No |  |  |
| 154752 |  |  |  | No | No |  |  |
| NCT01174121 | NCT01174121 | Overall response rate Tumor control rate | Immune Response Overall response rate CD4+ cell count Adverse Events Treatment Emergent Adverse Events | No | No |  |  |
| 1772 |  |  |  | No | No |  |  |
| NCT05686226 | NCT05686226 | Tumor response, Objective tumor response as measured by RECIST, 5 years | Adverse Events, Adverse events as measured by CTCAE, 5 years | No | No |  |  |
| NCT05639972 | NCT05639972 | Feasibility of administering E7 TCR-T cell therapy as induction treatment for LAHPVC, Proportion of subjects who complete treatment without an event that meets criteria for feasibility failure, 6 weeks | Objective tumor response rate at 6-weeks after treatment, Tumor response as assessed by imaging using Response Evaluation Criteria in Solid Tumors (RECIST) criteria Version 1.1 or PERCIST, 6 weeks\|2-year and 5-year disease free survival (DFS), DFS will be determined using the Kaplan-Meier method, 5 years | No | No |  |  |
| NCT07617753 | NCT07617753 | Incidence of dose-limiting toxicities (DLTs), 28 Days\|Incidence and severity of treatment-emergent adverse events (TEAEs) graded by CTCAE v5.0, 12 months | Objective response rate (ORR) by RECIST v1.1, 6 months\|Duration of response (DOR) among responders, 12 months | No | No |  |  |
| NCT07589543 | NCT07589543 | Incidence of dose-limiting toxicities (DLTs) to determine maximum tolerated dose (MTD), 28 days\|Incidence, severity, and relatedness of adverse events (AEs), 28 days | Objective response rate (ORR) per RECIST v1.1., 12 months\|Overall survival (OS), 12 months | No | No |  |  |
| NCT07410494 | NCT07410494 | Incidence, type, and severity of adverse events (AEs), graded by CTCAE v5.0, 28 DAYS\|Incidence of Dose-Limiting Toxicities (DLTs), 28 DAYS | Objective Response Rate (ORR) (RECIST 1.1), 12 months\|Disease Control Rate (DCR), 12 months | No | No |  |  |
| NCT07480954 | NCT07480954 | Incidence of dose-limiting toxicities (DLTs), 28 Days\|Incidence and severity of treatment-emergent adverse events, Incidence and severity of treatment-emergent adverse events (TEAEs) graded by CTCAE v5.0 (including CRS and ICANS), 12 months | Objective response rate (ORR), Objective response rate (ORR) by RECIST v1.1 (CR + PR), 6 months\|Duration of response (DOR) among responders, 12 months\|Progression-free survival (PFS), 12 months\|Overall survival (OS), 24 months | No | No |  |  |
| NCT05776355 | NCT05776355 | DLT, Dose-Limiting Toxicity, 28 days\|MTD, Maximal Tolerable Dose, 28 days |  | No | No |  |  |
| NCT05410717 | NCT05410717 | Safety by Common Terminology Criteria for Adverse Events (CTCAE) V5.0, The type, frequency, severity, and duration of adverse events as a result of Claudin6, GPC3, Mesothelin, or AXL- CAR-NK cells infusion will be summarized., After CAR-NK cells infusion, up to 52 weeks. | Objective Response Rate (ORR), Per Response Evaluation Criteria in Solid Tumours (RECIST 1.1) assessed by MRI or CT. ORR defined as the proportion of patients in whom a complete response (CR) or partial response (PR) is observed as best overall response, prior to progression or further anti-cancer therapy., After CAR-NK cells infusion, up to 52 weeks.\|Disease control rate (DCR), Disease control rate (DCR) defined as the proportion of patients in whom a CR or PR or stable disease (SD) (per RECIST 1.1, SD assessed at least 6 weeks after the first dose) is observed as best overall response., up to 52 weeks.\|Duration of response (DOR), Duration of response (DOR) defined as the time from first objective response (CR or PR per RECIST 1.1) to first occurrence of objective tumor progression (Progressive disease (PD) per RECIST 1.1/recurrence) or death from any cause, whichever occurs first., up to 36 months | No | No |  |  |
| NCT06215950 | NCT06215950 | Incidence of Adverse events after CD70 CAR-T cells infusion [Safety and Tolerability], Therapy-related adverse events were recorded and assessed according to the National Cancer Institute's Common Terminology Criteria for Adverse Events (CTCAE, Version 5.0), 28 days\|Obtain the maximum tolerated dose of CD70 CAR-T cells[Safety and Tolerability], Dose-limiting toxicity after cell infusion, 28 days | Disease control rate of CAR-T cell preparations in CD70 positive advanced malignancies [Effectiveness], Disease control rate: The proportion of subjects who achieved CR, PR, SD after CAR-T infusion accounted for all treated subjects (Assessed based on RECIST criteria)，the minimum value is 0%，maximum value is 100%, and higher scores mean a better outcome., 3 months\|Objective response rate (ORR) of CD70 CAR-T treatment in patients with CD70-positive advanced malignancies[Effectiveness], Objective response rate includes：The proportion of subjects who achieved CR, PR after CAR-T infusion accounted for all treated subjects (Assessed based on RECIST criteria)，the minimum value is 0%，maximum value is 100%, and higher scores mean a better outcome., 3 months\|Duration of Response (DOR) of CD70 CAR-T treatment in patients with CD70-positive advanced malignancies[Effectiveness], DOR will be assessed from the first assessment of CR/PR/SD to the first assessment of recurrence or progression of the disease or death from any cause, 2 years\|Progress-free survival(PFS) of CD70 CAR-T treatment in patients with CD70-positive advanced malignancies[Effectiveness], PFS will be assessed from the first CD70-CAR-T cell infusion to death from any cause or the first assessment of progression(Assessed based on RECIST criteria), 2 years\|Overall survival(OS)of CD70 CAR-T treatment in patients with CD70-positive advanced malignancies[Effectiveness], OS will be assessed from the first CD70-CAR-T cell infusion to death from any cause (Assessed based on RECIST criteria), 2 years\|AUCS of CD70 CAR-T cells [Cell dynamics], AUCS is defined as the area under the curve in 90 days, 3 months\|CMAX of CD70 CAR-T cells [Cell dynamics], CMAX is defined as the highest concentration of CD70 CAR-T cells expanded in peripheral blood, 3 months\|TMAX of CD70 CAR-T cells[Cell dynamics], TMAX is defined as the time to reach the highest concentration, 3 months\|Pharmacodynamics of CD70 CAR-T cells[Cell dynamics], Concentration levels of CAR-T-related serum cytokines such as CRP, IL-6, ferritin at each time point，which measured by Chemiluminescence method, 3 months The correlation between CD70 positive rate and safety, assessment the correlation between CD70 positive rate and the incidence of CRA and ICANS, 2 years\|Correlation between CD70 positive rate and efficacy, assessment the correlation between CD70 positive rate and the disease control rate，Disease control rate: including CR, PR and SD, 2 years\|Overall survival(OS)of CD70 CAR-T treatment in patients with CD70-positive advanced malignancies[Effectiveness], OS will be assessed from the first CD70-CAR-T cell infusion to death from any cause (Assessed by investigators based on IRECIST criteria), 2 years\|Progress-free survival(PFS) of CD70 CAR-T treatment in patients with CD70-positive advanced malignancies[Effectiveness], PFS will be assessed from the first CD70-CAR-T cell infusion to death from any cause or the first assessment of progression (Assessed by investigators based on IRECIST criteria), 2 years\|Duration of Response (DOR) of CD70 CAR-T treatment in patients with CD70-positive advanced malignancies[Effectiveness], DOR will be assessed from the first assessment of CR/PR/SD to the first assessment of recurrence or progression of the disease or death from any cause (Assessed by investigators based on IRECIST criteria), 2 years | No | No |  |  |
| NCT03692637 | NCT03692637 | Occurrence of treatment related adverse events as assessed by CTCAE v4.0, defined as \>= Grade 3 signs/symptoms, laboratory toxicities, and clinical events that are possibly, likely, or definitely related to study treatment, Day 3-Year 2 after injection |  | No | No |  |  |
| NCT05225363 | NCT05225363 | Incidence of dose limiting toxicities (DLTs), Rates and associated 90% Clopper and Pearson binomial confidence limits will be estimated., Up to 28 days\|Incidence of adverse events, Adverse Events are graded using NCI CTCAE v.5., Up to 1 year post treatment | Persistence of CAR T cells, Defined as CAR T cells \> 0.1% of total CD3 cells by flow-cytometry., Up to 28 days post treatment\|Expansion of CAR T cells, Max log10 copies/ug of genomic DNA, Up to 1 year post treatment\|Response (iRECIST), Assessed based on Immune-Related Response Criteria. Rates and associated 90% Clopper and Pearson binomial confidence limits will be estimated., Up to 1 year post treatment\|Overall survival (OS), Defined as death from all causes from first treatment date (either lymphodepletion or CAR T cell infusion, as applicable. Kaplan Meier methods will be used to estimate median OS, and graph the results., Up to 1 year post treatment\|Progression-free survival (PFS), Defined as survival without biochemical (CA125) or radiographic evidence of disease progression or relapse from first treatment date (either lymphodepletion or CAR T cell infusion, as applicable). Rates and associated 90% Clopper and Pearson binomial confidence limits will be estimated., Up to 6 months post treatment\|Serum cytokine profile, Serum cytokine profile before and after CAR T cell infusion: to assess potential cytokine release syndrome (CRS) toxicity and CAR T cell effector function, sequential serum samples will be analyzed for Th1/Th2 cytokines (e.g., IL-12, IFNgamma, TNFalpha, IL-10, IL-4, IL-5) by bead array, Up to 1 year post treatment Phenotypes and frequencies of immune cell subsets in the peripheral bloodpre- and post-therapy, Analysis will include CD4:CD8 ratios, differentiation status (CD62L, CD27, CD45 RA/RO), and exhaustion markers (PD1, Tim3, LAG3),trafficking (CCR7, alpha4beta7), proliferation markers (ki67) and effector functions (cytotoxicity, Th1/Th2 cytokines, and CD107a degranulation) on endogenous and CAR+ T cells. Will provide descriptive statistics for exploratory studies. Data will be presented as % of total cells, or as cell/mL blood or peritoneal fluid., Up to 1 year post treatment\|Phenotype of tumor-infiltrating lymphocytes, Will provide descriptive statistics for exploratory studies., Up to 1 year post treatment\|Gene expression, Assessed by ribonucleic acid sequencing (RNA-seq) of circulating tumor cells (CTCs). Will provide descriptive statistics for exploratory studies., Up to 1 year post treatment\|Circulating cell-free deoxyribonucleic acid in peripheral blood, Assessed by whole exome sequencing., Up to 1 year post treatment\|CAR immunogenicity, Assessed based on the presence of anti-TAG72 CAR antibodies or T cell mediated immune responses., Up to 1 year post treatment\|Microbial changes (Stool), Microbial changes in stool associated with CAR T cell therapy, Up to 1 year post treatment | No | No |  |  |
| NCT07435701 | NCT07435701 | Incidence of Adverse Events and Dose-Limiting Toxicities, Number of participants experiencing TEAEs and DLTs. Safety is evaluated using NCI CTCAE v5.0 and ASTCT consensus for CRS., From baseline up to 28 days post-infusion for DLT; safety follow-up through study completion (approx. 1 year)\|RP2D, To determine the optimal dose for the Phase II expansion phase based on the evaluation of DLTs and the overall safety profile observed in the dose-escalation cohorts., Up to approximately 28 days after the first dose of the last participant in the dose-escalation phase. | Ascites Response Rate, Defined as the proportion of participants who require no paracentesis (abdominal drainage) for at least 4 weeks and whose ascites volume (measured by ultrasound as average depth x 10 x abdominal circumference) is controlled at Day 29., At Day 29 (±3 days) post-treatment initiation\|Objective Response Rate (ORR), Defined as the percentage of participants who achieve a Best Overall Response (BOR) of Complete Response (CR) or Partial Response (PR) after intraperitoneal perfusion., Assessed periodically up to approximately 1 year\|Ascites Symptom Improvement Rate, Defined as the proportion of participants whose malignant ascites symptom scale score decreases by ≥30% compared to baseline., Assessed periodically up to approximately 1 year\|Duration of Ascites Relief (DoR), Defined as the time from the first documentation of objective response (CR or PR) to the first documented disease progression or death from any cause., From date of first response until progression or death, assessed up to approx. 1 year\|1-Year Overall Survival (OS) Rate, Defined as the percentage of participants who are alive at 1 year after the initiation of treatment., 1 year post-treatment initiation\|Persistence of SK-NK Cells in Peripheral Blood, Measured by qPCR to detect specific DNA sequences in peripheral blood samples., At Days 1, 8, 15, 22, and 29 during the treatment phase\|Persistence of SK-NK Cells in Ascites, Measured by qPCR to detect specific DNA sequences in ascites samples., At Days 1, 8, 15, 22, and 29 during the treatment phase Changes in Cytokine Levels in Peripheral Blood and Ascites, Evaluation of levels of IL-2, TNF-a, and IFN-y (measured in pg/mL) using flow cytometry., At Baseline, Day 8, Day 15, Day 22, and End of Treatment (Day 29).\|Changes in Immune Cell Subsets, Evaluation of the percentage of Tregs, Macrophages, and MDSCs relative to total CD45+ cells., At Baseline, Day 8, Day 15, Day 22, and End of Treatment (Day 29). | No | No |  |  |
| NCT05922930 | NCT05922930 | Incidence of Adverse Events, Graded According to National Cancer Institute Common Terminology Criteria for Adverse Events (NCI CTCAE) Version (v) 5.0, through study completion; an average of 1 year |  | No | No |  |  |
| NCT05856643 | NCT05856643 | Number of Adverse Events (AEs), To evaluate the safety of SZ011 CAR-NK Cells, Up to 6 months after infusion\|Objective response rate (ORR), To evaluate the ORR of SZ011 CAR-NK Cells, Up to 6 months after infusion\|Overall survival (OS), To determine the anti-tumor effectivity of SZ011 CAR-NK Cells, Up to 6 months after infusion\|Progression-free survival (PFS), To determine the anti-tumor effectivity of SZ011 CAR-NK Cells, Up to 6 months after infusion |  | No | No |  |  |
| NCT07410676 | NCT07410676 | Incidence and severity of treatment-emergent adverse events (TEAEs) and serious adverse events (SAEs), 30 DAYS\|Incidence of Dose-Limiting Toxicities (DLTs), Incidence of Dose-Limiting Toxicities (DLTs), 30 DAYS |  | No | No |  |  |
| NCT05963100 | NCT05963100 | Maximum tolerated dose (MTD) of KT127 cells, The highest dose level (cells/kg) where ≤2/6 evaluable subjects experience protocol-defined dose-limiting toxicities (DLTs) within 14 days post-infusion, with dose escalation terminated based on pre-specified safety criteria., up to second year\|Adverse events (AEs), The ASTCT 2019 standard will be used to grade cytokine release syndrome (CRS) and immune effector cell-associated neurotoxicity syndrome (ICANS), and the CTCAE version 5.0 standard will be used to grade other adverse events. Unless otherwise specified in the protocol, all AEs that occur during the entire study period will be evaluated., up to second year | objective response rate(ORR), The objective response rate evaluation will be based on the researcher's evaluation of imaging, and the efficacy evaluation will refer to the evaluation criteria for solid tumors (RECIST 1.1) The objective response rate evaluation will be based on the researcher's evaluation of imaging, and the efficacy evaluation will refer to the evaluation criteria for solid tumors(RECIST 1.1), up to second year\|progression-free survival(PFS), Progressive free survival is the time from receiving investigational drug treatment to the onset of the first PD or death from any cause in the absence of documented PD, up to second year\|Overall survival(OS), The total survival period is the time between receiving investigational drug treatment and subject death due to various reasons, up to second year | No | No |  |  |
| NCT02118285 | NCT02118285 | Maximum tolerated dose of INCB024360, To determine the maximum tolerated dose (MTD) of INCB024360 when administered with intraperitoneal haploidentical donor NK cells/IL-2 after a nonmyeloablative cyclophosphamide/fludarabine (Cy/Flu) preparative regimen in patients with recurrent ovarian, fallopian tube, and primary peritoneal cancer, 90 days | Initial tumor response, The proportion of patients with initial tumor response (CR or PR), 90 days\|Duration of tumor response, Duration of tumor response, 1 year\|Progression-free survival, Average time to progression., 5 years\|Overall survival, Average length of survival after treatment, 5 years | No | No |  |  |
| NCT03691376 | NCT03691376 | Incidence of adverse events, The frequency of toxicities will be tabulated by grade across all dose levels and cycles., Up to 9 months post infusion\|Maximum tolerated dose (MTD), Dose limiting toxicities will be used in the estimation of the MTD and the accompanying of the dose escalation decisions. The frequency of toxicities will also be tabulated for the dose estimated to be the MTD., Up to 9 months post-infusion | Change in Immunological parameters associated with T cell persistence, Baseline up to 15 years\|Tumor response rates to treatment, Based on the immune-related Response Evaluation Criteria in Solid Tumors criteria., Up to 15 years\|Progression-free survival, Will calculate the median progression free survival and the corresponding 95% confidence interval., From start of the treatment until the first occurrence of confirmed progression, assessed up to 15 years\|Overall survival, From start of the treatment until death, assessed up to 15 years\|Duration of response, Up to 15 years\|Appearance of target antigen/major histocompatibility complex loss variants upon disease recurrence, NY-ESO-1 expression will be evaluated by quantitative reverse transcriptase polymerase chain reaction (Q-RT-PCR) and/or immunohistochemistry. HLA-A\*0201 and -DP\*04 expression on samples will be evaluated by immunohistochemistry., Up to 15 years\|Assessment of bioactivity, measure pre and post treatment percentage of selective migration into the tumor sites, Baseline up to 15 years\|Assessment of Immunological parameters associated with functionality, Baseline up to 15 years | No | No |  |  |
| NCT01583686 | NCT01583686 | Number of Patients With Objective Tumor Regression, Objective tumor regression response was assessed by the Response Evaluation Criteria in Solid Tumors (RECIST)v1.0. Complete Response (CR) is disappearance of all target lesions. Partial Response (PR) is at least a 30% decrease in the sum of the longest diameter (LD) of target lesions taking as reference the baseline sum LD. Stable Disease (SD) is neither sufficient shrinkage to qualify for PR nor sufficient increase to qualify for progressive disease taking as reference the smallest sum LD. Progressive Disease (PD) is at least a 20% increase in the sum of the longest diameter (LD) of target lesions taking as reference the smallest sum LD recorded since the treatment started or the appearance of one or more new lesions., 3.5 mos.\|Number of Participants With Serious and Non-serious Adverse Events Assessed by the Common Terminology Criteria in Adverse Events (CTCAE v4.0), Here is the count of participants with serious and non-serious adverse events assessed by the Common Terminology Criteria in Adverse Events (CTCAE v4.0). A non-serious adverse event is any untoward medical occurrence. A serious adverse event is an adverse event or suspected adverse reaction that results in death, a life-threatening adverse drug experience, hospitalization, disruption of the ability to conduct normal life functions, congenital anomaly/birth defect or important medical events that jeopardize the patient or subject and may require medical or surgical intervention to prevent one of the previous outcomes mentioned., Date treatment consent signed to date off study, approximately 6 months and 17 days for Group A01, 16 months and 13 days for Group A02, 13 months and 3 days for Group A03, 10 months and 16 days for Group A04, and 11 months and 26 days for Group A05. |  | Yes | No |  |  |
| NCT03213964 | NCT03213964 | Maximum Tolerated Doze of FATE-NK100, To determine the maximum tolerated dose/maximum feasible dose (MTD/MFD) of FATE-NK100 when administered via intraperitoneal catheter in patients with recurrent ovarian, fallopian tube, and primary peritoneal cancer., 1 Year | Objective Response Rate, Incidence of objective response rate (ORR) of the FATE-NK100 treatment, Day 28\|Progression-Free Survival (PFS), Incidence of progression-free survival of treated patients 6 months post-infusion, 6 Months\|Overall Survival (OS), Incidence of overall survival of treated patients 6 months post-infusion, 6 Months | No | No |  |  |
| NCT01105650 | NCT01105650 | Response Rate, Response includes Complete Response (CR), Partial Response (PR), and Stable Disease (SD) as defined by Response Evaluation Criteria In Solid Tumors Criteria (RECIST v.1.1) for target lesions and assessed by CT or MRI. Complete Response (CR), disappearance of all target lesions; Partial Response (PR), \>=30% decrease in the sum of the longest diameter of target lesions; Stable Disease (SD), neither sufficient shrinkage to qualify for PR nor sufficient increase to qualify for Progressive Disease., Month 3 | Time to Disease Progression, Time from study entry until progressive disease or data collection cutoff., 1 Year\|Number of Participants With Progressive Disease at One Year, 1 Year\|Overall Survival, Number of participants alive at 1 year., 1 Year | Yes | No |  |  |
| NCT00652899 | NCT00652899 | Number of Patients With In Vivo Expansion of Infused Allogeneic Natural Killer (NK) Cell Product, Detection of an absolute donor derived cell count of \> or = 100 cells/mL after NK cell infusion., Day 12-14 | Number of Patients Per Disease Response, Response Evaluation Criteria in Solid Tumors (RECIST) criteria: Complete Response (CR)-Disappearance of all target lesions (TL); Partial Response (PR)-\< or = 30% decrease in the sum of the longest diameter (LD) of TL, reference baseline sum LD; Stable Disease (SD)-Neither sufficient shrinkage to qualify for PR nor sufficient increase to qualify for PD, reference the smallest sum LD since the treatment started; Progressive Disease (PD)- \< or = 20% increase in the sum of the LD of TL, reference the smallest sum LD recorded since treatment started or appearance of \< or = 1 new lesion., 1 Month After Natural Killer Cell Infusion (Day 30)\|Median Number of Days to Progression, Median number of days from first date of treatment to date of disease progression (appearance of new metastatic lesions or objective tumor progression). Defined by computated tomography (CT) imaging based on Response Evaluation Criteria In Solid Tumors (RECIST): Progressive Disease (PD) \> or = 20% increase in sum of all target or any new lesions., From date of first treatment to disease progression\|Median Overall Survival Number of Days Patients Alive After Treatment, Median number of days patients alive from date of treatment to date of death or date of last follow-up if censored., From first date on-study (treatment) to date of death | Yes | No |  |  |
| NCT06321484 | NCT06321484 | Maximum Tolerated Dose (MTD) (Cohort 1), The MTD of the use of cytokine induced memory-like natural killer (CIML NK) cell therapy is determined by the number of participants who experience a dose limiting toxicity (DLT). See subsequent primary outcome measure for DLT definition., 60 days\|Dose Limiting Toxicity (DLT) (Cohort 1), A DLT is defined as an adverse event that is related to CIML NK cell therapy with an attribution of possible, probable, or definite, and meets the criteria defined in protocol section 5.4., 60 days | Overall Response Rate (ORR), ORR is defined as the proportion of participants who achieved partial response or complete response during study treatment based on RECIST 1.1 and immune-related RECIST criteria., Up to 5 years\|Median Progression Free Survival (PFS), PFS is defined as the time from registration to the earlier of progression (PD) or death due to any cause based on Kaplan-Meier methodology. Participants alive without disease progression are censored at date of last disease evaluation., Up to 5 years\|Clinical Benefit Rate (CBR), CBR is defined as the proportion of participants who achieved a complete response, partial response, or had stable disease for 6 months or more based on RECIST 1.1 and immune-related RECIST criteria., Up to 5 years\|Duration of Response (DOR), DOR is measured from the time measurement criteria are met for complete response or partial response (whichever is first recorded) until the first data that recurrent or progressive disease is objectively documented (taking as reference for progressive disease the smallest measurements recorded since the treatment started, or death due to any cause. Participants without events reported are censored at the last disease evaluation., Up to 5 years\|6 months Progression Free Survival (PFS6), PFS6 is the percent probability estimate at 6 months based on the Kaplan-Meier method. PFS is defined as the time from registration to the earlier of progression (PD) or death due to any cause. Participants alive without disease progression are censored at date of last disease evaluation., 6 months | No | No |  |  |
| NCT07651124 | NCT07651124 | Evaluation of cytotoxicity of cells against cancer cells, * Primary cancer cells isolated and cultured from a single cell of the same patient's tumor, or TIL cells cultured from the ovarian cancer cell line OVCAR3, are co-cultured in a CO2 incubator for 20 hours. After 20 hours, the cells are stained with 7AAD, and the cancer cell killing ability is analyzed using a flow cytometer * Measurement of IFN-γ secreted by T cells: CD8+ T cells inhibit tumor cell differentiation and enhance immune function by secreting IFN-γ. After co-culturing target cells and T cells, the pellet is used for cytotoxicity evaluation, and the culture medium is collected to measure the amount of secreted IFN-γ using ELISA. * Microscopic assay: After co-culturing fluorescently labeled primary cancer cells with fluorescently labeled enhanced T cells for 20 hours, the number of viable tumor cells is measured., Treatment period- 2 months, follow-up- 4 months after completion of treatment\|Evaluation of toxicity of protocol therapy including lymphodepletion, CHA-TIL, and high dose IL2, * Toxicity evaluation variables subject to analysis include adverse events, clinical laboratory test results (e.g., hematology, coagulation, serum analysis, and urinalysis), physical examination, and vital signs. Clinically significant changes from baseline will be summarized using descriptive statistics. The severity of adverse events will be graded according to CTCAE v5.0 and recorded in the case report form. * The overall frequency of adverse events (number of subjects and percentage), the worst reported severity, and the association with the investigational medicinal product will be recorded for each subject. * Serious adverse events are summarized in a similar manner. A list of deaths, SAEs, and AEs that lead to early termination or withdrawal from the clinical trial will also be provided. * For all AEs, a complete list will be presented including the subject number, clinical trial regimen, severity, severity, actions taken, outcome, association with the clinical trial treatment,, Treatment periods 2 months, follow-up 4 months after completion of treatment |  | No | No |  |  |
| NCT05518253 | NCT05518253 | Incidence of Adverse events after CD70 CAR-T cells infusion [Safety and Tolerability], Therapy-related adverse events were recorded and assessed according to the National Cancer Institute's Common Terminology Criteria for Adverse Events (CTCAE, Version 5.0), 28 days\|Obtain the maximum tolerated dose of CD70 CAR-T cells[Safety and Tolerability], Dose-limiting toxicity after cell infusion, 28 days | Disease control rate of CAR-T cell preparations in CD70 positive advanced malignancies [Effectiveness], Disease control rate: including CR, PR and SD, 3 months\|Objective response rate (ORR) of CD70 CAR-T treatment in patients with CD70-positive advanced malignancies[Effectiveness], Objective response rate includes：CR、PR, 3 months\|Duration of Response (DOR) of CD70 CAR-T treatment in patients with CD70-positive advanced malignancies[Effectiveness], DOR will be assessed from the first assessment of CR/PR/SD to the first assessment of recurrence or progression of the disease or death from any cause, 3 months\|AUCS of CD70 CAR-T cells [Cell dynamics], AUCS is defined as the area under the curve in 28 days and 90 days, 3 months\|CMAX of CD70 CAR-T cells [Cell dynamics], CMAX is defined as the highest concentration of CD70 CAR-T cells expanded in peripheral blood, 3 months\|TMAX of CD70 CAR-T cells[Cell dynamics], TMAX is defined as the time to reach the highest concentration, 3 months\|Pharmacodynamics of CD70 CAR-T cells[Cell dynamics], Concentration levels of CAR-T-related serum cytokines such as CRP, IL-6, ferritin at each time point, 3 months The correlation between CD70 positive rate and safety, assessment the correlation between CD70 positive rate and the incidence of CRA and ICANS, 1 years\|Correlation between CD70 positive rate and efficacy, assessment the correlation between CD70 positive rate and the disease control rate，Disease control rate: including CR, PR and SD, 1 years\|Overall survival(OS)of CD70 CAR-T treatment in patients with CD70-positive advanced malignancies[Effectiveness], OS will be assessed from the first CD70-CAR-T cell infusion to death from any cause (Assessed by investigators based on IRECIST criteria), 1 years\|Progress-free survival(PFS) of CD70 CAR-T treatment in patients with CD70-positive advanced malignancies[Effectiveness], PFS will be assessed from the first CD70-CAR-T cell infusion to death from any cause or the first assessment of progression (Assessed by investigators based on IRECIST criteria), 1 years\|Duration of Response (DOR) of CD70 CAR-T treatment in patients with CD70-positive advanced malignancies[Effectiveness], DOR will be assessed from the first assessment of CR/PR/SD to the first assessment of recurrence or progression of the disease or death from any cause (Assessed by investigators based on IRECIST criteria), 1 years | No | No |  |  |
| NCT05420545 | NCT05420545 | Incidence of Adverse events after CD70 CAR-T cells infusion [Safety and Tolerability], Therapy-related adverse events were recorded and assessed according to the National Cancer Institute's Common Terminology Criteria for Adverse Events (CTCAE, Version 5.0), 28 days\|Obtain the maximum tolerated dose of CD70 CAR-T cells[Safety and Tolerability], Dose-limiting toxicity after cell infusion, 28 days | Disease control rate of CAR-T cell preparations in CD70 positive advanced malignancies [Effectiveness], Disease control rate: The proportion of subjects who achieved CR, PR, SD after treatment accounted for all treated subjects (Assessed based on RECIST criteria)，the minimum value is 0%，maximum value is 100%, and higher scores mean a better outcome., 3 months\|Objective response rate (ORR) of CD70 CAR-T treatment in patients with CD70-positive advanced malignancies[Effectiveness], Objective response rate includes：The proportion of subjects who achieved CR, PR after treatment accounted for all treated subjects (Assessed based on RECIST criteria)，the minimum value is 0%，maximum value is 100%, and higher scores mean a better outcome., 3 months\|Duration of Response (DOR) of CD70 CAR-T treatment in patients with CD70-positive advanced malignancies[Effectiveness], DOR will be assessed from the first assessment of CR/PR/SD to the first assessment of recurrence or progression of the disease or death from any cause, 3 months\|Overall survival(OS)of CD70 CAR-T treatment in patients with CD70-positive advanced malignancies[Effectiveness], OS will be assessed from the first CD70-CAR-T cell infusion to death from any cause (Assessed based on RECIST criteria), 2 years\|Progress-free survival(PFS) of CD70 CAR-T treatment in patients with CD70-positive advanced malignancies[Effectiveness], PFS will be assessed from the first CD70-CAR-T cell infusion to death from any cause or the first assessment of progression(Assessed based on RECIST criteria), 2 years\|AUCS of CD70 CAR-T cells [Cell dynamics], AUCS is defined as the area under the curve in 90 days, 3 months\|CMAX of CD70 CAR-T cells [Cell dynamics], CMAX is defined as the highest concentration of CD70 CAR-T cells expanded in peripheral blood, 3 months\|TMAX of CD70 CAR-T cells[Cell dynamics], TMAX is defined as the time to reach the highest concentration, 3 months\|Pharmacodynamics of CD70 CAR-T cells[Cell dynamics], Concentration levels of CAR-T-related serum cytokines such as CRP, IL-6, ferritin at each time point, 3 months The correlation between CD70 positive rate and safety, assessment the correlation between CD70 positive rate and the incidence of CRA and ICANS, 2 years\|Correlation between CD70 positive rate and efficacy, assessment the correlation between CD70 positive rate and the disease control rate，Disease control rate: including CR, PR and SD, 2 years\|Overall survival(OS)of CD70 CAR-T treatment in patients with CD70-positive advanced malignancies[Effectiveness], OS will be assessed from the first CD70-CAR-T cell infusion to death from any cause (Assessed by investigators based on IRECIST criteria), 2 years\|Progress-free survival(PFS) of CD70 CAR-T treatment in patients with CD70-positive advanced malignancies[Effectiveness], PFS will be assessed from the first CD70-CAR-T cell infusion to death from any cause or the first assessment of progression (Assessed by investigators based on IRECIST criteria), 2 years\|Duration of Response (DOR) of CD70 CAR-T treatment in patients with CD70-positive advanced malignancies[Effectiveness], DOR will be assessed from the first assessment of CR/PR/SD to the first assessment of recurrence or progression of the disease or death from any cause (Assessed by investigators based on IRECIST criteria), 2 years | No | No |  |  |
| NCT03539406 | NCT03539406 | Incidence of treatment emergent adverse events, Incidence of treatment emergent adverse events (following CTCAE criteria), 6 months | in vivo lifespan of the infused UCB-NK cells, determination of NK cell percentage in blood and peritoneal fluid, 28 days\|in vivo expansion of the infused UCB-NK cells, determination of NK cell percentage in peritoneal fluid and blood, 28 days\|Measurement of in vitro cytolytic activity of infused NK cells, in CFSE base killing assays a percentage of dead cells (K562 cells) will be measured., 28 days\|the effect of NK cell infusion on measurable disease, CA-125 testing in blood (in E/mL), 6 months\|the effect of NK cell infusion on measurable disease, CT-scan (measurement of visible leasions in cm), 6 months | No | No |  |  |
| NCT07613723 | NCT07613723 | Safety and tolerability of ZI-MA4-1, Assessed using clinical assessments and adverse event reporting, including dose-limiting toxicities (DLTs), treatment-emergent adverse events (TEAEs), and treatment-related adverse events (TRAEs), From baseline through end of study visit (up to 5 years) | MTD and RP2D of ZI-MA4-1, Identification of maximum tolerated dose (MTD) and the recommended Phase 2 dose (RP2D) of ZI-MA4-1 based on observed DLTs and predefined dose-escalation rules, Through completion of study response follow-up (up to 2 years)\|Long term safety, Evaluate ZI-MA4-1 for the occurrence of delayed adverse events (AEs), Through end of study visit (up to 5 years)\|Minimum biologically active dose (MBAD) of ZI-MA4-1, Assessment of preliminary anti-tumour activity, Through completion of study response follow-up (up to 2 years)\|Objective Response Rate (ORR), Assessed by RECIST 1.1, Through completion of study response follow-up (up to 2 years)\|Best Overall Response (BOR), Assessed by RECIST 1.1, Through completion of study response follow-up (up to 2 years)\|Disease Control Rate (DCR), Assessed by RECIST 1.1, Through completion of study response follow-up (up to 2 years)\|Pharmacokinetics of ZI-MA4-1, Evaluation of persistence of ZI-MA4-1 cells in the blood using vector copy number (VCN) analysis, 3 years post-infusion of ZI-MA4-1 | No | No |  |  |
| NCT03356795 | NCT03356795 | Safety of CART cells in patients using CTCAE version 4.0 standard to evaluate the level of adverse events, Physiological parameter (measuring cytokine response), 3 months | Persistence and proliferation of CART cells in patients, The expansion and functional persistence of CART cells in the peripheral blood of patients will be measured by qPCR on Day 7, 14, 21, 28, 60 and 90 after infusion., 3 months\|Anti-tumor effects, Objective response, such as complete response (CR), partial response (PR), stable disease (SD), or progressive disease (PD) will be assessed by the Response Evaluation Criteria in Solid Tumors (RECIST) v1.1 criteria., 1 year | No | No |  |  |
| NCT03585764 | NCT03585764 | Number of Study Subjects With Treatment-related Adverse Events Using NCI Common Terminology Criteria for Adverse Events (CTCAE) v5.0, For this study, collection of adverse events will begin at the time of apheresis and will continue until the subject is off-study., Up to 34 months\|Manufacturing Feasibility, Manufacturing feasibility is determined based on the "manufacturing failures" products. The number of manufactured products that do not meet release criteria for vector transduction efficiency, CART+ cell number, T cell purity, viability, and sterility will be determined and defined as "manufacturing failures"., 8 weeks\|Clinical Feasibility, Clinical feasibility is defined as the proportion of subjects enrolled on this protocol who do not receive MOv19-BBz CAR T cells. Reasons for this occurrence include rapid clinical deterioration or death, and subject withdrawal., 8 weeks | Progression-free Survival (PFS), PFS will be evaluated up to 5 years post-infusion or until subjects initiate a cancer-related therapy, Up to 33 months\|Overall Response Rates (ORR), ORR is the proportion of subjects with a best response of CR or PR based on RECIST 1.1, as compared to baseline. ORR will be evaluated up to 5 years post-infusion or until subjects initiate a cancer-related therapy., Up to 33 months\|Overall Survival (OS), Overall survival is defined as the time from the date of the infusion to the date of death due to any reason. OS will be evaluated up to 15 years post-infusion., Up to 33 months | Yes | No |  |  |
| NCT02580747 | NCT02580747 | Occurrence of Study related adverse events, defined as \>= Grade 3 signs/symptoms, laboratory toxicities, and clinical, Until week 24 | Anti-tumor responses to CART-meso cell infusions, up to 24 weeks In vivo existence of CART-meso, 1 year | No | No |  |  |
| NCT07096583 | NCT07096583 | The remission rate of bone marrow suppression, The objective of the study was to evaluate the efficacy and safety of NK cell adjuvant therapy in alleviating chemotherapy-induced myelosuppression in patients with ovarian cancer. The primary endpoints included the time to neutrophil recovery, the improvement rate of platelet count, and the incidence of infectious events, 3-month | Adverse event, Any adverse medical event that occurred after the subject received the trial drug, 12-month | No | No |  |  |
| NCT03634501 | NCT03634501 | Incidence of toxicity induced by NK infusion, The incidence of toxicity within each infusion within 6 months after NK administration, 6 months | Objective-response rate (ORR), Objective-response rate (ORR): defined as the proportion of patients who achieve partial response (PR) or complete response (CR) per Response Evaluation Criteria in Solid Tumors (RECIST) 1.1 at any time on study., 6 months after 6 infusions of NK administration\|Persistence of NK, Duration of NK persistence: defined as duration from Day 1 to the time when the NK level goes back to stable level, Day 0, Day 7, Day30, Day 60, Day 90, Day 180 | No | No |  |  |
| NCT05372692 | NCT05372692 | objective remission rate, objective remission rate, 4-6weeks |  | No | No |  |  |
| NCT03916679 | NCT03916679 | Safety measured by occurrence of study related adverse effects defined by NCI CTCAE 4.0, 12 months post infusion | Overall complete remission rate defined by the standard response criteria for malignant lymphoma for each arm, 12 months post infusion\|Progress Free Survival (PFS) after administration, 12 months post infusion\|Duration of CAR-positive T cells in circulation, 12 months post infusion\|Detection of PD1 antibody in serum, 12 months post infusion | No | No |  |  |
| NCT03735589 | NCT03735589 | Incidence of adverse events as assessed by Cancer Therapy Evaluation Program (CTEP) version 4 of the Common Terminology Criteria for Adverse Events (CTCAE), Up to 12 months\|Dose-limiting toxicities (DLT) assessed by CTCAE version 5, Will be used in the estimation of the maximum tolerated dose (MTD) and the accompanying of the dose escalation decisions. However, no formal analyses of DLTs are planned., Up to 14 days after intraperitoneal (IP) infusion of nCTLs\|Change in immune response, Change in immune response will be measured by the increase in the number of CD3+CD8+NKG2D (high) natural killer cell-like cytotoxic T-lymphocyte (CTLs) (nCTLs) and the increase in total CD3+CD8+ CTLs recovered in the peritoneal washes with evaluation on day 0 versus day 2. The analysis will consist of an analysis-of-covariance (ANCOVA) for the outcome of post-pre ACT treatment cell count with a factor for dose and sampling time 48 hrs +/- 24 hrs., From baseline (day 0) to day 2 (48 hours after adoptive cell therapy [ACT]) administration\|Persistence of nCTLs after their adoptive transfer, At day 0 will obtain peritoneal material (outflow and washes) directly before i.p. infusion of nCTLs, as well as day 2 (48 hours +/- 24 hours), day 7 (+/- 2 days), 2 weeks (14 days +/- 2 days) and 4 weeks (28 days +/- 3 days) later., Up to 4 weeks | T cell populations and higher anti-tumor responses, Will estimate the half-life of CD3/CD8/NKG2D (triple-positive nCTLs) cell counts and longitudinal changes CD3/CD8 cell counts (double-positive CTLs, which include both nCTLs and additional CTLs newly recruited CTLs from the circulation) cell counts as a function of dose and time. A nonlinear regression model with a random effect for subject (population pharmacokinetics \[PK\]) will be fit to a one-compartment kinetics first-order absorption model per dose level., Up to 4 weeks Progression-free survival assessed by immune-related response criteria (irRECIST), Will examine time-to-disease progression graphically via the generation of Kaplan-Meier survival curves., Up to 12 months\|Overall survival assessed by irRECIST, Will examine time-to-disease progression graphically via the generation of Kaplan-Meier survival curves., Up to 12 months | No | No |  |  |
| NCT06469281 | NCT06469281 | Incidence of treatment emergent adverse events (TEAEs), Part 1a, Up to 18 months\|Incidence of adverse events of special interest (AESIs), Part 1a, Up to 18 months\|Incidence of adverse events of dose limiting toxicities (DLTs), Part 1a, Up to 18 months\|Manufacturing feasibility of 27T51, Phase 1a/1b Determination of the feasibility of manufacturing 27T51 is measured by the percent of leukapheresis products collected that are able to be manufactured and released for infusion., Up to 3 years\|Overall response rate (ORR) as assessed by the investigator, Phase 1b, Up to 48 months | ORR as assessed by the investigator, Phase 1a, Up to 48 months\|Duration of response (DoR), Phase 1a/1b, Up to 48 months\|Disease control rate (DCR), Phase 1a/1b, Up to 48 months\|Incidence of TEAEs, Phase 1b, Up to 48 months\|Incidence of AESIs, Phase 1b, Up to 48 months\|Incidence of DLTs, Phase 1b - Arms B and C, Up to 48 months | No | No |  |  |
| NCT06395844 | NCT06395844 | R0 resection rate, the percentage of patients received R0 resection after METR-NK cells as neoadjuvant therapy for advanced epithelial ovarian cancer, 3-month | Overall Response Rate (ORR) After Neoadjuvant treatment, Overall Response Rate according to RECIST1.1 after Neoadjuvant treatment. ORR is defined as the proportion of participants achieving Complete Response (CR) or Partial Response (PR) as assessed by the investigator per RECIST (v.1.1). Per RECIST 1.1, CR is defined as the disappearance of all target lesions; PR is defined as at least a 30% decrease in the sum of diameters of target lesions., 3-month\|Pathological complete remission rate, No residual invasive carcinoma was found under the microscope, but ductal carcinoma in situ could be found; In addition, compare the amount of cancer cells in the specimen before and after the new adjuvant therapy, and use of the Miller Payne grading system to evaluate the effect of the new adjuvant therapy, 3-month\|Disease control rate, the percentage of patients with complete response, partial response, and stable disease for more than 4 weeks in which response can be evaluated, 3-month\|Progression free survival, the date of enrollment to the date of the first onset of disease progression or death from any cause, whichever comes first, 2-years\|Survival rate, the date of enrollment to the date of death from any cause, 3-years\|Adverse event, Adverse medical events in clinical trial subjects treated with METR-NK cells, 3-month | No | No |  |  |
| NCT00101257 | NCT00101257 | Safety and toxicity\|Duration of in vivo persistence\|Antitumor effects |  | No | No |  |  |
| NCT03799913 | NCT03799913 | Safety measured by occurrence of study related adverse effects defined by NCI CTCAE 4.0, 1 years post infusion | Overall complete remission rate defined by the standard response criteria for malignant lymphoma for each arm, 12 months post infusion\|Progress Free Survival (PFS) after administration, 12 months post infusion\|Duration of CAR-positive T cells in circulation, 12 months post infusion\|Detection of PD1 antibody in serum, 12 months post infusion | No | No |  |  |
| NCT03054298 | NCT03054298 | Number of Participants With Treatment-related Adverse Events as Assessed by CTCAE v4.03, 7 years | Progression-free Survival, Progression is defined using RECIST V1.1 - at least a 20% increase in the sum of diameters of target lesions, taking as reference the smallest sum on study (this includes the baseline sum if that is the smallest on study). In addition to the relative increase of 20%, the sum must also demonstrate an absolute increase of at least 5 mm. Note: the appearance of one or more new lesions is also considered progression., 7 years\|Overall Survival, 7 years\|Objective Response Rate, Objective response rate is the proportion of subjects in the efficacy-evaluable set with radiologically confirmed measurable disease at baseline, who achieved partial response (PR) or better after infusion as determined by RECIST 1.1. Missing or non-evaluable time points will not be included. Per RECIST 1.1, a complete response is defined as "disappearance of all target lesions. Any pathological lymph nodes (whether target or non-target) must have reduction in short axis to \<10 mm." A partial response is defined as "at least a 30% decrease in the sum of diameters of target lesions, taking as reference the baseline sum diameters.", Month 6 | Yes | No |  |  |
| NCT06884345 | NCT06884345 | R0 resection rate, the percentage of patients received R0 resection after METR-NK cells as neoadjuvant therapy for advanced epithelial ovarian cancer, 3-month | Overall Response Rate (ORR) After Neoadjuvant treatment, 3-month\|Pathological complete remission rate, 3-month\|Disease control rate, 3-month\|Progression free survival, 2-years\|Survival rate, 3-years\|Adverse event, 3-month | No | No |  |  |
| NCT03814447 | NCT03814447 | Adverse events (AEs) and Serious adverse event (SAEs), Safety measured by occurrence of study related adverse effects defined by NCI CTCAE 4.03, 1 year post infusion | Cmax, the highest concentration (Cmax) of anti-human MESO T cells in the peripheral blood after administration, 30 days post infusion\|Tmax, the time to reach the highest concentration (Tmax) of anti-human MESO T cells in the peripheral blood after administration, 30 days post infusion\|AUC(0-30d), the area under the curve of 30 days of anti-human MESO T cells in the peripheral blood after administration, 30 days post infusion\|Duration of Mesothelin-positive T cells in circulation, Duration of Mesothelin-positive T cells in circulation, 90 days post infusion\|ORR, Overall response rate after administration, 3 months post infusion\|PFS, Progress Free Survival after administration, 1 year post infusion\|EORTC Quality-of-Life Questionnaire Core 15 Palliative Care (QLQ-C15-PAL) of patients after administration, This QLQ-C15-PAL score consists of 15 questions; two multi-item functional scales (physical and emotional functioning), two multi-item symptom scales (fatigue and pain) together with five single-item symptom scales (nausea/vomiting, dyspnea, insomnia, appetite loss, constipation) and one final question referring to overall QOL. The physical functioning scale is based on three questions regarding walking, activities of daily living and time spent in bed or in a chair. The emotional functioning scale is based on two questions that ask about feeling tense or depressed. Patients rated each question on a Likert scale from 1 (not at all) to 4 (very much), with the exception of overall QOL, which was rated from 1 (very poor) to 7 (excellent), 1 year post infusion | No | No |  |  |
| NCT02280811 | NCT02280811 | Maximum Tolerated Dose (MTD), The MTD is the highest dose at which ≤1 of 6 patients experienced a dose limiting toxicity (DLT) or the highest dose level studied if DLTs are not observed at any of the dose levels., participants were followed for the duration of hospital stay, an average of 3 weeks\|Objective Tumor Response Rate (Complete or Partial Response), Objective tumor response rate is defined as the number of participants with a complete or partial response per the Response Evaluation Criteria in Solid Tumors (RECIST) v1.0. Complete response is disappearance of all target lesions. Partial response is at least a 30% decrease in the sum of the longest diameter (LD) of target lesions, taking as reference the baseline sum LD., 4 years\|Duration of Response, Duration of response is measured from the time measurement criteria are met for complete response or partial response (whichever is first recorded) until the first date that recurrent or progressive disease is objectively documented (taking as reference for progressive disease the smallest measurements recorded since the treatment started). Response is assessed by the Response Evaluation Criteria in Solid Tumors (RECIST) v1.0. Complete response is disappearance of all target lesions. Partial response is at least a 30% decrease in the sum of the longest diameter (LD) of target lesions, taking as reference the baseline sum LD. Progression is at least a 20% increase in the sum of target lesions, taking as reference the smallest sum LD recorded since the treatment started or the appearance of one or more new lesions., up to one year | Number of Participants With Serious and Non-serious Adverse Events, Here is the number of participants with serious and non-serious adverse events assessed by the Common Terminology Criteria in Adverse Events (CTCAE v3.0). A non-serious adverse event is any untoward medical occurrence. A serious adverse event is an adverse event or suspected adverse reaction that results in death, a life threatening adverse drug experience, hospitalization, disruption of the ability to conduct normal life functions, congenital anomaly/birth defect or important medical events that jeopardize the patient or subject and may require medical or surgical intervention to prevent one of the previous outcomes mentioned., 19 months and 7 days\|Number of Participants With a Dose Limiting Toxicity (DLT), A dose limiting toxicity is all Grade 3 and greater toxicities with the exception of myelosuppression, defined as lymphopenia, neutropenia, decreased hemoglobin, and thrombocytopenia, due to chemotherapy preparative regimen. Aldesleukin expected toxicities as defined in Appendix 2 and 3 of the protocol. Expected chemotherapy toxicities as defined in the pharmaceutical information section. Immediate hypersensitivity reactions (excluding symptomatic bronchospasm and grade 4 hypotension) occurring within 2 hours of cell infusion (related to cell infusion) that are reversible to a grade 2 or less within 24 hours of cell administration with standard therapy. Grade 3 fever. Events that are clearly related to the patient's disease., 19 months and 7 days\|Percentage of Cluster of Differentiation 3 (CD3+) Cells That Are E6 T-Cell Receptor Memory of Circulating T-Cells in Responders and Non-responders, Detection of E6 TCR T cells in patients peripheral blood leukocytes (PBL)/apheresis samples by flow cytometry., One month after treatment\|Expression of Programmed Cell Death 1 (PD-1) by Circulating E6 T-Cell Receptor (TCR) T-Cells, Presence of PD-1 on circulating lymphocytes by flow cytometry one month after treatment., one month after treatment | Yes | No |  |  |
| NCT00019084 | NCT00019084 |  |  | No | No |  |  |
| NCT03412877 | NCT03412877 | Response rate, Percentage of patients who receive pembrolizumab as part of the treatment regimen that have a clinical response to treatment (objective tumor regression), 6 and 12 weeks after cell infusion, then every 3 months x3, then every 6 months x 2 years, then per PI discretion | Safety and tolerance, Using standard CTCAE 5.0, 6 weeks ( 2 weeks) following administration of the cell product or 30 days following the last dose of pembrolizumab | No | No |  |  |
| NCT02713984 | NCT02713984 | Determine the toxicity profile of the HER2 targeted CAR T cells with Common Toxicity Criteria for Adverse Effects (CTCAE) version 4.0., Observe and handle the toxicity profile of the HER2 targeted CAR T cells with Common Toxicity Criteria for Adverse Effects (CTCAE) version 4.0., 3 years | Survival time of Anti-HER2 CAR T cells in vivo., Detect the existence of CAR-T cells in the blood of participants through qPCR or flow cytometry., 1 year\|Efficacy of anti-HER2 CAR T cells to confirm the ability of CAR T cells to eliminate HER2 positive cancer cells, Evaluate the anti-tumor efficacy of CAR-T therapy by Response Evaluation Criteria In Solid Tumors (RECIST) v1.1 and Immune-Related Response Criteria（iRECIST）., 12 weeks\|Maximum tolerated dose (MTD) of HER2 targeted CAR T cells, Determine the maximum tolerated dose of each participant through the grades of side effects., 4 weeks | No | No |  |  |
| NCT06284226 | NCT06284226 | To evaluate the safety and the efficacy, To evaluate the safety and objective remission rate of multiple injections of allogeneic natural killer cells (complete remission \[CR\] + partial remission \[PR\]), 12 months |  | No | No |  |  |
| NCT02457650 | NCT02457650 | Number of participants with Adverse Events, To evaluate the safety and feasibility of the administration of anti-NY-ESO-1 TCR transduced T cells in patients with HLA-A2+ NY-ESO-1-expressing malignancies., 8 weeks | Number of participants with Clinical responses, To determine if the treatment can result in clinical regression of malignant tumors in the patients., 2 years | No | No |  |  |
| NCT06885697 | NCT06885697 | Establish the recommended phase 2 dose (RP2D) of TNhYP218 CAR T cells based on dose-limiting toxicity (DLT) of defined adverse events (AEs)., The highest dose level below the maximum administered dose at which no more than 1 of 6 participants experience DLT from the initiation of CAR-T cell infusion (day 0) through day 28 after infusion (day 28)., DLT assessment will occur in participants in the dose escalation cohort daily on days 0-4, on day 7, on day 21 and during week 4.\|Determine the preliminary objective response rate of TNhYP218 CAR T cells in a limited number of participants with mesothelioma treated at the recommended phase 2 dose., The proportion of mesothelioma participants with partial response or complete response at the recommended phase 2 dose., assessed based on imaging studies at weeks 4, 8, 12 then every 12 weeks through disease progression or week 108, whichever occurs first. | Near term safety of autologous genetically modified TNhYP218 CAR T cells in adult study participants with mesothelin expressing unresectable, metastatic, or recurrent mesothelioma and other mesothelin expressing solid tumors., The near-term safety of the study therapy will be evaluated by reporting the grade and type of toxicity at each dose level., Assessments will occur daily during lymphodepletion, on day of cell infusion (D0), D10, D21, every 2 weeks from W4 to W8, then every 4 weeks through W 24 or disease progression.\|Long term safety of autologous genetically modified TNhYP218 CAR T cells in adult study participants with mesothelin expressing unresectable, metastatic, or recurrent mesothelioma and other mesothelin expressing solid tumors., The long-term safety will be assessed by the presence of RCL as well as clinical assessments., Monitored at baseline, weeks 4, 12, 24 and 48 after cell infusion and annually thereafter for up to 15 years if positive during year 1 or warranted based on clinical history collected annually during15 year follow-up.\|Determine the objective response rate of TNhYP218 CAR T cells in participants with mesothelin expressing solid tumors treated at doses other than the RP2D, The proportion of participants with partial response or complete response at doses other than the recommended phase 2 dose., assessed based on imaging studies at weeks 4, 8, 12 then every 12 weeks through disease progression or week 108, whichever occurs first.\|Determine progression free survival in participants with mesothelin expressing solid tumors, The duration of time from start of treatment (lymphodepletion) to time of progression or death, whichever occurs first., Assessed based on imaging studies at weeks 4, 8, 12 then every 12 weeks through disease progression or week 108, whichever occurs first.\|Determine duration of response in participants with mesothelin expressing solid tumors, The duration of overall response is measured from the time measurement criteria are met for CR or PR (whichever is first recorded) until the first date that recurrent or progressive disease is objectively documented (taking as reference for progressive disease the smallest measurements recorded since the treatment started)., Assessed based on imaging studies at weeks 4, 8, 12 then every 12 weeks through disease progression or week 108, whichever occurs first.\|Determine overall survival in participants with mesothelin expressing solid tumors, The duration of time from start of treatment (lymphodepletion) to time of death from any cause., Assessed from physical exams performed on D10, D21, W4, W6, W8, then every 4 weeks through W 24, and with semiannual remote assessments following active follow up through year 5.\|Evaluate feasibility of manufacturing TNhYP218 CAR T cells from participants with mesothelin expressing solid tumors., The fraction of participants with cell products produced with Tna(SqrRoot) ve/scm cells, Assessed prior to cell infusion (Day 0).\|Assess the tolerability of lymphodepletion followed by TNhYP218 CAR T-cell infusion in participants with mesothelin expressing solid tumors, -The fraction of participants able to receive all protocol therapy without treatment delay.-The fraction of participants able to receive cell product without interruption due to infusion related reactions., Assessed through cell infusion (Day 0) | No | No |  |  |
| NCT05592626 | NCT05592626 | Phase 1 (Dose Escalation):Number of Participants with Dose-limiting Toxicities (DLTs) in Cycle 1, Cycle 1 (Cycle length= 28 days)\|Phase 1 and 2 (Dose Escalation and Expansion): Number of Participants with Adverse Events (AEs) and Serious Adverse Events (SAEs), Up to 3 years\|Phase 2 (Dose Expansion): Percentage of Participants with Overall Objective Tumor Responses (ORR), Complete response (CR) and partial response (PR), Up to 3 years | Phase 1 and 2 (Dose Escalation and Expansion): Percentage of Participants with ORR, Up to 3 years\|Phase 1 and 2 (Dose Escalation and Expansion): Duration of Responses (DOR), Up to 3 years\|Phase 1 and 2 (Dose Escalation and Expansion): Percentage of Participants with Disease Control (CR, PR, and Stable Disease), Up to 3 years\|Phase 2 (Dose Expansion): Progression Free Survival (PFS), Up to 3 years\|Phase 2 (Dose Expansion): Overall Survival (OS), Up to 3 years\|Phase 1 and 2 (Dose Escalation and Expansion): Maximum Observed Plasma Concentration (Cmax) for STAR0602, Dose Escalation: Cycle 1 and Cycle 6 at predefined intervals up to 1 year; Dose Expansion: Cycle 1, Cycle 3, and Cycle 6 at predefined intervals up to 3 years (Cycle length= 28 days)\|Phase 1 and 2 (Dose Escalation and Expansion): Time (Tmax) to Reach the Maximum Plasma Concentration (Cmax) for STAR0602, Dose Escalation: Cycle 1 and Cycle 6 at predefined intervals up to 1 year; Dose Expansion: Cycle 1, Cycle 3, and Cycle 6 at predefined intervals up to 3 years (Cycle length= 28 days)\|Phase 1 and 2 (Dose Escalation and Expansion): Area Under the Plasma Concentration (AUC) Versus Time Curve for STAR0602, Dose Escalation: Cycle 1 and Cycle 6 at predefined intervals up to 1 year; Dose Expansion: Cycle 1, Cycle 3, and Cycle 6 at predefined intervals up to 3 years (Cycle length= 28 days)\|Phase 1 and 2 (Dose Escalation and Expansion): Terminal Elimination Half-life (t1/2) for STAR0602, Dose Escalation: Cycle 1 and Cycle 6 at predefined intervals up to 1 year; Dose Expansion: Cycle 1, Cycle 3, and Cycle 6 at predefined intervals up to 3 years (Cycle length= 28 days)\|Phase 1 and 2 (Dose Escalation and Expansion): Apparent Total Body Clearance (CL) for STAR0602, Dose Escalation: Cycle 1 and Cycle 6 at predefined intervals up to 1 year; Dose Expansion: Cycle 1, Cycle 3, and Cycle 6 at predefined intervals up to 3 years (Cycle length= 28 days)\|Phase 1 and 2 (Dose Escalation and Expansion): Apparent Volume of Distribution (Vd) for STAR0602, Dose Escalation: Cycle 1 and Cycle 6 at predefined intervals up to 1 year; Dose Expansion: Cycle 1, Cycle 3, and Cycle 6 at predefined intervals up to 3 years (Cycle length= 28 days)\|Phase 1 and 2 (Dose Escalation and Expansion): Anti-drug Antibody (ADA) formation, Dose Escalation and Expansion: Day 1 of predetermined cycles up to 3 years (Cycle length= 28 days) | No | No |  |  |
| NCT02482090 | NCT02482090 | Number and type of reported adverse events, Determine the safety of the administration of TIL therapy including lymphodepleting chemotherapy and Interleukin-2 for patients with metastatic Ovarian Cancer by reporting adverse events according to CTCAE v. 4.0., 0-24 weeks | Treatment related immune responses, To evaluate the immunological impact of TIL therapy for patients with metastatic Ovarian Cancer, Up to 12 months\|Objective response rate, Clinical responses will be evaluated by RECIST 1.1., Up to 12 months\|Overall Survival, Overall Survival (OS), defined as time from treatment initiation to death, will be described with use of Kaplan Meier curve., Up to 12 months\|Progression free survival, Progression free survival (PFS), defined as the time from treatment initiation to disease progression, relapse or death due to any cause, which ever comes first, will be described with Kaplan Meier curve., Up to 12 months | No | No |  |  |
| NCT06626256 | NCT06626256 | Infusion limiting toxicities (ILTs), Assessed using the National Cancer Institute (NCI) Common Terminology Criteria for Adverse Events (CTCAE) version (v) 5.0. ILTs will be summarized by severity, grade and attribution., From the start of STIL101 for injection up to 28 days\|Treatment-related adverse events (AEs), Assessed by NCI CTCAE v 5.0. AEs will be summarized by severity, grade and attribution., Up to 2 years | Overall response rate (ORR), The proportion of subjects with confirmed complete response (CR) / immune related (i)CR or partial response (PR) / iPR will be assessed using Response Evaluation Criteria in Solid Tumors (RECIST) 1.1 and Immune-Modified (i)RECIST 1.0., Up to 2 years\|Disease control rate, The proportion of subjects who achieve CR/iCR, PR/iPR or maintain at least stable disease will be assessed using RECIST 1.1/iRECIST 1.0., From start of STIL101 for injection infusion for at least one post-treatment scan up to 2 years\|Overall survival, Analysis will be descriptive., From the start of STIL101 for injection until death due to any cause up to 2 years\|Overall survival, Analysis will be descriptive., From excisional biopsy until death due to any cause up to 2 years\|Progression-free survival, Analysis will be descriptive., From start of STIL101 for injection infusion until documented progressive disease or death due to any cause up to 2 years\|Failure to receive STIL101 after excisional biopsy, Analysis will be descriptive., Up to 2 years\|Time to lymphodepleting chemotherapy, Analysis will be descriptive., From subject informed consent to start of lymphodepleting chemotherapy up to 2 years\|Excisional biopsy complication rate, Analysis will be descriptive., Up to 2 years\|STIL101 for injection generation times, Analysis will be descriptive., Up to 2 years\|Successful STIL101 for injection generation and administration, Success rate for generation will be summarized and causes of failure will be documented. Those unable to receive STIL101 for injection will also be summarized., Up to 2 years\|STIL101 cell count, STIL101 cell count in patients with respect to baseline characteristics, clinical outcome and AEs will be described., Up to 2 years | No | No |  |  |
| NCT05296564 | NCT05296564 | Incidence of Treatment-Emergent Adverse Events [Safety and Tolerability], Adverse events that occur following engineered T cell transfer, 5 years\|objective tumor regression, change in index lesions' size, 5 years | immune monitoring for transferred cells persistence, blood test for ESO-TCRT cells' presence, 5 years | No | No |  |  |
| NCT02153905 | NCT02153905 | Maximum Tolerated Cell Dose (MTD), Highest dose at which less than or equal to 1 of 6 patients experienced a dose limiting toxicity (DLT) or the highest dose level studied if DLTs are not observed at any of the dose levels. DLT is defined as follows: Grade 3-5 allergic reactions related to the study cell infusion. Grade 3 and greater autoimmune reactions. Grades 3 and greater organ toxicity (cardiac, dermatologic, gastrointestinal, hepatic, pulmonary, renal/genitourinary, or neurologic) not pre-existing or due to the underlying malignancy and occurring within 30 days of study cell infusion and does not resolve within 72 hours. Treatment-related death within 8 weeks of the study cell infusion., Within 30 days of study cell infusion, before progression to next-higher dose level\|Number of Patients With Objective Tumor Regression, Objective tumor regression is defined as the number of participants with a complete or partial response per the Response Evaluation Criteria in Solid Tumors (RECIST) v1.0. Complete response is disappearance of all target lesions. Partial response is at least a 30% decrease in the sum of the longest diameter (LD) of target lesions, taking as reference the baseline sum LD., 6 and 12 weeks after cell infusion on up to 2 years\|Number of Treatment Related Adverse Events Related to T-Cell Receptor (TCR) Gene-Engineered Cells, Aggregate of all Grade ≥3 adverse events and their frequency possibly, probably or definitely related to the research. Adverse Events were assessed by the Common Terminology Criteria for Adverse Events (CTCAE) v.4.0. Grade 3 is severe or medically significant but not immediately life-threatening; hospitalization or prolongation of hospitalization indicated; disabling; limiting self care activities of daily living (ADL). Grade 4 is life-threatening consequences; urgent intervention indicated. Grade 5 is death related to adverse event., Date treatment consent signed to end of treatment, approximately 30 days\|Number of Participants With Serious and Non-Serious Adverse Events, Here is the number of participants with serious and non-serious adverse events assessed by the Common Terminology Criteria in Adverse Events (CTCAE v4.0). A non-serious adverse event is any untoward medical occurrence. A serious adverse event is an adverse event or suspected adverse reaction that results in death, a life threatening adverse drug experience, hospitalization, disruption of the ability to conduct normal life functions, congenital anomaly/birth defect or important medical events that jeopardize the patient or subject and may require medical or surgical intervention to prevent one of the previous outcomes mentioned., Date treatment consent signed to date off study, approximately 53 days for the Anti-MAGE-A3 A1 TCR PBL 1x10^9 Cells + Interleukin-2 (IL-2) Arm/Group, and 1 year and 4 months for the Anti-MAGE-A3 A1 TCR PBL 1x10^8 Cells + Interleukin-2 (IL-2) Arm/Group. | In Vivo Survival of T-Cell Receptor (TCR) Cells, In vivo survival of gene-engineered lymphocytes derived from the infused cells will be analyzed by tetramer analysis and staining for the T-cell receptor (TCR). Tetramer analysis is measured by % of peripheral blood., Up to 3 years after study cell infusion\|Number of Participants With Dose-Limiting Toxicity (DLT), DLT is defined as follows: Grade 3-5 allergic reactions related to the study cell infusion. Grade 3 and greater autoimmune reactions. Grades 3 and greater organ toxicity (cardiac, dermatologic, gastrointestinal, hepatic, pulmonary, renal/genitourinary, or neurologic) not pre-existing or due to the underlying malignancy and occurring within 30 days of study cell infusion and does not resolve within 72 hours. Treatment-related death within 8 weeks of the study cell infusion., Within 30 days of study cell infusion | Yes | No |  |  |
| JPRN-jRCTc031200283 | JPRN-jRCTc031200283 | Best overall response (BOR) rate based on Response Evaluation Criteria in Solid Tumours (RECIST) guideline (version 1.1).<br>Adverse events (type, frequency and severity) based on Common Terminology Criteria for Adverse Events (CTCAE) version 4.0. | Duration of Response <br>Stable Period<br>Progression-Free Survival <br>Overall Survival | No | No |  |  |
| ChiCTR1800019780 | ChiCTR1800019780 | response evaluation of solid tumor;Adverse Events; | count of CAR-T;cytokines; | No | No |  |  |

# **S2A. Included trial records — Part 5 of 5: registry and source links**

**Note:** *One row represents one deduplicated trial record. The 211 records are repeated across five field groups and can be joined using Trial ID. Broader-basket eligibility does not imply that gynecologic participants were enrolled or that cohort-specific outcomes are available.*

| **Trial ID** | **Public registry ID** | **Registry URL** | **Source record URL** |
| --- | --- | --- | --- |
| NCT07644403 | NCT07644403 | https://clinicaltrials.gov/study/NCT07644403 | https://clinicalintelligence.citeline.com/trials/details/706228?qId=e0a34b28-1096-4150-bafe-a46e4590377f |
| NCT07534813 | NCT07534813 | https://clinicaltrials.gov/study/NCT07534813 | https://clinicalintelligence.citeline.com/trials/details/699832?qId=e0a34b28-1096-4150-bafe-a46e4590377f |
| 695613 | EUCT 2024-517594-24-01 | https://euclinicaltrials.eu/search-for-clinical-trials/?lang=en&EUCT=2024-517594-24-01; https://www.cbio.dk/clinical#pipeline; https://www.globenewswire.com/news-release/2026/03/12/3254322/0/en/Cbio-A-S-Receives-European-Regulatory-Clearance-to-Begin-First-in-Human-Clinical-Trial-of-Next-Generation-T-Cell-Therapy-in-late-stage-Cervical-Cancer.html | https://clinicalintelligence.citeline.com/trials/details/695613?qId=e0a34b28-1096-4150-bafe-a46e4590377f |
| NCT07420010 | NCT07420010 | https://clinicaltrials.gov/study/NCT07420010 | https://clinicalintelligence.citeline.com/trials/details/693163?qId=e0a34b28-1096-4150-bafe-a46e4590377f |
| NCT07266298 | NCT07266298 | https://clinicaltrials.gov/study/NCT07266298 | https://clinicalintelligence.citeline.com/trials/details/685876?qId=e0a34b28-1096-4150-bafe-a46e4590377f |
| NCT07231081 | NCT07231081 | https://clinicaltrials.gov/study/NCT07231081 | https://clinicalintelligence.citeline.com/trials/details/684015?qId=e0a34b28-1096-4150-bafe-a46e4590377f |
| NCT07216105 | NCT07216105 | https://clinicaltrials.gov/study/NCT07216105 | https://clinicalintelligence.citeline.com/trials/details/676847?qId=e0a34b28-1096-4150-bafe-a46e4590377f |
| 668508 | ChiCTR2500109506 | https://www.chictr.org.cn/showproj.html?proj=271594 | https://clinicalintelligence.citeline.com/trials/details/668508?qId=e0a34b28-1096-4150-bafe-a46e4590377f |
| NCT07181720 | NCT07181720 | https://clinicaltrials.gov/study/NCT07181720 | https://clinicalintelligence.citeline.com/trials/details/667771?qId=e0a34b28-1096-4150-bafe-a46e4590377f |
| NCT07106814 | NCT07106814 | https://clinicaltrials.gov/study/NCT07106814 | https://clinicalintelligence.citeline.com/trials/details/653201?qId=e0a34b28-1096-4150-bafe-a46e4590377f |
| NCT07101549 | NCT07101549 | https://clinicaltrials.gov/study/NCT07101549 | https://clinicalintelligence.citeline.com/trials/details/652460?qId=e0a34b28-1096-4150-bafe-a46e4590377f |
| NCT07067255 | NCT07067255 | https://clinicaltrials.gov/study/NCT07067255 | https://clinicalintelligence.citeline.com/trials/details/647094?qId=e0a34b28-1096-4150-bafe-a46e4590377f |
| NCT07030907 | NCT07030907 | https://clinicaltrials.gov/study/NCT07030907 | https://clinicalintelligence.citeline.com/trials/details/640247?qId=e0a34b28-1096-4150-bafe-a46e4590377f |
| NCT07010523 | NCT07010523 | https://clinicaltrials.gov/study/NCT07010523 | https://clinicalintelligence.citeline.com/trials/details/581716?qId=e0a34b28-1096-4150-bafe-a46e4590377f |
| NCT06951425 | NCT06951425 | https://clinicaltrials.gov/study/NCT06951425 | https://clinicalintelligence.citeline.com/trials/details/573207?qId=e0a34b28-1096-4150-bafe-a46e4590377f |
| NCT06912152 | NCT06912152 | https://clinicaltrials.gov/study/NCT06912152 | https://clinicalintelligence.citeline.com/trials/details/566586?qId=e0a34b28-1096-4150-bafe-a46e4590377f |
| NCT06904131 | NCT06904131 | https://clinicaltrials.gov/study/NCT06904131 | https://clinicalintelligence.citeline.com/trials/details/565481?qId=e0a34b28-1096-4150-bafe-a46e4590377f |
| NCT06846424 | NCT06846424 | https://clinicaltrials.gov/study/NCT06846424 | https://clinicalintelligence.citeline.com/trials/details/559126?qId=e0a34b28-1096-4150-bafe-a46e4590377f |
| NCT06834542 | NCT06834542 | https://clinicaltrials.gov/study/NCT06834542 | https://clinicalintelligence.citeline.com/trials/details/557885?qId=e0a34b28-1096-4150-bafe-a46e4590377f |
| NCT06748872 | NCT06748872 | https://clinicaltrials.gov/study/NCT06748872 | https://clinicalintelligence.citeline.com/trials/details/550110?qId=e0a34b28-1096-4150-bafe-a46e4590377f |
| NCT06710288 | NCT06710288 | https://clinicaltrials.gov/study/NCT06710288 | https://clinicalintelligence.citeline.com/trials/details/546102?qId=e0a34b28-1096-4150-bafe-a46e4590377f |
| 545596 | EUCT 2023-504632-17-00 | https://euclinicaltrials.eu/search-for-clinical-trials/?lang=en&EUCT=2023-504632-17-00 | https://clinicalintelligence.citeline.com/trials/details/545596?qId=e0a34b28-1096-4150-bafe-a46e4590377f |
| NCT06697665 | NCT06697665 | https://clinicaltrials.gov/study/NCT06697665 | https://clinicalintelligence.citeline.com/trials/details/544929?qId=e0a34b28-1096-4150-bafe-a46e4590377f |
| NCT06658951 | NCT06658951 | https://clinicaltrials.gov/study/NCT06658951 | https://clinicalintelligence.citeline.com/trials/details/541840?qId=e0a34b28-1096-4150-bafe-a46e4590377f |
| NCT06646627 | NCT06646627 | https://clinicaltrials.gov/study/NCT06646627 | https://clinicalintelligence.citeline.com/trials/details/539940?qId=e0a34b28-1096-4150-bafe-a46e4590377f |
| NCT06630611 | NCT06630611 | https://clinicaltrials.gov/study/NCT06630611 | https://clinicalintelligence.citeline.com/trials/details/538589?qId=e0a34b28-1096-4150-bafe-a46e4590377f |
| NCT06598371 | NCT06598371 | https://clinicaltrials.gov/study/NCT06598371 | https://clinicalintelligence.citeline.com/trials/details/535650?qId=e0a34b28-1096-4150-bafe-a46e4590377f |
| NCT06544720 | NCT06544720 | https://clinicaltrials.gov/study/NCT06544720 | https://clinicalintelligence.citeline.com/trials/details/530267?qId=e0a34b28-1096-4150-bafe-a46e4590377f |
| NCT06509490 | NCT06509490 | https://clinicaltrials.gov/study/NCT06509490 | https://clinicalintelligence.citeline.com/trials/details/526994?qId=e0a34b28-1096-4150-bafe-a46e4590377f |
| NCT06505551 | NCT06505551 | https://clinicaltrials.gov/study/NCT06505551 | https://clinicalintelligence.citeline.com/trials/details/526658?qId=e0a34b28-1096-4150-bafe-a46e4590377f |
| NCT06481592 | NCT06481592 | https://clinicaltrials.gov/study/NCT06481592 | https://clinicalintelligence.citeline.com/trials/details/524041?qId=e0a34b28-1096-4150-bafe-a46e4590377f |
| NCT06453057 | NCT06453057 | https://clinicaltrials.gov/study/NCT06453057 | https://clinicalintelligence.citeline.com/trials/details/520996?qId=e0a34b28-1096-4150-bafe-a46e4590377f |
| 518933 | ChiCTR2000032124 | https://meetings.asco.org/abstracts-presentations/239183; https://meetings.asco.org/abstracts-presentations/239183/slides; https://meetings.asco.org/abstracts-presentations/239183/video; https://www.chictr.org.cn/showproj.html?proj=49427 | https://clinicalintelligence.citeline.com/trials/details/518933?qId=e0a34b28-1096-4150-bafe-a46e4590377f |
| NCT06375187 | NCT06375187 | https://clinicaltrials.gov/study/NCT06375187 | https://clinicalintelligence.citeline.com/trials/details/513511?qId=e0a34b28-1096-4150-bafe-a46e4590377f |
| NCT06358053 | NCT06358053 | https://clinicaltrials.gov/study/NCT06358053 | https://clinicalintelligence.citeline.com/trials/details/512130?qId=e0a34b28-1096-4150-bafe-a46e4590377f |
| 510939 |  | https://d1io3yog0oux5.cloudfront.net/_0d39917b8043c2c8ee51e7c1d62b95b8/anixa/db/29/1138/pdf/ANIXA+Corp+Presentation++March+2024_final.pdf; https://d1io3yog0oux5.cloudfront.net/_1871eab178e352140bdc0724cd42af56/anixa/db/29/1195/pdf/ANIX-Nov-2024-3.pdf; https://d1io3yog0oux5.cloudfront.net/_b4512099caf1041097222cc7c07a0efd/anixa/db/29/1253/pdf/ANIX-June-2025.pdf; https://d1io3yog0oux5.cloudfront.net/_fa6e00e4f049e9b00d54ec4beef88dff/anixa/db/29/1228/pdf/ANIX-May-2025_2.pdf; https://darebioscience.gcs-web.com//static-files/1a03ec51-c40b-44f3-bfc7-17f655a68ed3; https://ir.anixa.com/press-releases/detail/1111/anixa-biosciences-reports-encouraging-patient-survival | https://clinicalintelligence.citeline.com/trials/details/510939?qId=e0a34b28-1096-4150-bafe-a46e4590377f |
| NCT06342336 | NCT06342336 | https://clinicaltrials.gov/study/NCT06342336 | https://clinicalintelligence.citeline.com/trials/details/510719?qId=e0a34b28-1096-4150-bafe-a46e4590377f |
| NCT06327997 | NCT06327997 | https://clinicaltrials.gov/study/NCT06327997 | https://clinicalintelligence.citeline.com/trials/details/509566?qId=e0a34b28-1096-4150-bafe-a46e4590377f |
| NCT06305299 | NCT06305299 | https://clinicaltrials.gov/study/NCT06305299 | https://clinicalintelligence.citeline.com/trials/details/507616?qId=e0a34b28-1096-4150-bafe-a46e4590377f |
| NCT06256055 | NCT06256055 | https://clinicaltrials.gov/study/NCT06256055 | https://clinicalintelligence.citeline.com/trials/details/504019?qId=e0a34b28-1096-4150-bafe-a46e4590377f |
| NCT06241781 | NCT06241781 | https://clinicaltrials.gov/study/NCT06241781 | https://clinicalintelligence.citeline.com/trials/details/502879?qId=e0a34b28-1096-4150-bafe-a46e4590377f |
| NCT06218914 | NCT06218914 | https://clinicaltrials.gov/study/NCT06218914 | https://clinicalintelligence.citeline.com/trials/details/501207?qId=e0a34b28-1096-4150-bafe-a46e4590377f |
| NCT06191900 | NCT06191900 | https://clinicaltrials.gov/study/NCT06191900 | https://clinicalintelligence.citeline.com/trials/details/498706?qId=e0a34b28-1096-4150-bafe-a46e4590377f |
| 496428 | CTR20233999 | http://www.chinadrugtrials.org.cn/; https://ascopubs.org/doi/10.1200/JCO.2025.43.16_suppl.9548; https://meetings.asco.org/abstracts-presentations/247341; https://meetings.asco.org/abstracts-presentations/247341/poster; https://mp.weixin.qq.com/s/WXojnheVx07O91OXsrW6Eg; https://www.abstractsonline.com/pp8/#!/21436/presentation/11384 | https://clinicalintelligence.citeline.com/trials/details/496428?qId=e0a34b28-1096-4150-bafe-a46e4590377f |
| NCT06145802 | NCT06145802 | https://clinicaltrials.gov/study/NCT06145802 | https://clinicalintelligence.citeline.com/trials/details/493453?qId=e0a34b28-1096-4150-bafe-a46e4590377f |
| 489390 |  | https://d1io3yog0oux5.cloudfront.net/_c2123266fc8bbab207b00308e51799b4/adaptimmune/db/249/3634/pdf/ADAP+Corporate+Deck+October+2023_ESMO+UPDATE.pdf; https://d1io3yog0oux5.cloudfront.net/_d5d9a3ef15c5ab4c07a646a6d70a45e3/adaptimmune/db/249/3664/pdf/JP+Morgan_ADAP_Final_Jan2024.pdf; https://www.adaptimmune.com/investors-and-media/news-center/press-releases/detail/232/adaptimmune-enhances-its-pipeline-of-wholly-owned-cell; https://www.adaptimmune.com/investors-and-media/news-center/press-releases/detail/255/adaptimmune-selects-adp-600-as-clinical-candidate-for; https://www.adaptimmune.com/investors-and-media/news-center/press-releases/detail/256/adaptimmune-reports-third-quarter-financial-results-and; https://www.adaptimmune.com/investors-and-media/news-center/press-releases/detail/273/adaptimmune-reports-q2-2024-financial-and-business-updates; https://www.adaptimmune.com/pipeline; https://www.adaptimmune.com/pipeline/pre-clinical-pipeline | https://clinicalintelligence.citeline.com/trials/details/489390?qId=e0a34b28-1096-4150-bafe-a46e4590377f |
| NCT06051695 | NCT06051695 | https://clinicaltrials.gov/study/NCT06051695 | https://clinicalintelligence.citeline.com/trials/details/486804?qId=e0a34b28-1096-4150-bafe-a46e4590377f |
| NCT06010875 | NCT06010875 | https://clinicaltrials.gov/study/NCT06010875 | https://clinicalintelligence.citeline.com/trials/details/482876?qId=e0a34b28-1096-4150-bafe-a46e4590377f |
| NCT05973487 | NCT05973487 | https://clinicaltrials.gov/study/NCT05973487 | https://clinicalintelligence.citeline.com/trials/details/479984?qId=e0a34b28-1096-4150-bafe-a46e4590377f |
| NCT05971576 | NCT05971576 | https://clinicaltrials.gov/study/NCT05971576 | https://clinicalintelligence.citeline.com/trials/details/479867?qId=e0a34b28-1096-4150-bafe-a46e4590377f |
| NCT05952947 | NCT05952947 | https://clinicaltrials.gov/study/NCT05952947 | https://clinicalintelligence.citeline.com/trials/details/478322?qId=e0a34b28-1096-4150-bafe-a46e4590377f |
| NCT05941936 | NCT05941936 | https://clinicaltrials.gov/study/NCT05941936 | https://clinicalintelligence.citeline.com/trials/details/477254?qId=e0a34b28-1096-4150-bafe-a46e4590377f |
| NCT05902520 | NCT05902520 | https://clinicaltrials.gov/study/NCT05902520 | https://clinicalintelligence.citeline.com/trials/details/473930?qId=e0a34b28-1096-4150-bafe-a46e4590377f |
| NCT05877599 | NCT05877599 | https://clinicaltrials.gov/study/NCT05877599 | https://clinicalintelligence.citeline.com/trials/details/471137?qId=e0a34b28-1096-4150-bafe-a46e4590377f |
| NCT05848999 | NCT05848999 | https://clinicaltrials.gov/study/NCT05848999 | https://clinicalintelligence.citeline.com/trials/details/468622?qId=e0a34b28-1096-4150-bafe-a46e4590377f |
| NCT05787535 | NCT05787535 | https://clinicaltrials.gov/study/NCT05787535 | https://clinicalintelligence.citeline.com/trials/details/463603?qId=e0a34b28-1096-4150-bafe-a46e4590377f |
| 463409 |  | https://coeptis.investorroom.com/2023-03-21-Coeptis-Therapeutics-SNAP-CAR-Technology-Platform-Highlighted-in-Peer-Reviewed-Article-in-Translational-Medicine; https://coeptis.investorroom.com/2024-05-02-Coeptis-Therapeutics-Announces-Poster-Data-Presentation-at-American-Society-of-Gene-Cell-Therapy-ASGCT-27th-Annual-Meeting; https://coeptistx.com/our-science/overview; https://coeptistx.com/our-science/snap-car-t; https://coeptistx.com/wp-content/uploads/2024/06/Coeptis-Corporate-Summary-deck-C10060-v1.0-June2024.pdf; https://investors.coeptistx.com/news-releases/news-release-details/coeptis-therapeutics-secures-worldwide-development-and; https://news.engineering.pitt.edu/customizing-t-cell-based-immunotherapies-in-a-snap/ | https://clinicalintelligence.citeline.com/trials/details/463409?qId=e0a34b28-1096-4150-bafe-a46e4590377f |
| NCT05783089 | NCT05783089 | https://clinicaltrials.gov/study/NCT05783089 | https://clinicalintelligence.citeline.com/trials/details/463263?qId=e0a34b28-1096-4150-bafe-a46e4590377f |
| NCT05775666 | NCT05775666 | https://clinicaltrials.gov/study/NCT05775666 | https://clinicalintelligence.citeline.com/trials/details/462742?qId=e0a34b28-1096-4150-bafe-a46e4590377f |
| NCT05729399 | NCT05729399 | https://clinicaltrials.gov/study/NCT05724732 | https://clinicalintelligence.citeline.com/trials/details/458909?qId=e0a34b28-1096-4150-bafe-a46e4590377f |
| 456726 |  | https://investor.regeneron.com//static-files/4ac68b22-7abe-4d1f-a15a-2a9b739cc935; https://ir.2seventybio.com//news-releases/news-release-details/2seventy-bio-announces-strategic-restructuring-prioritize-growth; https://ir.2seventybio.com//news-releases/news-release-details/2seventy-bio-provides-company-outlook-2023; https://ir.2seventybio.com/static-files/fe5e9d43-9559-449f-a59f-4213be9d85e8; https://www.regeneron.com/science/investigational-pipeline | https://clinicalintelligence.citeline.com/trials/details/456726?qId=e0a34b28-1096-4150-bafe-a46e4590377f |
| NCT05672459 | NCT05672459 | https://clinicaltrials.gov/study/NCT05672459 | https://clinicalintelligence.citeline.com/trials/details/450963?qId=e0a34b28-1096-4150-bafe-a46e4590377f |
| NCT05881525 | NCT05881525 | https://clinicaltrials.gov/study/NCT05881525 | https://clinicalintelligence.citeline.com/trials/details/449492?qId=e0a34b28-1096-4150-bafe-a46e4590377f |
| 443882 | ChiCTR2200064112 | https://www.chictr.org.cn/showproj.html?proj=180320 | https://clinicalintelligence.citeline.com/trials/details/443882?qId=e0a34b28-1096-4150-bafe-a46e4590377f |
| NCT05568680 | NCT05568680 | https://clinicaltrials.gov/study/NCT05568680 | https://clinicalintelligence.citeline.com/trials/details/443103?qId=e0a34b28-1096-4150-bafe-a46e4590377f |
| NCT05539768 | NCT05539768 | https://clinicaltrials.gov/study/NCT05539768 | https://clinicalintelligence.citeline.com/trials/details/442289?qId=e0a34b28-1096-4150-bafe-a46e4590377f |
| 439506 | CTR20221207 | http://www.chinadrugtrials.org.cn/; https://www.cde.org.cn/main/xxgk/listpage/4b5255eb0a84820cef4ca3e8b6bbe20c; https://www.juncell.com/en/clinical-trail-16 | https://clinicalintelligence.citeline.com/trials/details/439506?qId=e0a34b28-1096-4150-bafe-a46e4590377f |
| NCT05483491 | NCT05483491 | https://clinicaltrials.gov/study/NCT05483491 | https://clinicalintelligence.citeline.com/trials/details/438548?qId=e0a34b28-1096-4150-bafe-a46e4590377f |
| NCT05475847 | NCT05475847 | https://clinicaltrials.gov/study/NCT05475847 | https://clinicalintelligence.citeline.com/trials/details/438059?qId=e0a34b28-1096-4150-bafe-a46e4590377f |
| NCT05472857 | NCT05472857 | https://clinicaltrials.gov/study/NCT05472857 | https://clinicalintelligence.citeline.com/trials/details/437829?qId=e0a34b28-1096-4150-bafe-a46e4590377f |
| NCT05468307 | NCT05468307 | https://clinicaltrials.gov/study/NCT05468307 | https://clinicalintelligence.citeline.com/trials/details/437503?qId=e0a34b28-1096-4150-bafe-a46e4590377f |
| NCT05468190 | NCT05468190 | https://clinicaltrials.gov/study/NCT05468190 | https://clinicalintelligence.citeline.com/trials/details/437498?qId=e0a34b28-1096-4150-bafe-a46e4590377f |
| NCT05430373 | NCT05430373 | https://clinicaltrials.gov/study/NCT05430373 | https://clinicalintelligence.citeline.com/trials/details/435318?qId=e0a34b28-1096-4150-bafe-a46e4590377f |
| NCT05417750 | NCT05417750 | https://clinicaltrials.gov/study/NCT05417750 | https://clinicalintelligence.citeline.com/trials/details/434502?qId=e0a34b28-1096-4150-bafe-a46e4590377f |
| NCT05397093 | NCT05397093 | https://clinicaltrials.gov/study/NCT05397093 | https://clinicalintelligence.citeline.com/trials/details/433483?qId=e0a34b28-1096-4150-bafe-a46e4590377f |
| NCT05373147 | NCT05373147 | https://clinicaltrials.gov/study/NCT04503980 | https://clinicalintelligence.citeline.com/trials/details/432295?qId=e0a34b28-1096-4150-bafe-a46e4590377f |
| NCT05366478 | NCT05366478 | https://clinicaltrials.gov/study/NCT05366478 | https://clinicalintelligence.citeline.com/trials/details/431701?qId=e0a34b28-1096-4150-bafe-a46e4590377f |
| 431389 |  | https://ir.tscan.com//static-files/1d066354-1ed3-4f4f-b97b-28806d9da9f5; https://ir.tscan.com/news-releases/news-release-details/tscan-therapeutics-reports-third-quarter-2021-financial-results; https://ir.tscan.com/static-files/1d066354-1ed3-4f4f-b97b-28806d9da9f5; https://tscan.gcs-web.com//news-releases/news-release-details/tscan-therapeutics-presents-phase-1-trial-design-solid-tumor; https://www.tscan.com/pipeline/ | https://clinicalintelligence.citeline.com/trials/details/431389?qId=e0a34b28-1096-4150-bafe-a46e4590377f |
| 430435 | CTR20260154 | http://www.chinadrugtrials.org.cn/clinicaltrials.searchlistdetail.dhtml?keywords=CTR20260154; https://mp.weixin.qq.com/s/Cf75PPvkam1w9AN1Bb-4KA; https://www.cde.org.cn/main/xxgk/listpage/4b5255eb0a84820cef4ca3e8b6bbe20c; https://www.cde.org.cn/main/xxgk/listpage/9f9c74c73e0f8f56a8bfbc646055026d; https://www.medvalley.cn/yiyao/73263.html | https://clinicalintelligence.citeline.com/trials/details/430435?qId=e0a34b28-1096-4150-bafe-a46e4590377f |
| NCT05342506 | NCT05342506 | https://clinicaltrials.gov/study/NCT05342506 | https://clinicalintelligence.citeline.com/trials/details/430365?qId=e0a34b28-1096-4150-bafe-a46e4590377f |
| NCT05302037 | NCT05302037 | https://clinicaltrials.gov/study/NCT05302037 | https://clinicalintelligence.citeline.com/trials/details/428709?qId=e0a34b28-1096-4150-bafe-a46e4590377f |
| 427237 | ChiCTR2200057362 | http://www.chictr.org.cn/showproj.aspx?proj=153219 | https://clinicalintelligence.citeline.com/trials/details/427237?qId=e0a34b28-1096-4150-bafe-a46e4590377f |
| NCT05238818 | NCT05238818 | https://clinicaltrials.gov/study/NCT05238818 | https://clinicalintelligence.citeline.com/trials/details/424970?qId=e0a34b28-1096-4150-bafe-a46e4590377f |
| NCT05211557 | NCT05211557 | https://clinicaltrials.gov/study/NCT05211557 | https://clinicalintelligence.citeline.com/trials/details/423666?qId=e0a34b28-1096-4150-bafe-a46e4590377f |
| NCT05194735 | NCT05194735 | https://clinicaltrials.gov/study/NCT05194735 | https://clinicalintelligence.citeline.com/trials/details/422987?qId=e0a34b28-1096-4150-bafe-a46e4590377f |
| 421887 | ChiCTR2100054914 | https://www.chictr.org.cn/showprojEN.html?proj=146487; https://www.ictbio.com/pipeline/ | https://clinicalintelligence.citeline.com/trials/details/421887?qId=e0a34b28-1096-4150-bafe-a46e4590377f |
| NCT05274451 | NCT05274451 | https://clinicaltrials.gov/study/NCT05274451 | https://clinicalintelligence.citeline.com/trials/details/421034?qId=e0a34b28-1096-4150-bafe-a46e4590377f |
| NCT05152797 | NCT05152797 | https://clinicaltrials.gov/study/NCT05152797 | https://clinicalintelligence.citeline.com/trials/details/420441?qId=e0a34b28-1096-4150-bafe-a46e4590377f |
| NCT05141253 | NCT05141253 | https://clinicaltrials.gov/study/NCT05141253 | https://clinicalintelligence.citeline.com/trials/details/419745?qId=e0a34b28-1096-4150-bafe-a46e4590377f |
| NCT05357027 | NCT05357027 | https://clinicaltrials.gov/study/NCT05357027 | https://clinicalintelligence.citeline.com/trials/details/419430?qId=e0a34b28-1096-4150-bafe-a46e4590377f |
| NCT05122221 | NCT05122221 | https://clinicaltrials.gov/study/NCT05122221 | https://clinicalintelligence.citeline.com/trials/details/418329?qId=e0a34b28-1096-4150-bafe-a46e4590377f |
| NCT05098171 | NCT05098171 | https://clinicaltrials.gov/study/NCT05098171 | https://clinicalintelligence.citeline.com/trials/details/417045?qId=e0a34b28-1096-4150-bafe-a46e4590377f |
| NCT05089266 | NCT05089266 | https://clinicaltrials.gov/study/NCT05089266 | https://clinicalintelligence.citeline.com/trials/details/416574?qId=e0a34b28-1096-4150-bafe-a46e4590377f |
| NCT05499715 | NCT05499715 | https://clinicaltrials.gov/study/NCT05499715 | https://clinicalintelligence.citeline.com/trials/details/411513?qId=e0a34b28-1096-4150-bafe-a46e4590377f |
| NCT04981691 | NCT04981691 | https://clinicaltrials.gov/study/NCT04981691 | https://clinicalintelligence.citeline.com/trials/details/409929?qId=e0a34b28-1096-4150-bafe-a46e4590377f |
| NCT05393635 | NCT05393635 | https://clinicaltrials.gov/study/NCT05393635 | https://clinicalintelligence.citeline.com/trials/details/403666?qId=e0a34b28-1096-4150-bafe-a46e4590377f |
| 403252 |  | https://cartherics.com/; https://cartherics.com/news/good-news-from-cartherics-the-companys-off-the-shelf-immune-therapy-for-ovarian-cancer-is-close-to-clinical-trials/; https://cartherics.com/the-pipeline/ | https://clinicalintelligence.citeline.com/trials/details/403252?qId=e0a34b28-1096-4150-bafe-a46e4590377f |
| NCT04766320 | NCT04766320 | https://clinicaltrials.gov/study/NCT04766320 | https://clinicalintelligence.citeline.com/trials/details/397575?qId=e0a34b28-1096-4150-bafe-a46e4590377f |
| NCT05107739 | NCT05107739 | https://clinicaltrials.gov/study/NCT05107739 | https://clinicalintelligence.citeline.com/trials/details/397292?qId=e0a34b28-1096-4150-bafe-a46e4590377f |
| 394743 | ChiCTR2100042320 | https://www.chictr.org.cn/showproj.html?proj=54238 | https://clinicalintelligence.citeline.com/trials/details/394743?qId=e0a34b28-1096-4150-bafe-a46e4590377f |
| NCT04674488 | NCT04674488 | https://clinicaltrials.gov/study/NCT04674488 | https://clinicalintelligence.citeline.com/trials/details/393284?qId=e0a34b28-1096-4150-bafe-a46e4590377f |
| NCT05239143 | NCT05239143 | https://clinicaltrials.gov/study/NCT05239143 | https://clinicalintelligence.citeline.com/trials/details/392853?qId=e0a34b28-1096-4150-bafe-a46e4590377f |
| NCT04670068 | NCT04670068 | https://clinicaltrials.gov/study/NCT04670068 | https://clinicalintelligence.citeline.com/trials/details/392151?qId=e0a34b28-1096-4150-bafe-a46e4590377f |
| 389985 |  | http://www.senlangbio.com/col.jsp?id=105 | https://clinicalintelligence.citeline.com/trials/details/389985?qId=e0a34b28-1096-4150-bafe-a46e4590377f |
| 389901 |  | https://docs.wixstatic.com/ugd/233d22_8890544160a9433fbfbfaab0f903d337.pdf; https://ea68ab67-ef93-4814-a431-d4276b866a62.filesusr.com/ugd/233d22_2231a96a695e45deb71245baa7a7009d.pdf; https://ea68ab67-ef93-4814-a431-d4276b866a62.filesusr.com/ugd/233d22_fc3291d7fbb448e0806a7fd90c3e97ef.pdf; https://www.tmunity.com/pipeline | https://clinicalintelligence.citeline.com/trials/details/389901?qId=e0a34b28-1096-4150-bafe-a46e4590377f |
| NCT04627740 | NCT04627740 | https://clinicaltrials.gov/study/NCT04627740 | https://clinicalintelligence.citeline.com/trials/details/389328?qId=e0a34b28-1096-4150-bafe-a46e4590377f |
| NCT04611126 | NCT04611126 | https://clinicaltrials.gov/study/NCT04611126 | https://clinicalintelligence.citeline.com/trials/details/388365?qId=e0a34b28-1096-4150-bafe-a46e4590377f |
| NCT04562298 | NCT04562298 | https://clinicaltrials.gov/study/NCT04562298 | https://clinicalintelligence.citeline.com/trials/details/385342?qId=e0a34b28-1096-4150-bafe-a46e4590377f |
| NCT04556669 | NCT04556669 | https://clinicaltrials.gov/study/NCT04556669 | https://clinicalintelligence.citeline.com/trials/details/384951?qId=e0a34b28-1096-4150-bafe-a46e4590377f |
| NCT04483778 | NCT04483778 | https://clinicaltrials.gov/study/NCT04483778 | https://clinicalintelligence.citeline.com/trials/details/380162?qId=e0a34b28-1096-4150-bafe-a46e4590377f |
| NCT04476251 | NCT04476251 | https://clinicaltrials.gov/study/NCT04476251 | https://clinicalintelligence.citeline.com/trials/details/379828?qId=e0a34b28-1096-4150-bafe-a46e4590377f |
| NCT04443296 | NCT04443296 | https://clinicaltrials.gov/study/NCT04443296 | https://clinicalintelligence.citeline.com/trials/details/377794?qId=e0a34b28-1096-4150-bafe-a46e4590377f |
| NCT04389229 | NCT04389229 | https://clinicaltrials.gov/study/NCT04389229 | https://clinicalintelligence.citeline.com/trials/details/374638?qId=e0a34b28-1096-4150-bafe-a46e4590377f |
| 372638 |  | http://t-cure.com/pipeline/#820tcr; http://t-cure.com/wp-content/uploads/2020/04/T-Cure-and-NCI-Collaboration.pdf; https://t-cure.com/2021/10/05/october-5-2021-t-cure-bioscience-inc-and-atlas-antibodies-ab-enter-a-collaboration-agreement-for-the-development-manufacture-and-supply-of-ct83-kk-lc-1-monoclonal-antibodies/; https://t-cure.com/october-6-2022-nci-and-rutgers-university-open-phase-1-clinical-trials-of-t-cell-receptor-therapy-targeting-kk-lc-1-for-multiple-solid-tumors/; https://www.abstractsonline.com/pp8/#!/9325/presentation/2597; https://www.biospace.com/article/releases/t-cure-bioscience-signs-exclusive-worldwide-license-with-nci-for-the-development-of-t-cell-receptor-therapy-for-treatment-of-solid-tumors/ | https://clinicalintelligence.citeline.com/trials/details/372638?qId=e0a34b28-1096-4150-bafe-a46e4590377f |
| NCT05035407 | NCT05035407 | https://clinicaltrials.gov/study/NCT05035407 | https://clinicalintelligence.citeline.com/trials/details/372067?qId=e0a34b28-1096-4150-bafe-a46e4590377f |
| NCT04318964 | NCT04318964 | https://clinicaltrials.gov/study/NCT04318964 | https://clinicalintelligence.citeline.com/trials/details/370361?qId=e0a34b28-1096-4150-bafe-a46e4590377f |
| NCT04503278 | NCT04503278 | https://clinicaltrials.gov/study/NCT04503278 | https://clinicalintelligence.citeline.com/trials/details/364693?qId=e0a34b28-1096-4150-bafe-a46e4590377f |
| NCT04025216 | NCT04025216 | https://clinicaltrials.gov/study/NCT04025216 | https://clinicalintelligence.citeline.com/trials/details/353563?qId=e0a34b28-1096-4150-bafe-a46e4590377f |
| NCT03992326 | NCT03992326 | https://clinicaltrials.gov/study/NCT03992326 | https://clinicalintelligence.citeline.com/trials/details/351898?qId=e0a34b28-1096-4150-bafe-a46e4590377f |
| NCT03970382 | NCT03970382 | https://clinicaltrials.gov/study/NCT03970382 | https://clinicalintelligence.citeline.com/trials/details/350735?qId=e0a34b28-1096-4150-bafe-a46e4590377f |
| NCT03912831 | NCT03912831 | https://clinicaltrials.gov/study/NCT03912831 | https://clinicalintelligence.citeline.com/trials/details/347369?qId=e0a34b28-1096-4150-bafe-a46e4590377f |
| NCT03907527 | NCT03907527 | https://clinicaltrials.gov/study/NCT03907527 | https://clinicalintelligence.citeline.com/trials/details/342799?qId=e0a34b28-1096-4150-bafe-a46e4590377f |
| NCT04650451 | NCT04650451 | https://clinicaltrials.gov/study/NCT04650451 | https://clinicalintelligence.citeline.com/trials/details/342672?qId=e0a34b28-1096-4150-bafe-a46e4590377f |
| NCT03745326 | NCT03745326 | https://clinicaltrials.gov/study/NCT03745326 | https://clinicalintelligence.citeline.com/trials/details/337024?qId=e0a34b28-1096-4150-bafe-a46e4590377f |
| NCT03686124 | NCT03686124 | https://clinicaltrials.gov/study/NCT03686124 | https://clinicalintelligence.citeline.com/trials/details/333619?qId=e0a34b28-1096-4150-bafe-a46e4590377f |
| NCT03638206 | NCT03638206 | https://clinicaltrials.gov/study/NCT03638206 | https://clinicalintelligence.citeline.com/trials/details/330903?qId=e0a34b28-1096-4150-bafe-a46e4590377f |
| NCT03610490 | NCT03610490 | https://clinicaltrials.gov/study/NCT03610490 | https://clinicalintelligence.citeline.com/trials/details/329355?qId=e0a34b28-1096-4150-bafe-a46e4590377f |
| NCT03578406 | NCT03578406 | https://clinicaltrials.gov/study/NCT03578406 | https://clinicalintelligence.citeline.com/trials/details/327552?qId=e0a34b28-1096-4150-bafe-a46e4590377f |
| 327082 | ChiCTR1800016805 | http://www.chictr.org.cn/showproj.aspx?proj=28543 | https://clinicalintelligence.citeline.com/trials/details/327082?qId=e0a34b28-1096-4150-bafe-a46e4590377f |
| 326892 | ChiCTR1800016764 | http://www.chictr.org.cn/showproj.aspx?proj=28460 | https://clinicalintelligence.citeline.com/trials/details/326892?qId=e0a34b28-1096-4150-bafe-a46e4590377f |
| NCT03545815 | NCT03545815 | https://clinicaltrials.gov/study/NCT03545815 | https://clinicalintelligence.citeline.com/trials/details/325491?qId=e0a34b28-1096-4150-bafe-a46e4590377f |
| NCT03449108 | NCT03449108 | https://clinicaltrials.gov/study/NCT03449108 | https://clinicalintelligence.citeline.com/trials/details/319502?qId=e0a34b28-1096-4150-bafe-a46e4590377f |
| NCT04102436 | NCT04102436 | https://clinicaltrials.gov/study/NCT04102436 | https://clinicalintelligence.citeline.com/trials/details/317523?qId=e0a34b28-1096-4150-bafe-a46e4590377f |
| NCT03412526 | NCT03412526 | https://clinicaltrials.gov/study/NCT03412526 | https://clinicalintelligence.citeline.com/trials/details/317271?qId=e0a34b28-1096-4150-bafe-a46e4590377f |
| NCT03907852 | NCT03907852 | https://clinicaltrials.gov/study/NCT03907852 | https://clinicalintelligence.citeline.com/trials/details/311929?qId=e0a34b28-1096-4150-bafe-a46e4590377f |
| NCT03296137 | NCT03296137 | https://clinicaltrials.gov/study/NCT03296137 | https://clinicalintelligence.citeline.com/trials/details/310133?qId=e0a34b28-1096-4150-bafe-a46e4590377f |
| NCT04072263 | NCT04072263 | https://clinicaltrials.gov/study/NCT04072263 | https://clinicalintelligence.citeline.com/trials/details/309768?qId=e0a34b28-1096-4150-bafe-a46e4590377f |
| NCT03287674 | NCT03287674 | https://clinicaltrials.gov/study/NCT03287674 | https://clinicalintelligence.citeline.com/trials/details/309488?qId=e0a34b28-1096-4150-bafe-a46e4590377f |
| NCT03158935 | NCT03158935 | https://clinicaltrials.gov/study/NCT03158935 | https://clinicalintelligence.citeline.com/trials/details/301931?qId=e0a34b28-1096-4150-bafe-a46e4590377f |
| NCT03159585 | NCT03159585 | https://clinicaltrials.gov/study/NCT03159585 | https://clinicalintelligence.citeline.com/trials/details/301913?qId=e0a34b28-1096-4150-bafe-a46e4590377f |
| NCT03108495 | NCT03108495 | https://clinicaltrials.gov/study/NCT03108495 | https://clinicalintelligence.citeline.com/trials/details/299073?qId=e0a34b28-1096-4150-bafe-a46e4590377f |
| NCT03132922 | NCT03132922 | https://clinicaltrials.gov/study/NCT03132922 | https://clinicalintelligence.citeline.com/trials/details/294102?qId=e0a34b28-1096-4150-bafe-a46e4590377f |
| NCT02930993 | NCT02930993 | https://clinicaltrials.gov/study/NCT02930993 | https://clinicalintelligence.citeline.com/trials/details/288324?qId=e0a34b28-1096-4150-bafe-a46e4590377f |
| NCT02858310 | NCT02858310 | https://clinicaltrials.gov/study/NCT02858310 | https://clinicalintelligence.citeline.com/trials/details/283483?qId=e0a34b28-1096-4150-bafe-a46e4590377f |
| NCT02650986 | NCT02650986 | https://clinicaltrials.gov/study/NCT02650986 | https://clinicalintelligence.citeline.com/trials/details/271118?qId=e0a34b28-1096-4150-bafe-a46e4590377f |
| NCT02498912 | NCT02498912 | https://clinicaltrials.gov/study/NCT02498912 | https://clinicalintelligence.citeline.com/trials/details/256984?qId=e0a34b28-1096-4150-bafe-a46e4590377f |
| NCT02159716 | NCT02159716 | https://clinicaltrials.gov/study/NCT02159716 | https://clinicalintelligence.citeline.com/trials/details/210699?qId=e0a34b28-1096-4150-bafe-a46e4590377f |
| NCT02111850 | NCT02111850 | https://clinicaltrials.gov/study/NCT02111850 | https://clinicalintelligence.citeline.com/trials/details/206897?qId=e0a34b28-1096-4150-bafe-a46e4590377f |
| NCT01883297 | NCT01883297 | https://clinicaltrials.gov/study/NCT01883297 | https://clinicalintelligence.citeline.com/trials/details/188864?qId=e0a34b28-1096-4150-bafe-a46e4590377f |
| NCT01585428 | NCT01585428 | https://clinicaltrials.gov/study/NCT01585428 | https://clinicalintelligence.citeline.com/trials/details/166586?qId=e0a34b28-1096-4150-bafe-a46e4590377f |
| NCT01567891 | NCT01567891 | https://clinicaltrials.gov/study/NCT01567891 | https://clinicalintelligence.citeline.com/trials/details/165120?qId=e0a34b28-1096-4150-bafe-a46e4590377f |
| 154752 |  | http://66.147.244.217/~genesix6/wp//wp-content/uploads/2011/09/Fact_Sheet.pdf; http://66.147.244.217/~genesix6/wp//wp-content/uploads/2011/11/Genesis_Biopharma_Overview_November_2011_FINAL.pdf; http://www.businesswire.com/news/home/20130725005516/en/Genesis-Biopharma-Announces-Completion-Merger-Lion-Biotechnologies; http://www.genesis-biopharma.com/?page_id=113; http://www.sec.gov/Archives/edgar/data/1425205/000114420412018611/v307730_10k.htm | https://clinicalintelligence.citeline.com/trials/details/154752?qId=e0a34b28-1096-4150-bafe-a46e4590377f |
| NCT01174121 | NCT01174121 | https://clinicaltrials.gov/study/NCT01174121 | https://clinicalintelligence.citeline.com/trials/details/132388?qId=e0a34b28-1096-4150-bafe-a46e4590377f |
| 1772 |  | http://www.cancer.gov/clinicaltrials/view_clinicaltrials.aspx?version=healthprofessional&cdrid=64912&protocolsearchid=436448 | https://clinicalintelligence.citeline.com/trials/details/1772?qId=e0a34b28-1096-4150-bafe-a46e4590377f |
| NCT05686226 | NCT05686226 | https://clinicaltrials.gov/study/NCT05686226 |  |
| NCT05639972 | NCT05639972 | https://clinicaltrials.gov/study/NCT05639972 |  |
| NCT07617753 | NCT07617753 | https://clinicaltrials.gov/study/NCT07617753 |  |
| NCT07589543 | NCT07589543 | https://clinicaltrials.gov/study/NCT07589543 |  |
| NCT07410494 | NCT07410494 | https://clinicaltrials.gov/study/NCT07410494 |  |
| NCT07480954 | NCT07480954 | https://clinicaltrials.gov/study/NCT07480954 |  |
| NCT05776355 | NCT05776355 | https://clinicaltrials.gov/study/NCT05776355 |  |
| NCT05410717 | NCT05410717 | https://clinicaltrials.gov/study/NCT05410717 |  |
| NCT06215950 | NCT06215950 | https://clinicaltrials.gov/study/NCT06215950 |  |
| NCT03692637 | NCT03692637 | https://clinicaltrials.gov/study/NCT03692637 |  |
| NCT05225363 | NCT05225363 | https://clinicaltrials.gov/study/NCT05225363 |  |
| NCT07435701 | NCT07435701 | https://clinicaltrials.gov/study/NCT07435701 |  |
| NCT05922930 | NCT05922930 | https://clinicaltrials.gov/study/NCT05922930 |  |
| NCT05856643 | NCT05856643 | https://clinicaltrials.gov/study/NCT05856643 |  |
| NCT07410676 | NCT07410676 | https://clinicaltrials.gov/study/NCT07410676 |  |
| NCT05963100 | NCT05963100 | https://clinicaltrials.gov/study/NCT05963100 |  |
| NCT02118285 | NCT02118285 | https://clinicaltrials.gov/study/NCT02118285 |  |
| NCT03691376 | NCT03691376 | https://clinicaltrials.gov/study/NCT03691376 |  |
| NCT01583686 | NCT01583686 | https://clinicaltrials.gov/study/NCT01583686 |  |
| NCT03213964 | NCT03213964 | https://clinicaltrials.gov/study/NCT03213964 |  |
| NCT01105650 | NCT01105650 | https://clinicaltrials.gov/study/NCT01105650 |  |
| NCT00652899 | NCT00652899 | https://clinicaltrials.gov/study/NCT00652899 |  |
| NCT06321484 | NCT06321484 | https://clinicaltrials.gov/study/NCT06321484 |  |
| NCT07651124 | NCT07651124 | https://clinicaltrials.gov/study/NCT07651124 |  |
| NCT05518253 | NCT05518253 | https://clinicaltrials.gov/study/NCT05518253 |  |
| NCT05420545 | NCT05420545 | https://clinicaltrials.gov/study/NCT05420545 |  |
| NCT03539406 | NCT03539406 | https://clinicaltrials.gov/study/NCT03539406 |  |
| NCT07613723 | NCT07613723 | https://clinicaltrials.gov/study/NCT07613723 |  |
| NCT03356795 | NCT03356795 | https://clinicaltrials.gov/study/NCT03356795 |  |
| NCT03585764 | NCT03585764 | https://clinicaltrials.gov/study/NCT03585764 |  |
| NCT02580747 | NCT02580747 | https://clinicaltrials.gov/study/NCT02580747 |  |
| NCT07096583 | NCT07096583 | https://clinicaltrials.gov/study/NCT07096583 |  |
| NCT03634501 | NCT03634501 | https://clinicaltrials.gov/study/NCT03634501 |  |
| NCT05372692 | NCT05372692 | https://clinicaltrials.gov/study/NCT05372692 |  |
| NCT03916679 | NCT03916679 | https://clinicaltrials.gov/study/NCT03916679 |  |
| NCT03735589 | NCT03735589 | https://clinicaltrials.gov/study/NCT03735589 |  |
| NCT06469281 | NCT06469281 | https://clinicaltrials.gov/study/NCT06469281 |  |
| NCT06395844 | NCT06395844 | https://clinicaltrials.gov/study/NCT06395844 |  |
| NCT00101257 | NCT00101257 | https://clinicaltrials.gov/study/NCT00101257 |  |
| NCT03799913 | NCT03799913 | https://clinicaltrials.gov/study/NCT03799913 |  |
| NCT03054298 | NCT03054298 | https://clinicaltrials.gov/study/NCT03054298 |  |
| NCT06884345 | NCT06884345 | https://clinicaltrials.gov/study/NCT06884345 |  |
| NCT03814447 | NCT03814447 | https://clinicaltrials.gov/study/NCT03814447 |  |
| NCT02280811 | NCT02280811 | https://clinicaltrials.gov/study/NCT02280811 |  |
| NCT00019084 | NCT00019084 | https://clinicaltrials.gov/study/NCT00019084 |  |
| NCT03412877 | NCT03412877 | https://clinicaltrials.gov/study/NCT03412877 |  |
| NCT02713984 | NCT02713984 | https://clinicaltrials.gov/study/NCT02713984 |  |
| NCT06284226 | NCT06284226 | https://clinicaltrials.gov/study/NCT06284226 |  |
| NCT02457650 | NCT02457650 | https://clinicaltrials.gov/study/NCT02457650 |  |
| NCT06885697 | NCT06885697 | https://clinicaltrials.gov/study/NCT06885697 |  |
| NCT05592626 | NCT05592626 | https://clinicaltrials.gov/study/NCT05592626 |  |
| NCT02482090 | NCT02482090 | https://clinicaltrials.gov/study/NCT02482090 |  |
| NCT06626256 | NCT06626256 | https://clinicaltrials.gov/study/NCT06626256 |  |
| NCT05296564 | NCT05296564 | https://clinicaltrials.gov/study/NCT05296564 |  |
| NCT02153905 | NCT02153905 | https://clinicaltrials.gov/study/NCT02153905 |  |
| JPRN-jRCTc031200283 | JPRN-jRCTc031200283 | https://jrct.mhlw.go.jp/latest-detail/jRCTc031200283 | https://jrct.mhlw.go.jp/latest-detail/jRCTc031200283 |
| ChiCTR1800019780 | ChiCTR1800019780 | http://www.chictr.org.cn/showproj.aspx?proj=33241 | http://www.chictr.org.cn/showproj.aspx?proj=33241 |

# **S2B. Twelve records excluded after therapeutic-scope review**

**Note:** *All 12 records were excluded from the primary analysis to restrict the landscape to therapeutic ACT trials for invasive gynecologic malignancies. The original 223-record audit set is retained for reproducibility.*

| **Trial ID** | **Public registry ID** | **Registry type** | **Registry URL** | **Discovery source** | **Trial title** | **Primary exclusion reason** |
| --- | --- | --- | --- | --- | --- | --- |
| NCT06431100 | NCT06431100 | ClinicalTrials.gov | https://clinicaltrials.gov/study/NCT06431100 | Both | Clinical Study on the Safety and Efficacy of GK01 Autologous Tumor-reactive T Cells (TRT) in Patients With Advanced Solid Tumors | The record describes TCR-T exposure but is registered as an observational study and does not prospectively administer a new ACT intervention within the registered protocol. |
| NCT06976333 | NCT06976333 | ClinicalTrials.gov | https://clinicaltrials.gov/study/NCT06976333 | ClinicalTrials.gov | Tumor-Infiltrating Lymphocytes in Endometrial Cancer | TIL percentage and imaging features are measured; no cellular product is administered. |
| NCT04411134 | NCT04411134 | ClinicalTrials.gov | https://clinicaltrials.gov/study/NCT04411134 | ClinicalTrials.gov | E7 TCR T Cell Immunotherapy for High-Grade Cervical Intraepithelial Neoplasia | The treated condition is high-grade cervical intraepithelial neoplasia rather than invasive cancer. |
| NCT05292859 | NCT05292859 | ClinicalTrials.gov | https://clinicaltrials.gov/study/NCT05292859 | ClinicalTrials.gov | Long-Term Follow-up Study of Subjects Treated With Autologous T Cells Using the Sleeping Beauty System to Express TCRs | Subjects were treated in a prior protocol; this record adds no new cell-therapy administration. |
| NCT04106167 | NCT04106167 | ClinicalTrials.gov | https://clinicaltrials.gov/study/NCT04106167 | ClinicalTrials.gov | Long-term, Non-interventional, Observational Study Following Treatment With Fate Therapeutics FT500 Cellular Immunotherapy | The registry explicitly describes observational follow-up after prior FT500 treatment. |
| NCT00173290 | NCT00173290 | ClinicalTrials.gov | https://clinicaltrials.gov/study/NCT00173290 | ClinicalTrials.gov | Cytokine Regulation of Natural Killer Receptors in Inhibiting Activated T Cell Function | The record studies NK-receptor biology and lists no administered intervention. |
| NCT03922776 | NCT03922776 | ClinicalTrials.gov | https://clinicaltrials.gov/study/NCT03922776 | ClinicalTrials.gov | Phenotypic Characterization Tumor-infiltrating Lymphocytes at Diagnosis and After Chemotherapy in Ovarian Cancer | The only intervention is blood/sample collection for TIL phenotyping; no ACT is administered. |
| NCT05124743 | NCT05124743 | ClinicalTrials.gov | https://clinicaltrials.gov/study/NCT05124743 | ClinicalTrials.gov | HLA Typing & Tumor Neoantigen Identification for Phase I/II Study of Autologous TCR-T Cells in Subjects With Solid Tumors | HLA typing and neoantigen identification screen patients for a separate treatment protocol. |
| NCT02522585 | NCT02522585 | ClinicalTrials.gov | https://clinicaltrials.gov/study/NCT02522585 | ClinicalTrials.gov | p16 and Ki-67 Stainings and Natural Killer (NK) Cells in CIN-II Management | Observational conservative management study; NK cells are measured, not administered. |
| NCT03937791 | NCT03937791 | ClinicalTrials.gov | https://clinicaltrials.gov/study/NCT03937791 | ClinicalTrials.gov | Immunotherapy With E7 T Cell Receptor T Cells for Vulvar High-Grade Squamous Intraepithelial Lesions | The treated condition is vulvar high-grade squamous intraepithelial lesion rather than invasive cancer. |
| NCT04509726 | NCT04509726 | ClinicalTrials.gov | https://clinicaltrials.gov/study/NCT04509726 | ClinicalTrials.gov | EBV Specific Cytokine Secreting TCR-T Cells in the Treatment of EBV-Positive Metastatic/Refractory Nasopharyngeal Carcinoma | The registry record is restricted to nasopharyngeal carcinoma. |
| NCT04981119 | NCT04981119 | ClinicalTrials.gov | https://clinicaltrials.gov/study/NCT04981119 | ClinicalTrials.gov | Solid Tumor Analysis for HLA Loss of Heterozygosity (LOH) and Apheresis for CAR T- Cell Manufacturing | Apheresis and sequencing support possible later CAR-T enrollment; no CAR-T product is administered in this record. |
