# Supplementary material for: Clinical trial landscape and translational priorities for adoptive T-cell and NK-cell therapies in gynecologic malignancies
Source: Front Immunol. 2026 Sep 28;17:1936815. doi: 10.3389/fimmu.2026.1936815 (PMC13655096; doi:10.3389/fimmu.2026.1936815)
Supplement: Supplementary file 3 [file Table3.docx]

**Supplementary Table S3**

# **S3A. Aggregated respondent profile**

**Note:** *The questionnaire did not specifically ascertain Phase I/II trial experience, institutional affiliation, or institution count.*

| **Profile domain** | **Category** | **Count, n** | **Denominator, N** | **Percent** |
| --- | --- | --- | --- | --- |
| Primary field | Gynecologic oncology | 7 | 25 | 28.0 |
|  | Medical oncology | 5 | 25 | 20.0 |
|  | Cellular therapy / CAR-T / TCR-T / NK-cell therapy | 5 | 25 | 20.0 |
|  | Tumor immunotherapy | 3 | 25 | 12.0 |
|  | Clinical trial design | 3 | 25 | 12.0 |
|  | Translational medicine | 2 | 25 | 8.0 |
| Current role | Clinician | 11 | 25 | 44.0 |
|  | Clinical researcher | 6 | 25 | 24.0 |
|  | Clinical trial research staff | 3 | 25 | 12.0 |
|  | Basic researcher | 2 | 25 | 8.0 |
|  | Translational medicine researcher | 2 | 25 | 8.0 |
|  | Industry R&D or translational staff | 1 | 25 | 4.0 |
| Years of relevant experience | 5–10 years | 13 | 25 | 52.0 |
|  | >10 years | 9 | 25 | 36.0 |
|  | 3–5 years | 3 | 25 | 12.0 |
| Main work region | Mainland China | 16 | 25 | 64.0 |
|  | Europe | 3 | 25 | 12.0 |
|  | Other Asian regions | 3 | 25 | 12.0 |
|  | North America | 2 | 25 | 8.0 |
|  | Hong Kong, Macao, or Taiwan, China | 1 | 25 | 4.0 |
| Relevant experience (multiple selection permitted) | CAR-T / TCR-T / NK / TIL research | 5 | 25 | 20.0 |
|  | Clinical trial design or management | 15 | 25 | 60.0 |
|  | Clinical research in gynecologic malignancies | 15 | 25 | 60.0 |
|  | Cellular therapy research | 18 | 25 | 72.0 |
|  | Tumor immunology research | 12 | 25 | 48.0 |
|  | Translational medicine research | 10 | 25 | 40.0 |

# **S3B. Item-level survey results — Part 1 of 2: summary statistics**

**Note:** *All item-level denominators are shown. Median, quartiles, IQR, top-box n/N, and full score distributions are preferred for the 1–5 ordinal items. The mean is retained only as a labelled descriptive statistic for continuity with Figure 7.*

| **Order** | **Questionnaire variable** | **Domain** | **Item** | **Valid N** | **Median** | **Q1** | **Q3** | **IQR** | **Top-box (4–5), n/N** | **Top-box (4–5), %** | **Mean (descriptive only)** |
| --- | --- | --- | --- | --- | --- | --- | --- | --- | --- | --- | --- |
| 1 | q6_early_stage | Current-state appraisal | Clinical development remains early stage | 25 | 4.0 | 4.0 | 4.0 | 4-4 | 23/25 | 92.0 | 4.16 |
| 2 | q7_basket_dependence |  | Development depends heavily on basket trials and lacks disease-specific trials | 25 | 4.0 | 3.0 | 4.0 | 3-4 | 18/25 | 72.0 | 3.84 |
| 3 | q8_imbalance_targets_diseases_modalities |  | Targets, disease types, and treatment modalities are unevenly distributed | 25 | 4.0 | 3.0 | 4.0 | 3-4 | 18/25 | 72.0 | 3.88 |
| 4 | q9_lack_effective_targets | Development barriers | Lack of well-defined effective targets | 25 | 4.0 | 4.0 | 4.0 | 4-4 | 25/25 | 100.0 | 4.24 |
| 5 | q9_tumor_heterogeneity |  | Tumour heterogeneity | 25 | 4.0 | 4.0 | 4.0 | 4-4 | 21/25 | 84.0 | 3.96 |
| 6 | q9_immunosuppressive_tme |  | Immunosuppressive tumour microenvironment | 25 | 4.0 | 4.0 | 5.0 | 4-5 | 24/25 | 96.0 | 4.36 |
| 7 | q9_insufficient_cell_infiltration |  | Insufficient cellular infiltration | 25 | 4.0 | 3.0 | 4.0 | 3-4 | 18/25 | 72.0 | 3.92 |
| 8 | q9_insufficient_expansion_persistence |  | Insufficient expansion and persistence | 25 | 4.0 | 3.0 | 4.0 | 3-4 | 16/25 | 64.0 | 3.68 |
| 9 | q9_high_manufacturing_cost_complexity |  | High manufacturing cost and complexity | 25 | 4.0 | 3.0 | 4.0 | 3-4 | 18/25 | 72.0 | 3.76 |
| 10 | q9_patient_recruitment_difficulty |  | Patient-recruitment difficulty | 25 | 4.0 | 4.0 | 4.0 | 4-4 | 21/25 | 84.0 | 3.96 |
| 11 | q9_lack_standardized_endpoints |  | Lack of standardized clinical endpoints | 25 | 4.0 | 4.0 | 4.0 | 4-4 | 20/25 | 80.0 | 3.96 |
| 12 | q9_crs_icans_safety_management |  | CRS/ICANS and safety-management challenges | 25 | 4.0 | 3.0 | 4.0 | 3-4 | 16/25 | 64.0 | 3.68 |
| 13 | q9_lack_gynecologic_specific_cohorts |  | Lack of gynecologic-specific cohorts | 25 | 4.0 | 4.0 | 5.0 | 4-5 | 24/25 | 96.0 | 4.36 |
| 14 | q10_ovarian_cancer | Disease priorities | Ovarian cancer | 25 | 5.0 | 4.0 | 5.0 | 4-5 | 25/25 | 100.0 | 4.56 |
| 15 | q10_cervical_cancer |  | Cervical cancer | 25 | 4.0 | 4.0 | 5.0 | 4-5 | 24/25 | 96.0 | 4.28 |
| 16 | q10_endometrial_cancer |  | Endometrial cancer | 25 | 4.0 | 3.0 | 4.0 | 3-4 | 17/25 | 68.0 | 3.8 |
| 17 | q10_uterine_sarcoma |  | Uterine sarcoma | 25 | 3.0 | 3.0 | 3.0 | 3-3 | 5/25 | 20.0 | 3.04 |
| 18 | q10_vulvar_cancer |  | Vulvar cancer | 25 | 2.0 | 2.0 | 3.0 | 2-3 | 1/25 | 4.0 | 2.36 |
| 19 | q10_vaginal_cancer |  | Vaginal cancer | 25 | 2.0 | 2.0 | 2.0 | 2-2 | 0/25 | 0.0 | 2.16 |
| 20 | q10_fallopian_tube_cancer |  | Fallopian-tube cancer | 25 | 4.0 | 4.0 | 4.0 | 4-4 | 19/25 | 76.0 | 3.84 |
| 21 | q10_primary_peritoneal_cancer |  | Primary peritoneal cancer | 25 | 4.0 | 4.0 | 4.0 | 4-4 | 19/25 | 76.0 | 3.8 |
| 22 | q11_car_t | Modality priorities | CAR-T-cell therapy | 25 | 4.0 | 4.0 | 4.0 | 4-4 | 23/25 | 92.0 | 4.16 |
| 23 | q11_tcr_t |  | TCR-T-cell therapy | 25 | 4.0 | 4.0 | 5.0 | 4-5 | 24/25 | 96.0 | 4.4 |
| 24 | q11_til_therapy |  | Tumour-infiltrating lymphocyte therapy | 25 | 5.0 | 4.0 | 5.0 | 4-5 | 23/25 | 92.0 | 4.44 |
| 25 | q11_nk_cell_therapy |  | NK-cell therapy | 25 | 4.0 | 4.0 | 4.0 | 4-4 | 19/25 | 76.0 | 3.8 |
| 26 | q11_car_nk |  | CAR-NK-cell therapy | 25 | 4.0 | 4.0 | 5.0 | 4-5 | 23/25 | 92.0 | 4.2 |
| 27 | q11_multi_target_car_t |  | Multi-target CAR-T-cell therapy | 25 | 4.0 | 4.0 | 5.0 | 4-5 | 21/25 | 84.0 | 4.16 |
| 28 | q11_cell_therapy_plus_checkpoint_inhibitor |  | Cell therapy plus immune-checkpoint inhibition | 25 | 4.0 | 4.0 | 4.0 | 4-4 | 25/25 | 100.0 | 4.2 |
| 29 | q11_cell_therapy_plus_chemo_radiotherapy |  | Cell therapy plus chemotherapy/radiotherapy | 25 | 3.0 | 3.0 | 4.0 | 3-4 | 11/25 | 44.0 | 3.36 |
| 30 | q11_cell_therapy_plus_targeted_therapy |  | Cell therapy plus targeted therapy | 25 | 4.0 | 3.0 | 4.0 | 3-4 | 17/25 | 68.0 | 3.72 |
| 31 | q12_msln | Target priorities | MSLN | 25 | 5.0 | 4.0 | 5.0 | 4-5 | 25/25 | 100.0 | 4.56 |
| 32 | q12_hpv_e6_e7 |  | HPV E6/E7 | 25 | 4.0 | 4.0 | 5.0 | 4-5 | 23/25 | 92.0 | 4.28 |
| 33 | q12_her2 |  | HER2 | 25 | 4.0 | 3.0 | 4.0 | 3-4 | 15/25 | 60.0 | 3.72 |
| 34 | q12_folr1 |  | FOLR1/FRalpha | 25 | 4.0 | 4.0 | 4.0 | 4-4 | 24/25 | 96.0 | 4.2 |
| 35 | q12_ny_eso_1 |  | NY-ESO-1 | 25 | 3.0 | 3.0 | 4.0 | 3-4 | 10/25 | 40.0 | 3.36 |
| 36 | q12_muc16_ca125 |  | MUC16/CA125 | 25 | 4.0 | 4.0 | 4.0 | 4-4 | 23/25 | 92.0 | 4.08 |
| 37 | q12_b7_h3 |  | B7-H3 | 25 | 4.0 | 3.0 | 4.0 | 3-4 | 18/25 | 72.0 | 3.76 |
| 38 | q12_claudin_6 |  | Claudin 6 | 25 | 3.0 | 3.0 | 4.0 | 3-4 | 9/25 | 36.0 | 3.28 |
| 39 | q12_patient_specific_neoantigen |  | Patient-specific neoantigens | 25 | 4.0 | 4.0 | 5.0 | 4-5 | 22/25 | 88.0 | 4.16 |
| 40 | q12_future_new_targets |  | Other future targets | 25 | 4.0 | 4.0 | 4.0 | 4-4 | 22/25 | 88.0 | 4.0 |
| 41 | q13_increase_gynecologic_specific_trials | Trial-design priorities | Increase gynecologic-specific clinical trials | 25 | 4.0 | 4.0 | 5.0 | 4-5 | 24/25 | 96.0 | 4.36 |
| 42 | q13_reduce_basket_trial_dependence |  | Reduce dependence on basket trials | 25 | 4.0 | 4.0 | 4.0 | 4-4 | 23/25 | 92.0 | 4.12 |
| 43 | q13_report_cell_product_information |  | Report cell-product and manufacturing information | 25 | 4.0 | 4.0 | 5.0 | 4-5 | 24/25 | 96.0 | 4.4 |
| 44 | q13_report_target_information |  | Report target information | 25 | 5.0 | 4.0 | 5.0 | 4-5 | 25/25 | 100.0 | 4.52 |
| 45 | q13_unify_response_assessment |  | Standardize response assessment | 25 | 4.0 | 4.0 | 4.0 | 4-4 | 20/25 | 80.0 | 3.88 |
| 46 | q13_strengthen_long_term_follow_up |  | Strengthen long-term follow-up | 25 | 4.0 | 4.0 | 4.0 | 4-4 | 19/25 | 76.0 | 3.8 |
| 47 | q13_increase_biomarker_analysis |  | Increase biomarker analyses | 25 | 4.0 | 4.0 | 5.0 | 4-5 | 25/25 | 100.0 | 4.4 |
| 48 | q13_standardize_crs_icans_monitoring |  | Standardize CRS/ICANS monitoring | 25 | 4.0 | 4.0 | 4.0 | 4-4 | 20/25 | 80.0 | 3.88 |
| 49 | q13_report_expansion_persistence |  | Report cellular expansion and persistence | 25 | 4.0 | 4.0 | 4.0 | 4-4 | 24/25 | 96.0 | 4.12 |
| 50 | q13_improve_registry_completeness |  | Improve completeness of registry records | 25 | 5.0 | 4.0 | 5.0 | 4-5 | 25/25 | 100.0 | 4.52 |

# **S3B. Item-level survey results — Part 2 of 2: full score distributions**

| **Order** | **Questionnaire variable** | **Domain** | **Item** | **Valid N** | **Score 1, n** | **Score 2, n** | **Score 3, n** | **Score 4, n** | **Score 5, n** |
| --- | --- | --- | --- | --- | --- | --- | --- | --- | --- |
| 1 | q6_early_stage | Current-state appraisal | Clinical development remains early stage | 25 | 0 | 0 | 2 | 17 | 6 |
| 2 | q7_basket_dependence |  | Development depends heavily on basket trials and lacks disease-specific trials | 25 | 0 | 0 | 7 | 15 | 3 |
| 3 | q8_imbalance_targets_diseases_modalities |  | Targets, disease types, and treatment modalities are unevenly distributed | 25 | 0 | 0 | 7 | 14 | 4 |
| 4 | q9_lack_effective_targets | Development barriers | Lack of well-defined effective targets | 25 | 0 | 0 | 0 | 19 | 6 |
| 5 | q9_tumor_heterogeneity |  | Tumour heterogeneity | 25 | 0 | 0 | 4 | 18 | 3 |
| 6 | q9_immunosuppressive_tme |  | Immunosuppressive tumour microenvironment | 25 | 0 | 0 | 1 | 14 | 10 |
| 7 | q9_insufficient_cell_infiltration |  | Insufficient cellular infiltration | 25 | 0 | 0 | 7 | 13 | 5 |
| 8 | q9_insufficient_expansion_persistence |  | Insufficient expansion and persistence | 25 | 0 | 0 | 9 | 15 | 1 |
| 9 | q9_high_manufacturing_cost_complexity |  | High manufacturing cost and complexity | 25 | 0 | 0 | 7 | 17 | 1 |
| 10 | q9_patient_recruitment_difficulty |  | Patient-recruitment difficulty | 25 | 0 | 1 | 3 | 17 | 4 |
| 11 | q9_lack_standardized_endpoints |  | Lack of standardized clinical endpoints | 25 | 0 | 0 | 5 | 16 | 4 |
| 12 | q9_crs_icans_safety_management |  | CRS/ICANS and safety-management challenges | 25 | 0 | 0 | 9 | 15 | 1 |
| 13 | q9_lack_gynecologic_specific_cohorts |  | Lack of gynecologic-specific cohorts | 25 | 0 | 0 | 1 | 14 | 10 |
| 14 | q10_ovarian_cancer | Disease priorities | Ovarian cancer | 25 | 0 | 0 | 0 | 11 | 14 |
| 15 | q10_cervical_cancer |  | Cervical cancer | 25 | 0 | 0 | 1 | 16 | 8 |
| 16 | q10_endometrial_cancer |  | Endometrial cancer | 25 | 0 | 0 | 8 | 14 | 3 |
| 17 | q10_uterine_sarcoma |  | Uterine sarcoma | 25 | 0 | 4 | 16 | 5 | 0 |
| 18 | q10_vulvar_cancer |  | Vulvar cancer | 25 | 2 | 13 | 9 | 1 | 0 |
| 19 | q10_vaginal_cancer |  | Vaginal cancer | 25 | 2 | 17 | 6 | 0 | 0 |
| 20 | q10_fallopian_tube_cancer |  | Fallopian-tube cancer | 25 | 0 | 0 | 6 | 17 | 2 |
| 21 | q10_primary_peritoneal_cancer |  | Primary peritoneal cancer | 25 | 0 | 0 | 6 | 18 | 1 |
| 22 | q11_car_t | Modality priorities | CAR-T-cell therapy | 25 | 0 | 0 | 2 | 17 | 6 |
| 23 | q11_tcr_t |  | TCR-T-cell therapy | 25 | 0 | 0 | 1 | 13 | 11 |
| 24 | q11_til_therapy |  | Tumour-infiltrating lymphocyte therapy | 25 | 0 | 0 | 2 | 10 | 13 |
| 25 | q11_nk_cell_therapy |  | NK-cell therapy | 25 | 0 | 0 | 6 | 18 | 1 |
| 26 | q11_car_nk |  | CAR-NK-cell therapy | 25 | 0 | 0 | 2 | 16 | 7 |
| 27 | q11_multi_target_car_t |  | Multi-target CAR-T-cell therapy | 25 | 0 | 0 | 4 | 13 | 8 |
| 28 | q11_cell_therapy_plus_checkpoint_inhibitor |  | Cell therapy plus immune-checkpoint inhibition | 25 | 0 | 0 | 0 | 20 | 5 |
| 29 | q11_cell_therapy_plus_chemo_radiotherapy |  | Cell therapy plus chemotherapy/radiotherapy | 25 | 0 | 4 | 10 | 9 | 2 |
| 30 | q11_cell_therapy_plus_targeted_therapy |  | Cell therapy plus targeted therapy | 25 | 0 | 0 | 8 | 16 | 1 |
| 31 | q12_msln | Target priorities | MSLN | 25 | 0 | 0 | 0 | 11 | 14 |
| 32 | q12_hpv_e6_e7 |  | HPV E6/E7 | 25 | 0 | 0 | 2 | 14 | 9 |
| 33 | q12_her2 |  | HER2 | 25 | 0 | 0 | 10 | 12 | 3 |
| 34 | q12_folr1 |  | FOLR1/FRalpha | 25 | 0 | 0 | 1 | 18 | 6 |
| 35 | q12_ny_eso_1 |  | NY-ESO-1 | 25 | 0 | 2 | 13 | 9 | 1 |
| 36 | q12_muc16_ca125 |  | MUC16/CA125 | 25 | 0 | 0 | 2 | 19 | 4 |
| 37 | q12_b7_h3 |  | B7-H3 | 25 | 0 | 0 | 7 | 17 | 1 |
| 38 | q12_claudin_6 |  | Claudin 6 | 25 | 0 | 2 | 14 | 9 | 0 |
| 39 | q12_patient_specific_neoantigen |  | Patient-specific neoantigens | 25 | 0 | 0 | 3 | 15 | 7 |
| 40 | q12_future_new_targets |  | Other future targets | 25 | 0 | 0 | 3 | 19 | 3 |
| 41 | q13_increase_gynecologic_specific_trials | Trial-design priorities | Increase gynecologic-specific clinical trials | 25 | 0 | 0 | 1 | 14 | 10 |
| 42 | q13_reduce_basket_trial_dependence |  | Reduce dependence on basket trials | 25 | 0 | 0 | 2 | 18 | 5 |
| 43 | q13_report_cell_product_information |  | Report cell-product and manufacturing information | 25 | 0 | 0 | 1 | 13 | 11 |
| 44 | q13_report_target_information |  | Report target information | 25 | 0 | 0 | 0 | 12 | 13 |
| 45 | q13_unify_response_assessment |  | Standardize response assessment | 25 | 0 | 0 | 5 | 18 | 2 |
| 46 | q13_strengthen_long_term_follow_up |  | Strengthen long-term follow-up | 25 | 0 | 0 | 6 | 18 | 1 |
| 47 | q13_increase_biomarker_analysis |  | Increase biomarker analyses | 25 | 0 | 0 | 0 | 15 | 10 |
| 48 | q13_standardize_crs_icans_monitoring |  | Standardize CRS/ICANS monitoring | 25 | 0 | 0 | 5 | 18 | 2 |
| 49 | q13_report_expansion_persistence |  | Report cellular expansion and persistence | 25 | 0 | 0 | 1 | 20 | 4 |
| 50 | q13_improve_registry_completeness |  | Improve completeness of registry records | 25 | 0 | 0 | 0 | 12 | 13 |

# **S3C. English translations of de-identified open-text responses**

**Note:** *Responses to Q14–Q16 were provided in Chinese and are presented here as faithful English translations under anonymous respondent IDs. No formal qualitative thematic analysis was undertaken, and no automated theme coding is used in the manuscript conclusions.*

| **Anonymous respondent ID** | **Q14. Key issues for the next 3–5 years (English translation)** | **Q15. Promising modalities or targets (English translation)** | **Q16. Trial-design suggestions (English translation)** |
| --- | --- | --- | --- |
| EXP001 | The solid-tumour microenvironment remains the greatest challenge, particularly whether cells can continue to expand and retain function after entering the tumour. | HPV E6/E7-targeted TCR-T has the strongest biological rationale in cervical cancer because viral antigens offer greater disease specificity. | Trial registrations should specify the cell source, engineering method, target, dose, and combination-treatment regimen. |
| EXP002 | Gynecologic cancers require clearer target stratification; experience from solid tumours cannot simply be transferred directly. | TIL therapy in some gynecologic cancers is more closely aligned with existing solid-tumour experience and may therefore yield clinical signals sooner. | Gynecologic cancer-specific cohorts should be established whenever possible, with basket-trial results treated as exploratory rather than definitive evidence. |
| EXP003 | There are too few disease-specific cohorts, and many signals are diluted in basket trials; future clinical designs need to be more focused. | MSLN remains worthy of priority investigation, but therapeutic-window issues arising from normal-tissue expression and heterogeneity need to be addressed. | Efficacy assessment should be standardized, with simultaneous reporting of RECIST, immune-related response criteria, and duration of response. |
| EXP004 | I believe the key issues are the target safety window and tumour heterogeneity, particularly in patients with recurrent ovarian cancer. | FOLR1 has a clear translational basis in ovarian cancer and, if combined with cell-product optimization, may provide a relatively clear development path. | Long-term follow-up and delayed toxicity must be incorporated into protocols, particularly for genetically modified cell products. |
| EXP005 | There is a mismatch between manufacturing time and patient condition; heavily pretreated patients often cannot wait for an optimal cell product. | CAR-NK is attractive for its safety and off-the-shelf potential, but more evidence is needed regarding infiltration into solid tumours. | Tissue sampling and peripheral-blood monitoring before and after treatment should be increased to explain cellular expansion and microenvironmental changes. |
| EXP006 | Cellular expansion, persistence, and tumour infiltration should be core translational endpoints in early-phase trials. | MUC16/CA125 is highly attractive in ovarian cancer, but heterogeneity in target expression and antigen shedding require careful management. | Registry records should not use only terms such as “immunotherapy” or “cellular therapy”; the product type should be specified. |
| EXP007 | Viral antigens are relatively well defined in cervical cancer, but improving response durability remains a challenge. | Multi-target CAR-T may help reduce antigen escape, but complexity and safety will become new challenges. | Safety reporting should distinguish CRS, ICANS, haematologic toxicities, and other organ toxicities rather than describe them collectively. |
| EXP008 | Gynecologic cancer cell therapy should not focus only on novel targets; eligibility criteria and combination strategies also need improvement. | TCR-T targeting NY-ESO-1 or other tumour antigens remains valuable, although patient selection and HLA restriction will limit broader use. | Patient-selection criteria should be reported more transparently, including target-assay methods, positivity thresholds, and specimen sources. |
| EXP009 | Patient recruitment will be a practical challenge, particularly because of constraints related to tissue acquisition, prior lines of therapy, and performance status. | Cell therapy combined with immune checkpoint inhibitors deserves priority testing because microenvironmental suppression may be a central barrier. | For rare gynecologic cancers, multicentre collaboration and prespecified stratification should be used instead of fragmented, case-by-case enrolment. |
| EXP010 | Over the next three to five years, the field most needs a set of interpretable early clinical signals rather than continued fragmented exploration. | I am more optimistic about TIL combined with immunomodulatory strategies than about relying on CAR-T alone to achieve a breakthrough in solid tumours. | Statistical designs can be more pragmatic; early-phase trials should emphasize feasibility, successful product manufacture, and translational endpoints. |
| EXP011 | Safety is not the only barrier, but reporting of CRS, neurotoxicity, and long-term follow-up still needs standardization. | HPV-related targets have advantages in cervical cancer and some vulvar/vaginal cancers, but the applicable population must be clearly defined. | A minimum registration reporting checklist should be established to reduce missing information on targets, cell products, and endpoints. |
| EXP012 | Different gynecologic cancers should be considered separately; the priorities for ovarian, cervical, and endometrial cancers are not the same. | B7-H3 is an emerging target worth monitoring, but its safety and expression profile in gynecologic cancers require further confirmation. | Combination trials should state the rationale for the combination rather than mechanically adding treatments simply because monotherapy has limited efficacy. |
| EXP013 | I am more concerned about biomarker selection; without predictive markers, subsequent clinical development will be very inefficient. | Patient-specific neoantigens are a cutting-edge direction, but cost, turnaround time, and scalability limit near-term clinical adoption. | Manufacturing failure, waiting time, and bridging therapy need to be reported because they are highly relevant to cell therapy. |
| EXP014 | Insufficient disclosure of cell-product information makes many trials difficult to compare and reproduce even when registered. | HER2 could be explored in some uterine serous carcinomas or other subtypes, but it is not suitable for all gynecologic cancers. | Biomarker analysis plans should be prespecified; otherwise, results are often difficult to interpret. |
| EXP015 | Antigen escape and microenvironmental suppression must be addressed; a single-target strategy may be unlikely to work over the long term. | Claudin 6 has potential, but I would place it in a second tier pending more expression and safety data. | Trial design should account for prior lines of therapy and tumour burden because they can substantially affect efficacy and safety. |
| EXP016 | Enrolment in rare gynecologic cancers is difficult and will require multicentre and even cross-regional collaboration. | NK-cell therapy may be better suited to combination or maintenance strategies than to providing the entire antitumour effect as monotherapy. | Cell persistence, peak expansion, and clearance time should be reported both in registry entries and publications. |
| EXP017 | Future work should prioritize identifying which patients are genuinely likely to benefit from cell therapy rather than broadening indications. | TCR-T and TIL have the advantage of more closely reflecting endogenous antigen recognition, but product consistency still needs improvement. | CRS/ICANS monitoring standards should align with those used in haematologic-malignancy cell therapy while also accounting for the characteristics of patients with solid tumours. |
| EXP018 | The greatest current challenge is moving from feasibility studies to verifiable disease-specific evidence. | MSLN, FOLR1, and MUC16 may form a priority target set in ovarian cancer, but this requires support from molecular subtyping. | Data reporting should enable cross-study comparisons, particularly for disease-subtype and target-expression information. |
| EXP019 | Both TIL and TCR-T require better patient selection, particularly assessment of antigen expression and immune status. | Future breakthroughs may come from combining target selection with microenvironmental modification rather than from any single target. | Early studies may use adaptive designs, but the criteria triggering cohort expansion must be prespecified. |
| EXP020 | Manufacturing cost and quality-control processes will affect real-world translation; early efficacy signals alone are insufficient. | HPV E6/E7 remains the clearest direction for cervical cancer, whereas ovarian cancer will require multi-target strategies. | Patient access and cost metrics should be included in translational discussions, not only efficacy endpoints. |
| EXP021 | Clinical trial endpoints and reporting conventions need to be standardized; otherwise, cross-study comparisons are difficult. | I remain cautiously optimistic about CAR-T in gynecologic solid tumours; CAR-NK and combination strategies may be more flexible. | For virus-associated targets, the HPV genotype, assay platform, and evidence of antigen expression should be specified. |
| EXP022 | Translational research should be designed alongside the clinical trial rather than added after trial completion to explain mechanisms. | If manufacturing time can be shortened, personalized neoantigen-specific T-cell therapy may demonstrate value in small precision cohorts. | Reasons for negative or terminated trials should be disclosed because they are important for assessing the field’s actual progress. |
| EXP023 | NK or CAR-NK may be better suited to an off-the-shelf approach, but their infiltration and persistence in gynecologic solid tumours still need to be demonstrated. | FOLR1 and MSLN are more realistic near-term targets; neoantigens are more of a medium- to long-term direction. | Gynecologic oncology multidisciplinary teams and cell-therapy teams should both be involved in trial design to improve feasibility. |
| EXP024 | The key is to establish an integrated development pathway from target selection and product design to clinical endpoints. | The potential of cell therapy plus chemoradiotherapy depends on whether it can improve antigen release and immune infiltration, but toxicity must be controlled. | Registries should improve information completeness, particularly for the target, cell product, endpoint, and follow-up fields. |
| EXP025 | Without more stable gynecologic-specific cohorts, the development activity seen in registries will be difficult to translate into clinical evidence. | The most promising approach is not a single modality, but a strategy that jointly matches disease, target, and combination therapy. | Information on patient-derived tissue sources, specimen availability, and time points for translational testing should be reported to help explain reasons for failure. |

# **S3D. English-only rendering of the full questionnaire**

**Note:** *The English content is presented in the original document order. Response-option cells within a source table row are separated by vertical bars.*

| **Source element** | **English questionnaire content** |
| --- | --- |
| Paragraph | Expert Survey on Translational Priorities and Development Challenges for Adoptive T-cell and NK-cell Therapies in Gynecologic Malignancies |
| Table 1, row 1 | Questionnaire description \| This questionnaire aims to evaluate current development status, major translational challenges, future priorities, potential therapeutic modalities, target selection, and clinical trial design priorities for adoptive T-cell and NK-cell therapies in gynecologic malignancies. \| The questionnaire is intended for clinical and research experts in gynecologic oncology, tumor immunotherapy, cellular therapy, translational medicine, clinical trial design, and related fields. \| It collects expert professional opinions only and does not involve patients, biological samples, clinical interventions, or directly identifiable personal information. Results will be used for academic research in aggregate form. \| Estimated completion time: 5-8 minutes. |
| **Paragraph** | **Informed consent** |
| Paragraph | Are you aware of the purpose of this questionnaire and willing to participate in this anonymous expert survey? |
| Table 2, row 1 | Yes, I understand and agree to participate |
| Table 2, row 2 | No, I do not agree to participate |
| **Paragraph** | **Part I. Expert background** |
| Paragraph | What is your primary area of expertise? |
| Table 3, row 1 | Gynecologic oncology \| Medical oncology |
| Table 3, row 2 | Tumor immunotherapy \| Cellular therapy / CAR-T / TCR-T / NK-cell therapy |
| Table 3, row 3 | Translational medicine \| Clinical trial design |
| Table 3, row 4 | Basic immunology research \| Other: __________ |
| Paragraph | What is your current primary professional role? |
| Table 4, row 1 | Clinician \| Clinical researcher |
| Table 4, row 2 | Basic researcher \| Translational researcher |
| Table 4, row 3 | Clinical trial researcher/staff \| Industry R&D or translational staff |
| Table 4, row 4 | Other: __________ |
| Paragraph | How long have you worked in the relevant field? |
| Table 5, row 1 | <3 years \| 3-5 years |
| Table 5, row 2 | 5-10 years \| >10 years |
| Paragraph | In which region do you primarily work? |
| Table 6, row 1 | Mainland China \| Hong Kong, Macao, or Taiwan, China |
| Table 6, row 2 | Other Asian regions \| Europe |
| Table 6, row 3 | North America \| Other region: __________ |
| Paragraph | Have you participated in any of the following activities? (Multiple choices allowed; "no relevant experience" is mutually exclusive.) |
| Table 7, row 1 | Clinical research in gynecologic malignancies \| Cellular therapy research |
| Table 7, row 2 | CAR-T / TCR-T / NK / TIL-related research \| Clinical trial design or management |
| Table 7, row 3 | Tumor immunology research \| Translational medicine research |
| Table 7, row 4 | No relevant experience listed above |
| **Paragraph** | **Part II. Current development status** |
| Table 8, row 1 | Scale description \| 1 = strongly disagree; 2 = disagree; 3 = neutral; 4 = agree; 5 = strongly agree. |
| Paragraph | Adoptive cellular therapy in gynecologic malignancies remains at an early stage of clinical development. |
| Table 9, row 1 | □ 1 \| □ 2 \| □ 3 \| □ 4 \| □ 5 |
| Paragraph | Current cellular therapy research in gynecologic malignancies relies heavily on basket trials, whereas disease-specific clinical trials remain relatively limited. |
| Table 10, row 1 | □ 1 \| □ 2 \| □ 3 \| □ 4 \| □ 5 |
| Paragraph | The distribution of targets, disease types, and therapeutic modalities in current gynecologic cancer cellular therapy studies remains insufficiently balanced. |
| Table 11, row 1 | □ 1 \| □ 2 \| □ 3 \| □ 4 \| □ 5 |
| **Paragraph** | **Part III. Major developmental barriers** |
| Table 12, row 1 | Scale description \| 1 = not important; 2 = slightly important; 3 = moderately important; 4 = important; 5 = very important. |
| Paragraph | To what extent do the following factors limit the development of cellular therapy in gynecologic malignancies? |
| Table 13, row 1 | Barrier \| 1 \| 2 \| 3 \| 4 \| 5 |
| Table 13, row 2 | Lack of clearly effective therapeutic targets \| □ \| □ \| □ \| □ \| □ |
| Table 13, row 3 | Tumor heterogeneity \| □ \| □ \| □ \| □ \| □ |
| Table 13, row 4 | Immunosuppressive tumor microenvironment \| □ \| □ \| □ \| □ \| □ |
| Table 13, row 5 | Insufficient cellular infiltration \| □ \| □ \| □ \| □ \| □ |
| Table 13, row 6 | Insufficient expansion and persistence \| □ \| □ \| □ \| □ \| □ |
| Table 13, row 7 | High manufacturing cost and process complexity \| □ \| □ \| □ \| □ \| □ |
| Table 13, row 8 | Patient recruitment difficulty \| □ \| □ \| □ \| □ \| □ |
| Table 13, row 9 | Lack of standardized clinical endpoints \| □ \| □ \| □ \| □ \| □ |
| Table 13, row 10 | Safety-management challenges such as CRS / ICANS \| □ \| □ \| □ \| □ \| □ |
| Table 13, row 11 | Lack of gynecologic cancer-specific study cohorts \| □ \| □ \| □ \| □ \| □ |
| **Paragraph** | **Part IV. Priority disease settings** |
| Table 14, row 1 | Scale description \| 1 = very low; 2 = low; 3 = moderate; 4 = high; 5 = very high. |
| Paragraph | Which gynecologic malignancies are more suitable as priority disease settings for future cellular therapy development? |
| Table 15, row 1 | Disease type \| 1 \| 2 \| 3 \| 4 \| 5 |
| Table 15, row 2 | Ovarian cancer \| □ \| □ \| □ \| □ \| □ |
| Table 15, row 3 | Cervical cancer \| □ \| □ \| □ \| □ \| □ |
| Table 15, row 4 | Endometrial cancer \| □ \| □ \| □ \| □ \| □ |
| Table 15, row 5 | Uterine sarcoma \| □ \| □ \| □ \| □ \| □ |
| Table 15, row 6 | Vulvar cancer \| □ \| □ \| □ \| □ \| □ |
| Table 15, row 7 | Vaginal cancer \| □ \| □ \| □ \| □ \| □ |
| Table 15, row 8 | Fallopian tube cancer \| □ \| □ \| □ \| □ \| □ |
| Table 15, row 9 | Primary peritoneal cancer \| □ \| □ \| □ \| □ \| □ |
| **Paragraph** | **Part V. Priority cellular therapy modalities** |
| Table 16, row 1 | Scale description \| 1 = very low; 2 = low; 3 = moderate; 4 = high; 5 = very high. |
| Paragraph | How do you rate the future potential of the following cellular therapy modalities in gynecologic malignancies? |
| Table 17, row 1 | Therapeutic modality \| 1 \| 2 \| 3 \| 4 \| 5 |
| Table 17, row 2 | CAR-T-cell therapy \| □ \| □ \| □ \| □ \| □ |
| Table 17, row 3 | TCR-T-cell therapy \| □ \| □ \| □ \| □ \| □ |
| Table 17, row 4 | Tumor-infiltrating lymphocyte therapy \| □ \| □ \| □ \| □ \| □ |
| Table 17, row 5 | NK-cell therapy \| □ \| □ \| □ \| □ \| □ |
| Table 17, row 6 | CAR-NK-cell therapy \| □ \| □ \| □ \| □ \| □ |
| Table 17, row 7 | Multi-target CAR-T-cell therapy \| □ \| □ \| □ \| □ \| □ |
| Table 17, row 8 | Cellular therapy plus immune checkpoint inhibitors \| □ \| □ \| □ \| □ \| □ |
| Table 17, row 9 | Cellular therapy plus chemotherapy / radiotherapy \| □ \| □ \| □ \| □ \| □ |
| Table 17, row 10 | Cellular therapy plus targeted therapy \| □ \| □ \| □ \| □ \| □ |
| **Paragraph** | **Part VI. Potential target selection** |
| Table 18, row 1 | Scale description \| 1 = very low; 2 = low; 3 = moderate; 4 = high; 5 = very high. |
| Paragraph | How do you rate the future translational value of the following targets for cellular therapy in gynecologic malignancies? |
| Table 19, row 1 | Target \| 1 \| 2 \| 3 \| 4 \| 5 |
| Table 19, row 2 | MSLN \| □ \| □ \| □ \| □ \| □ |
| Table 19, row 3 | HPV E6 / E7 \| □ \| □ \| □ \| □ \| □ |
| Table 19, row 4 | HER2 \| □ \| □ \| □ \| □ \| □ |
| Table 19, row 5 | FOLR1 \| □ \| □ \| □ \| □ \| □ |
| Table 19, row 6 | NY-ESO-1 \| □ \| □ \| □ \| □ \| □ |
| Table 19, row 7 | MUC16 / CA125 \| □ \| □ \| □ \| □ \| □ |
| Table 19, row 8 | B7-H3 \| □ \| □ \| □ \| □ \| □ |
| Table 19, row 9 | Claudin 6 \| □ \| □ \| □ \| □ \| □ |
| Table 19, row 10 | Patient-specific neoantigens \| □ \| □ \| □ \| □ \| □ |
| Table 19, row 11 | Other future targets \| □ \| □ \| □ \| □ \| □ |
| **Paragraph** | **Part VII. Future clinical trial design priorities** |
| Table 20, row 1 | Scale description \| 1 = not important; 2 = slightly important; 3 = moderately important; 4 = important; 5 = very important. |
| Paragraph | Which aspects of future clinical trials of cellular therapy in gynecologic malignancies most need improvement? |
| Table 21, row 1 | Improvement priority \| 1 \| 2 \| 3 \| 4 \| 5 |
| Table 21, row 2 | Increase gynecologic cancer-specific trials \| □ \| □ \| □ \| □ \| □ |
| Table 21, row 3 | Reduce reliance on basket trials alone \| □ \| □ \| □ \| □ \| □ |
| Table 21, row 4 | Clearly report cell-product type and manufacturing information \| □ \| □ \| □ \| □ \| □ |
| Table 21, row 5 | Clearly report therapeutic target information \| □ \| □ \| □ \| □ \| □ |
| Table 21, row 6 | Standardize efficacy assessment criteria \| □ \| □ \| □ \| □ \| □ |
| Table 21, row 7 | Strengthen long-term follow-up \| □ \| □ \| □ \| □ \| □ |
| Table 21, row 8 | Increase biomarker analyses \| □ \| □ \| □ \| □ \| □ |
| Table 21, row 9 | Standardize safety monitoring such as CRS / ICANS \| □ \| □ \| □ \| □ \| □ |
| Table 21, row 10 | Report cellular expansion and in vivo persistence \| □ \| □ \| □ \| □ \| □ |
| Table 21, row 11 | Improve completeness of trial registration information \| □ \| □ \| □ \| □ \| □ |
| **Paragraph** | **Part VIII. Open expert comments** |
| Paragraph | What key issues should adoptive cellular therapy in gynecologic malignancies address over the next 3-5 years? |
| Paragraph | Which cellular therapy modality or target is most likely to achieve a clinical breakthrough in gynecologic malignancies, and why? |
| Paragraph | Do you have any additional suggestions regarding future clinical trial design, translational research, or data reporting standards? |
| **Paragraph** | **Closing statement** |
| Paragraph | Thank you very much for participating in this expert survey. Your professional opinions will help us better understand the developmental barriers, translational priorities, and future clinical research directions for adoptive T-cell and NK-cell therapies in gynecologic malignancies. |
| Paragraph | Thank you for your valuable time and support. |
